# Trends in access of plant biodiversity data revealed by Google Analytics

**DOI:** 10.3897/BDJ.2.e1558

**Published:** 2014-11-11

**Authors:** Timothy Mark Jones, David G. Baxter, Gregor Hagedorn, Ben Legler, Edward Gilbert, Kevin Thiele, Yalma Vargas-Rodriguez, Lowell E. Urbatsch

**Affiliations:** † Louisiana State University, Baton Rouge, United States of America; ‡ University of California, Berkeley, United States of America; § Julius Kühn-Institut, Berlin, Germany; | University of Washington Herbarium, Seattle, United States of America; ¶ Arizona State University, Phoenix, United States of America; # Western Australian Herbarium, Perth, Australia

**Keywords:** Biodiversity, big data, herbarium, Google Analytics, botany, museums, vascular plants, systematics, taxonomy, collections, digitization, web development, Kingdom Plantae

## Abstract

The amount of plant biodiversity data available via the web has exploded in the last decade, but making these data available requires a considerable investment of time and work, both vital considerations for organizations and institutions looking to validate the impact factors of these online works. Here we used Google Analytics (GA), to measure the value of this digital presence. In this paper we examine usage trends using 15 different GA accounts, spread across 451 institutions or botanical projects that comprise over five percent of the world's herbaria. They were studied at both one year and total years. User data from the sample reveal: 1) over 17 million web sessions, 2) on five primary operating systems, 3) search and direct traffic dominates with minimal impact from social media, 4) mobile and new device types have doubled each year for the past three years, 5) and web browsers, the tools we use to interact with the web, are changing. Server-side analytics differ from site to site making the comparison of their data sets difficult. However, use of Google Analytics erases the reporting heterogeneity of unique server-side analytics, as they can now be examined with a standard that provides a clarity for data-driven decisions. The knowledge gained here empowers any collection-based environment regardless of size, with metrics about usability, design, and possible directions for future development.

## Introduction

Herbaria are natural history museums that preserve collections of millions of specimens that offer a well established distributional model for a large-scale taxon (Nakrem 1996, Suppl. material 1). Traditionally, usage reports for herbaria were developed from handwritten data gathered from the requisite sign-in book common to herbaria. A standard format for these usage reports does not currently exist, because each institution developed a data set deemed useful for their specific needs. Furthermore, data included may vary over time in response to changing emphases or requirements, see for example Utah State University Herbarium Log (Suppl. material 2), or New York Botanical Garden Log (Suppl. material 3). However in recent years, herbaria have taken advantage of web resources for the sharing of information. With the rapid development of geographic information systems and inexpensive imaging technology, websites that used to provide little more than lists of specimens were modified to display distribution maps and specimen images (Gries et al. 2014). Now some herbarium websites provide access to other taxonomic resources such as nomenclatural information, identification tools (Brach and Song 2005, Dallwitz 2000, Hagedorn 2007, Jones 2013, Thiele 1993, Gries et al. 2014) and formal descriptions. Understanding how the tools are being used is crucial to planning educational, financial, and research activities.

The goal of this manuscript is twofold: to provide recommendations for current information managers and developers concerning the user interface and experience; and to provide a picture about the possible directions to take for those in-charge of the creation of information at all levels. Online plant databases can facilitate the democratization of botanical information through their availability, via open information that exceeds the speed of retrieval from a cabinet or bookshelf. Specimens, including type specimens, no longer need to be shipped back and forth across the globe; thereby limiting wear and tear to these important biodiversity objects while eliminating shipping costs. And importantly, all researchers can now share equal access globally, without travel, to a well established model at kingdom level (Stevens 2009, Sanderson et al. 2008).

Understanding how taxonomic resources now provided via the World Wide Web (WWW) are used, represents a new challenge. For this reason, presented here are collected data obtained from contributors using Google Analytics that functioned as a standard report (Fang 2007, Hasan et al. 2009, Kent et al. 2011). The data considered include: a count of sessions, country/city/network of origin, types of devices used, operating systems used, traffic distinctions between search, direct, and social, as well as returning versus new visitors. In this paper we examine Google Analytics (GA) data from several plant & fungal related websites. Documented here is the extent to which websites serving plant biodiversity data are being used. Particularly, changes that might suggest new directions that should be taken to maximize the value of the investment museums and herbaria are making in digitization efforts (Nicholas and Clark 2014, Nicholas and Clark 2013, Nicholas et al. 2013). We wished to address the following questions: are these resources effective at delivering information throughout the world? What is the breakdown of direct versus search traffic, or social; is one more important than the other? What technology are they using? Finally, can we provide a metric that quantifies the amount of botanical work being done online globally on the WWW?

### Latest user analytics

We selected GA for website usage analytics for multiple reasons: 1) It is free to use, so is widely adopted, 2) It is standardized so analytics can be compared across institutional users, and 3) GA only tracks human usage, as opposed to most server-side analytics programs which track human and robot traffic indiscriminately.

**Figure 1.**
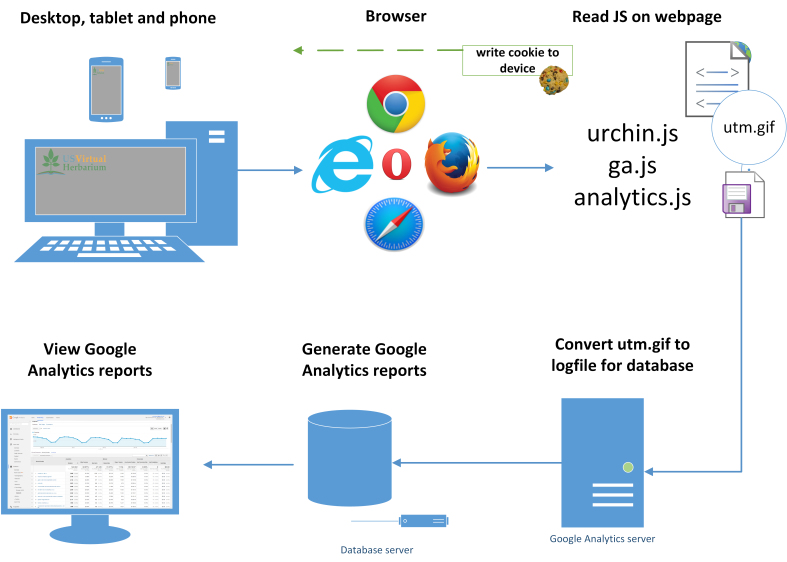
What is Google Analytics? A user directs a browser to a website that contains a tracking code. This tracking code or script leverages the information already being gathered by the browser; but then also writes a cookie back to the device that yields additional information that the browser cannot provide, such as time-on-site or page-views. The packaged set of collected data is then sent back to a Google server in the form of a GIF file. Lastly, the GIF file is then interpreted and incorporated into reports.

In order to be tracked, Google Analytics requires the inclusion of a snippet of Javascript (JS) (Fig. 1) on every webpage.

## Material and methods

Sites were selected for this study by searching Hyper Text Markup Language (HTML) source code of biodiversity websites for the presence of Google Analytics. After identifying sites of interest, Jones contacted curators, directors, and developers via email or phone. This process led to the inclusion of fifteen sites (Table 1). All calculations are based upon the full calendar year, i.e. 24 hours per day, 365 days per year. * GBIF and ON also feature animal and invertebrate data. Here both are treated with corresponding portions of data holdings concerning plant data respectively at 28% and 87% (G. Hagedorn, pers. comm., July 2014) of their session counts.

## Data resources

**Figure 2.**
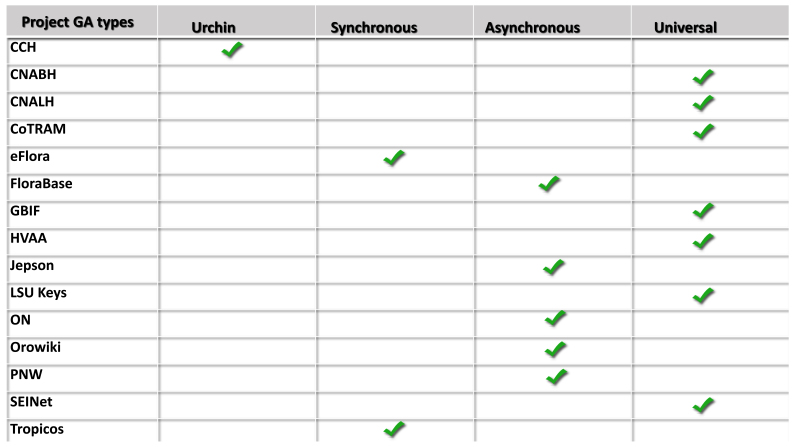
Four variants of GA are represented in this study. Urchin is the first iteration of GA, derived from software developed by Urchin Software acquired by Google in 2005. It is unique in that it employed multiple means of information gathering, using both server logs and multiple cookies. The second iteration, synchronous or traditional, released in late 2007, also used multiple cookies, plus required that the JS load in a linear fashion. Penalizing content over tracking. Asynchronous came out two years later, and allowed for faster loads of content as the webpage loads first, and GA JS loads post-content delivery. The latest variant, universal, addresses issues with mobile and the internet-of-things (emerging wearable devices and existing household appliances that can communicate via the web), as it can assimilate into reports any device that can contact a server.

A total of four types of GA resources are charted (Fig. 2) across the population. CCH and Jepson was shared via Google Sheets by Baxter with Jones. Additionaly, for the sharing of GA resources, issues arose with institutional gmail accounts often not enabled for sharing of Google additional services, e.g., John-Doe@tigers.lsu.edu fails to share data (an institutional Gmail), while John-Doe@gmail.com (regular Gmail) is successful.

## Results

**Number of sessions** – 17,198,976 sessions from inception (when each organization began tracking) were found across the 15 GA numbers (Table 2, Suppl. material 4).

**Figure 3.**
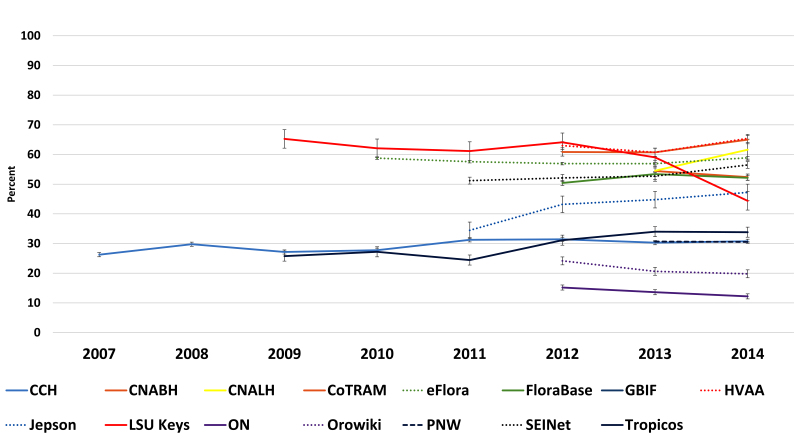
Historical bounce rates of study participants as compared year by year from January 01 to January 01 (Suppl. material 7).

**Stable bounce rates** – Bounce is defined as the user visiting the primary page only and then exiting. Bounces are not included across the statistics, as they are treated as zeros. All participants in the study show relatively stable bounce rates. See discussion (Fig. 3).

**Figure 4.**
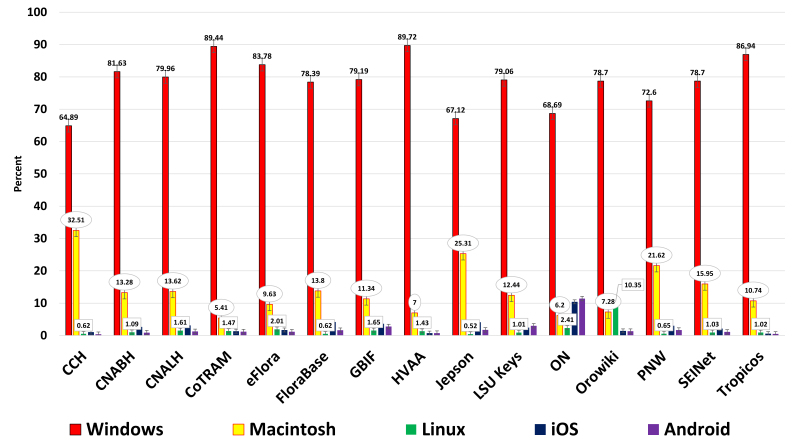
Historical operating systems to January 01, 2014 (Suppl. material 10). ON has a disproportionately high value for Android usage due to the inclusion of the same GA number for a deployed Android mobile app concerning the same material.

**Figure 5.**
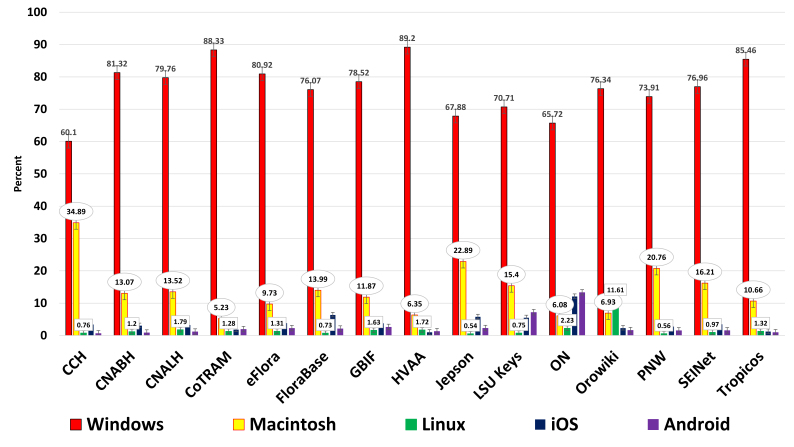
One year of operating systems from January 01, 2013 to January 01, 2014, showing same ON trend (Suppl. material 10).

**Operating systems** – Revealed five major operating systems: Windows, Macintosh, Linux, iOS, and Android (Figs 4, 5).

**Outreach** – Each site's traffic favors its country of origin but all nations, territories, and/or commonwealths are represented across the sample (Tables 3, 4).

**Mobile growth** – Phone & tablet usage is steadily increasing for all resources (Fig. 7).

**Device types** – The number of different device types has grown exponentially in recent years, from just a few types in 2010 to over 1500 in 2014 (Fig. 9).

**Browser Wars** – Five web browsers are in a slow-motion-knife-fight for dominance (Fig. 11).

**Figure 6.**
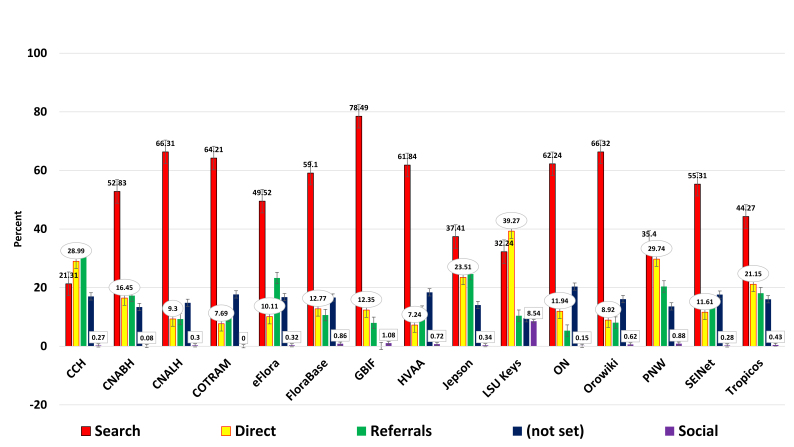
Yearly traffic* broken down by search, direct, referral, 'not set', and social (Suppl. material 14). Search makes up the lion-share of all traffic. Direct traffic is second in size overall due to people that type or bookmark. Referrals are web links posted on other websites that directly refer a web user to another site. 'Not set' is a difficult parameter to define but is probably due to: individuals blocking JS as a security measure; those using private browser settings; use of browser plugins that block JS; or may also be the result of improper GA usage by referring sites. Interestingly, social traffic remains below one percent of all traffic when examined across the entire sample. *Caveat: this data was derived from 'Acquisition, Channels' which only became available on July 25, 2013, creating an 11 month data set.

**Figure 7.**
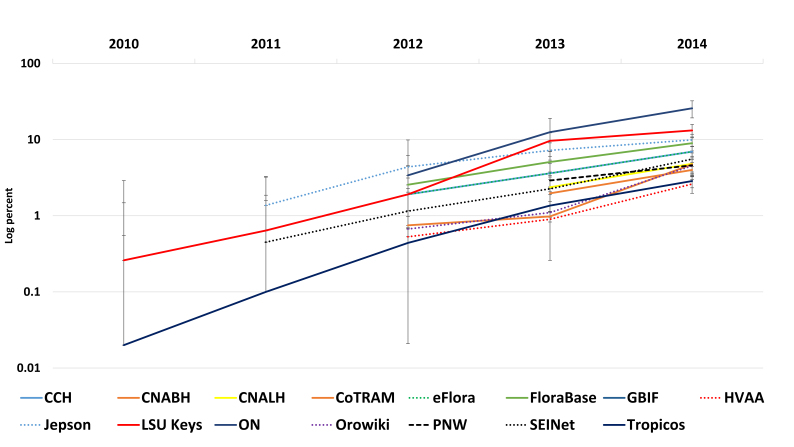
Combined phone and tablet usage by percentage at log, showing emergence of mobile in 2010 in a changing landscape of device use (see Fig. 8). Mobile makes up less than ten percent of all traffic when averaged across the sample but is growing yearly on all sites. Interestingly, these sites show significant mobile and tablet usage growth, despite primarily lacking affordances for delivery on mobile platforms (Suppl. material 8).

**Search, Direct, Referrals, and Social** – Traffic types were examined in a one year study (Fig. 6) to reveal that search, direct, and referrals are all significant contributors to traffic. Social remains at less than one percent of all traffic across the sampled websites (see Discussion).

**Figure 8.**
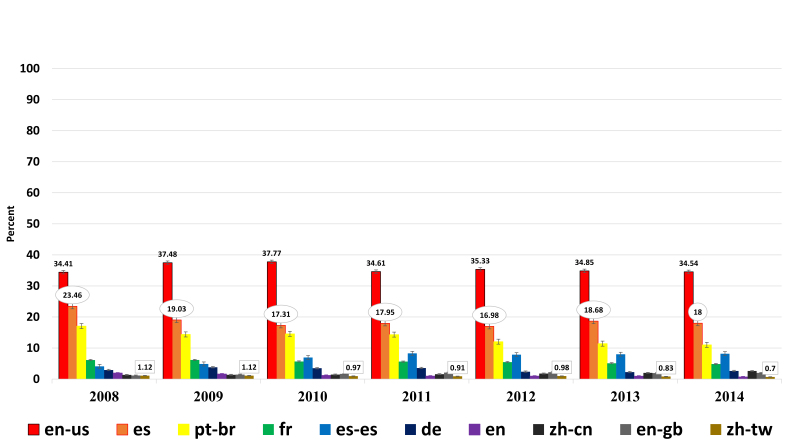
Ten top International Organization for Standardization (ISO) languages in use at Tropicos over six years; in order of percentage of usage (Suppl. materials 12, 13, 15, 16, 17, 18, 19). As only two nations websites are represented across the study, U.S.A. and Germany, the results show the expected language-of-origin dominance.
en-us English of U.S.A.es Spanishpt-br Portuguese of Brazilfr Frenches-es Spanish of Spainde Germanen Englishzh-cn Chinese simplifieden-gb English of Great Britianzh-tw Chinese of Taiwan

**Figure 9.**
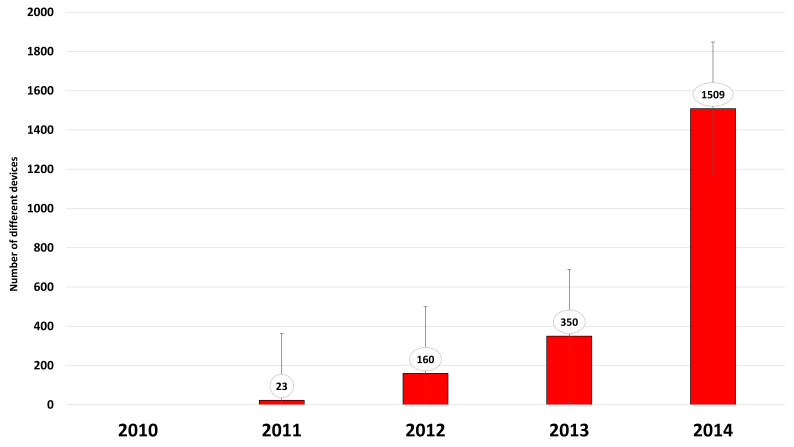
Tropicos showing the exponential growth of mobile device types over a five year period (Suppl. material 5).

**Language** – Tropicos demonstrated relatively stable language usage across the user base. With the dominate languages noted being English, Spanish, Brazilian Portuguese, French, German, and Chinese (Fig. 8). ON and Orowiki, both German websites, revealed German as their primary language, as expected.

**Figure 10.**
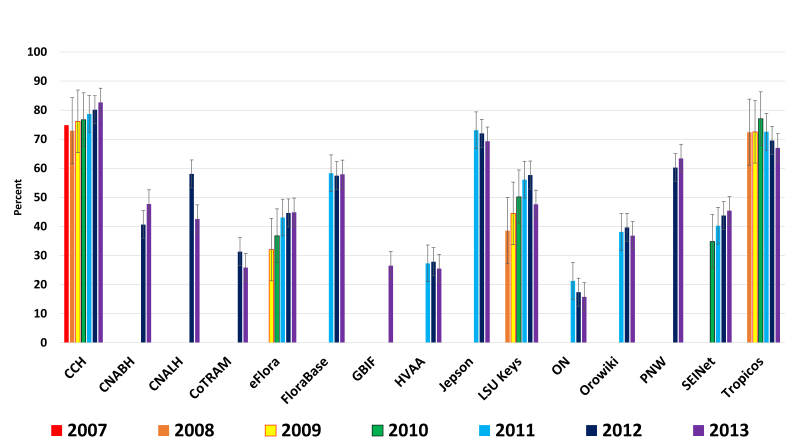
Consistent pattern of usage over seven years of returning users for each resource (Suppl. material 21).

**Figure 11.**
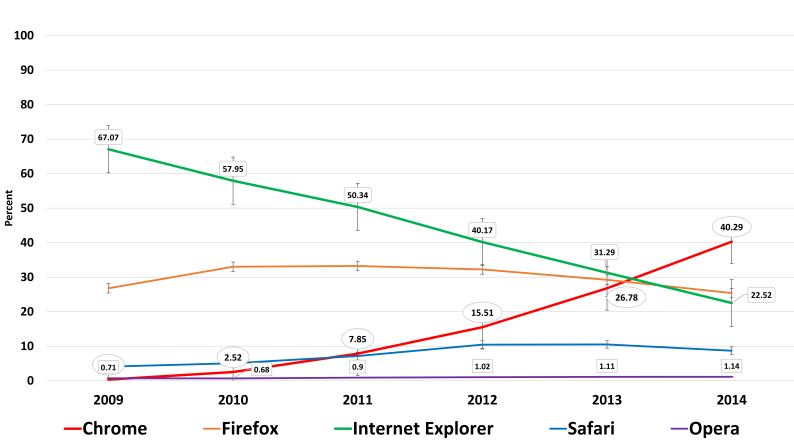
Browsers and their design are vital to how we interact with the WWW. Browser usage at Tropicos from 2009 reveals a changing landscape in the user base of of browsers. This same trend is seen at CCH, eFlora, LSU Keys, and SEINet. Nostalgically and historically, the Netscape browser is also noted in these data at a high of two percent (Suppl. material 20).

**Returning Visitors Vs. New Visitors** – Consistent usage demonstrated a stable regime of returning plant biodiversity data consumers (Fig. 10).

## Discussion

Reinvention and re-purposing of traditional materials have enabled disciplines surrounding plant biodiversity to grow online, as these types of data are ideally suited for the web (Godfray 2002). Herbaria provide a vast array of informational services beyond basic plant preservation to include: nomenclatural resources, literature, identification, requisite glossaries for botanical jargon, and important specimen-derived information. These resources further enable evolutionary and ecological studies that provide an additional advantage of a well-established model found in the kingdom of plants. Differing yet congruent information types make up the whole of web-based botany today, used globally every day (Table 4).

Table 5 is presented in discussion due to its fuzzy nature, as it is a how-many-wheelbarrows-are-pushed approach, which requires extrapolation and the use of one average value from GA. This is achieved by multiplying the number of sessions by the average duration time, yielding a metric for the years of time spent on these sites. This totals over 271 years of user-time over a seven year period (Table 5).

**How a session is determined** – A session is started after a browser requests a tracked webpage. On each, time spent and page views are recorded via a cookie (on desktops, or 90% of this data). By default, each session will expire after thirty minutes. If the user does not progress to another page, it is recorded as a bounce. For example, a researcher clicks on webpage, and then decides to eat lunch for thirty minutes, without clicking on anything after visiting the site. This would count as a 30 minute session, right? *No*, because they bounced.

**Bounce rate** – Bounces are not recorded as sessions since the user did not progress through the site after visiting the first page. For example, the same researcher uses the identical website again after lunch for 30 seconds, does a search for *Carex aurea*, which returns a results page. This results page further links to data-based specimen images which the researcher importantly clicks on. Three clicks and pages into the site now with a good broadband connection. Immediately upon instantiation of the third page, the researcher gets a phone call that lasts for 30 minutes. Here, due to the progression over *three different* web pages (two pages would count too), the session counts. And a bonus dwell time of 30 minutes is recorded in the report. While the actual session lasted only ~30 seconds. Nevertheless, total duration of a session remains informative because it allows for comparison, albeit a somewhat blurry picture of what is actually happening due to the lunch problem. So, progression is the key to a session, as those that do not progress do not count. This possibly skews overall results downwards, especially for those serving one-page websites such as blogs or apps.

**Did that latest upgrade really do anything?** – Additionally, when a user clicks on a directed event (campaign), new informational chains are instantiated. Campaigns are modifications to the JS that reveal supplementary information such as URL parameters that can identify a "web development push". FloraBase is unique in this sample, in that they are modifying their GA JS code to reveal additional parameters with their use. However, it can result in occasional double counting of sessions. This minor discrepancy is trivial when compared to the valuable information that can be gleaned from the data about the change in user behavior after an upgrade.

**Bring your own device (B.Y.O.D.) or here comes mobile** – 2013 was the first year that over one billion smartphones were shipped worldwide, and during this same time period only 300 million PC's were purchased (https://www.gartner.com/doc/2665319). Not so surprisingly, mobile growth has nearly doubled for the examined projects over the years examined (Center, Pew Research 2014). However, desktops continue to dominate traffic overall and comprise over 90% of all traffic. They are running primarily Microsoft Windows for desktops, while the phone & tablet devices favor iOS products. As stated previously, most of these sites are designed for desktop usage first, and mobile second. The trend now is to design for mobile first, while still delivering to desktops and laptops, by using a responsive framework. Vertnet (Constable et al. 2010) is now delivering content that scales itself to any device size using a framework called Bootstrap (Otto, M. and Thorton, J. 2011), thereby serving all device sizes simultaneously, without appification or a log-in.

**Plants aren't social?** – Overall, the amount of social media interaction was found to be trivial (Nicholas and Clark 2014), though it is doubling year to year, but with minor values, e.g. 1–2%. Article levels metrics (Neylon and Wu 2009) are unfortunately not available through GA as it is a standalone that does not incorporate other traffic instances. These low values seen in the population may be the result of multiple factors. One being that curators of museums, experts in their fields, tend to be older individuals, as expert-level knowledge requires time to acquire. Based upon one study, curators have an average age of approximately 50 years, while the 75th percentile is at 58 years (American Alliance of Museums and Philip M. Katz 2012) and older individuals do not engage in social media as much as the younger generations (Duggan and Brenner 2013). Plus, this is another hat to wear by those already wearing many hats. One exception was LSU Keys which did an ad hoc experiment on social media over the past year that pushed the social value to double digits. This on-the-fly effort was an attempt to increase the amount of social traffic by posting to Facebook, Twitter, LinkedIn, and Reddit. These posts were less than ten per site over the year and generated a measurable change when viewed across the population (Fig. 2). Social media requires that developers, curators, and parent institutions work to provide a web presence via fresh content to social media sites, e.g., press releases, publications, images. Thereby generating discernible interest and traffic. Another factor is that developers have yet to find novel ways to engage their audience besides just the standard Facebook, Twitter, and Google+ buttons on a landing page. Lastly, institutions might do more to leverage social media, through collaborative efforts of curators and developers with e-marketing professionals versed in the nuances of social media.

**What not to do** – While canvassing institutions for access to their GA accounts, a few unexpected issues arose concerning the administration of GA accounts:

- Not knowing who owns the GA administrator account. An understandable confusion caused by relocation or promotion of the individual that had originally set up GA for that institution years ago.
- Copying one GA code across different institutions and/or continents resulting in a global miasma of information that requires cleaning and pruning for even simple interpretation.
- Using one GA code from front-door to back-door institutionally; meaning it tracked book-your-wedding user data as well as specimen user data; as well as from the entomology department, the anthropology....
- Deploying GA code to a landing page only. To be effective, all pages require the placement of the tracking code.
- Ignoring the trends towards future mobile usage.

Many institutions still rely only on server-based tracking. This balloons the data through the inclusion of bots or spiders that constantly scour the web to index pages for search or other not-so-noble reasons. It was recently estimated that over half of all web traffic now is non-human or machine based (http://www.incapsula.com/blog/bot-traffic-report-2013.html) basically rendering those that use this server-log method to be data blind (Clark et al. 2014).

**Next-generation of GA?** – Upgrading any GA user to Universal GA, requires the replacement of GA codes on all pages being tracked. A relatively new method, that still requires a one-time total code replacement, is the use of Google Tag Manager (GTM) (http://www.google.com/tagmanager/), as the International Plant Names Index (http://www.ipni.org/) is currently doing. GTM uniquely generates a script that permits future changes by functioning as an "analytic tattoo" for a website; thereby allowing for easy updating across all the deployed pages without wholesale replacement of all scripts. The tattooed script remains the same, but the instructions to that script are mutable, allowing for coding on-the-fly, and allowing for rapid experimentation across site(s). Surely, traffic for all biodiversity based web sites would dwarf these figures for plant biodiversity sites alone. Then considering that less than five percent of all collections-based biodiversity information is now online (Ariño 2010), and the coming voluminous biodiversity yet to be discovered and cataloged (Mora et al. 2011​), these numbers will only grow. It will be interesting to observe what happens to our individual and institutional informational models, and the hard technological carrying capacities, as these data come online. Finally, with modifications to the JS code like those accomplished at FloraBase or IPNI, different parameters will be revealed about usability. It will be exciting to see where vision, creativity and innovation drive these capabilities in the future.

## Supplementary Material

Supplementary material 1 Index Herbariorum – Georeferenced herbaria of the world list Data type: csv
Brief description: Georeferenced list of world's herbaria
File: oo_8436.csv Barbara Thiers, Mary Barkworth

Supplementary material 2 Utah State University Herbarium Records Data type: Many categories of data concerning the development and use of the Intermountain Herbarium
Brief description: The data for all years prior to 1981 were taken from the herbarium's annual report to the Utah Agricultural Experiment Statement. Initially, only specimen growth was included in these reports. With time, we started tracking additional aspects. We have never included our GA data in the report. This is something we should have added when we first installed the software on our pages but we did not. We no longer have easy access to the web site and the GA data.
File: oo_6980.xlsx Mary Barkworth and Michael Piep

Supplementary material 3 New York Botanical Garden Steere Herbarium Records Data type: doc
File: oo_7800.docx Barbara Thiers

Supplementary material 4 Original start date total Data type: xls
Brief description: Total of page, user, and duration
File: oo_33442.xlsx Google

Supplementary material 5 Device types short-term at Tropicos Data type: PDF
Brief description: Different devices used on Tropicos over the past year by model and manufacturer.
File: oo_9538.pdf Tropicos, Google

Supplementary material 6 List of contributing herbaria Data type: xlsx
Brief description: List of herbaria and specimen numbers in respective institutions
File: oo_8493.xlsx Jones, Baxter, Gilbert, Legler, Thiele

Supplementary material 7 Bounce rate supplemental Data type: xls
Brief description: Bounce rates by years
File: oo_11056.xlsx Google,Baxter, Jones

Supplementary material 8 Long term – mobile and tablet combined percentage of all traffic Data type: xlsx
Brief description: Years are determined by using January 01 (or start date of that year) to January 01
File: oo_8506.xlsx Baxter, Jones

Supplementary material 9 Short-term – traffic by device type Data type: xlsx
Brief description: From January 01, 2013 to January 01, 2014
File: oo_8507.xlsx Baxter, Jones

Supplementary material 10 Long and short term operating systems Data type: xls
Brief description: Long and short term operating systems across top-five operating systems.
File: oo_9535.xlsx Google, Jones

Supplementary material 11 Five top language percentages at Tropicos over six years Data type: PDF
Brief description: Top fiver languages over time at Tropicos
File: oo_8534.pdf Tropicos, Google

Supplementary material 12 Tropicos by year for language Data type: PDF
Brief description: 2007-2008
File: oo_8535.pdf Tropicos, Google

Supplementary material 13 Tropicos by year for language 2 Data type: PDF
Brief description: 2008-2009
File: oo_8536.pdf Tropicos, Google

Supplementary material 14 Search, direct, referrals, and social Data type: xls
Brief description: Search, diirect, referrals, not set, and social
File: oo_9537.xlsx Baxter, Jones

Supplementary material 15 Tropicos by year for language 3 Data type: PDF
Brief description: 2009-2010
File: oo_8537.pdf Tropicos, Google

Supplementary material 16 Tropicos by year for language 4 Data type: PDF
Brief description: 2010-2011
File: oo_8538.pdf Tropicos, Google

Supplementary material 17 Tropicos by year for languages Data type: PDF
Brief description: 2011-2012
File: oo_8539.pdf Tropicos, Google

Supplementary material 18 Tropicos by year for language Data type: PDF
Brief description: 2012-2013
File: oo_8540.pdf Tropicos, Google

Supplementary material 19 Tropicos by year for language Data type: PDF
Brief description: 2013-2014
File: oo_8541.pdf Tropicos, Google

Supplementary material 20 Browser wars over five years Data type: xls
Brief description: Browser percentage by years at Jan. 01 to Jan. 01.
File: oo_9536.xlsx Google, Baxter, Jones

Supplementary material 21 Percent returning sessions Data type: xls
Brief description: Percent returning sessions.
File: oo_9558.xlsx Google, Baxter, Jones

## Figures and Tables

**Table 1.** Participants and their start dates.

Project	GA Start date	Participants	Website	Tracked analytic
Consortium of California Herbaria (CCH)	2-May-07	30	ucjeps.berkeley.edu	UA-1304595-1
Consortium of North American Bryophyte Herbaria (CNABH)	1-Jul-12	62	bryophyteportal.org	UA-50594803-2
Consortium of North American Lichen Herbaria (CNALH)	17-Jul-12	59	lichenportal.org	UA-50594803-1
Consortium of Pacific Northwest Herbaria (PNW)	20-Aug-11	24	pnwherbaria.org	UA-29550699-1
Cooperative Taxonomic Resource for American Myrtaceae (CoTRAM)	8-May-11	5	cotram.org	UA-19854426-5
eFlora	24-Oct-09	1	efloras.org	UA-3783322-15
FloraBase	24-Aug-11	1	florabase.dpaw.wa.gov.au	UA-25269128-1
Global Biodiversity Information Facility (GBIF)	28-Jun-13	172*	gbif.org	UA-42057855-1
Herbario Virtual Austral Americano (HVAA)	8-May-11	5	herbariovaa.org	UA-19854426-4
Jepson eFlora (Jepson)	18-Nov-11	1	ucjeps.berkeley.edu	UA-43909100-1
Louisiana State University Herbarium Keys (LSU Keys)	24-Aug-08	1	herbarium.lsu.edu/keys	UA-1414632-44
Offene Naturführer (ON)	6-Nov-11	1*	offene-naturfuehrer.de	UA-27110487-1
Orowiki	6-Nov-11	1	orowiki.org	UA-27158322-1
Southwest Environmental Information Network (SEINet)	19-Nov-10	87	swbiodiversity.org	UA-19854426-1
Tropicos	25-Mar-08	1	tropicos.org	UA-3783322-3

**Table 2.** One year of use, across all sites from June 01, 2013 to June 01, 2014, showing over 4.5 million sessions.

Project	Sessions	Average Page Views	Average User Duration (min)
CCH	73508	7.7	10:41
CNABH	11164	3.98	5:35
CNALH	59138	2.74	3:54
CoTRAM	3630	2.33	1:59
eFlora	1131425	4.68	4:30
FloraBase	388838	9.55	8:44
GBIF	709036	3.99	3:07
HVAA	5403	2.29	1:35
Jepson	121891	5.79	8:35
LSU Keys	7329	3.83	4:38
ON	164788	1.88	1:41
Orowiki	6259	4.91	4:03
PNW	24247	5.96	7:46
SEINet	235603	4.87	5:46
Tropicos	1638764	11.32	12:07

**Table 3.** Long-term outreach in countries, cities, and networks across variable project start dates through June 01, 2014.

Project	Countries	Cities	Networks
CCH	148	5228	8935
CNABH	124	3090	3228
CNALH	175	6891	8015
CoTRAM	134	1614	1969
eFlora	238	28738	109754
FloraBase	222	12558	23415
GBIF	234	17725	36097
HVAA	137	2309	2951
Jepson	188	7361	10376
LSU Keys	135	3514	3577
ON	144	5179	10330
Orowiki	143	2689	3651
PNW	110	2282	2090
SEINet	223	16950	32305
Tropicos	238	23923	68509

**Table 4.** One-year outreach in countries, cities, and networks from June 01, 2013 to June 01, 2014.

Project	Countries	Cities	Networks
CCH	95	1794	1982
CNABH	110	1990	1981
CNALH	164	5303	5864
CoTRAM	100	861	999
eFlora	230	20569	41826
FloraBase	211	8030	11659
GBIF	234	17725	36097
HVAA	114	1128	1366
Jepson	175	5533	6933
LSU Keys	108	1660	1477
ON	117	4104	6641
Orowiki	118	1620	1878
PNW	105	1915	1693
SEINet	209	11204	15756
Tropicos	229	16172	30531

**Table 5.** **271 years total-session-time in seven years.** Total user duration time yields 271 years since inception. Derived by sessions multiplied by the avg time to yield years of usage. *Caveats: those denoted by asterisks are sub-sampled by GA, so it is a population that is sub-sampled due to scale.

Project	Sessions	Average User Duration (seconds)	Total Duration (years)
CCH	433964	650	8.9
CNABH	21880	237	0.17
CNALH	104933	233	0.78
CoTRAM	10457	136	0.05
eFlora*	5337830	233	39.43
FloraBase*	1233942	423	16.6
GBIF	803552	248	6.3
HVAA	17819	105	0.07
Jepson	276009	561	4.9
LSU Keys	25732	270	0.2
ON	410910	103	1.2
Orowiki	16534	308	0.2
PNW	38216	484	0.6
SEINet	740129	295	6.9
Tropicos*	7486692	778	184.7
**Total time**			**271 Years**
