# Supplementary material for: Trends in access of plant biodiversity data revealed by Google Analytics
Source: Biodivers Data J. 2014 Nov 11;(2):e1558. doi: 10.3897/BDJ.2.e1558 (PMC4238075; doi:10.3897/BDJ.2.e1558)
Supplement: Supplementary material 3 — New York Botanical Garden Steere Herbarium Records [file biodiversity_data_journal-2-e1558-s003.docx]

## New York Botanical Garden Steere Herbarium

## FIVE YEAR CUMULATIVE STATISTICS 2005-2009 (NSF FORMAT)

| **Category** | **2008** | **2009** | **2010** | **2011** | **2012** | **Total** | **Yearly Average** |
| --- | --- | --- | --- | --- | --- | --- | --- |
| **Specimens added to herbarium** | 52,402 | 51,297 | 51,569 | 59,528 | 37,345 | 252,141 | **50,428** |
| **Loans sent to professionals** | 197 | 218 | 264 | 257 | 233 | 1,169 | **234** |
| **Specimens loaned to professionals** | 13,153 | 14,178 | 14568 | 17832 | 12,083 | 71,814 | **14,363** |
| **Loans sent to students** | 75 | 73 | 63 | 63 | 88 | 362 | **72** |
| **Specimens loaned to students** | 8153 | 9884 | 4352 | 4458 | 4719 | 31,566 | **6,313** |
| **Number of countries receiving loans** | 28 | 38 | 35 | 29 | 33 |  |  |
| **Number of states receiving loans** | 29 | 38 | 33 | 35 | 27 |  |  |
| **Loans requested for NYBG scientists** | 98 | 130 | 88 | 73 | 92 | 481 | **96** |
| **Specimens received for NYBG scientists** | 8290 | 7469 | 4435 | 4289 | 6565 | 31,048 | **6,210** |
| **Loans received for NYBG students** | 29 | 47 | 36 | 27 | 45 | 184 | **37** |
| **Specimens received for NYBG students** | 2685 | 2700 | 2016 | 1430 | 3845 | 12,676 | **2,535** |
| **Specimens sent to specialists as gifts for identification** | 2116 | 504 | 721 | 2699 | 2916 | 8,956 | **1,791** |
| **Number of visitors using herbarium** | 188 | 155 | 178 | 202 | 247 | 970 | **194** |
| **Total length of visits (days)** | 2644 | 2519 | 2437 | 2405 | 3918 | 13,923 | **2,785** |
| **Number of states** | 25 | 25 | 26 | 30 | 30 |  |  |
| **Number of countries** | 28 | 23 | 27 | 31 | 30 |  |  |
| **Information requests (professional)** | 38 | 45 | 48 | 55 | 67 | 186 | **37** |
| **Information requests (students)** | 12 | 12 | 2 | 4 | 5 | 30 | **6** |
| **Average hits per month for Virtual Herbarium** | 234,672 | 254,890 | 331,641 | 292,565 | 366,978 | 1,480,746 | **296,149** |
| **Publications by professionals that cite NYBG specimens** | 209 | 232 | 224 | 255 | 202 | 1,122 | **224** |
| **Publications by students that cite NYBG specimens** | 41 | 30 | 42 | 55 | 67 | 235 | **47** |
