# Supplementary material for: Trends in access of plant biodiversity data revealed by Google Analytics
Source: Biodivers Data J. 2014 Nov 11;(2):e1558. doi: 10.3897/BDJ.2.e1558 (PMC4238075; doi:10.3897/BDJ.2.e1558)
Supplement: Supplementary material 5 — Device types short-term at Tropicos [file biodiversity_data_journal-2-e1558-s005.pdf]

Google Analytics

http://www.tropicos.org - http://www.tro… www.tropicos.org [Go to this report]

Devices

Jun 1, 2013 - Jun 1, 2014

All Sessions
3.68%

+ Add Segment

Explorer

Summary

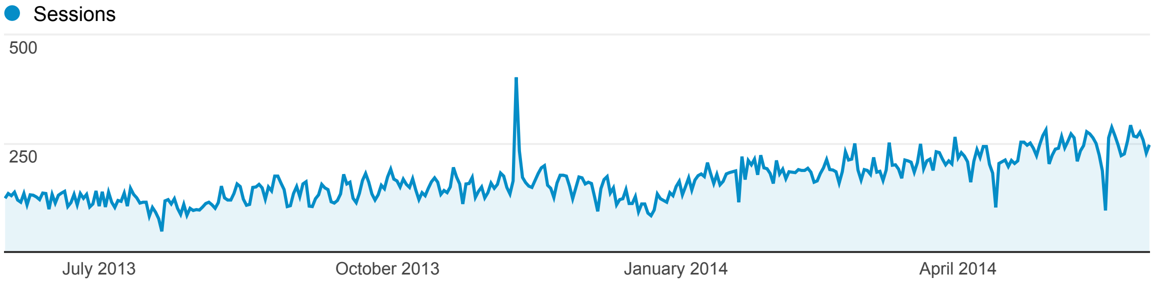

	Mobile Device Info	Acquisition: Sessions	Acquisition: % New Sessions	Acquisition: New Users	Behavior: Bounce Rate	Behavior: Pages / Session	Behavior: Avg. Session Duration	Conversions: Goal Conversion Rate	Conversions: Goal Completions	Conversions: Goal Value
		60,369 % of Total: 3.68% (1,638,764)	69.05% Site Avg: 33.60% (105.48%)	41,686 % of Total: 7.57% (550,703)	62.48% Site Avg: 33.01% (89.29%)	3.73 Site Avg: 11.32 (-67.06%)	00:03:02 Site Avg: 00:12:07 (-75.01%)	0.00% Site Avg: 0.00% (0.00%)	0 % of Total: 0.00% (0)	$0.00 % of Total: 0.00% ($0.00)
1.	Apple iPad	**17,305** **(28.67%)**	67.37%	11,659 (27.97%)	61.47%	4.22	00:02:51	0.00%	0 (0.00%)	$0.00 (0.00%)
2.	Apple iPhone	**9,707** **(16.08%)**	72.37%	7,025 (16.85%)	65.50%	2.81	00:02:10	0.00%	0 (0.00%)	$0.00 (0.00%)
3.	(not set)	**6,953** **(11.52%)**	77.15%	5,364 (12.87%)	65.91%	2.87	00:02:39	0.00%	0 (0.00%)	$0.00 (0.00%)
4.	Samsung GT-I9300 Galaxy S III	**826** **(1.37%)**	63.68%	526 (1.26%)	65.74%	3.22	00:02:21	0.00%	0 (0.00%)	$0.00 (0.00%)
5.	Apple iPod	**670** **(1.11%)**	62.54%	419 (1.01%)	57.31%	3.02	00:02:17	0.00%	0 (0.00%)	$0.00 (0.00%)
6.	Opera Opera Mini for S60	**437** **(0.72%)**	77.57%	339 (0.81%)	68.88%	2.33	00:01:51	0.00%	0 (0.00%)	$0.00 (0.00%)
7.	Samsung GT-I9100 Galaxy S II	**424** **(0.70%)**	74.53%	316 (0.76%)	67.45%	3.06	00:01:45	0.00%	0 (0.00%)	$0.00 (0.00%)
8.	Google Nexus 7	**414** **(0.69%)**	72.22%	299 (0.72%)	68.12%	2.93	00:01:43	0.00%	0 (0.00%)	$0.00 (0.00%)
9.	Samsung GT-I9500 Galaxy S IV	**386** **(0.64%)**	81.87%	316 (0.76%)	68.13%	2.43	00:01:39	0.00%	0 (0.00%)	$0.00 (0.00%)
10.	Samsung GT-I8190L Galaxy S III Mini	**374** **(0.62%)**	63.90%	239 (0.57%)	50.27%	4.47	00:04:13	0.00%	0 (0.00%)	$0.00 (0.00%)
11.	Samsung GT-N7100 Galaxy Note II	**366** **(0.61%)**	69.67%	255 (0.61%)	66.12%	3.04	00:02:10	0.00%	0 (0.00%)	$0.00 (0.00%)
12.	Samsung SHV-E160S Galaxy Note	**364** **(0.60%)**	2.20%	8 (0.02%)	31.32%	4.32	00:04:24	0.00%	0 (0.00%)	$0.00 (0.00%)
13.	Samsung GT-N5100 Galaxy Note 8.0	**353** **(0.58%)**	17.28%	61 (0.15%)	31.44%	32.93	00:25:50	0.00%	0 (0.00%)	$0.00 (0.00%)
14.	Samsung GT-P5110 Galaxy Tab 2 10.1	**340** **(0.56%)**	59.12%	201 (0.48%)	62.35%	3.57	00:03:09	0.00%	0 (0.00%)	$0.00 (0.00%)

15.	Samsung GT-P5113 Galaxy Tab 2 10.1	**319** **(0.53%)**	14.42%	46 (0.11%)	28.84%	14.22	00:19:37	0.00%	0 (0.00%)	$0.00 (0.00%)
16.	Samsung GT-P3110 Galaxy Tab 2 7.0	**315** **(0.52%)**	46.67%	147 (0.35%)	43.81%	4.90	00:05:01	0.00%	0 (0.00%)	$0.00 (0.00%)
17.	Nokia Lumia 520	**299** **(0.50%)**	71.57%	214 (0.51%)	52.84%	4.15	00:02:40	0.00%	0 (0.00%)	$0.00 (0.00%)
18.	Samsung GT-I9300 Galaxy SIII	**276** **(0.46%)**	76.81%	212 (0.51%)	69.93%	3.00	00:02:24	0.00%	0 (0.00%)	$0.00 (0.00%)
19.	Samsung GT-N8000 Galaxy Note 10.1	**269** **(0.45%)**	53.90%	145 (0.35%)	52.42%	4.78	00:03:31	0.00%	0 (0.00%)	$0.00 (0.00%)
20.	Samsung GT-P3100 Galaxy Tab 2 7.0	**253** **(0.42%)**	66.01%	167 (0.40%)	50.59%	4.77	00:05:58	0.00%	0 (0.00%)	$0.00 (0.00%)
21.	Samsung GT-I9505 Galaxy S IV	**251** **(0.42%)**	88.45%	222 (0.53%)	77.29%	1.83	00:00:41	0.00%	0 (0.00%)	$0.00 (0.00%)
22.	Samsung GT-P5100 Galaxy Tab 2 10.1	**223** **(0.37%)**	67.71%	151 (0.36%)	56.95%	3.88	00:03:49	0.00%	0 (0.00%)	$0.00 (0.00%)
23.	Samsung GT-C3322i Metro Duos	**215** **(0.36%)**	84.65%	182 (0.44%)	71.63%	1.96	00:01:34	0.00%	0 (0.00%)	$0.00 (0.00%)
24.	LG P768 Optimus L9	**210** **(0.35%)**	19.05%	40 (0.10%)	61.43%	2.40	00:02:25	0.00%	0 (0.00%)	$0.00 (0.00%)
25.	Samsung GT-P3113 Galaxy Tab 2 7.0	**185** **(0.31%)**	33.51%	62 (0.15%)	67.03%	2.25	00:03:17	0.00%	0 (0.00%)	$0.00 (0.00%)
26.	Samsung GT-I8190 Galaxy S III Mini	**184** **(0.30%)**	72.83%	134 (0.32%)	62.50%	3.03	00:02:29	0.00%	0 (0.00%)	$0.00 (0.00%)
27.	Samsung GT-S5830M Galaxy Ace	**178** **(0.29%)**	61.24%	109 (0.26%)	45.51%	7.27	00:07:33	0.00%	0 (0.00%)	$0.00 (0.00%)
28.	Samsung GT-N8010 Galaxy Note 10.1	**177** **(0.29%)**	67.23%	119 (0.29%)	72.88%	3.05	00:02:14	0.00%	0 (0.00%)	$0.00 (0.00%)
29.	Alcatel OT-960C One Touch 960C	**170** **(0.28%)**	3.53%	6 (0.01%)	85.88%	1.22	00:00:13	0.00%	0 (0.00%)	$0.00 (0.00%)
30.	Google Nexus 4	**164** **(0.27%)**	57.32%	94 (0.23%)	60.37%	3.24	00:02:20	0.00%	0 (0.00%)	$0.00 (0.00%)
31.	BlackBerry 9320 BlackBerry Curve 9320	**161** **(0.27%)**	80.12%	129 (0.31%)	58.39%	3.61	00:05:40	0.00%	0 (0.00%)	$0.00 (0.00%)
32.	LG E612g Optimus L5	**161** **(0.27%)**	12.42%	20 (0.05%)	34.16%	7.50	00:04:43	0.00%	0 (0.00%)	$0.00 (0.00%)
33.	Samsung GT-P5210 Galaxy Tab 3 10.1	**152** **(0.25%)**	68.42%	104 (0.25%)	63.16%	5.66	00:04:58	0.00%	0 (0.00%)	$0.00 (0.00%)
34.	Spice M6800 Flo	**148** **(0.25%)**	85.81%	127 (0.30%)	66.89%	2.20	00:01:56	0.00%	0 (0.00%)	$0.00 (0.00%)
35.	Samsung GT-E2200	**136** **(0.23%)**	78.68%	107 (0.26%)	64.71%	3.01	00:03:04	0.00%	0 (0.00%)	$0.00 (0.00%)
36.	Samsung GT-P7510 Galaxy Tab 10.1	**136** **(0.23%)**	55.88%	76 (0.18%)	54.41%	8.61	00:06:15	0.00%	0 (0.00%)	$0.00 (0.00%)
37.	Amazon KFTT Kindle Fire HD 7	**135** **(0.22%)**	74.81%	101 (0.24%)	65.19%	3.14	00:01:35	0.00%	0 (0.00%)	$0.00 (0.00%)
38.	Samsung GT-S5360L Galaxy Y	**132** **(0.22%)**	67.42%	89 (0.21%)	51.52%	3.77	00:03:32	0.00%	0 (0.00%)	$0.00 (0.00%)
39.	SonyEricsson LT26i Xperia Arc HD	**131** **(0.22%)**	46.56%	61 (0.15%)	48.85%	4.76	00:06:29	0.00%	0 (0.00%)	$0.00 (0.00%)
40.	Nokia 311 Asha 311	**129** **(0.21%)**	83.72%	108 (0.26%)	57.36%	3.74	00:03:26	0.00%	0 (0.00%)	$0.00 (0.00%)

41.	Samsung GT-I9082 Galaxy Grand Duos	**128** (0.21%)	67.97%	87 (0.21%)	59.38%	2.55	00:03:30	0.00%	0 (0.00%)	$0.00 (0.00%)
42.	Nokia Lumia 710	**124** (0.21%)	69.35%	86 (0.21%)	55.65%	4.45	00:05:53	0.00%	0 (0.00%)	$0.00 (0.00%)
43.	Nokia 5233	**123** (0.20%)	95.12%	117 (0.28%)	78.05%	1.42	00:00:35	0.00%	0 (0.00%)	$0.00 (0.00%)
44.	Motorola XT914 Razr D1	**119** (0.20%)	36.13%	43 (0.10%)	49.58%	3.55	00:04:01	0.00%	0 (0.00%)	$0.00 (0.00%)
45.	Lenovo 1838 ThinkPad Tablet	**117** (0.19%)	2.56%	3 (0.01%)	18.80%	11.84	00:21:28	0.00%	0 (0.00%)	$0.00 (0.00%)
46.	Samsung GT-S5360 Galaxy Y	**109** (0.18%)	89.91%	98 (0.24%)	75.23%	2.06	00:01:23	0.00%	0 (0.00%)	$0.00 (0.00%)
47.	Nokia Lumia 610	**108** (0.18%)	45.37%	49 (0.12%)	53.70%	3.17	00:03:25	0.00%	0 (0.00%)	$0.00 (0.00%)
48.	Samsung GT-S5830i Galaxy Ace	**108** (0.18%)	78.70%	85 (0.20%)	66.67%	2.62	00:02:10	0.00%	0 (0.00%)	$0.00 (0.00%)
49.	Samsung GT-I9070 Galaxy S Advance	**106** (0.18%)	72.64%	77 (0.18%)	58.49%	3.42	00:02:49	0.00%	0 (0.00%)	$0.00 (0.00%)
50.	Huawei U9200 Ascend P1	**103** (0.17%)	12.62%	13 (0.03%)	24.27%	7.67	00:04:41	0.00%	0 (0.00%)	$0.00 (0.00%)
51.	Motorola XT1033 Moto G	**103** (0.17%)	39.81%	41 (0.10%)	52.43%	3.68	00:03:41	0.00%	0 (0.00%)	$0.00 (0.00%)
52.	Samsung GT-N7000 Galaxy Note	**101** (0.17%)	85.15%	86 (0.21%)	75.25%	2.19	00:01:11	0.00%	0 (0.00%)	$0.00 (0.00%)
53.	BlackBerry 9900 Dakota	**98** (0.16%)	72.45%	71 (0.17%)	60.20%	3.82	00:02:47	0.00%	0 (0.00%)	$0.00 (0.00%)
54.	Samsung GT-S5830 Galaxy Ace	**97** (0.16%)	73.20%	71 (0.17%)	51.55%	3.87	00:04:37	0.00%	0 (0.00%)	$0.00 (0.00%)
55.	Samsung SGH-T999 Galaxy S III	**97** (0.16%)	40.21%	39 (0.09%)	81.44%	2.18	00:01:02	0.00%	0 (0.00%)	$0.00 (0.00%)
56.	Nokia 200 Asha 200	**96** (0.16%)	96.88%	93 (0.22%)	67.71%	1.76	00:01:12	0.00%	0 (0.00%)	$0.00 (0.00%)
57.	Samsung GT-N5110 Galaxy Note 8.0 WiFi	**96** (0.16%)	32.29%	31 (0.07%)	32.29%	6.28	00:06:02	0.00%	0 (0.00%)	$0.00 (0.00%)
58.	Nokia Asha 302 Asha 302	**94** (0.16%)	91.49%	86 (0.21%)	73.40%	1.81	00:01:26	0.00%	0 (0.00%)	$0.00 (0.00%)
59.	Nokia X2-02	**92** (0.15%)	83.70%	77 (0.18%)	60.87%	2.83	00:04:34	0.00%	0 (0.00%)	$0.00 (0.00%)
60.	Samsung SM-T210 Galaxy Tab 3 7.0	**91** (0.15%)	70.33%	64 (0.15%)	59.34%	3.33	00:03:50	0.00%	0 (0.00%)	$0.00 (0.00%)
61.	Sony Tablet S	**91** (0.15%)	25.27%	23 (0.06%)	43.96%	7.79	00:08:17	0.00%	0 (0.00%)	$0.00 (0.00%)
62.	BlackBerry 9300 Curve 3G	**90** (0.15%)	81.11%	73 (0.18%)	63.33%	3.20	00:02:43	0.00%	0 (0.00%)	$0.00 (0.00%)
63.	Samsung GT-N5110 Galaxy Note 8.0	**90** (0.15%)	16.67%	15 (0.04%)	27.78%	7.48	00:05:29	0.00%	0 (0.00%)	$0.00 (0.00%)
64.	Nokia Lumia 505	**88** (0.15%)	55.68%	49 (0.12%)	43.18%	3.55	00:04:58	0.00%	0 (0.00%)	$0.00 (0.00%)
65.	Motorola XT1032 Moto G	**87** (0.14%)	71.26%	62 (0.15%)	59.77%	3.18	00:01:56	0.00%	0 (0.00%)	$0.00 (0.00%)
66.	Samsung GT-S7562 Galaxy S Duos	**85** (0.14%)	89.41%	76 (0.18%)	71.76%	2.08	00:01:59	0.00%	0 (0.00%)	$0.00 (0.00%)
67.	Opera Mini 1	**84** (0.14%)	82.14%	69 (0.17%)	65.48%	2.44	00:02:40	0.00%	0 (0.00%)	$0.00 (0.00%)
68.	HTC M7 One	**83** (0.14%)	80.72%	67 (0.16%)	75.90%	1.60	00:00:22	0.00%	0 (0.00%)	$0.00 (0.00%)
69.	Motorola MOTXT912B Droid Razr 4G	**83** (0.14%)	66.27%	55 (0.13%)	67.47%	5.10	00:04:31	0.00%	0 (0.00%)	$0.00 (0.00%)

70.	Nokia 308 Asha 308	**83** **(0.14%)**	93.98%	78 (0.19%)	62.65%	2.10	00:01:36	0.00%	0 (0.00%)	$0.00 (0.00%)
71.	BlackBerry 9800 Torch	**80** **(0.13%)**	82.50%	66 (0.16%)	67.50%	1.79	00:01:40	0.00%	0 (0.00%)	$0.00 (0.00%)
72.	Samsung GT-I8190N Galaxy S III Mini	**80** **(0.13%)**	73.75%	59 (0.14%)	68.75%	2.18	00:01:08	0.00%	0 (0.00%)	$0.00 (0.00%)
73.	Samsung GT-P1000 Galaxy Tab	**79** **(0.13%)**	56.96%	45 (0.11%)	48.10%	13.35	00:08:50	0.00%	0 (0.00%)	$0.00 (0.00%)
74.	Sony C1904 Xperia M	**78** **(0.13%)**	28.21%	22 (0.05%)	23.08%	6.42	00:07:23	0.00%	0 (0.00%)	$0.00 (0.00%)
75.	Samsung Galaxy Nexus	**77** **(0.13%)**	58.44%	45 (0.11%)	74.03%	2.92	00:01:31	0.00%	0 (0.00%)	$0.00 (0.00%)
76.	Samsung SM-T311 Galaxy Tab 3 8.0	**77** **(0.13%)**	31.17%	24 (0.06%)	61.04%	3.91	00:03:34	0.00%	0 (0.00%)	$0.00 (0.00%)
77.	Samsung GT-S5830L Galaxy Ace	**76** **(0.13%)**	71.05%	54 (0.13%)	59.21%	3.54	00:03:18	0.00%	0 (0.00%)	$0.00 (0.00%)
78.	Samsung SM-C101 GALAXY S4 Zoom	**76** **(0.13%)**	10.53%	8 (0.02%)	28.95%	7.38	00:07:41	0.00%	0 (0.00%)	$0.00 (0.00%)
79.	Amazon Kindle Fire Kindle Fire	**75** **(0.12%)**	61.33%	46 (0.11%)	70.67%	3.44	00:03:09	0.00%	0 (0.00%)	$0.00 (0.00%)
80.	Samsung SM-T310 Galaxy Tab 3 8.0	**75** **(0.12%)**	57.33%	43 (0.10%)	58.67%	3.67	00:03:47	0.00%	0 (0.00%)	$0.00 (0.00%)
81.	Google Nexus 10	**74** **(0.12%)**	68.92%	51 (0.12%)	70.27%	4.08	00:02:11	0.00%	0 (0.00%)	$0.00 (0.00%)
82.	Samsung SM-N9005 Galaxy Note 3	**74** **(0.12%)**	89.19%	66 (0.16%)	82.43%	1.80	00:00:31	0.00%	0 (0.00%)	$0.00 (0.00%)
83.	Nokia Lumia 620	**71** **(0.12%)**	70.42%	50 (0.12%)	56.34%	3.62	00:02:59	0.00%	0 (0.00%)	$0.00 (0.00%)
84.	Samsung GT-I9195 Galaxy S4 Mini	**71** **(0.12%)**	81.69%	58 (0.14%)	77.46%	2.32	00:01:49	0.00%	0 (0.00%)	$0.00 (0.00%)
85.	Lenovo A2107A-H IdeaTab A2107A-H	**70** **(0.12%)**	27.14%	19 (0.05%)	54.29%	4.13	00:07:55	0.00%	0 (0.00%)	$0.00 (0.00%)
86.	Nokia Nokia Asha 201 Asha 201	**69** **(0.11%)**	91.30%	63 (0.15%)	73.91%	2.07	00:02:24	0.00%	0 (0.00%)	$0.00 (0.00%)
87.	SonyEricsson C6603 Xperia Z	**69** **(0.11%)**	84.06%	58 (0.14%)	73.91%	2.43	00:01:06	0.00%	0 (0.00%)	$0.00 (0.00%)
88.	Nokia C3-00	**68** **(0.11%)**	94.12%	64 (0.15%)	60.29%	2.31	00:03:55	0.00%	0 (0.00%)	$0.00 (0.00%)
89.	Nokia Lumia 920	**66** **(0.11%)**	72.73%	48 (0.12%)	56.06%	3.53	00:02:32	0.00%	0 (0.00%)	$0.00 (0.00%)
90.	Toshiba AT200 Excite AT200	**66** **(0.11%)**	4.55%	3 (0.01%)	69.70%	2.32	00:03:28	0.00%	0 (0.00%)	$0.00 (0.00%)
91.	Samsung SPH-L710 Galaxy S III	**65** **(0.11%)**	80.00%	52 (0.12%)	75.38%	2.89	00:01:33	0.00%	0 (0.00%)	$0.00 (0.00%)
92.	BlackBerry 8520 Curve	**64** **(0.11%)**	85.94%	55 (0.13%)	64.06%	1.91	00:02:20	0.00%	0 (0.00%)	$0.00 (0.00%)
93.	BlackBerry 9360 Curve	**64** **(0.11%)**	78.12%	50 (0.12%)	65.62%	2.14	00:02:04	0.00%	0 (0.00%)	$0.00 (0.00%)
94.	Motorola XT1058 Moto X	**64** **(0.11%)**	35.94%	23 (0.06%)	57.81%	3.02	00:02:04	0.00%	0 (0.00%)	$0.00 (0.00%)
95.	Nokia Lumia 800	**64** **(0.11%)**	82.81%	53 (0.13%)	76.56%	3.72	00:02:20	0.00%	0 (0.00%)	$0.00 (0.00%)
96.	Nokia 305 Asha 305	**63** **(0.10%)**	93.65%	59 (0.14%)	69.84%	1.59	00:00:56	0.00%	0 (0.00%)	$0.00 (0.00%)
97.	Samsung SM-T211 Galaxy Tab 3 7.0 3G	**63** **(0.10%)**	53.97%	34 (0.08%)	41.27%	4.60	00:04:11	0.00%	0 (0.00%)	$0.00 (0.00%)

98.	HTC PJ83100 One X	**61** (0.10%)	78.69%	48 (0.12%)	77.05%	1.61	00:00:59	0.00%	0 (0.00%)	$0.00 (0.00%)
99.	LG E400f Optimus L3	**61** (0.10%)	45.90%	28 (0.07%)	39.34%	11.41	00:17:41	0.00%	0 (0.00%)	$0.00 (0.00%)
100.	Motorola XT923 Droid Razr HD	**61** (0.10%)	52.46%	32 (0.08%)	63.93%	4.13	00:02:14	0.00%	0 (0.00%)	$0.00 (0.00%)
101.	Nokia 206 Asha 206	**58** (0.10%)	82.76%	48 (0.12%)	60.34%	2.14	00:01:29	0.00%	0 (0.00%)	$0.00 (0.00%)
102.	LG Nexus 5	**57** (0.09%)	85.96%	49 (0.12%)	85.96%	1.51	00:00:34	0.00%	0 (0.00%)	$0.00 (0.00%)
103.	Samsung GT-I8262 Galaxy Duos	**57** (0.09%)	82.46%	47 (0.11%)	50.88%	3.75	00:04:01	0.00%	0 (0.00%)	$0.00 (0.00%)
104.	Samsung GT-N7105 Galaxy Note II	**57** (0.09%)	91.23%	52 (0.12%)	78.95%	2.86	00:01:33	0.00%	0 (0.00%)	$0.00 (0.00%)
105.	Nokia Lumia 900	**56** (0.09%)	57.14%	32 (0.08%)	58.93%	2.77	00:02:59	0.00%	0 (0.00%)	$0.00 (0.00%)
106.	Samsung GT-P7500 Galaxy Tab 10.1	**56** (0.09%)	78.57%	44 (0.11%)	75.00%	1.86	00:00:57	0.00%	0 (0.00%)	$0.00 (0.00%)
107.	Samsung SGH-T989 Galaxy SII	**56** (0.09%)	69.64%	39 (0.09%)	69.64%	2.95	00:01:28	0.00%	0 (0.00%)	$0.00 (0.00%)
108.	Samsung GT-I8160 Galaxy Ace 2	**55** (0.09%)	78.18%	43 (0.10%)	58.18%	3.49	00:03:21	0.00%	0 (0.00%)	$0.00 (0.00%)
109.	Samsung GT-I9000 Galaxy S	**55** (0.09%)	81.82%	45 (0.11%)	74.55%	1.53	00:01:10	0.00%	0 (0.00%)	$0.00 (0.00%)
110.	Samsung GT-S6802B Galaxy Ace Duos	**55** (0.09%)	14.55%	8 (0.02%)	23.64%	6.73	00:08:49	0.00%	0 (0.00%)	$0.00 (0.00%)
111.	Asus ME173X Memo Pad HD7	**54** (0.09%)	51.85%	28 (0.07%)	75.93%	2.09	00:01:03	0.00%	0 (0.00%)	$0.00 (0.00%)
112.	Motorola XT890 RAZR i	**53** (0.09%)	73.58%	39 (0.09%)	50.94%	2.68	00:02:41	0.00%	0 (0.00%)	$0.00 (0.00%)
113.	Samsung SGH-I337 Galaxy S IV	**53** (0.09%)	88.68%	47 (0.11%)	79.25%	2.92	00:01:23	0.00%	0 (0.00%)	$0.00 (0.00%)
114.	SonyEricsson LT22i Xperia P LT22i	**53** (0.09%)	86.79%	46 (0.11%)	67.92%	2.55	00:01:31	0.00%	0 (0.00%)	$0.00 (0.00%)
115.	Asus TF300T Transformer Pad TF300T	**52** (0.09%)	76.92%	40 (0.10%)	61.54%	3.92	00:03:23	0.00%	0 (0.00%)	$0.00 (0.00%)
116.	LG MS770 Motion 4G	**52** (0.09%)	67.31%	35 (0.08%)	82.69%	1.56	00:01:21	0.00%	0 (0.00%)	$0.00 (0.00%)
117.	Samsung GT-I9192 Galaxy S4 Mini	**52** (0.09%)	73.08%	38 (0.09%)	59.62%	3.35	00:02:32	0.00%	0 (0.00%)	$0.00 (0.00%)
118.	BlackBerry 9700 Bold	**51** (0.08%)	78.43%	40 (0.10%)	70.59%	1.80	00:01:25	0.00%	0 (0.00%)	$0.00 (0.00%)
119.	Samsung GT-S6102B Galaxy Y Duos	**51** (0.08%)	68.63%	35 (0.08%)	45.10%	7.47	00:08:22	0.00%	0 (0.00%)	$0.00 (0.00%)
120.	Nokia Lumia 820	**50** (0.08%)	62.00%	31 (0.07%)	42.00%	10.54	00:12:50	0.00%	0 (0.00%)	$0.00 (0.00%)
121.	Samsung GT-I9305 Galaxy S III LTE International	**50** (0.08%)	86.00%	43 (0.10%)	72.00%	2.46	00:00:57	0.00%	0 (0.00%)	$0.00 (0.00%)
122.	Samsung GT-S6810P Galaxy Fame	**50** (0.08%)	42.00%	21 (0.05%)	42.00%	9.28	00:09:03	0.00%	0 (0.00%)	$0.00 (0.00%)
123.	Samsung SCH-I545 Galaxy S IV	**50** (0.08%)	84.00%	42 (0.10%)	78.00%	2.10	00:01:00	0.00%	0 (0.00%)	$0.00 (0.00%)
124.	Samsung GT-I9190 Galaxy S4	**49** (0.08%)	59.18%	29 (0.07%)	61.22%	2.84	00:04:19	0.00%	0 (0.00%)	$0.00 (0.00%)

	Mini									
125.	Samsung GT-S5360B Galaxy Y	**49** **(0.08%)**	67.35%	33 (0.08%)	48.98%	4.29	00:06:15	0.00%	0 (0.00%)	$0.00 (0.00%)
126.	Samsung GT-S5570 Galaxy Mini	**49** **(0.08%)**	89.80%	44 (0.11%)	71.43%	1.67	00:01:10	0.00%	0 (0.00%)	$0.00 (0.00%)
127.	BlackBerry 9780	**48** **(0.08%)**	75.00%	36 (0.09%)	66.67%	2.65	00:01:37	0.00%	0 (0.00%)	$0.00 (0.00%)
128.	BlackBerry PlayBook	**48** **(0.08%)**	79.17%	38 (0.09%)	54.17%	2.35	00:01:15	0.00%	0 (0.00%)	$0.00 (0.00%)
129.	LG E425f Optimus L3 II	**48** **(0.08%)**	16.67%	8 (0.02%)	35.42%	10.42	00:18:24	0.00%	0 (0.00%)	$0.00 (0.00%)
130.	Samsung GT-S6102 Galaxy Y Duos	**48** **(0.08%)**	79.17%	38 (0.09%)	70.83%	2.12	00:01:58	0.00%	0 (0.00%)	$0.00 (0.00%)
131.	LG E400g Optimus L3	**47** **(0.08%)**	53.19%	25 (0.06%)	48.94%	4.43	00:06:08	0.00%	0 (0.00%)	$0.00 (0.00%)
132.	Nokia 205 Asha 205	**47** **(0.08%)**	93.62%	44 (0.11%)	87.23%	1.23	00:00:33	0.00%	0 (0.00%)	$0.00 (0.00%)
133.	BlackBerry 9810 Torch 4G	**46** **(0.08%)**	86.96%	40 (0.10%)	58.70%	3.41	00:02:36	0.00%	0 (0.00%)	$0.00 (0.00%)
134.	Samsung SM-N900T Galaxy Note 3	**46** **(0.08%)**	82.61%	38 (0.09%)	67.39%	3.33	00:02:10	0.00%	0 (0.00%)	$0.00 (0.00%)
135.	Motorola XT919 RAZR D3	**45** **(0.07%)**	73.33%	33 (0.08%)	55.56%	3.07	00:02:48	0.00%	0 (0.00%)	$0.00 (0.00%)
136.	Samsung SGH-i520V	**44** **(0.07%)**	100.00%	44 (0.11%)	70.45%	1.43	00:00:49	0.00%	0 (0.00%)	$0.00 (0.00%)
137.	Sony ST21a Xperia Tipo	**44** **(0.07%)**	45.45%	20 (0.05%)	63.64%	3.07	00:03:36	0.00%	0 (0.00%)	$0.00 (0.00%)
138.	BlackBerry 9790 BlackBerry Bold 9790	**43** **(0.07%)**	93.02%	40 (0.10%)	72.09%	2.07	00:00:52	0.00%	0 (0.00%)	$0.00 (0.00%)
139.	Sony LT30p Xperia T	**43** **(0.07%)**	60.47%	26 (0.06%)	74.42%	4.07	00:01:42	0.00%	0 (0.00%)	$0.00 (0.00%)
140.	Motorola XT295 Droid Razr HD	**42** **(0.07%)**	57.14%	24 (0.06%)	52.38%	5.71	00:05:54	0.00%	0 (0.00%)	$0.00 (0.00%)
141.	Nokia Lumia 925	**42** **(0.07%)**	35.71%	15 (0.04%)	26.19%	10.12	00:11:41	0.00%	0 (0.00%)	$0.00 (0.00%)
142.	Samsung GT-E1282T	**42** **(0.07%)**	54.76%	23 (0.06%)	61.90%	2.29	00:01:52	0.00%	0 (0.00%)	$0.00 (0.00%)
143.	Samsung GT-S7500L Galaxy Ace Plus	**42** **(0.07%)**	61.90%	26 (0.06%)	42.86%	6.79	00:09:49	0.00%	0 (0.00%)	$0.00 (0.00%)
144.	Samsung SGH-I727 Galaxy S II Skyrocket	**42** **(0.07%)**	33.33%	14 (0.03%)	83.33%	1.83	00:02:30	0.00%	0 (0.00%)	$0.00 (0.00%)
145.	Huawei Y300 Ascend Y 300	**41** **(0.07%)**	68.29%	28 (0.07%)	56.10%	2.73	00:01:26	0.00%	0 (0.00%)	$0.00 (0.00%)
146.	Samsung SM-T210R Galaxy Tab 3 7.0	**41** **(0.07%)**	80.49%	33 (0.08%)	65.85%	2.63	00:04:38	0.00%	0 (0.00%)	$0.00 (0.00%)
147.	Sony C2104 Xperia L	**41** **(0.07%)**	73.17%	30 (0.07%)	39.02%	5.22	00:04:52	0.00%	0 (0.00%)	$0.00 (0.00%)
148.	SonyEricsson ST25a Xperia U	**41** **(0.07%)**	53.66%	22 (0.05%)	60.98%	3.15	00:03:14	0.00%	0 (0.00%)	$0.00 (0.00%)
149.	Alcatel OT-4030A S-POP	**40** **(0.07%)**	30.00%	12 (0.03%)	40.00%	14.70	00:13:05	0.00%	0 (0.00%)	$0.00 (0.00%)
150.	Asus TF101 Eee Pad Transformer TF101	**40** **(0.07%)**	77.50%	31 (0.07%)	70.00%	3.08	00:01:16	0.00%	0 (0.00%)	$0.00 (0.00%)
151.	Nokia 200 Nokia Asha 200	**40** **(0.07%)**	92.50%	37 (0.09%)	60.00%	1.98	00:01:34	0.00%	0 (0.00%)	$0.00 (0.00%)
152.	Nokia 303 Asha	**40** **(0.07%)**	95.00%	38 (0.09%)	77.50%	1.30	00:00:12	0.00%	0 (0.00%)	$0.00 (0.00%)

152.	[illegible] 303	**40** (0.07%)	[illegible]	[illegible]	[illegible]	[illegible]	[illegible]	0.00%	0 (0.00%)	$0.00 (0.00%)
153.	Samsung GT-S5570L	**40** (0.07%)	67.50%	27 (0.06%)	57.50%	4.90	00:03:43	0.00%	0 (0.00%)	$0.00 (0.00%)
154.	Sony SGPT12 Xperia Tablet S	**40** (0.07%)	40.00%	16 (0.04%)	40.00%	8.15	00:06:54	0.00%	0 (0.00%)	$0.00 (0.00%)
155.	Huawei MediaPad 7 Lite	**39** (0.06%)	46.15%	18 (0.04%)	51.28%	6.00	00:06:02	0.00%	0 (0.00%)	$0.00 (0.00%)
156.	Nokia Asha 202 Asha 202	**39** (0.06%)	94.87%	37 (0.09%)	61.54%	1.82	00:01:12	0.00%	0 (0.00%)	$0.00 (0.00%)
157.	Samsung GT-S6810 Galaxy Fame	**39** (0.06%)	48.72%	19 (0.05%)	53.85%	3.59	00:06:11	0.00%	0 (0.00%)	$0.00 (0.00%)
158.	Samsung GT-S7710 Galaxy Xcover 2	**39** (0.06%)	56.41%	22 (0.05%)	46.15%	3.87	00:02:37	0.00%	0 (0.00%)	$0.00 (0.00%)
159.	Acer A1-810	**38** (0.06%)	57.89%	22 (0.05%)	52.63%	5.39	00:02:21	0.00%	0 (0.00%)	$0.00 (0.00%)
160.	Samsung SCH-I605 Galaxy Note II	**38** (0.06%)	57.89%	22 (0.05%)	73.68%	2.32	00:01:38	0.00%	0 (0.00%)	$0.00 (0.00%)
161.	Google Nexus One HTC Nexus One	**37** (0.06%)	78.38%	29 (0.07%)	54.05%	10.59	00:02:53	0.00%	0 (0.00%)	$0.00 (0.00%)
162.	HTC A510 Wildfire S	**37** (0.06%)	54.05%	20 (0.05%)	48.65%	2.54	00:02:41	0.00%	0 (0.00%)	$0.00 (0.00%)
163.	LG E610 Optimus L5	**37** (0.06%)	59.46%	22 (0.05%)	78.38%	1.38	00:01:33	0.00%	0 (0.00%)	$0.00 (0.00%)
164.	Samsung GT-I8552B Galaxy Grand Quattro	**37** (0.06%)	45.95%	17 (0.04%)	54.05%	3.49	00:03:36	0.00%	0 (0.00%)	$0.00 (0.00%)
165.	Motorola XT907 DROID RAZR M 4G LTE	**36** (0.06%)	69.44%	25 (0.06%)	77.78%	4.53	00:02:06	0.00%	0 (0.00%)	$0.00 (0.00%)
166.	RIM Z10	**36** (0.06%)	88.89%	32 (0.08%)	61.11%	3.36	00:02:08	0.00%	0 (0.00%)	$0.00 (0.00%)
167.	Samsung GT-S5222R Star III Duos	**36** (0.06%)	69.44%	25 (0.06%)	50.00%	4.53	00:03:05	0.00%	0 (0.00%)	$0.00 (0.00%)
168.	Samsung GT-S5310L Galaxy Pocket Neo	**36** (0.06%)	52.78%	19 (0.05%)	41.67%	3.56	00:02:20	0.00%	0 (0.00%)	$0.00 (0.00%)
169.	Sony C6903 Xperia Z1	**36** (0.06%)	97.22%	35 (0.08%)	86.11%	2.94	00:02:16	0.00%	0 (0.00%)	$0.00 (0.00%)
170.	Amazon KFOT Kindle Fire 7	**35** (0.06%)	91.43%	32 (0.08%)	68.57%	2.63	00:01:54	0.00%	0 (0.00%)	$0.00 (0.00%)
171.	Samsung GT-S6810L Galaxy Fame	**35** (0.06%)	57.14%	20 (0.05%)	42.86%	8.49	00:08:53	0.00%	0 (0.00%)	$0.00 (0.00%)
172.	SonyEricsson ST25i Xperia U	**35** (0.06%)	94.29%	33 (0.08%)	85.71%	1.77	00:00:37	0.00%	0 (0.00%)	$0.00 (0.00%)
173.	Alcatel OT-4010A One Touch T'Pop 4010A	**34** (0.06%)	82.35%	28 (0.07%)	70.59%	2.79	00:01:14	0.00%	0 (0.00%)	$0.00 (0.00%)
174.	Alcatel OT-5020A One Touch M'Pop	**34** (0.06%)	73.53%	25 (0.06%)	70.59%	3.94	00:02:43	0.00%	0 (0.00%)	$0.00 (0.00%)
175.	Amazon KFTHW Kindle Fire HDX 7 3rd Gen	**34** (0.06%)	85.29%	29 (0.07%)	70.59%	1.38	00:00:14	0.00%	0 (0.00%)	$0.00 (0.00%)
176.	BlackBerry 9220	**34** (0.06%)	88.24%	30 (0.07%)	64.71%	2.79	00:04:57	0.00%	0 (0.00%)	$0.00 (0.00%)
177.	LG P880 Optimus 4X HD	**34** (0.06%)	52.94%	18 (0.04%)	55.88%	3.76	00:03:46	0.00%	0 (0.00%)	$0.00 (0.00%)
178.	Nokia Lumia 720	**34** (0.06%)	82.35%	28 (0.07%)	50.00%	3.38	00:01:58	0.00%	0 (0.00%)	$0.00 (0.00%)
179.	Samsung SGH-I317 Galaxy Note	**34** (0.06%)	67.65%	23 (0.06%)	88.24%	1.21	00:00:18	0.00%	0 (0.00%)	$0.00 (0.00%)

179.	I317 Galaxy Note II	[illegible]	[illegible]	[illegible]	[illegible]	[illegible]	[illegible]	[illegible]	[illegible]	0.00%	0	(0.00%)	$0.00	(0.00%)
180.	Acer A500 Picasso	**33**	**(0.05%)**	90.91%	30	(0.07%)	51.52%	3.06	00:01:31	0.00%	0	(0.00%)	$0.00	(0.00%)
181.	Nokia 110	**33**	**(0.05%)**	100.00%	33	(0.08%)	45.45%	2.52	00:02:52	0.00%	0	(0.00%)	$0.00	(0.00%)
182.	Samsung GT-P5200 Galaxy Tab 3 10.1 3G	**33**	**(0.05%)**	75.76%	25	(0.06%)	66.67%	3.79	00:04:50	0.00%	0	(0.00%)	$0.00	(0.00%)
183.	Sony ST26i Xperia J	**33**	**(0.05%)**	60.61%	20	(0.05%)	63.64%	1.97	00:02:16	0.00%	0	(0.00%)	$0.00	(0.00%)
184.	HTC PJ401 One S	**32**	**(0.05%)**	78.12%	25	(0.06%)	65.62%	3.41	00:01:53	0.00%	0	(0.00%)	$0.00	(0.00%)
185.	HTC T328E Desire X	**32**	**(0.05%)**	46.88%	15	(0.04%)	75.00%	1.41	00:01:18	0.00%	0	(0.00%)	$0.00	(0.00%)
186.	LG P769 Optimus L9	**32**	**(0.05%)**	78.12%	25	(0.06%)	71.88%	2.50	00:01:17	0.00%	0	(0.00%)	$0.00	(0.00%)
187.	Nokia Lumia 510	**32**	**(0.05%)**	71.88%	23	(0.06%)	56.25%	2.12	00:03:45	0.00%	0	(0.00%)	$0.00	(0.00%)
188.	Samsung GT-I9082L Galaxy Grand Duos	**32**	**(0.05%)**	65.62%	21	(0.05%)	53.12%	2.50	00:01:57	0.00%	0	(0.00%)	$0.00	(0.00%)
189.	Samsung GT-N8013 Galaxy Note 10.1	**32**	**(0.05%)**	78.12%	25	(0.06%)	46.88%	3.72	00:02:38	0.00%	0	(0.00%)	$0.00	(0.00%)
190.	Samsung SGH-M919 Galaxy S IV	**32**	**(0.05%)**	87.50%	28	(0.07%)	78.12%	1.75	00:00:33	0.00%	0	(0.00%)	$0.00	(0.00%)
191.	LG E450g Optimus L5 II	**31**	**(0.05%)**	35.48%	11	(0.03%)	32.26%	5.52	00:04:22	0.00%	0	(0.00%)	$0.00	(0.00%)
192.	Nokia C2-02	**31**	**(0.05%)**	77.42%	24	(0.06%)	51.61%	3.74	00:08:05	0.00%	0	(0.00%)	$0.00	(0.00%)
193.	Sony C5303 Xperia SP	**31**	**(0.05%)**	70.97%	22	(0.05%)	64.52%	1.90	00:01:54	0.00%	0	(0.00%)	$0.00	(0.00%)
194.	SonyEricsson ST27i	**31**	**(0.05%)**	87.10%	27	(0.06%)	64.52%	2.90	00:03:19	0.00%	0	(0.00%)	$0.00	(0.00%)
195.	Barnes and Noble BNTV600 Nook HD+ Tablet	**30**	**(0.05%)**	73.33%	22	(0.05%)	50.00%	4.83	00:03:48	0.00%	0	(0.00%)	$0.00	(0.00%)
196.	HP Slate 7	**30**	**(0.05%)**	60.00%	18	(0.04%)	66.67%	2.30	00:02:03	0.00%	0	(0.00%)	$0.00	(0.00%)
197.	HTC ADR6300 Incredible	**30**	**(0.05%)**	20.00%	6	(0.01%)	43.33%	3.47	00:01:43	0.00%	0	(0.00%)	$0.00	(0.00%)
198.	LG E615f Optimus L5 Dual	**30**	**(0.05%)**	40.00%	12	(0.03%)	40.00%	5.37	00:10:59	0.00%	0	(0.00%)	$0.00	(0.00%)
199.	Nokia C2-01	**30**	**(0.05%)**	93.33%	28	(0.07%)	70.00%	1.60	00:01:37	0.00%	0	(0.00%)	$0.00	(0.00%)
200.	Samsung GT-I9080L Galaxy Grand	**30**	**(0.05%)**	43.33%	13	(0.03%)	70.00%	2.57	00:02:59	0.00%	0	(0.00%)	$0.00	(0.00%)
201.	HTC Desire HD	**29**	**(0.05%)**	51.72%	15	(0.04%)	58.62%	2.41	00:01:02	0.00%	0	(0.00%)	$0.00	(0.00%)
202.	Nokia 112	**29**	**(0.05%)**	86.21%	25	(0.06%)	79.31%	1.66	00:00:51	0.00%	0	(0.00%)	$0.00	(0.00%)
203.	Opera Mini 3	**29**	**(0.05%)**	96.55%	28	(0.07%)	75.86%	1.62	00:00:24	0.00%	0	(0.00%)	$0.00	(0.00%)
204.	PiPO M1	**29**	**(0.05%)**	96.55%	28	(0.07%)	93.10%	1.10	00:00:08	0.00%	0	(0.00%)	$0.00	(0.00%)
205.	Samsung GT-S6310N Galaxy Young	**29**	**(0.05%)**	51.72%	15	(0.04%)	82.76%	2.24	00:03:46	0.00%	0	(0.00%)	$0.00	(0.00%)
206.	Samsung GT-S7560M Galaxy Ace II x	**29**	**(0.05%)**	72.41%	21	(0.05%)	79.31%	1.52	00:01:05	0.00%	0	(0.00%)	$0.00	(0.00%)
207.	Sony C2105 Xperia L	**29**	**(0.05%)**	79.31%	23	(0.06%)	75.86%	1.90	00:00:22	0.00%	0	(0.00%)	$0.00	(0.00%)
208.	SonyEricsson R800i Xperia PLAY	**29**	**(0.05%)**	34.48%	10	(0.02%)	48.28%	5.21	00:09:28	0.00%	0	(0.00%)	$0.00	(0.00%)

209.	Acer B1-A71 Iconia B1-A71	**28**	**(0.05%)**	57.14%	16	(0.04%)	50.00%	5.61	00:02:19	0.00%	0 (0.00%)	$0.00	(0.00%)
210.	LG P714 Optimus L7X	**28**	**(0.05%)**	64.29%	18	(0.04%)	57.14%	3.11	00:03:44	0.00%	0 (0.00%)	$0.00	(0.00%)
211.	Motorola MB865 Atrix 2	**28**	**(0.05%)**	35.71%	10	(0.02%)	42.86%	6.75	00:05:53	0.00%	0 (0.00%)	$0.00	(0.00%)
212.	Nokia 306 Asha 306	**28**	**(0.05%)**	92.86%	26	(0.06%)	75.00%	1.82	00:00:47	0.00%	0 (0.00%)	$0.00	(0.00%)
213.	Nokia 501 Asha 501	**28**	**(0.05%)**	92.86%	26	(0.06%)	75.00%	1.32	00:00:54	0.00%	0 (0.00%)	$0.00	(0.00%)
214.	Nokia Lumia 625	**28**	**(0.05%)**	82.14%	23	(0.06%)	46.43%	3.43	00:02:17	0.00%	0 (0.00%)	$0.00	(0.00%)
215.	Asus ME371MG Fonepad ME371MG	**27**	**(0.04%)**	55.56%	15	(0.04%)	44.44%	6.26	00:05:23	0.00%	0 (0.00%)	$0.00	(0.00%)
216.	HTC 9292 EVO 4G	**27**	**(0.04%)**	33.33%	9	(0.02%)	44.44%	2.19	00:01:59	0.00%	0 (0.00%)	$0.00	(0.00%)
217.	Huawei G510-0251 Ascend	**27**	**(0.04%)**	66.67%	18	(0.04%)	44.44%	5.74	00:04:11	0.00%	0 (0.00%)	$0.00	(0.00%)
218.	LG E612f Optimus L5	**27**	**(0.04%)**	96.30%	26	(0.06%)	59.26%	4.30	00:06:37	0.00%	0 (0.00%)	$0.00	(0.00%)
219.	Motorola xt875 Droid Bionic	**27**	**(0.04%)**	66.67%	18	(0.04%)	48.15%	3.81	00:05:49	0.00%	0 (0.00%)	$0.00	(0.00%)
220.	Samsung GT-S7500 Galaxy Ace Plus	**27**	**(0.04%)**	62.96%	17	(0.04%)	55.56%	2.56	00:03:54	0.00%	0 (0.00%)	$0.00	(0.00%)
221.	Samsung GT-S7562L Galaxy S Duos	**27**	**(0.04%)**	77.78%	21	(0.05%)	37.04%	4.19	00:04:34	0.00%	0 (0.00%)	$0.00	(0.00%)
222.	SonyEricsson LT18i Xperia Arc	**27**	**(0.04%)**	81.48%	22	(0.05%)	88.89%	1.70	00:00:54	0.00%	0 (0.00%)	$0.00	(0.00%)
223.	Amazon KFAPW Kindle Fire HDX 8.9	**26**	**(0.04%)**	23.08%	6	(0.01%)	80.77%	3.15	00:00:56	0.00%	0 (0.00%)	$0.00	(0.00%)
224.	Asus ME172V MeMO Pad ME172V	**26**	**(0.04%)**	53.85%	14	(0.03%)	69.23%	2.73	00:02:20	0.00%	0 (0.00%)	$0.00	(0.00%)
225.	LG F320S G2	**26**	**(0.04%)**	11.54%	3	(0.01%)	50.00%	3.85	00:04:28	0.00%	0 (0.00%)	$0.00	(0.00%)
226.	LG P705g Optimus L7	**26**	**(0.04%)**	57.69%	15	(0.04%)	50.00%	4.69	00:06:03	0.00%	0 (0.00%)	$0.00	(0.00%)
227.	Nokia C2-03	**26**	**(0.04%)**	100.00%	26	(0.06%)	53.85%	1.85	00:00:50	0.00%	0 (0.00%)	$0.00	(0.00%)
228.	Nokia N73	**26**	**(0.04%)**	92.31%	24	(0.06%)	76.92%	1.85	00:01:22	0.00%	0 (0.00%)	$0.00	(0.00%)
229.	Nokia N8-00 N8	**26**	**(0.04%)**	100.00%	26	(0.06%)	69.23%	3.38	00:01:46	0.00%	0 (0.00%)	$0.00	(0.00%)
230.	Sony ST23i Xperia miro	**26**	**(0.04%)**	80.77%	21	(0.05%)	73.08%	1.46	00:00:37	0.00%	0 (0.00%)	$0.00	(0.00%)
231.	BlackBerry 9930	**25**	**(0.04%)**	84.00%	21	(0.05%)	68.00%	1.52	00:02:03	0.00%	0 (0.00%)	$0.00	(0.00%)
232.	HTC Desire	**25**	**(0.04%)**	44.00%	11	(0.03%)	64.00%	8.28	00:09:37	0.00%	0 (0.00%)	$0.00	(0.00%)
233.	Nokia 300 Asha 300	**25**	**(0.04%)**	100.00%	25	(0.06%)	92.00%	1.52	00:01:04	0.00%	0 (0.00%)	$0.00	(0.00%)
234.	Nokia Asha 210 Asha 210	**25**	**(0.04%)**	96.00%	24	(0.06%)	80.00%	2.08	00:01:16	0.00%	0 (0.00%)	$0.00	(0.00%)
235.	Nokia E5-00 Mystic	**25**	**(0.04%)**	100.00%	25	(0.06%)	56.00%	2.44	00:04:15	0.00%	0 (0.00%)	$0.00	(0.00%)
236.	Samsung GT-I9001	**25**	**(0.04%)**	64.00%	16	(0.04%)	56.00%	2.84	00:03:07	0.00%	0 (0.00%)	$0.00	(0.00%)
237.	Samsung GT-N8013 Galaxy Note 10.1 WiFi	**25**	**(0.04%)**	76.00%	19	(0.05%)	60.00%	2.36	00:04:32	0.00%	0 (0.00%)	$0.00	(0.00%)
238.	Samsung GT-S5302 Galaxy	**25**	**(0.04%)**	84.00%	21	(0.05%)	56.00%	1.96	00:00:52	0.00%	0 (0.00%)	$0.00	(0.00%)

	Pocket Duos									
239.	Samsung SM-P600 Galaxy Note 10.1 2014	**25** **(0.04%)**	76.00%	19 (0.05%)	56.00%	2.60	00:00:33	0.00%	0 (0.00%)	$0.00 (0.00%)
240.	Sony C1504 Xperia E	**25** **(0.04%)**	68.00%	17 (0.04%)	44.00%	2.76	00:03:09	0.00%	0 (0.00%)	$0.00 (0.00%)
241.	Motorola XT894 Droid 4	**24** **(0.04%)**	87.50%	21 (0.05%)	62.50%	3.08	00:01:05	0.00%	0 (0.00%)	$0.00 (0.00%)
242.	Nokia 305	**24** **(0.04%)**	100.00%	24 (0.06%)	83.33%	2.50	00:02:32	0.00%	0 (0.00%)	$0.00 (0.00%)
243.	Nokia X2-01	**24** **(0.04%)**	100.00%	24 (0.06%)	75.00%	1.54	00:01:11	0.00%	0 (0.00%)	$0.00 (0.00%)
244.	Samsung GT-I8150 Galaxy W	**24** **(0.04%)**	58.33%	14 (0.03%)	62.50%	3.42	00:01:11	0.00%	0 (0.00%)	$0.00 (0.00%)
245.	Samsung GT-S6500D Galaxy Mini 2	**24** **(0.04%)**	87.50%	21 (0.05%)	75.00%	1.62	00:00:23	0.00%	0 (0.00%)	$0.00 (0.00%)
246.	Samsung SPH-D710 Galaxy SII Epic 4G Touch	**24** **(0.04%)**	87.50%	21 (0.05%)	70.83%	1.62	00:00:47	0.00%	0 (0.00%)	$0.00 (0.00%)
247.	Sony ST21i Xperia Tipo	**24** **(0.04%)**	70.83%	17 (0.04%)	75.00%	2.42	00:00:38	0.00%	0 (0.00%)	$0.00 (0.00%)
248.	Asus TF700T Transformer Pad TF700T	**23** **(0.04%)**	82.61%	19 (0.05%)	60.87%	2.22	00:00:31	0.00%	0 (0.00%)	$0.00 (0.00%)
249.	LG P760 Optimus L9	**23** **(0.04%)**	86.96%	20 (0.05%)	82.61%	1.39	00:01:04	0.00%	0 (0.00%)	$0.00 (0.00%)
250.	Motorola XT1053 Moto X	**23** **(0.04%)**	60.87%	14 (0.03%)	52.17%	5.26	00:04:26	0.00%	0 (0.00%)	$0.00 (0.00%)
251.	Motorola XT920 RAZR D3	**23** **(0.04%)**	52.17%	12 (0.03%)	69.57%	2.65	00:01:24	0.00%	0 (0.00%)	$0.00 (0.00%)
252.	Samsung GT-P5113 Galaxy Tab 2 10.1 WiFi	**23** **(0.04%)**	69.57%	16 (0.04%)	52.17%	4.87	00:08:57	0.00%	0 (0.00%)	$0.00 (0.00%)
253.	Samsung SGH-I337M Galaxy S IV	**23** **(0.04%)**	73.91%	17 (0.04%)	65.22%	3.22	00:00:52	0.00%	0 (0.00%)	$0.00 (0.00%)
254.	Motorola MZ604 Xoom	**22** **(0.04%)**	31.82%	7 (0.02%)	63.64%	3.73	00:02:01	0.00%	0 (0.00%)	$0.00 (0.00%)
255.	Samsung GT-S6310 Galaxy Young	**22** **(0.04%)**	77.27%	17 (0.04%)	54.55%	2.91	00:03:31	0.00%	0 (0.00%)	$0.00 (0.00%)
256.	Samsung GT-S6500 Galaxy Mini 2	**22** **(0.04%)**	72.73%	16 (0.04%)	81.82%	2.23	00:00:57	0.00%	0 (0.00%)	$0.00 (0.00%)
257.	Samsung SCH-I747 Galaxy S III	**22** **(0.04%)**	63.64%	14 (0.03%)	54.55%	4.91	00:01:47	0.00%	0 (0.00%)	$0.00 (0.00%)
258.	Amazon KFJWI Kindle Fire HD 8.9	**21** **(0.03%)**	95.24%	20 (0.05%)	66.67%	2.95	00:01:01	0.00%	0 (0.00%)	$0.00 (0.00%)
259.	HTC Z710 Sensation	**21** **(0.03%)**	57.14%	12 (0.03%)	76.19%	1.81	00:01:30	0.00%	0 (0.00%)	$0.00 (0.00%)
260.	Huawei Y210-0151 Ascend Y210	**21** **(0.03%)**	66.67%	14 (0.03%)	52.38%	4.33	00:07:14	0.00%	0 (0.00%)	$0.00 (0.00%)
261.	Motorola XT615	**21** **(0.03%)**	90.48%	19 (0.05%)	66.67%	1.95	00:02:37	0.00%	0 (0.00%)	$0.00 (0.00%)
262.	Samsung GT-I9100P Galaxy S II NFC	**21** **(0.03%)**	95.24%	20 (0.05%)	80.95%	1.71	00:00:25	0.00%	0 (0.00%)	$0.00 (0.00%)
263.	Samsung GT-S5839i Galaxy Ace	**21** **(0.03%)**	100.00%	21 (0.05%)	85.71%	1.33	00:00:16	0.00%	0 (0.00%)	$0.00 (0.00%)
264.	Samsung SPH-L710 Galaxy SIII	**21** **(0.03%)**	80.95%	17 (0.04%)	61.90%	3.05	00:00:55	0.00%	0 (0.00%)	$0.00 (0.00%)
	Toshiba AT300									

265.	Toshiba AT300 Regza AT300	**21** **(0.03%)**	38.10%	8 (0.02%)	71.43%	3.19	00:01:31	0.00%	0 (0.00%)	$0.00 (0.00%)
266.	Lenovo IdeaTab S6000-H	**20** **(0.03%)**	50.00%	10 (0.02%)	65.00%	2.80	00:02:29	0.00%	0 (0.00%)	$0.00 (0.00%)
267.	LG E450f Optimus L5 II	**20** **(0.03%)**	65.00%	13 (0.03%)	50.00%	3.35	00:03:34	0.00%	0 (0.00%)	$0.00 (0.00%)
268.	Micromax A110 Superfone Canvas 2	**20** **(0.03%)**	80.00%	16 (0.04%)	80.00%	1.70	00:00:20	0.00%	0 (0.00%)	$0.00 (0.00%)
269.	Samsung GT-I9105P Galaxy SII Plus	**20** **(0.03%)**	85.00%	17 (0.04%)	80.00%	1.70	00:00:36	0.00%	0 (0.00%)	$0.00 (0.00%)
270.	Samsung GT-P6200 GALAXY Tab 7.0 Plus	**20** **(0.03%)**	75.00%	15 (0.04%)	80.00%	1.20	00:00:24	0.00%	0 (0.00%)	$0.00 (0.00%)
271.	Samsung SGH-I537 Galaxy S4 Active	**20** **(0.03%)**	70.00%	14 (0.03%)	70.00%	4.50	00:04:26	0.00%	0 (0.00%)	$0.00 (0.00%)
272.	ZTE V768 Concord	**20** **(0.03%)**	70.00%	14 (0.03%)	65.00%	1.95	00:00:35	0.00%	0 (0.00%)	$0.00 (0.00%)
273.	BlackBerry Z10	**19** **(0.03%)**	78.95%	15 (0.04%)	52.63%	2.58	00:01:26	0.00%	0 (0.00%)	$0.00 (0.00%)
274.	Feiteng GT-I930(	**19** **(0.03%)**	73.68%	14 (0.03%)	68.42%	2.11	00:01:24	0.00%	0 (0.00%)	$0.00 (0.00%)
275.	Huawei S7-301w MediaPad 10 Link	**19** **(0.03%)**	42.11%	8 (0.02%)	21.05%	6.53	00:03:14	0.00%	0 (0.00%)	$0.00 (0.00%)
276.	Motorola XT303 Motosmart Me	**19** **(0.03%)**	84.21%	16 (0.04%)	63.16%	4.53	00:02:56	0.00%	0 (0.00%)	$0.00 (0.00%)
277.	Nokia 206	**19** **(0.03%)**	94.74%	18 (0.04%)	84.21%	1.26	00:00:11	0.00%	0 (0.00%)	$0.00 (0.00%)
278.	Samsung GT-P7300 Galaxy Tab 8.9	**19** **(0.03%)**	84.21%	16 (0.04%)	78.95%	1.84	00:00:32	0.00%	0 (0.00%)	$0.00 (0.00%)
279.	Samsung GT-S5830D Galaxy Ace	**19** **(0.03%)**	26.32%	5 (0.01%)	47.37%	8.53	00:10:14	0.00%	0 (0.00%)	$0.00 (0.00%)
280.	Samsung SGH-I747 Galaxy SIII	**19** **(0.03%)**	68.42%	13 (0.03%)	52.63%	9.32	00:06:17	0.00%	0 (0.00%)	$0.00 (0.00%)
281.	SonyEricsson LT15i Xperia Arc	**19** **(0.03%)**	68.42%	13 (0.03%)	78.95%	1.47	00:01:36	0.00%	0 (0.00%)	$0.00 (0.00%)
282.	SonyEricsson MT11i Xperia Neo V	**19** **(0.03%)**	89.47%	17 (0.04%)	63.16%	2.26	00:00:33	0.00%	0 (0.00%)	$0.00 (0.00%)
283.	SonyEricsson WT19i Live	**19** **(0.03%)**	47.37%	9 (0.02%)	89.47%	2.84	00:03:22	0.00%	0 (0.00%)	$0.00 (0.00%)
284.	Barnes and Noble BNTV400 Nook HD Tablet	**18** **(0.03%)**	66.67%	12 (0.03%)	72.22%	4.94	00:02:13	0.00%	0 (0.00%)	$0.00 (0.00%)
285.	HTC ADR6350 Droid Incredible 2	**18** **(0.03%)**	72.22%	13 (0.03%)	66.67%	4.56	00:02:37	0.00%	0 (0.00%)	$0.00 (0.00%)
286.	LG E510 Optimus Hub	**18** **(0.03%)**	33.33%	6 (0.01%)	16.67%	14.28	00:21:53	0.00%	0 (0.00%)	$0.00 (0.00%)
287.	LG P350 Optimus Me	**18** **(0.03%)**	61.11%	11 (0.03%)	55.56%	2.28	00:03:04	0.00%	0 (0.00%)	$0.00 (0.00%)
288.	Nokia C6-01	**18** **(0.03%)**	100.00%	18 (0.04%)	5.56%	7.39	00:31:50	0.00%	0 (0.00%)	$0.00 (0.00%)
289.	Samsung GT-S5300B Galaxy Pocket	**18** **(0.03%)**	61.11%	11 (0.03%)	50.00%	3.56	00:04:54	0.00%	0 (0.00%)	$0.00 (0.00%)
290.	Samsung GT-S5301L Galaxy Pocket Plus	**18** **(0.03%)**	50.00%	9 (0.02%)	55.56%	3.89	00:02:15	0.00%	0 (0.00%)	$0.00 (0.00%)
291.	Samsung GT-S5570I Galaxy Pop Plus	**18** **(0.03%)**	94.44%	17 (0.04%)	44.44%	5.22	00:08:58	0.00%	0 (0.00%)	$0.00 (0.00%)
[illegible]	Samsung SGH [illegible]	[illegible]	[illegible]	[illegible]	[illegible]	[illegible]	[illegible]	[illegible]	[illegible]	[illegible]

292.	T599N	**18** **(0.03%)**	72.22%	13 (0.03%)	72.22%	3.67	00:01:29	0.00%	0 (0.00%)	$0.00 (0.00%)
293.	Samsung SGH-T889 Galaxy Note II	**18** **(0.03%)**	100.00%	18 (0.04%)	66.67%	1.50	00:00:12	0.00%	0 (0.00%)	$0.00 (0.00%)
294.	Samsung SPH-L900 Galaxy Note II	**18** **(0.03%)**	94.44%	17 (0.04%)	83.33%	2.56	00:01:12	0.00%	0 (0.00%)	$0.00 (0.00%)
295.	Sony C1905 Xperia M	**18** **(0.03%)**	72.22%	13 (0.03%)	72.22%	2.33	00:01:05	0.00%	0 (0.00%)	$0.00 (0.00%)
296.	Sony ST23a Xperia miro	**18** **(0.03%)**	66.67%	12 (0.03%)	33.33%	4.33	00:05:29	0.00%	0 (0.00%)	$0.00 (0.00%)
297.	Acer A700	**17** **(0.03%)**	47.06%	8 (0.02%)	76.47%	2.18	00:00:46	0.00%	0 (0.00%)	$0.00 (0.00%)
298.	Asus ME301T MeMO Pad Smart 10	**17** **(0.03%)**	76.47%	13 (0.03%)	70.59%	2.06	00:00:46	0.00%	0 (0.00%)	$0.00 (0.00%)
299.	BlackBerry 9380 Blackberry 9380 Curve	**17** **(0.03%)**	76.47%	13 (0.03%)	70.59%	1.82	00:01:42	0.00%	0 (0.00%)	$0.00 (0.00%)
300.	Huawei U8667	**17** **(0.03%)**	35.29%	6 (0.01%)	58.82%	2.71	00:03:29	0.00%	0 (0.00%)	$0.00 (0.00%)
301.	Lenovo A1000-F	**17** **(0.03%)**	41.18%	7 (0.02%)	64.71%	3.35	00:02:05	0.00%	0 (0.00%)	$0.00 (0.00%)
302.	LG P500h Optimus One	**17** **(0.03%)**	70.59%	12 (0.03%)	58.82%	3.00	00:02:58	0.00%	0 (0.00%)	$0.00 (0.00%)
303.	Motorola XT1080 Ultra	**17** **(0.03%)**	76.47%	13 (0.03%)	58.82%	3.06	00:02:36	0.00%	0 (0.00%)	$0.00 (0.00%)
304.	Samsung GT-I9100G Galaxy S II	**17** **(0.03%)**	70.59%	12 (0.03%)	70.59%	1.76	00:00:32	0.00%	0 (0.00%)	$0.00 (0.00%)
305.	Samsung GT-I9305 Galaxy S3 LTE International	**17** **(0.03%)**	94.12%	16 (0.04%)	88.24%	1.59	00:00:35	0.00%	0 (0.00%)	$0.00 (0.00%)
306.	Samsung SGH-T999 Galaxy SIII	**17** **(0.03%)**	76.47%	13 (0.03%)	47.06%	4.71	00:04:07	0.00%	0 (0.00%)	$0.00 (0.00%)
307.	Sony ST21i2 Xperia Tipo Dual	**17** **(0.03%)**	52.94%	9 (0.02%)	64.71%	2.29	00:02:00	0.00%	0 (0.00%)	$0.00 (0.00%)
308.	SonyEricsson C6602 Xperia Z	**17** **(0.03%)**	82.35%	14 (0.03%)	76.47%	1.82	00:02:36	0.00%	0 (0.00%)	$0.00 (0.00%)
309.	SonyEricsson WT19a Live	**17** **(0.03%)**	76.47%	13 (0.03%)	58.82%	8.82	00:12:41	0.00%	0 (0.00%)	$0.00 (0.00%)
310.	LG E400 Optimus L3	**16** **(0.03%)**	93.75%	15 (0.04%)	56.25%	7.25	00:09:14	0.00%	0 (0.00%)	$0.00 (0.00%)
311.	Micromax A116 Canvas HD	**16** **(0.03%)**	75.00%	12 (0.03%)	62.50%	2.94	00:05:21	0.00%	0 (0.00%)	$0.00 (0.00%)
312.	Motorola XT910 RAZR XT910 Spider	**16** **(0.03%)**	87.50%	14 (0.03%)	87.50%	1.19	00:00:11	0.00%	0 (0.00%)	$0.00 (0.00%)
313.	Nokia 114	**16** **(0.03%)**	93.75%	15 (0.04%)	56.25%	2.94	00:02:45	0.00%	0 (0.00%)	$0.00 (0.00%)
314.	Samsung GT-I9195L Galaxy S4 Mini	**16** **(0.03%)**	87.50%	14 (0.03%)	50.00%	3.12	00:03:20	0.00%	0 (0.00%)	$0.00 (0.00%)
315.	Samsung GT-S6802 Galaxy Ace Duos	**16** **(0.03%)**	93.75%	15 (0.04%)	68.75%	1.94	00:01:03	0.00%	0 (0.00%)	$0.00 (0.00%)
316.	Samsung SM-N900 Galaxy Note 3	**16** **(0.03%)**	87.50%	14 (0.03%)	81.25%	2.00	00:00:36	0.00%	0 (0.00%)	$0.00 (0.00%)
317.	Sony SO-04D Xperia GX SO-04D for DoCoMo	**16** **(0.03%)**	25.00%	4 (0.01%)	37.50%	7.44	00:03:45	0.00%	0 (0.00%)	$0.00 (0.00%)
318.	Sprint X325c/APX325CKT EVO 4G LTE	**16** **(0.03%)**	81.25%	13 (0.03%)	68.75%	1.94	00:00:42	0.00%	0 (0.00%)	$0.00 (0.00%)
319.	ZTE N9120 Avid 4G	**16** **(0.03%)**	56.25%	9 (0.02%)	100.00%	1.00	00:00:00	0.00%	0 (0.00%)	$0.00 (0.00%)

320.	BlackBerry 9860 Monza	**15** **(0.02%)**	86.67%	13 (0.03%)	66.67%	4.40	00:04:34	0.00%	0 (0.00%)	$0.00 (0.00%)
321.	LG D802 G2	**15** **(0.02%)**	93.33%	14 (0.03%)	93.33%	1.07	00:00:21	0.00%	0 (0.00%)	$0.00 (0.00%)
322.	Nokia 5800 XpressMusic XpressMusic	**15** **(0.02%)**	93.33%	14 (0.03%)	86.67%	1.13	00:00:40	0.00%	0 (0.00%)	$0.00 (0.00%)
323.	Samsung GT-I8530	**15** **(0.02%)**	66.67%	10 (0.02%)	53.33%	13.33	00:13:14	0.00%	0 (0.00%)	$0.00 (0.00%)
324.	Samsung GT-I8552 Galaxy Grand Quattro	**15** **(0.02%)**	100.00%	15 (0.04%)	93.33%	1.20	00:00:13	0.00%	0 (0.00%)	$0.00 (0.00%)
325.	Samsung GT-I9205 Galaxy Mega 6.3	**15** **(0.02%)**	60.00%	9 (0.02%)	93.33%	1.07	00:00:02	0.00%	0 (0.00%)	$0.00 (0.00%)
326.	Samsung GT-S5300 Galaxy Pocket	**15** **(0.02%)**	86.67%	13 (0.03%)	73.33%	2.07	00:01:10	0.00%	0 (0.00%)	$0.00 (0.00%)
327.	Samsung GT-S5670 Galaxy Fit	**15** **(0.02%)**	80.00%	12 (0.03%)	60.00%	3.20	00:01:44	0.00%	0 (0.00%)	$0.00 (0.00%)
328.	Samsung GT-S6312 Galaxy Young	**15** **(0.02%)**	53.33%	8 (0.02%)	60.00%	1.80	00:02:06	0.00%	0 (0.00%)	$0.00 (0.00%)
329.	Samsung SPH-D710BST Galaxy SII Epic 4G Touch	**15** **(0.02%)**	60.00%	9 (0.02%)	66.67%	5.20	00:04:31	0.00%	0 (0.00%)	$0.00 (0.00%)
330.	Samsung SPH-L720 Galaxy S IV	**15** **(0.02%)**	86.67%	13 (0.03%)	66.67%	2.20	00:00:28	0.00%	0 (0.00%)	$0.00 (0.00%)
331.	Samsung YP-G70 Galaxy Player 5	**15** **(0.02%)**	73.33%	11 (0.03%)	66.67%	4.93	00:03:05	0.00%	0 (0.00%)	$0.00 (0.00%)
332.	Sony LT25i Xperia V	**15** **(0.02%)**	93.33%	14 (0.03%)	80.00%	1.47	00:00:20	0.00%	0 (0.00%)	$0.00 (0.00%)
333.	SonyEricsson E15a Xperia X8	**15** **(0.02%)**	93.33%	14 (0.03%)	60.00%	2.53	00:04:28	0.00%	0 (0.00%)	$0.00 (0.00%)
334.	Toshiba AT100	**15** **(0.02%)**	73.33%	11 (0.03%)	60.00%	3.00	00:01:20	0.00%	0 (0.00%)	$0.00 (0.00%)
335.	Acer A200 Picasso_E	**14** **(0.02%)**	71.43%	10 (0.02%)	64.29%	3.71	00:08:02	0.00%	0 (0.00%)	$0.00 (0.00%)
336.	Asus TF300TG Transformer Pad TF300T	**14** **(0.02%)**	42.86%	6 (0.01%)	42.86%	3.36	00:01:19	0.00%	0 (0.00%)	$0.00 (0.00%)
337.	HTC A310e Explorer	**14** **(0.02%)**	92.86%	13 (0.03%)	64.29%	1.64	00:02:30	0.00%	0 (0.00%)	$0.00 (0.00%)
338.	HTC Inspire 4G	**14** **(0.02%)**	85.71%	12 (0.03%)	64.29%	1.64	00:00:16	0.00%	0 (0.00%)	$0.00 (0.00%)
339.	Huawei P6-U06 Ascend P6	**14** **(0.02%)**	71.43%	10 (0.02%)	57.14%	5.00	00:04:55	0.00%	0 (0.00%)	$0.00 (0.00%)
340.	LG E410 Optimus L1 II	**14** **(0.02%)**	28.57%	4 (0.01%)	57.14%	1.50	00:01:26	0.00%	0 (0.00%)	$0.00 (0.00%)
341.	RIM KBD	**14** **(0.02%)**	92.86%	13 (0.03%)	78.57%	1.50	00:00:30	0.00%	0 (0.00%)	$0.00 (0.00%)
342.	Samsung GT-I9003 Galaxy SL	**14** **(0.02%)**	85.71%	12 (0.03%)	78.57%	1.64	00:00:19	0.00%	0 (0.00%)	$0.00 (0.00%)
343.	Samsung GT-I9152 Galaxy Mega 5.8	**14** **(0.02%)**	100.00%	14 (0.03%)	64.29%	1.86	00:02:11	0.00%	0 (0.00%)	$0.00 (0.00%)
344.	Samsung GT-I9305T Galaxy S3 LTE	**14** **(0.02%)**	92.86%	13 (0.03%)	78.57%	1.79	00:02:42	0.00%	0 (0.00%)	$0.00 (0.00%)
345.	Samsung GT-N5120 Galaxy Note 8.0 LTE	**14** **(0.02%)**	35.71%	5 (0.01%)	64.29%	3.21	00:02:13	0.00%	0 (0.00%)	$0.00 (0.00%)
346.	Samsung SCH-R720 Admire	**14** **(0.02%)**	57.14%	8 (0.02%)	92.86%	1.43	00:00:12	0.00%	0 (0.00%)	$0.00 (0.00%)

347.	Samsung SM-N900A Galaxy Note 3	**14**	**(0.02%)**	100.00%	14	(0.03%)	85.71%	1.71	00:00:15	0.00%	0	(0.00%)	$0.00	(0.00%)
348.	Asus Eee Pad TF201 Transformer Prime	**13**	**(0.02%)**	92.31%	12	(0.03%)	84.62%	1.69	00:00:15	0.00%	0	(0.00%)	$0.00	(0.00%)
349.	BlackBerry 9650 Bold	**13**	**(0.02%)**	92.31%	12	(0.03%)	46.15%	2.00	00:03:28	0.00%	0	(0.00%)	$0.00	(0.00%)
350.	DoCoMo SO-04D Xperia GX	**13**	**(0.02%)**	76.92%	10	(0.02%)	76.92%	1.77	00:01:08	0.00%	0	(0.00%)	$0.00	(0.00%)
351.	HTC 1000C<br><br>Desire C	**13**	**(0.02%)**	46.15%	6	(0.01%)	69.23%	1.38	00:01:27	0.00%	0	(0.00%)	$0.00	(0.00%)
352.	Motorola MB526	**13**	**(0.02%)**	61.54%	8	(0.02%)	69.23%	3.54	00:05:14	0.00%	0	(0.00%)	$0.00	(0.00%)
353.	Motorola MB612 XPRT	**13**	**(0.02%)**	7.69%	1	(0.00%)	46.15%	2.38	00:09:26	0.00%	0	(0.00%)	$0.00	(0.00%)
354.	Nokia 500	**13**	**(0.02%)**	100.00%	13	(0.03%)	53.85%	2.08	00:01:04	0.00%	0	(0.00%)	$0.00	(0.00%)
355.	Samsung GT-B5510L Galaxy Y Pro	**13**	**(0.02%)**	46.15%	6	(0.01%)	30.77%	3.92	00:06:04	0.00%	0	(0.00%)	$0.00	(0.00%)
356.	Samsung GT-C3312R Rex 60 Duos	**13**	**(0.02%)**	69.23%	9	(0.02%)	69.23%	1.62	00:01:12	0.00%	0	(0.00%)	$0.00	(0.00%)
357.	Samsung GT-I8160P Galaxy Ace 2 NFC	**13**	**(0.02%)**	84.62%	11	(0.03%)	84.62%	1.31	00:00:05	0.00%	0	(0.00%)	$0.00	(0.00%)
358.	Samsung GT-I8260L Galaxy Core	**13**	**(0.02%)**	61.54%	8	(0.02%)	30.77%	4.15	00:01:27	0.00%	0	(0.00%)	$0.00	(0.00%)
359.	Samsung GT-P6800 Galaxy Tab 7.7	**13**	**(0.02%)**	61.54%	8	(0.02%)	53.85%	2.92	00:07:43	0.00%	0	(0.00%)	$0.00	(0.00%)
360.	Samsung GT-S5301 Galaxy Pocket Plus	**13**	**(0.02%)**	100.00%	13	(0.03%)	69.23%	1.69	00:00:46	0.00%	0	(0.00%)	$0.00	(0.00%)
361.	Samsung GT-S5360T Galaxy Y	**13**	**(0.02%)**	46.15%	6	(0.01%)	38.46%	3.77	00:05:42	0.00%	0	(0.00%)	$0.00	(0.00%)
362.	Samsung GT-S5660 Galaxy Gio	**13**	**(0.02%)**	84.62%	11	(0.03%)	69.23%	1.77	00:00:26	0.00%	0	(0.00%)	$0.00	(0.00%)
363.	Samsung GT-S7392 Galaxy Trend	**13**	**(0.02%)**	46.15%	6	(0.01%)	92.31%	1.08	00:00:22	0.00%	0	(0.00%)	$0.00	(0.00%)
364.	Samsung SCH-I337 Galaxy S IV	**13**	**(0.02%)**	100.00%	13	(0.03%)	92.31%	1.15	00:00:05	0.00%	0	(0.00%)	$0.00	(0.00%)
365.	Samsung SGH-I317M Galaxy Note II	**13**	**(0.02%)**	92.31%	12	(0.03%)	84.62%	1.38	00:00:19	0.00%	0	(0.00%)	$0.00	(0.00%)
366.	Samsung SGH-i717 Galaxy Note	**13**	**(0.02%)**	92.31%	12	(0.03%)	76.92%	1.54	00:00:21	0.00%	0	(0.00%)	$0.00	(0.00%)
367.	Sony C5302 Xperia SP	**13**	**(0.02%)**	38.46%	5	(0.01%)	46.15%	2.85	00:03:53	0.00%	0	(0.00%)	$0.00	(0.00%)
368.	SonyEricsson E10a Xperia X10 Mini pro	**13**	**(0.02%)**	69.23%	9	(0.02%)	23.08%	6.00	00:10:12	0.00%	0	(0.00%)	$0.00	(0.00%)
369.	SonyEricsson MK16 Xperia Pro	**13**	**(0.02%)**	69.23%	9	(0.02%)	69.23%	2.62	00:03:25	0.00%	0	(0.00%)	$0.00	(0.00%)
370.	SonyEricsson ST27a Xperia Go	**13**	**(0.02%)**	84.62%	11	(0.03%)	53.85%	5.38	00:06:40	0.00%	0	(0.00%)	$0.00	(0.00%)
371.	ZTE V791	**13**	**(0.02%)**	100.00%	13	(0.03%)	84.62%	1.38	00:00:30	0.00%	0	(0.00%)	$0.00	(0.00%)
372.	Acer Iconia B1-710	**12**	**(0.02%)**	75.00%	9	(0.02%)	75.00%	1.75	00:05:47	0.00%	0	(0.00%)	$0.00	(0.00%)

373.	HTC ADR6410LVW 4G Droid Incredible 4G LTE	**12** **(0.02%)**	50.00%	6 (0.01%)	50.00%	4.17	00:01:37	0.00%	0 (0.00%)	$0.00 (0.00%)
374.	LG E460 Optimus L5 II	**12** **(0.02%)**	91.67%	11 (0.03%)	75.00%	1.83	00:00:51	0.00%	0 (0.00%)	$0.00 (0.00%)
375.	LG F160K Optimus LTE2	**12** **(0.02%)**	50.00%	6 (0.01%)	50.00%	3.67	00:01:14	0.00%	0 (0.00%)	$0.00 (0.00%)
376.	LG P708G Optimus L7	**12** **(0.02%)**	83.33%	10 (0.02%)	66.67%	4.08	00:01:56	0.00%	0 (0.00%)	$0.00 (0.00%)
377.	Motorola MB886 Atrix HD	**12** **(0.02%)**	66.67%	8 (0.02%)	83.33%	2.50	00:00:28	0.00%	0 (0.00%)	$0.00 (0.00%)
378.	Motorola XT862 Droid 3	**12** **(0.02%)**	75.00%	9 (0.02%)	66.67%	3.08	00:03:25	0.00%	0 (0.00%)	$0.00 (0.00%)
379.	Mozilla Firefox OS	**12** **(0.02%)**	100.00%	12 (0.03%)	41.67%	7.42	00:03:30	0.00%	0 (0.00%)	$0.00 (0.00%)
380.	Nokia 5230 Nuron	**12** **(0.02%)**	91.67%	11 (0.03%)	58.33%	1.92	00:00:35	0.00%	0 (0.00%)	$0.00 (0.00%)
381.	Nokia Lumia 1020	**12** **(0.02%)**	75.00%	9 (0.02%)	66.67%	2.25	00:02:12	0.00%	0 (0.00%)	$0.00 (0.00%)
382.	Orange Daytona	**12** **(0.02%)**	91.67%	11 (0.03%)	66.67%	1.92	00:01:52	0.00%	0 (0.00%)	$0.00 (0.00%)
383.	Samsung GT-C3262	**12** **(0.02%)**	91.67%	11 (0.03%)	75.00%	1.42	00:00:45	0.00%	0 (0.00%)	$0.00 (0.00%)
384.	Samsung GT-I9200 Galaxy Mega 6.3	**12** **(0.02%)**	83.33%	10 (0.02%)	75.00%	1.92	00:00:54	0.00%	0 (0.00%)	$0.00 (0.00%)
385.	Samsung GT-P1010 Galaxy Tab	**12** **(0.02%)**	100.00%	12 (0.03%)	41.67%	7.08	00:08:20	0.00%	0 (0.00%)	$0.00 (0.00%)
386.	Samsung GT-P6210 Samsung Galaxy Tab 7.0 Plus	**12** **(0.02%)**	25.00%	3 (0.01%)	41.67%	3.33	00:03:36	0.00%	0 (0.00%)	$0.00 (0.00%)
387.	Samsung GT-P7100 Galaxy Tab 10.1V	**12** **(0.02%)**	58.33%	7 (0.02%)	75.00%	2.17	00:01:16	0.00%	0 (0.00%)	$0.00 (0.00%)
388.	Samsung GT-S6810M Galaxy Fame	**12** **(0.02%)**	66.67%	8 (0.02%)	83.33%	4.33	00:03:01	0.00%	0 (0.00%)	$0.00 (0.00%)
389.	Samsung GT-S6812 Galaxy Fame	**12** **(0.02%)**	91.67%	11 (0.03%)	41.67%	3.58	00:02:06	0.00%	0 (0.00%)	$0.00 (0.00%)
390.	Samsung SGH-I997 Infuse	**12** **(0.02%)**	83.33%	10 (0.02%)	58.33%	4.17	00:05:07	0.00%	0 (0.00%)	$0.00 (0.00%)
391.	Samsung SHV-E250S Galaxy Note II	**12** **(0.02%)**	58.33%	7 (0.02%)	33.33%	5.25	00:08:40	0.00%	0 (0.00%)	$0.00 (0.00%)
392.	Samsung SM-N900V Galaxy Note 3	**12** **(0.02%)**	75.00%	9 (0.02%)	91.67%	1.08	00:00:02	0.00%	0 (0.00%)	$0.00 (0.00%)
393.	Acer A210	**11** **(0.02%)**	81.82%	9 (0.02%)	100.00%	1.00	00:00:00	0.00%	0 (0.00%)	$0.00 (0.00%)
394.	Alcatel OT-4007D One Touch Pixi 4007D	**11** **(0.02%)**	100.00%	11 (0.03%)	45.45%	2.09	00:01:22	0.00%	0 (0.00%)	$0.00 (0.00%)
395.	Alcatel OT-6030A One Touch Idol	**11** **(0.02%)**	90.91%	10 (0.02%)	63.64%	2.09	00:05:36	0.00%	0 (0.00%)	$0.00 (0.00%)
396.	Asus Fonepad 7	**11** **(0.02%)**	81.82%	9 (0.02%)	72.73%	1.36	00:00:32	0.00%	0 (0.00%)	$0.00 (0.00%)
397.	HP Mesquite	**11** **(0.02%)**	81.82%	9 (0.02%)	72.73%	1.91	00:00:21	0.00%	0 (0.00%)	$0.00 (0.00%)
398.	HTC One 801e One	**11** **(0.02%)**	45.45%	5 (0.01%)	45.45%	3.91	00:01:46	0.00%	0 (0.00%)	$0.00 (0.00%)
399.	Huawei C8651 Evolution II	**11** **(0.02%)**	72.73%	8 (0.02%)	81.82%	1.36	00:00:37	0.00%	0 (0.00%)	$0.00 (0.00%)

400.	Huawei U8185 Ascend Y100	**11**	**(0.02%)**	45.45%	5	(0.01%)	54.55%	2.09	00:01:01	0.00%	0	(0.00%)	$0.00	(0.00%)
401.	Huawei U8665 Fusion 2	**11**	**(0.02%)**	81.82%	9	(0.02%)	81.82%	2.09	00:00:47	0.00%	0	(0.00%)	$0.00	(0.00%)
402.	Huawei U8825D Ascend G330D	**11**	**(0.02%)**	100.00%	11	(0.03%)	72.73%	1.36	00:00:20	0.00%	0	(0.00%)	$0.00	(0.00%)
403.	Lenovo A2109A IdeaTab A2109A	**11**	**(0.02%)**	72.73%	8	(0.02%)	36.36%	5.18	00:07:37	0.00%	0	(0.00%)	$0.00	(0.00%)
404.	Lifetab P9514	**11**	**(0.02%)**	54.55%	6	(0.01%)	100.00%	1.00	00:00:00	0.00%	0	(0.00%)	$0.00	(0.00%)
405.	Motorola MB860 Atrix	**11**	**(0.02%)**	63.64%	7	(0.02%)	45.45%	2.91	00:01:06	0.00%	0	(0.00%)	$0.00	(0.00%)
406.	Motorola MZ601 Xoom	**11**	**(0.02%)**	90.91%	10	(0.02%)	72.73%	1.64	00:00:28	0.00%	0	(0.00%)	$0.00	(0.00%)
407.	Motorola Xoom	**11**	**(0.02%)**	100.00%	11	(0.03%)	63.64%	3.09	00:00:58	0.00%	0	(0.00%)	$0.00	(0.00%)
408.	Motorola XT320 Defy Mini	**11**	**(0.02%)**	72.73%	8	(0.02%)	72.73%	1.27	00:00:31	0.00%	0	(0.00%)	$0.00	(0.00%)
409.	Nokia E63	**11**	**(0.02%)**	100.00%	11	(0.03%)	54.55%	2.18	00:02:47	0.00%	0	(0.00%)	$0.00	(0.00%)
410.	Nokia X3-02	**11**	**(0.02%)**	100.00%	11	(0.03%)	63.64%	4.00	00:03:57	0.00%	0	(0.00%)	$0.00	(0.00%)
411.	Sagem my721X	**11**	**(0.02%)**	100.00%	11	(0.03%)	63.64%	1.64	00:00:54	0.00%	0	(0.00%)	$0.00	(0.00%)
412.	Samsung GT-I9080 Galaxy Grand	**11**	**(0.02%)**	63.64%	7	(0.02%)	54.55%	11.91	00:08:49	0.00%	0	(0.00%)	$0.00	(0.00%)
413.	Samsung GT-i5500 Galaxy Europa	**11**	**(0.02%)**	63.64%	7	(0.02%)	54.55%	3.73	00:11:08	0.00%	0	(0.00%)	$0.00	(0.00%)
414.	Samsung GT-S5282 Galaxy Star	**11**	**(0.02%)**	90.91%	10	(0.02%)	72.73%	1.73	00:00:48	0.00%	0	(0.00%)	$0.00	(0.00%)
415.	Samsung GT-S5367 Samsung Galaxy Y TV	**11**	**(0.02%)**	90.91%	10	(0.02%)	45.45%	2.91	00:01:04	0.00%	0	(0.00%)	$0.00	(0.00%)
416.	Samsung GT-S7582 Galaxy S Duos 2	**11**	**(0.02%)**	54.55%	6	(0.01%)	72.73%	1.82	00:00:24	0.00%	0	(0.00%)	$0.00	(0.00%)
417.	Samsung SCH-R740C Galaxy Discover	**11**	**(0.02%)**	63.64%	7	(0.02%)	63.64%	1.45	00:01:25	0.00%	0	(0.00%)	$0.00	(0.00%)
418.	Samsung SGH-i677 Focus Flash	**11**	**(0.02%)**	45.45%	5	(0.01%)	18.18%	3.27	00:02:58	0.00%	0	(0.00%)	$0.00	(0.00%)
419.	Samsung SM-G730A Galaxy S III Mini	**11**	**(0.02%)**	27.27%	3	(0.01%)	36.36%	2.27	00:01:13	0.00%	0	(0.00%)	$0.00	(0.00%)
420.	Sony SGP311 Xperia Tablet Z	**11**	**(0.02%)**	90.91%	10	(0.02%)	45.45%	2.82	00:00:41	0.00%	0	(0.00%)	$0.00	(0.00%)
421.	SonyEricsson ST18i Urushi	**11**	**(0.02%)**	100.00%	11	(0.03%)	100.00%	1.00	00:00:00	0.00%	0	(0.00%)	$0.00	(0.00%)
422.	Acer A501 Picasso	**10**	**(0.02%)**	40.00%	4	(0.01%)	20.00%	18.30	00:15:33	0.00%	0	(0.00%)	$0.00	(0.00%)
423.	Amazon KFSOW Kindle Fire HD 7 3rd Gen	**10**	**(0.02%)**	70.00%	7	(0.02%)	80.00%	1.20	00:00:01	0.00%	0	(0.00%)	$0.00	(0.00%)
424.	BlackBerry KBD	**10**	**(0.02%)**	60.00%	6	(0.01%)	70.00%	1.30	00:00:06	0.00%	0	(0.00%)	$0.00	(0.00%)
425.	Huawei MT1-U06 Ascend Mate	**10**	**(0.02%)**	60.00%	6	(0.01%)	50.00%	7.20	00:06:46	0.00%	0	(0.00%)	$0.00	(0.00%)
426.	Huawei T8951 Ascend G510	**10**	**(0.02%)**	90.00%	9	(0.02%)	70.00%	3.50	00:02:40	0.00%	0	(0.00%)	$0.00	(0.00%)
427.	Lenovo A2107A-F IdeaTab A2107A-F	**10**	**(0.02%)**	40.00%	4	(0.01%)	50.00%	47.70	00:23:54	0.00%	0	(0.00%)	$0.00	(0.00%)
428.	LG D805 G2	**10**	**(0.02%)**	80.00%	8	(0.02%)	50.00%	3.40	00:01:43	0.00%	0	(0.00%)	$0.00	(0.00%)

429.	LG E455 Optimus L5 II Dual	**10**	**(0.02%)**	60.00%	6	(0.01%)	80.00%	8.40	00:05:11	0.00%	0	(0.00%)	$0.00	(0.00%)
430.	LG E610v Optimus L5	**10**	**(0.02%)**	80.00%	8	(0.02%)	100.00%	1.00	00:00:00	0.00%	0	(0.00%)	$0.00	(0.00%)
431.	LG E975 Optimus G	**10**	**(0.02%)**	80.00%	8	(0.02%)	70.00%	2.00	00:00:28	0.00%	0	(0.00%)	$0.00	(0.00%)
432.	LG VS840 4G Lucid	**10**	**(0.02%)**	60.00%	6	(0.01%)	50.00%	3.20	00:03:55	0.00%	0	(0.00%)	$0.00	(0.00%)
433.	Motorola MB525 DEFY	**10**	**(0.02%)**	80.00%	8	(0.02%)	90.00%	1.20	00:00:06	0.00%	0	(0.00%)	$0.00	(0.00%)
434.	Nokia 2700 classic	**10**	**(0.02%)**	90.00%	9	(0.02%)	90.00%	1.10	00:00:12	0.00%	0	(0.00%)	$0.00	(0.00%)
435.	Nokia 309 Asha 309	**10**	**(0.02%)**	90.00%	9	(0.02%)	90.00%	1.10	00:00:03	0.00%	0	(0.00%)	$0.00	(0.00%)
436.	Nokia E72	**10**	**(0.02%)**	100.00%	10	(0.02%)	50.00%	1.60	00:00:51	0.00%	0	(0.00%)	$0.00	(0.00%)
437.	Samsung GT-B5330 Galaxy Chat	**10**	**(0.02%)**	80.00%	8	(0.02%)	90.00%	1.30	00:00:35	0.00%	0	(0.00%)	$0.00	(0.00%)
438.	Samsung GT-C3312 Rex 60 Duos	**10**	**(0.02%)**	100.00%	10	(0.02%)	100.00%	1.00	00:00:00	0.00%	0	(0.00%)	$0.00	(0.00%)
439.	Samsung GT-I8730 Galaxy Express	**10**	**(0.02%)**	90.00%	9	(0.02%)	60.00%	1.60	00:00:23	0.00%	0	(0.00%)	$0.00	(0.00%)
440.	Samsung GT-I8750 ATIV Odyssey	**10**	**(0.02%)**	70.00%	7	(0.02%)	50.00%	3.50	00:01:27	0.00%	0	(0.00%)	$0.00	(0.00%)
441.	Samsung GT-I9295 Galaxy S4 Active	**10**	**(0.02%)**	100.00%	10	(0.02%)	80.00%	2.40	00:00:40	0.00%	0	(0.00%)	$0.00	(0.00%)
442.	Samsung GT-I9506 Galaxy S IV	**10**	**(0.02%)**	90.00%	9	(0.02%)	70.00%	2.40	00:00:27	0.00%	0	(0.00%)	$0.00	(0.00%)
443.	Samsung SCH-I200 Galaxy Stellar 4G	**10**	**(0.02%)**	100.00%	10	(0.02%)	90.00%	1.10	00:00:16	0.00%	0	(0.00%)	$0.00	(0.00%)
444.	Samsung SGH-I747 Galaxy S III	**10**	**(0.02%)**	100.00%	10	(0.02%)	80.00%	1.30	00:00:20	0.00%	0	(0.00%)	$0.00	(0.00%)
445.	Samsung SPH-M830 Galaxy Rush	**10**	**(0.02%)**	80.00%	8	(0.02%)	30.00%	4.20	00:03:02	0.00%	0	(0.00%)	$0.00	(0.00%)
446.	Sony C2305 Xperia C	**10**	**(0.02%)**	90.00%	9	(0.02%)	80.00%	3.10	00:01:59	0.00%	0	(0.00%)	$0.00	(0.00%)
447.	Sony SGP321 Xperia Tablet Z LTE	**10**	**(0.02%)**	80.00%	8	(0.02%)	30.00%	6.20	00:06:15	0.00%	0	(0.00%)	$0.00	(0.00%)
448.	SonyEricsson SK17a Xperia Mini Pro	**10**	**(0.02%)**	90.00%	9	(0.02%)	50.00%	3.10	00:02:31	0.00%	0	(0.00%)	$0.00	(0.00%)
449.	T-Mobile myTouch4G	**10**	**(0.02%)**	70.00%	7	(0.02%)	80.00%	5.70	00:17:22	0.00%	0	(0.00%)	$0.00	(0.00%)
450.	Asus A68 PadFone 2	**9**	**(0.01%)**	88.89%	8	(0.02%)	66.67%	3.78	00:00:47	0.00%	0	(0.00%)	$0.00	(0.00%)
451.	DoCoMo SO-03D Xperia acro HD	**9**	**(0.01%)**	22.22%	2	(0.00%)	22.22%	8.11	00:04:54	0.00%	0	(0.00%)	$0.00	(0.00%)
452.	HTC One mini	**9**	**(0.01%)**	88.89%	8	(0.02%)	88.89%	1.11	00:00:05	0.00%	0	(0.00%)	$0.00	(0.00%)
453.	Lenovo A3000-F IdeaTab A3000-F	**9**	**(0.01%)**	22.22%	2	(0.00%)	66.67%	1.89	00:00:58	0.00%	0	(0.00%)	$0.00	(0.00%)
454.	LG E410g Optimus L1X	**9**	**(0.01%)**	100.00%	9	(0.02%)	88.89%	1.11	00:00:04	0.00%	0	(0.00%)	$0.00	(0.00%)
455.	LG E430 Optimus L3 II	**9**	**(0.01%)**	88.89%	8	(0.02%)	77.78%	2.00	00:00:31	0.00%	0	(0.00%)	$0.00	(0.00%)

456.	LG V500 G Pad 8.3	**9**	**(0.01%)**	44.44%	4	(0.01%)	77.78%	4.33	00:01:22	0.00%	0	(0.00%)	$0.00 (0.00%)
457.	Samsung GT-I8160L Galaxy Ace 2	**9**	**(0.01%)**	100.00%	9	(0.02%)	88.89%	1.11	00:00:01	0.00%	0	(0.00%)	$0.00 (0.00%)
458.	Samsung GT-S6010 Galaxy Music	**9**	**(0.01%)**	88.89%	8	(0.02%)	66.67%	2.11	00:01:06	0.00%	0	(0.00%)	$0.00 (0.00%)
459.	Samsung SCH-S738C Galaxy Centura	**9**	**(0.01%)**	88.89%	8	(0.02%)	88.89%	1.11	00:01:22	0.00%	0	(0.00%)	$0.00 (0.00%)
460.	Samsung SM-T110 Galaxy Tab 3 Lite	**9**	**(0.01%)**	33.33%	3	(0.01%)	44.44%	6.44	00:06:04	0.00%	0	(0.00%)	$0.00 (0.00%)
461.	Sony C6506 Xperia ZL	**9**	**(0.01%)**	66.67%	6	(0.01%)	77.78%	1.78	00:00:32	0.00%	0	(0.00%)	$0.00 (0.00%)
462.	Sony ST26a Xperia J	**9**	**(0.01%)**	100.00%	9	(0.02%)	100.00%	1.00	00:00:00	0.00%	0	(0.00%)	$0.00 (0.00%)
463.	SonyEricsson MT27i Xperia Sola	**9**	**(0.01%)**	100.00%	9	(0.02%)	55.56%	3.22	00:02:17	0.00%	0	(0.00%)	$0.00 (0.00%)
464.	Vodafone 875 Smart Mini	**9**	**(0.01%)**	66.67%	6	(0.01%)	33.33%	2.44	00:01:33	0.00%	0	(0.00%)	$0.00 (0.00%)
465.	ALCATEL ONE TOUCH 6012X	**8**	**(0.01%)**	62.50%	5	(0.01%)	50.00%	2.38	00:00:50	0.00%	0	(0.00%)	$0.00 (0.00%)
466.	Alcatel OT-918A One Touch 918A	**8**	**(0.01%)**	75.00%	6	(0.01%)	37.50%	3.50	00:03:38	0.00%	0	(0.00%)	$0.00 (0.00%)
467.	Asus ME302C MeMO Pad ME302C	**8**	**(0.01%)**	62.50%	5	(0.01%)	87.50%	2.50	00:00:10	0.00%	0	(0.00%)	$0.00 (0.00%)
468.	Barnes and Noble BNTV250A NOOK Tablet	**8**	**(0.01%)**	100.00%	8	(0.02%)	50.00%	5.00	00:03:46	0.00%	0	(0.00%)	$0.00 (0.00%)
469.	DoCoMo N-02E One Piece Limited Edition	**8**	**(0.01%)**	12.50%	1	(0.00%)	0.00%	9.38	00:13:24	0.00%	0	(0.00%)	$0.00 (0.00%)
470.	HTC A810e ChaCha	**8**	**(0.01%)**	75.00%	6	(0.01%)	50.00%	6.00	00:05:16	0.00%	0	(0.00%)	$0.00 (0.00%)
471.	HTC Amaze 4G	**8**	**(0.01%)**	37.50%	3	(0.01%)	62.50%	2.38	00:01:48	0.00%	0	(0.00%)	$0.00 (0.00%)
472.	HTC X515C EVC 3D	**8**	**(0.01%)**	62.50%	5	(0.01%)	75.00%	1.62	00:00:18	0.00%	0	(0.00%)	$0.00 (0.00%)
473.	Huawei Y210-0100 Ascend Y210	**8**	**(0.01%)**	75.00%	6	(0.01%)	75.00%	1.75	00:00:55	0.00%	0	(0.00%)	$0.00 (0.00%)
474.	Kyocera C5170 Hydro C5170	**8**	**(0.01%)**	100.00%	8	(0.02%)	62.50%	2.12	00:00:26	0.00%	0	(0.00%)	$0.00 (0.00%)
475.	LG E405F Optimus L3	**8**	**(0.01%)**	62.50%	5	(0.01%)	25.00%	5.75	00:11:07	0.00%	0	(0.00%)	$0.00 (0.00%)
476.	LG E615 Optimus L5 Dual	**8**	**(0.01%)**	62.50%	5	(0.01%)	37.50%	4.88	00:04:34	0.00%	0	(0.00%)	$0.00 (0.00%)
477.	LG LG-C195	**8**	**(0.01%)**	75.00%	6	(0.01%)	62.50%	1.50	00:00:59	0.00%	0	(0.00%)	$0.00 (0.00%)
478.	LG LG-T375	**8**	**(0.01%)**	100.00%	8	(0.02%)	87.50%	1.38	00:00:14	0.00%	0	(0.00%)	$0.00 (0.00%)
479.	LG LS980 G2	**8**	**(0.01%)**	100.00%	8	(0.02%)	75.00%	2.75	00:01:07	0.00%	0	(0.00%)	$0.00 (0.00%)
480.	LG P700 Optimus L7	**8**	**(0.01%)**	100.00%	8	(0.02%)	87.50%	1.12	00:00:07	0.00%	0	(0.00%)	$0.00 (0.00%)
481.	LG P970h Optimus Black	**8**	**(0.01%)**	62.50%	5	(0.01%)	62.50%	2.12	00:01:10	0.00%	0	(0.00%)	$0.00 (0.00%)
482.	LG VS920 4G Revolution 2	**8**	**(0.01%)**	37.50%	3	(0.01%)	50.00%	2.88	00:00:34	0.00%	0	(0.00%)	$0.00 (0.00%)
483.	Nokia 310 Asha 310	**8**	**(0.01%)**	75.00%	6	(0.01%)	87.50%	1.25	00:00:11	0.00%	0	(0.00%)	$0.00 (0.00%)

484.	Nokia Asha 210.2 Asha 210	**8**	**(0.01%)**	100.00%	8	(0.02%)	37.50%	3.62	00:01:59	0.00%	0	(0.00%)	$0.00	(0.00%)
485.	Nokia C2-06	**8**	**(0.01%)**	100.00%	8	(0.02%)	75.00%	1.50	00:00:16	0.00%	0	(0.00%)	$0.00	(0.00%)
486.	Nokia C5-03 C5	**8**	**(0.01%)**	100.00%	8	(0.02%)	100.00%	1.00	00:00:00	0.00%	0	(0.00%)	$0.00	(0.00%)
487.	Nokia N900	**8**	**(0.01%)**	87.50%	7	(0.02%)	87.50%	3.00	00:02:48	0.00%	0	(0.00%)	$0.00	(0.00%)
488.	Pantech ADR8995 4G Breakout	**8**	**(0.01%)**	100.00%	8	(0.02%)	87.50%	1.50	00:00:06	0.00%	0	(0.00%)	$0.00	(0.00%)
489.	Pantech IM-A850K Vega R3	**8**	**(0.01%)**	50.00%	4	(0.01%)	87.50%	1.50	00:00:14	0.00%	0	(0.00%)	$0.00	(0.00%)
490.	RIM PlayBook	**8**	**(0.01%)**	50.00%	4	(0.01%)	62.50%	8.88	00:04:47	0.00%	0	(0.00%)	$0.00	(0.00%)
491.	Samsung GT-P5220 Galaxy Tab 3 10.1 LTE	**8**	**(0.01%)**	100.00%	8	(0.02%)	62.50%	2.88	00:00:39	0.00%	0	(0.00%)	$0.00	(0.00%)
492.	Samsung GT-S5253	**8**	**(0.01%)**	100.00%	8	(0.02%)	50.00%	1.88	00:03:07	0.00%	0	(0.00%)	$0.00	(0.00%)
493.	Samsung GT-S5690 Xcover	**8**	**(0.01%)**	75.00%	6	(0.01%)	75.00%	2.75	00:01:32	0.00%	0	(0.00%)	$0.00	(0.00%)
494.	Samsung GT-S5690L Xcover	**8**	**(0.01%)**	50.00%	4	(0.01%)	0.00%	5.75	00:11:13	0.00%	0	(0.00%)	$0.00	(0.00%)
495.	Samsung GT-S6313T	**8**	**(0.01%)**	62.50%	5	(0.01%)	37.50%	2.00	00:01:05	0.00%	0	(0.00%)	$0.00	(0.00%)
496.	Samsung GT-S6500L Galaxy Mini 2	**8**	**(0.01%)**	62.50%	5	(0.01%)	50.00%	1.75	00:00:15	0.00%	0	(0.00%)	$0.00	(0.00%)
497.	Samsung GT-S7560 Galaxy Ace II x	**8**	**(0.01%)**	100.00%	8	(0.02%)	37.50%	2.12	00:00:54	0.00%	0	(0.00%)	$0.00	(0.00%)
498.	Samsung SGH-I547 Galaxy Rugby Pro	**8**	**(0.01%)**	100.00%	8	(0.02%)	62.50%	2.38	00:01:03	0.00%	0	(0.00%)	$0.00	(0.00%)
499.	Samsung SGH-I577 Exhilarate	**8**	**(0.01%)**	87.50%	7	(0.02%)	87.50%	1.12	00:00:02	0.00%	0	(0.00%)	$0.00	(0.00%)
500.	Samsung SHV-E250K Galaxy Note II	**8**	**(0.01%)**	100.00%	8	(0.02%)	62.50%	2.00	00:00:38	0.00%	0	(0.00%)	$0.00	(0.00%)
501.	Samsung SPH-D700 Epic 4G	**8**	**(0.01%)**	87.50%	7	(0.02%)	75.00%	1.38	00:00:09	0.00%	0	(0.00%)	$0.00	(0.00%)
502.	Sony C1604 Xperia E Dual	**8**	**(0.01%)**	75.00%	6	(0.01%)	75.00%	2.88	00:05:29	0.00%	0	(0.00%)	$0.00	(0.00%)
503.	SonyEricsson MT15i Xperia Neo	**8**	**(0.01%)**	100.00%	8	(0.02%)	100.00%	1.00	00:00:00	0.00%	0	(0.00%)	$0.00	(0.00%)
504.	SonyEricsson SK17i Xperia Mini Pro	**8**	**(0.01%)**	100.00%	8	(0.02%)	50.00%	1.88	00:01:52	0.00%	0	(0.00%)	$0.00	(0.00%)
505.	SonyEricsson X10i Xperia X10	**8**	**(0.01%)**	100.00%	8	(0.02%)	75.00%	3.00	00:05:29	0.00%	0	(0.00%)	$0.00	(0.00%)
506.	TWM Fantastic T3	**8**	**(0.01%)**	25.00%	2	(0.00%)	50.00%	1.75	00:02:01	0.00%	0	(0.00%)	$0.00	(0.00%)
507.	Xiaomi MI-2	**8**	**(0.01%)**	75.00%	6	(0.01%)	50.00%	2.75	00:01:00	0.00%	0	(0.00%)	$0.00	(0.00%)
508.	Barnes and Noble BNTV250 NOOK Tablet	**7**	**(0.01%)**	85.71%	6	(0.01%)	57.14%	2.00	00:00:20	0.00%	0	(0.00%)	$0.00	(0.00%)
509.	Google Nexus S	**7**	**(0.01%)**	71.43%	5	(0.01%)	71.43%	1.71	00:02:24	0.00%	0	(0.00%)	$0.00	(0.00%)
510.	HTC APC715CKT EVO Design 4G	**7**	**(0.01%)**	100.00%	7	(0.02%)	85.71%	1.57	00:00:19	0.00%	0	(0.00%)	$0.00	(0.00%)
511.	HTC HTC6435LVW Droid DNA	**7**	**(0.01%)**	100.00%	7	(0.02%)	71.43%	1.71	00:01:05	0.00%	0	(0.00%)	$0.00	(0.00%)

512.	HTC ISW11HT HTC EVO WiMAX	**7**	**(0.01%)**	28.57%	2	(0.00%)	14.29%	27.71	00:07:41	0.00%	0	(0.00%)	$0.00	(0.00%)
513.	HTC One V	**7**	**(0.01%)**	100.00%	7	(0.02%)	71.43%	15.14	00:09:55	0.00%	0	(0.00%)	$0.00	(0.00%)
514.	HTC S510e Desire S	**7**	**(0.01%)**	57.14%	4	(0.01%)	42.86%	3.14	00:06:09	0.00%	0	(0.00%)	$0.00	(0.00%)
515.	HTC X315e Sensation XL with Beats Audio	**7**	**(0.01%)**	57.14%	4	(0.01%)	42.86%	4.43	00:02:50	0.00%	0	(0.00%)	$0.00	(0.00%)
516.	Lenovo A1000-T IdeaTab A1000-T	**7**	**(0.01%)**	85.71%	6	(0.01%)	85.71%	1.14	00:00:08	0.00%	0	(0.00%)	$0.00	(0.00%)
517.	LG E425g Optimus L3 II	**7**	**(0.01%)**	100.00%	7	(0.02%)	42.86%	2.00	00:03:29	0.00%	0	(0.00%)	$0.00	(0.00%)
518.	LG E430f Optimus L3 II	**7**	**(0.01%)**	71.43%	5	(0.01%)	28.57%	2.43	00:03:29	0.00%	0	(0.00%)	$0.00	(0.00%)
519.	LG E440g Optimus L4 II	**7**	**(0.01%)**	71.43%	5	(0.01%)	14.29%	5.14	00:02:54	0.00%	0	(0.00%)	$0.00	(0.00%)
520.	LG E980 Optimus G Pro	**7**	**(0.01%)**	100.00%	7	(0.02%)	71.43%	2.00	00:00:16	0.00%	0	(0.00%)	$0.00	(0.00%)
521.	LG F240L Optimus G Pro	**7**	**(0.01%)**	100.00%	7	(0.02%)	57.14%	4.86	00:00:58	0.00%	0	(0.00%)	$0.00	(0.00%)
522.	LG P705 Optimus L7	**7**	**(0.01%)**	57.14%	4	(0.01%)	57.14%	2.71	00:06:01	0.00%	0	(0.00%)	$0.00	(0.00%)
523.	LogicPD Zoom2 Barnes & Noble Nook Color	**7**	**(0.01%)**	85.71%	6	(0.01%)	100.00%	1.00	00:00:00	0.00%	0	(0.00%)	$0.00	(0.00%)
524.	Medion LIFETAB S9512	**7**	**(0.01%)**	100.00%	7	(0.02%)	100.00%	1.00	00:00:00	0.00%	0	(0.00%)	$0.00	(0.00%)
525.	Micromax A110Q Canvas 2 Plus	**7**	**(0.01%)**	85.71%	6	(0.01%)	85.71%	1.14	00:00:05	0.00%	0	(0.00%)	$0.00	(0.00%)
526.	Motorola DroidX	**7**	**(0.01%)**	100.00%	7	(0.02%)	71.43%	1.57	00:00:12	0.00%	0	(0.00%)	$0.00	(0.00%)
527.	Motorola MB632 Pro+	**7**	**(0.01%)**	42.86%	3	(0.01%)	28.57%	4.43	00:04:55	0.00%	0	(0.00%)	$0.00	(0.00%)
528.	Motorola XT1030 Droid Mini	**7**	**(0.01%)**	57.14%	4	(0.01%)	57.14%	2.43	00:00:24	0.00%	0	(0.00%)	$0.00	(0.00%)
529.	Motorola XT1032 DVX	**7**	**(0.01%)**	85.71%	6	(0.01%)	57.14%	2.86	00:02:04	0.00%	0	(0.00%)	$0.00	(0.00%)
530.	Motorola XT316 Dominoq	**7**	**(0.01%)**	71.43%	5	(0.01%)	28.57%	5.14	00:05:07	0.00%	0	(0.00%)	$0.00	(0.00%)
531.	Motorola XT389 Motoluxe XT389	**7**	**(0.01%)**	71.43%	5	(0.01%)	28.57%	10.43	00:09:16	0.00%	0	(0.00%)	$0.00	(0.00%)
532.	Motorola XT621 Primus	**7**	**(0.01%)**	85.71%	6	(0.01%)	42.86%	3.00	00:02:12	0.00%	0	(0.00%)	$0.00	(0.00%)
533.	Motorola XT626	**7**	**(0.01%)**	100.00%	7	(0.02%)	42.86%	6.14	00:02:24	0.00%	0	(0.00%)	$0.00	(0.00%)
534.	Motorola XT890	**7**	**(0.01%)**	57.14%	4	(0.01%)	57.14%	3.14	00:01:24	0.00%	0	(0.00%)	$0.00	(0.00%)
535.	Nokia 5530 XpressMusic	**7**	**(0.01%)**	85.71%	6	(0.01%)	85.71%	2.00	00:03:44	0.00%	0	(0.00%)	$0.00	(0.00%)
536.	Nokia Asha 210.5 Asha 210	**7**	**(0.01%)**	85.71%	6	(0.01%)	28.57%	7.71	00:11:38	0.00%	0	(0.00%)	$0.00	(0.00%)
537.	Nokia C1-01	**7**	**(0.01%)**	100.00%	7	(0.02%)	100.00%	1.00	00:00:00	0.00%	0	(0.00%)	$0.00	(0.00%)
538.	Nokia C2-05	**7**	**(0.01%)**	100.00%	7	(0.02%)	42.86%	1.71	00:01:43	0.00%	0	(0.00%)	$0.00	(0.00%)
539.	Nokia C7-00 Astound	**7**	**(0.01%)**	100.00%	7	(0.02%)	85.71%	1.14	00:00:29	0.00%	0	(0.00%)	$0.00	(0.00%)
540.	Nokia Lumia 521	**7**	**(0.01%)**	100.00%	7	(0.02%)	57.14%	3.43	00:01:07	0.00%	0	(0.00%)	$0.00	(0.00%)
541.	Nokia X2-00	**7**	**(0.01%)**	100.00%	7	(0.02%)	42.86%	2.71	00:01:02	0.00%	0	(0.00%)	$0.00	(0.00%)
542.	QMobile A10 Noi A10	**7**	**(0.01%)**	85.71%	6	(0.01%)	71.43%	1.86	00:01:11	0.00%	0	(0.00%)	$0.00	(0.00%)
[illegible]	Samsung GT-	[illegible]	[illegible]	[illegible]	[illegible]	[illegible]	[illegible]	[illegible]	[illegible]	[illegible]	[illegible]	[illegible]	[illegible]	[illegible]

543.	Samsung GT-C3312	**7** **(0.01%)**	85.71%	6 (0.01%)	57.14%	3.14	00:03:44	0.00%	0 (0.00%)	$0.00 (0.00%)
544.	Samsung GT-E2250 Utica	**7** **(0.01%)**	100.00%	7 (0.02%)	57.14%	1.86	00:00:43	0.00%	0 (0.00%)	$0.00 (0.00%)
545.	Samsung GT-I8260 Galaxy Core	**7** **(0.01%)**	100.00%	7 (0.02%)	85.71%	1.57	00:00:12	0.00%	0 (0.00%)	$0.00 (0.00%)
546.	Samsung GT-i8350 Omnia W	**7** **(0.01%)**	57.14%	4 (0.01%)	85.71%	1.86	00:00:23	0.00%	0 (0.00%)	$0.00 (0.00%)
547.	Samsung GT-I8550L Galaxy Grand Quattro	**7** **(0.01%)**	57.14%	4 (0.01%)	100.00%	1.00	00:00:00	0.00%	0 (0.00%)	$0.00 (0.00%)
548.	Samsung GT-I9105 Galaxy SII Plus	**7** **(0.01%)**	100.00%	7 (0.02%)	85.71%	1.14	00:00:02	0.00%	0 (0.00%)	$0.00 (0.00%)
549.	Samsung GT-N8020 Galaxy Note 10.1 LTE	**7** **(0.01%)**	100.00%	7 (0.02%)	57.14%	5.14	00:03:29	0.00%	0 (0.00%)	$0.00 (0.00%)
550.	Samsung GT-P6200L GALAXY Tab 7.0 Plus	**7** **(0.01%)**	85.71%	6 (0.01%)	42.86%	4.14	00:01:58	0.00%	0 (0.00%)	$0.00 (0.00%)
551.	Samsung GT-S3802 Rex 70 Duos	**7** **(0.01%)**	85.71%	6 (0.01%)	100.00%	1.00	00:00:00	0.00%	0 (0.00%)	$0.00 (0.00%)
552.	Samsung GT-S7262 Galaxy Star S7262	**7** **(0.01%)**	100.00%	7 (0.02%)	85.71%	1.14	00:04:20	0.00%	0 (0.00%)	$0.00 (0.00%)
553.	Samsung GT-S7580 Trend Plus	**7** **(0.01%)**	42.86%	3 (0.01%)	57.14%	1.86	00:00:22	0.00%	0 (0.00%)	$0.00 (0.00%)
554.	Samsung SGH-T769 Galaxy S Blaze 4G	**7** **(0.01%)**	85.71%	6 (0.01%)	71.43%	2.71	00:00:44	0.00%	0 (0.00%)	$0.00 (0.00%)
555.	Samsung SGH-T999L Galaxy SIII LTE	**7** **(0.01%)**	85.71%	6 (0.01%)	71.43%	1.57	00:00:21	0.00%	0 (0.00%)	$0.00 (0.00%)
556.	Samsung SHW-M440S Galaxy SIII	**7** **(0.01%)**	28.57%	2 (0.00%)	14.29%	4.86	00:08:25	0.00%	0 (0.00%)	$0.00 (0.00%)
557.	Sony C6502 Xperia ZL	**7** **(0.01%)**	100.00%	7 (0.02%)	71.43%	1.57	00:01:05	0.00%	0 (0.00%)	$0.00 (0.00%)
558.	Sony C6906 Xperia Z1	**7** **(0.01%)**	71.43%	5 (0.01%)	42.86%	6.43	00:03:57	0.00%	0 (0.00%)	$0.00 (0.00%)
559.	SonyEricsson R800a Xperia PLAY	**7** **(0.01%)**	100.00%	7 (0.02%)	85.71%	1.43	00:00:40	0.00%	0 (0.00%)	$0.00 (0.00%)
560.	Alcatel OT-5035A One Touch X'Pop 5035A	**6** **(0.01%)**	50.00%	3 (0.01%)	50.00%	5.00	00:04:16	0.00%	0 (0.00%)	$0.00 (0.00%)
561.	Asus ME302C MeMO Pad FHD 10	**6** **(0.01%)**	50.00%	3 (0.01%)	83.33%	1.33	00:00:24	0.00%	0 (0.00%)	$0.00 (0.00%)
562.	bq Aquarius 5	**6** **(0.01%)**	100.00%	6 (0.01%)	83.33%	2.00	00:00:46	0.00%	0 (0.00%)	$0.00 (0.00%)
563.	DoCoMo SC-05D Galaxy Note	**6** **(0.01%)**	66.67%	4 (0.01%)	100.00%	1.00	00:00:00	0.00%	0 (0.00%)	$0.00 (0.00%)
564.	Ginovo MID	**6** **(0.01%)**	83.33%	5 (0.01%)	33.33%	3.50	00:02:35	0.00%	0 (0.00%)	$0.00 (0.00%)
565.	HTC S510b Rhyme	**6** **(0.01%)**	66.67%	4 (0.01%)	16.67%	7.50	00:15:17	0.00%	0 (0.00%)	$0.00 (0.00%)
566.	HTC X515 EVO 3D	**6** **(0.01%)**	100.00%	6 (0.01%)	83.33%	1.33	00:00:04	0.00%	0 (0.00%)	$0.00 (0.00%)
567.	LG L38C Optimus Dynamic	**6** **(0.01%)**	100.00%	6 (0.01%)	50.00%	2.83	00:00:49	0.00%	0 (0.00%)	$0.00 (0.00%)
568.	LG T515 T515 Cookie Duo	**6** **(0.01%)**	100.00%	6 (0.01%)	66.67%	2.00	00:02:18	0.00%	0 (0.00%)	$0.00 (0.00%)
569.	LG VS980 4G [illegible]	**6** **(0.01%)**	83.33%	5 (0.01%)	83.33%	1.33	00:00:01	0.00%	0 (0.00%)	$0.00 (0.00%)

	Optimus G2 4G	[illegible]	[illegible]	[illegible]	[illegible]	[illegible]	[illegible]	[illegible]	[illegible]	[illegible]	[illegible]	[illegible]	[illegible]
570.	Medion LifeTab P9516	**6**	**(0.01%)**	100.00%	6	(0.01%)	66.67%	2.67	00:00:43	0.00%	0	(0.00%)	$0.00 (0.00%)
571.	Motorola Droid X2	**6**	**(0.01%)**	100.00%	6	(0.01%)	83.33%	1.17	00:00:07	0.00%	0	(0.00%)	$0.00 (0.00%)
572.	Nokia Asha 503	**6**	**(0.01%)**	100.00%	6	(0.01%)	50.00%	2.33	00:00:55	0.00%	0	(0.00%)	$0.00 (0.00%)
573.	Nokia C5-00 5MF C5	**6**	**(0.01%)**	100.00%	6	(0.01%)	66.67%	1.33	00:01:07	0.00%	0	(0.00%)	$0.00 (0.00%)
574.	Nokia C6-00	**6**	**(0.01%)**	100.00%	6	(0.01%)	50.00%	7.00	00:05:18	0.00%	0	(0.00%)	$0.00 (0.00%)
575.	Nokia E71	**6**	**(0.01%)**	100.00%	6	(0.01%)	50.00%	2.17	00:02:58	0.00%	0	(0.00%)	$0.00 (0.00%)
576.	Nokia Lumia 822	**6**	**(0.01%)**	83.33%	5	(0.01%)	83.33%	2.67	00:00:23	0.00%	0	(0.00%)	$0.00 (0.00%)
577.	Samsung GT-B5330L Galaxy Chat	**6**	**(0.01%)**	66.67%	4	(0.01%)	66.67%	1.50	00:04:28	0.00%	0	(0.00%)	$0.00 (0.00%)
578.	Samsung GT-i5500 Galaxy 5	**6**	**(0.01%)**	83.33%	5	(0.01%)	50.00%	2.33	00:05:56	0.00%	0	(0.00%)	$0.00 (0.00%)
579.	Samsung GT-N8005 Galaxy Note 10.1	**6**	**(0.01%)**	50.00%	3	(0.01%)	66.67%	1.67	00:00:26	0.00%	0	(0.00%)	$0.00 (0.00%)
580.	Samsung GT-S3570 Ch@t	**6**	**(0.01%)**	100.00%	6	(0.01%)	50.00%	1.50	00:00:37	0.00%	0	(0.00%)	$0.00 (0.00%)
581.	Samsung GT-S5301B Galaxy Pocket Plus	**6**	**(0.01%)**	83.33%	5	(0.01%)	66.67%	3.17	00:05:52	0.00%	0	(0.00%)	$0.00 (0.00%)
582.	Samsung GT-S5312 Galaxy Pocket Neo	**6**	**(0.01%)**	83.33%	5	(0.01%)	83.33%	1.17	00:02:34	0.00%	0	(0.00%)	$0.00 (0.00%)
583.	Samsung GT-S5830B Galaxy Ace	**6**	**(0.01%)**	83.33%	5	(0.01%)	33.33%	3.17	00:05:02	0.00%	0	(0.00%)	$0.00 (0.00%)
584.	Samsung GT-S7270 Galaxy Ace 3	**6**	**(0.01%)**	83.33%	5	(0.01%)	50.00%	2.50	00:01:14	0.00%	0	(0.00%)	$0.00 (0.00%)
585.	Samsung GT-S7275R Galaxy Ace 3	**6**	**(0.01%)**	100.00%	6	(0.01%)	100.00%	1.00	00:00:00	0.00%	0	(0.00%)	$0.00 (0.00%)
586.	Samsung SCH-i405 Stratosphere	**6**	**(0.01%)**	83.33%	5	(0.01%)	83.33%	1.33	00:00:04	0.00%	0	(0.00%)	$0.00 (0.00%)
587.	Samsung SCH-I500 Fascinate	**6**	**(0.01%)**	50.00%	3	(0.01%)	16.67%	4.00	00:03:03	0.00%	0	(0.00%)	$0.00 (0.00%)
588.	Samsung SCH-I915 Galaxy Tab 2 10.1	**6**	**(0.01%)**	100.00%	6	(0.01%)	66.67%	2.67	00:01:41	0.00%	0	(0.00%)	$0.00 (0.00%)
589.	Samsung SCH-R830 Galaxy Axiom	**6**	**(0.01%)**	83.33%	5	(0.01%)	100.00%	1.00	00:00:00	0.00%	0	(0.00%)	$0.00 (0.00%)
590.	Samsung SGH-I497 Galaxy Tab 2 10.1	**6**	**(0.01%)**	100.00%	6	(0.01%)	66.67%	1.83	00:00:18	0.00%	0	(0.00%)	$0.00 (0.00%)
591.	Samsung SGH-I897 Galaxy S Captivate	**6**	**(0.01%)**	100.00%	6	(0.01%)	83.33%	1.67	00:00:23	0.00%	0	(0.00%)	$0.00 (0.00%)
592.	Samsung SGH-i917 Omnia 7	**6**	**(0.01%)**	100.00%	6	(0.01%)	66.67%	2.00	00:00:25	0.00%	0	(0.00%)	$0.00 (0.00%)
593.	Samsung SGH-T699 Galaxy S Relay 4G	**6**	**(0.01%)**	33.33%	2	(0.00%)	100.00%	1.00	00:00:00	0.00%	0	(0.00%)	$0.00 (0.00%)
594.	Samsung SHV-E160K Galaxy Note	**6**	**(0.01%)**	100.00%	6	(0.01%)	50.00%	3.00	00:01:11	0.00%	0	(0.00%)	$0.00 (0.00%)
595.	Samsung SHV-E250L Galaxy Note II LTE	**6**	**(0.01%)**	83.33%	5	(0.01%)	50.00%	5.50	00:04:06	0.00%	0	(0.00%)	$0.00 (0.00%)

596.	Samsung SM-G900V Galaxy S5	**6** **(0.01%)**	83.33%	5 (0.01%)	83.33%	1.17	00:00:02	0.00%	0 (0.00%)	$0.00 (0.00%)
597.	Sony C1505 Xperia E	**6** **(0.01%)**	100.00%	6 (0.01%)	83.33%	1.17	00:00:02	0.00%	0 (0.00%)	$0.00 (0.00%)
598.	ZTE Skate	**6** **(0.01%)**	33.33%	2 (0.00%)	16.67%	9.33	00:07:49	0.00%	0 (0.00%)	$0.00 (0.00%)
599.	ZTE X501 Groove	**6** **(0.01%)**	83.33%	5 (0.01%)	66.67%	3.50	00:02:15	0.00%	0 (0.00%)	$0.00 (0.00%)
600.	Alcatel OT-5020D One Touch M'Pop	**5** **(0.01%)**	80.00%	4 (0.01%)	100.00%	1.00	00:00:00	0.00%	0 (0.00%)	$0.00 (0.00%)
601.	Archos 101 G9	**5** **(0.01%)**	100.00%	5 (0.01%)	80.00%	1.20	00:00:04	0.00%	0 (0.00%)	$0.00 (0.00%)
602.	BlackBerry 9720	**5** **(0.01%)**	100.00%	5 (0.01%)	60.00%	2.60	00:06:38	0.00%	0 (0.00%)	$0.00 (0.00%)
603.	HTC 8S	**5** **(0.01%)**	100.00%	5 (0.01%)	80.00%	1.20	00:00:22	0.00%	0 (0.00%)	$0.00 (0.00%)
604.	HTC Desire 500	**5** **(0.01%)**	100.00%	5 (0.01%)	60.00%	3.40	00:01:22	0.00%	0 (0.00%)	$0.00 (0.00%)
605.	HTC HTL21 J Butterfly	**5** **(0.01%)**	100.00%	5 (0.01%)	100.00%	1.00	00:00:00	0.00%	0 (0.00%)	$0.00 (0.00%)
606.	HTC PH39100 Vivid 4G	**5** **(0.01%)**	100.00%	5 (0.01%)	100.00%	1.00	00:00:00	0.00%	0 (0.00%)	$0.00 (0.00%)
607.	HTC PI86100 Titan II	**5** **(0.01%)**	40.00%	2 (0.00%)	60.00%	2.60	00:01:23	0.00%	0 (0.00%)	$0.00 (0.00%)
608.	HTC Windows Phone 8X	**5** **(0.01%)**	100.00%	5 (0.01%)	100.00%	1.00	00:00:00	0.00%	0 (0.00%)	$0.00 (0.00%)
609.	Huawei BM-SWU300 IDEOS	**5** **(0.01%)**	80.00%	4 (0.01%)	100.00%	1.00	00:00:00	0.00%	0 (0.00%)	$0.00 (0.00%)
610.	Huawei C8812 Ascend C8812	**5** **(0.01%)**	60.00%	3 (0.01%)	60.00%	2.00	00:02:03	0.00%	0 (0.00%)	$0.00 (0.00%)
611.	KDDI ISW12K Urbano Progresso	**5** **(0.01%)**	20.00%	1 (0.00%)	40.00%	3.40	00:01:17	0.00%	0 (0.00%)	$0.00 (0.00%)
612.	Lenovo A3000-H IdeaTab A3000-H	**5** **(0.01%)**	80.00%	4 (0.01%)	80.00%	1.20	00:02:46	0.00%	0 (0.00%)	$0.00 (0.00%)
613.	Lenovo P700i	**5** **(0.01%)**	60.00%	3 (0.01%)	40.00%	7.40	00:15:41	0.00%	0 (0.00%)	$0.00 (0.00%)
614.	LG LS670 Optimus S	**5** **(0.01%)**	60.00%	3 (0.01%)	80.00%	5.80	00:02:57	0.00%	0 (0.00%)	$0.00 (0.00%)
615.	LG LS860 Mach LS860	**5** **(0.01%)**	60.00%	3 (0.01%)	100.00%	1.00	00:00:00	0.00%	0 (0.00%)	$0.00 (0.00%)
616.	LG MS769 Optimus L9	**5** **(0.01%)**	80.00%	4 (0.01%)	100.00%	1.00	00:00:00	0.00%	0 (0.00%)	$0.00 (0.00%)
617.	LG MS870 Spirit 4G	**5** **(0.01%)**	100.00%	5 (0.01%)	80.00%	1.20	00:00:23	0.00%	0 (0.00%)	$0.00 (0.00%)
618.	LG P920 Optimus 3D	**5** **(0.01%)**	100.00%	5 (0.01%)	100.00%	1.00	00:00:00	0.00%	0 (0.00%)	$0.00 (0.00%)
619.	LG T395	**5** **(0.01%)**	80.00%	4 (0.01%)	40.00%	5.80	00:03:51	0.00%	0 (0.00%)	$0.00 (0.00%)
620.	Micromax A27	**5** **(0.01%)**	100.00%	5 (0.01%)	60.00%	2.20	00:00:50	0.00%	0 (0.00%)	$0.00 (0.00%)
621.	Motorola XT1060 Moto X	**5** **(0.01%)**	100.00%	5 (0.01%)	60.00%	2.40	00:00:27	0.00%	0 (0.00%)	$0.00 (0.00%)
622.	Nokia 2690	**5** **(0.01%)**	100.00%	5 (0.01%)	80.00%	1.20	00:00:03	0.00%	0 (0.00%)	$0.00 (0.00%)
623.	Nokia 5310 XpressMusic XpressMusic	**5** **(0.01%)**	100.00%	5 (0.01%)	100.00%	1.00	00:00:00	0.00%	0 (0.00%)	$0.00 (0.00%)
624.	Nokia 7230	**5** **(0.01%)**	100.00%	5 (0.01%)	100.00%	1.00	00:00:00	0.00%	0 (0.00%)	$0.00 (0.00%)
625.	Nokia Asha 500	**5** **(0.01%)**	100.00%	5 (0.01%)	60.00%	7.00	00:05:29	0.00%	0 (0.00%)	$0.00 (0.00%)
626.	Nokia E71-2	**5** **(0.01%)**	100.00%	5 (0.01%)	20.00%	7.00	00:08:35	0.00%	0 (0.00%)	$0.00 (0.00%)
627.	Nokia Lumia 920T	**5** **(0.01%)**	60.00%	3 (0.01%)	40.00%	4.40	00:06:59	0.00%	0 (0.00%)	$0.00 (0.00%)
[illegible]	Nokia RM-899 [illegible]	[illegible]	[illegible]	[illegible]	[illegible]	[illegible]	[illegible]	[illegible]	[illegible]	[illegible]

628.	Nokia RM-899 Asha 501s	**5** **(0.01%)**	80.00%	4 (0.01%)	80.00%	1.60	00:00:14	0.00%	0 (0.00%)	$0.00 (0.00%)
629.	Nokia X6-00	**5** **(0.01%)**	100.00%	5 (0.01%)	80.00%	1.20	00:00:27	0.00%	0 (0.00%)	$0.00 (0.00%)
630.	Opera Mini 2	**5** **(0.01%)**	100.00%	5 (0.01%)	80.00%	1.20	00:02:18	0.00%	0 (0.00%)	$0.00 (0.00%)
631.	Pantech P9070 Burst	**5** **(0.01%)**	100.00%	5 (0.01%)	40.00%	2.40	00:00:24	0.00%	0 (0.00%)	$0.00 (0.00%)
632.	Pegatron Chagall	**5** **(0.01%)**	100.00%	5 (0.01%)	80.00%	1.40	00:00:22	0.00%	0 (0.00%)	$0.00 (0.00%)
633.	QMobile A2 Noir	**5** **(0.01%)**	100.00%	5 (0.01%)	80.00%	2.60	00:00:55	0.00%	0 (0.00%)	$0.00 (0.00%)
634.	Samsung GT-B5512	**5** **(0.01%)**	40.00%	2 (0.00%)	0.00%	5.00	00:10:17	0.00%	0 (0.00%)	$0.00 (0.00%)
635.	Samsung GT-I9000B Galaxy S Vibrant	**5** **(0.01%)**	80.00%	4 (0.01%)	40.00%	5.60	00:05:10	0.00%	0 (0.00%)	$0.00 (0.00%)
636.	Samsung GT-I9060 Galaxy Grand Neo	**5** **(0.01%)**	80.00%	4 (0.01%)	60.00%	2.00	00:00:25	0.00%	0 (0.00%)	$0.00 (0.00%)
637.	Samsung GT-I9070P Galaxy S Advance	**5** **(0.01%)**	100.00%	5 (0.01%)	60.00%	2.40	00:01:00	0.00%	0 (0.00%)	$0.00 (0.00%)
638.	Samsung GT-S5222 Star III Duos	**5** **(0.01%)**	100.00%	5 (0.01%)	80.00%	1.20	00:00:12	0.00%	0 (0.00%)	$0.00 (0.00%)
639.	Samsung GT-S5303 Galaxy Y Plus	**5** **(0.01%)**	60.00%	3 (0.01%)	40.00%	6.00	00:08:24	0.00%	0 (0.00%)	$0.00 (0.00%)
640.	Samsung GT-S6012 Galaxy Music Duos	**5** **(0.01%)**	80.00%	4 (0.01%)	40.00%	5.20	00:10:17	0.00%	0 (0.00%)	$0.00 (0.00%)
641.	Samsung SCH-I800 Galaxy Tab 7	**5** **(0.01%)**	100.00%	5 (0.01%)	60.00%	3.40	00:03:18	0.00%	0 (0.00%)	$0.00 (0.00%)
642.	Samsung SCH-i905 Galaxy Tab 10.1 4G	**5** **(0.01%)**	100.00%	5 (0.01%)	100.00%	1.00	00:00:00	0.00%	0 (0.00%)	$0.00 (0.00%)
643.	Samsung SCH-S720C Galaxy Proclaim	**5** **(0.01%)**	100.00%	5 (0.01%)	40.00%	2.60	00:00:41	0.00%	0 (0.00%)	$0.00 (0.00%)
644.	Samsung SGH-I747M Galaxy S III	**5** **(0.01%)**	100.00%	5 (0.01%)	60.00%	1.60	00:00:12	0.00%	0 (0.00%)	$0.00 (0.00%)
645.	Samsung SGH-I777 Galaxy S II	**5** **(0.01%)**	100.00%	5 (0.01%)	80.00%	1.20	00:00:22	0.00%	0 (0.00%)	$0.00 (0.00%)
646.	Samsung SGH-T679 Exhibit II 4G	**5** **(0.01%)**	100.00%	5 (0.01%)	80.00%	2.20	00:00:30	0.00%	0 (0.00%)	$0.00 (0.00%)
647.	Samsung SHV-E270K Galaxy Grand	**5** **(0.01%)**	80.00%	4 (0.01%)	40.00%	6.40	00:02:26	0.00%	0 (0.00%)	$0.00 (0.00%)
648.	Samsung SHW-M180S GALAXY Tab (SKT)	**5** **(0.01%)**	100.00%	5 (0.01%)	80.00%	2.40	00:00:40	0.00%	0 (0.00%)	$0.00 (0.00%)
649.	Samsung SM-G900A Galaxy S5	**5** **(0.01%)**	100.00%	5 (0.01%)	60.00%	1.80	00:00:12	0.00%	0 (0.00%)	$0.00 (0.00%)
650.	Samsung SM-N900W8 Galaxy Note 3	**5** **(0.01%)**	60.00%	3 (0.01%)	80.00%	1.40	00:00:06	0.00%	0 (0.00%)	$0.00 (0.00%)
651.	SonyEricsson E15i Xperia X8	**5** **(0.01%)**	80.00%	4 (0.01%)	60.00%	1.60	00:00:12	0.00%	0 (0.00%)	$0.00 (0.00%)
652.	SonyEricsson E16i Walkman 8	**5** **(0.01%)**	40.00%	2 (0.00%)	40.00%	8.20	00:10:02	0.00%	0 (0.00%)	$0.00 (0.00%)
653.	SonyEricsson ST15i Xperia Mini	**5** **(0.01%)**	100.00%	5 (0.01%)	80.00%	1.40	00:00:06	0.00%	0 (0.00%)	$0.00 (0.00%)
654.	Xiaomi 2013022	**5** **(0.01%)**	100.00%	5 (0.01%)	100.00%	1.00	00:00:00	0.00%	0 (0.00%)	$0.00 (0.00%)

654.	Red Rice	**5**	**(0.01%)**	100.00%	5	(0.01%)	100.00%	1.00	00:00:00	0.00%	0	(0.00%)	$0.00	(0.00%)
655.	ZTE N861 Warp	**5**	**(0.01%)**	60.00%	3	(0.01%)	60.00%	3.00	00:01:20	0.00%	0	(0.00%)	$0.00	(0.00%)
656.	Acer A100 Vangogh	**4**	**(0.01%)**	75.00%	3	(0.01%)	100.00%	1.00	00:00:00	0.00%	0	(0.00%)	$0.00	(0.00%)
657.	Alcatel OT-4030X S-POP	**4**	**(0.01%)**	100.00%	4	(0.01%)	50.00%	7.50	00:03:32	0.00%	0	(0.00%)	$0.00	(0.00%)
658.	Alcatel OT-7024W One Touch Fierce	**4**	**(0.01%)**	100.00%	4	(0.01%)	75.00%	1.50	00:06:52	0.00%	0	(0.00%)	$0.00	(0.00%)
659.	Alcatel v860 Smart II 3G	**4**	**(0.01%)**	50.00%	2	(0.00%)	100.00%	1.00	00:00:00	0.00%	0	(0.00%)	$0.00	(0.00%)
660.	Arnova AN10BG3 10b G3	**4**	**(0.01%)**	100.00%	4	(0.01%)	100.00%	1.00	00:00:00	0.00%	0	(0.00%)	$0.00	(0.00%)
661.	Asus Eee Pad Transformer TF101G Eee Pad Transformer TF101	**4**	**(0.01%)**	100.00%	4	(0.01%)	75.00%	1.75	00:00:11	0.00%	0	(0.00%)	$0.00	(0.00%)
662.	BlackBerry 9630 Tour	**4**	**(0.01%)**	75.00%	3	(0.01%)	50.00%	3.00	00:05:49	0.00%	0	(0.00%)	$0.00	(0.00%)
663.	Casio C771 G'zOne	**4**	**(0.01%)**	75.00%	3	(0.01%)	75.00%	1.50	00:04:00	0.00%	0	(0.00%)	$0.00	(0.00%)
664.	DoCoMo SO-02E Xperia Z	**4**	**(0.01%)**	100.00%	4	(0.01%)	100.00%	1.00	00:00:00	0.00%	0	(0.00%)	$0.00	(0.00%)
665.	DOOGEE DG500 Discovery	**4**	**(0.01%)**	100.00%	4	(0.01%)	75.00%	1.50	00:00:24	0.00%	0	(0.00%)	$0.00	(0.00%)
666.	Fly IQ238	**4**	**(0.01%)**	75.00%	3	(0.01%)	50.00%	3.50	00:01:42	0.00%	0	(0.00%)	$0.00	(0.00%)
667.	Fly IQ442	**4**	**(0.01%)**	50.00%	2	(0.00%)	75.00%	1.50	00:00:06	0.00%	0	(0.00%)	$0.00	(0.00%)
668.	HP Touchpad	**4**	**(0.01%)**	100.00%	4	(0.01%)	50.00%	2.75	00:00:14	0.00%	0	(0.00%)	$0.00	(0.00%)
669.	HTC A510e Wildfire S	**4**	**(0.01%)**	100.00%	4	(0.01%)	50.00%	2.00	00:01:21	0.00%	0	(0.00%)	$0.00	(0.00%)
670.	HTC A6366 Aria	**4**	**(0.01%)**	0.00%	0	(0.00%)	0.00%	8.25	00:10:55	0.00%	0	(0.00%)	$0.00	(0.00%)
671.	HTC SensationXE Beats Z715e Sensation	**4**	**(0.01%)**	75.00%	3	(0.01%)	25.00%	8.25	00:07:42	0.00%	0	(0.00%)	$0.00	(0.00%)
672.	HTC Spark 7 Trophy	**4**	**(0.01%)**	0.00%	0	(0.00%)	25.00%	5.25	00:03:57	0.00%	0	(0.00%)	$0.00	(0.00%)
673.	HTC T8698 7 Mozart	**4**	**(0.01%)**	100.00%	4	(0.01%)	100.00%	1.00	00:00:00	0.00%	0	(0.00%)	$0.00	(0.00%)
674.	Huawei C8813	**4**	**(0.01%)**	100.00%	4	(0.01%)	75.00%	1.50	00:00:13	0.00%	0	(0.00%)	$0.00	(0.00%)
675.	Huawei G510-0100 Ascend G510	**4**	**(0.01%)**	100.00%	4	(0.01%)	75.00%	2.00	00:00:23	0.00%	0	(0.00%)	$0.00	(0.00%)
676.	Huawei U8860 Honor	**4**	**(0.01%)**	100.00%	4	(0.01%)	75.00%	5.75	00:01:29	0.00%	0	(0.00%)	$0.00	(0.00%)
677.	Kyocera C5133 Event	**4**	**(0.01%)**	75.00%	3	(0.01%)	75.00%	2.25	00:00:50	0.00%	0	(0.00%)	$0.00	(0.00%)
678.	Kyocera C5155 Rise C5155	**4**	**(0.01%)**	100.00%	4	(0.01%)	100.00%	1.00	00:00:00	0.00%	0	(0.00%)	$0.00	(0.00%)
679.	Lenovo IdeaPad A1 IdeaPad A1 Tablet	**4**	**(0.01%)**	100.00%	4	(0.01%)	75.00%	1.50	00:00:13	0.00%	0	(0.00%)	$0.00	(0.00%)
680.	LG E970 Optimus G	**4**	**(0.01%)**	100.00%	4	(0.01%)	100.00%	1.00	00:00:00	0.00%	0	(0.00%)	$0.00	(0.00%)
681.	LG L35G Optimus Logic	**4**	**(0.01%)**	100.00%	4	(0.01%)	100.00%	1.00	00:00:00	0.00%	0	(0.00%)	$0.00	(0.00%)
682.	LG P970 Optimus Black	**4**	**(0.01%)**	100.00%	4	(0.01%)	100.00%	1.00	00:00:00	0.00%	0	(0.00%)	$0.00	(0.00%)

683.	LG T385	**4**	**(0.01%)**	100.00%	4	(0.01%)	100.00%	1.00	00:00:00	0.00%	0 (0.00%)	$0.00	(0.00%)
684.	Micromax A35 Bolt Ninja A35	**4**	**(0.01%)**	100.00%	4	(0.01%)	50.00%	2.50	00:03:08	0.00%	0 (0.00%)	$0.00	(0.00%)
685.	Motorola Droid 2	**4**	**(0.01%)**	75.00%	3	(0.01%)	25.00%	12.25	00:20:50	0.00%	0 (0.00%)	$0.00	(0.00%)
686.	Motorola MB855 Photon	**4**	**(0.01%)**	100.00%	4	(0.01%)	75.00%	1.50	00:00:08	0.00%	0 (0.00%)	$0.00	(0.00%)
687.	Motorola MOT-EX225	**4**	**(0.01%)**	75.00%	3	(0.01%)	75.00%	1.25	00:00:12	0.00%	0 (0.00%)	$0.00	(0.00%)
688.	Motorola MotoMZ616 Xoom 2	**4**	**(0.01%)**	25.00%	1	(0.00%)	100.00%	1.00	00:00:00	0.00%	0 (0.00%)	$0.00	(0.00%)
689.	Motorola XT1033 DVX	**4**	**(0.01%)**	50.00%	2	(0.00%)	75.00%	3.25	00:01:19	0.00%	0 (0.00%)	$0.00	(0.00%)
690.	Motorola XT915 RAZR D1	**4**	**(0.01%)**	25.00%	1	(0.00%)	100.00%	1.00	00:00:00	0.00%	0 (0.00%)	$0.00	(0.00%)
691.	Nokia 301 Asha 301	**4**	**(0.01%)**	75.00%	3	(0.01%)	50.00%	1.75	00:00:27	0.00%	0 (0.00%)	$0.00	(0.00%)
692.	Nokia 306	**4**	**(0.01%)**	100.00%	4	(0.01%)	100.00%	1.00	00:00:00	0.00%	0 (0.00%)	$0.00	(0.00%)
693.	Nokia 5130 XpressMusic	**4**	**(0.01%)**	100.00%	4	(0.01%)	75.00%	1.25	00:00:09	0.00%	0 (0.00%)	$0.00	(0.00%)
694.	Nokia E7-00	**4**	**(0.01%)**	100.00%	4	(0.01%)	100.00%	1.00	00:00:00	0.00%	0 (0.00%)	$0.00	(0.00%)
695.	Nokia Lumia 1520	**4**	**(0.01%)**	100.00%	4	(0.01%)	75.00%	1.25	00:00:05	0.00%	0 (0.00%)	$0.00	(0.00%)
696.	Pandigital Novel	**4**	**(0.01%)**	100.00%	4	(0.01%)	50.00%	7.00	00:06:57	0.00%	0 (0.00%)	$0.00	(0.00%)
697.	Pantech IM-A760S SKY Vega Racer (SKT)	**4**	**(0.01%)**	100.00%	4	(0.01%)	75.00%	1.25	00:00:03	0.00%	0 (0.00%)	$0.00	(0.00%)
698.	Pantech IM-A830S Vega Racer 2	**4**	**(0.01%)**	25.00%	1	(0.00%)	25.00%	6.75	00:15:22	0.00%	0 (0.00%)	$0.00	(0.00%)
699.	Pantech P4100 Element	**4**	**(0.01%)**	50.00%	2	(0.00%)	100.00%	1.00	00:00:00	0.00%	0 (0.00%)	$0.00	(0.00%)
700.	Polaroid MIDC410	**4**	**(0.01%)**	100.00%	4	(0.01%)	50.00%	3.75	00:01:27	0.00%	0 (0.00%)	$0.00	(0.00%)
701.	Prestigio PMP5080CPRO MultiPad 5080 Pro	**4**	**(0.01%)**	25.00%	1	(0.00%)	100.00%	1.00	00:00:00	0.00%	0 (0.00%)	$0.00	(0.00%)
702.	Samsung GT-B5510 Galaxy Y	**4**	**(0.01%)**	100.00%	4	(0.01%)	50.00%	1.75	00:00:28	0.00%	0 (0.00%)	$0.00	(0.00%)
703.	Samsung GT-C3222	**4**	**(0.01%)**	50.00%	2	(0.00%)	75.00%	1.25	00:00:09	0.00%	0 (0.00%)	$0.00	(0.00%)
704.	Samsung GT-I9210T Galaxy SII 4G	**4**	**(0.01%)**	100.00%	4	(0.01%)	100.00%	1.00	00:00:00	0.00%	0 (0.00%)	$0.00	(0.00%)
705.	Samsung GT-P7501 Galaxy Tab 10.1N	**4**	**(0.01%)**	100.00%	4	(0.01%)	100.00%	1.00	00:00:00	0.00%	0 (0.00%)	$0.00	(0.00%)
706.	Samsung GT-S5270L Ch@t 527	**4**	**(0.01%)**	100.00%	4	(0.01%)	50.00%	1.75	00:01:30	0.00%	0 (0.00%)	$0.00	(0.00%)
707.	Samsung GT-S5303B Galaxy Y Plus	**4**	**(0.01%)**	50.00%	2	(0.00%)	50.00%	2.75	00:01:05	0.00%	0 (0.00%)	$0.00	(0.00%)
708.	Samsung GT-S5369 Galaxy Y	**4**	**(0.01%)**	100.00%	4	(0.01%)	100.00%	1.00	00:00:00	0.00%	0 (0.00%)	$0.00	(0.00%)
709.	Samsung GT-S7270L Galaxy Ace 3	**4**	**(0.01%)**	100.00%	4	(0.01%)	100.00%	1.00	00:00:00	0.00%	0 (0.00%)	$0.00	(0.00%)
710.	Samsung GT-S7275 Galaxy Ace 3 LTE	**4**	**(0.01%)**	75.00%	3	(0.01%)	50.00%	2.50	00:01:02	0.00%	0 (0.00%)	$0.00	(0.00%)

711.	Samsung SC-06D Galaxy S III	**4**	**(0.01%)**	100.00%	4	(0.01%)	75.00%	3.50	00:02:11	0.00%	0	(0.00%)	$0.00 (0.00%)
712.	Samsung SCH-I415 Galaxy Stratosphere II	**4**	**(0.01%)**	100.00%	4	(0.01%)	75.00%	1.50	00:08:10	0.00%	0	(0.00%)	$0.00 (0.00%)
713.	Samsung SCH-i509 Galaxy Young CDMA	**4**	**(0.01%)**	50.00%	2	(0.00%)	100.00%	1.00	00:00:00	0.00%	0	(0.00%)	$0.00 (0.00%)
714.	Samsung SCH-I925 Galaxy Note 10.1 LTE	**4**	**(0.01%)**	100.00%	4	(0.01%)	75.00%	1.75	00:00:09	0.00%	0	(0.00%)	$0.00 (0.00%)
715.	Samsung SCH-R530M Galaxy S III	**4**	**(0.01%)**	100.00%	4	(0.01%)	50.00%	2.25	00:00:16	0.00%	0	(0.00%)	$0.00 (0.00%)
716.	Samsung SCH-U660 Convoy 2	**4**	**(0.01%)**	100.00%	4	(0.01%)	75.00%	1.75	00:00:20	0.00%	0	(0.00%)	$0.00 (0.00%)
717.	Samsung SHV-E160L Galaxy Note	**4**	**(0.01%)**	100.00%	4	(0.01%)	75.00%	1.25	00:00:04	0.00%	0	(0.00%)	$0.00 (0.00%)
718.	Samsung SM-G3815 Galaxy Express 2	**4**	**(0.01%)**	100.00%	4	(0.01%)	75.00%	2.00	00:00:34	0.00%	0	(0.00%)	$0.00 (0.00%)
719.	Samsung SM-N9006 Galaxy Note 3	**4**	**(0.01%)**	50.00%	2	(0.00%)	75.00%	1.25	00:01:48	0.00%	0	(0.00%)	$0.00 (0.00%)
720.	Samsung SM-N900P Galaxy Note 3	**4**	**(0.01%)**	50.00%	2	(0.00%)	75.00%	1.25	00:00:07	0.00%	0	(0.00%)	$0.00 (0.00%)
721.	Samsung SM-P601	**4**	**(0.01%)**	100.00%	4	(0.01%)	75.00%	2.75	00:01:05	0.00%	0	(0.00%)	$0.00 (0.00%)
722.	Samsung SM-T217S Galaxy Tab 3 7.0	**4**	**(0.01%)**	50.00%	2	(0.00%)	50.00%	1.75	00:00:16	0.00%	0	(0.00%)	$0.00 (0.00%)
723.	Samsung SPH-L300 Galaxy Victory 4G LTE	**4**	**(0.01%)**	75.00%	3	(0.01%)	50.00%	2.50	00:00:37	0.00%	0	(0.00%)	$0.00 (0.00%)
724.	Samsung SPH-M840 Galaxy Ring	**4**	**(0.01%)**	100.00%	4	(0.01%)	75.00%	1.25	00:00:01	0.00%	0	(0.00%)	$0.00 (0.00%)
725.	Sony C6503 Xperia ZL	**4**	**(0.01%)**	75.00%	3	(0.01%)	75.00%	2.00	00:00:14	0.00%	0	(0.00%)	$0.00 (0.00%)
726.	Sony C6833 Xperia Z Ultra	**4**	**(0.01%)**	75.00%	3	(0.01%)	50.00%	2.00	00:00:18	0.00%	0	(0.00%)	$0.00 (0.00%)
727.	Sony SonySO-03E Xperia Tablet Z LTE	**4**	**(0.01%)**	100.00%	4	(0.01%)	75.00%	1.25	00:00:02	0.00%	0	(0.00%)	$0.00 (0.00%)
728.	SonyEricsson LT26w Xperia Acro S	**4**	**(0.01%)**	75.00%	3	(0.01%)	75.00%	2.00	00:00:37	0.00%	0	(0.00%)	$0.00 (0.00%)
729.	SonyEricsson LT28i Xperia Ion	**4**	**(0.01%)**	100.00%	4	(0.01%)	75.00%	1.75	00:00:49	0.00%	0	(0.00%)	$0.00 (0.00%)
730.	SonyEricsson MT25i Xperia neo L	**4**	**(0.01%)**	100.00%	4	(0.01%)	75.00%	1.75	00:00:55	0.00%	0	(0.00%)	$0.00 (0.00%)
731.	SonyEricsson U20i Xperia X10 Mini pro	**4**	**(0.01%)**	100.00%	4	(0.01%)	75.00%	2.00	00:01:07	0.00%	0	(0.00%)	$0.00 (0.00%)
732.	SonyEricsson X10a Xperia X10	**4**	**(0.01%)**	75.00%	3	(0.01%)	75.00%	5.25	00:01:05	0.00%	0	(0.00%)	$0.00 (0.00%)
733.	T-Mobile myTouch myTouch	**4**	**(0.01%)**	75.00%	3	(0.01%)	50.00%	2.75	00:04:14	0.00%	0	(0.00%)	$0.00 (0.00%)
734.	T-Mobile myTouch myTouch Q	**4**	**(0.01%)**	100.00%	4	(0.01%)	100.00%	1.00	00:00:00	0.00%	0	(0.00%)	$0.00 (0.00%)
735.	Tecno N3	**4**	**(0.01%)**	50.00%	2	(0.00%)	50.00%	1.50	00:01:35	0.00%	0	(0.00%)	$0.00 (0.00%)

736.	Tesco Hudl HT7S3	**4** **(0.01%)**	75.00%	3 (0.01%)	25.00%	2.50	00:02:43	0.00%	0 (0.00%)	$0.00 (0.00%)
737.	Virgin Mobile Chaser	**4** **(0.01%)**	50.00%	2 (0.00%)	50.00%	2.75	00:00:50	0.00%	0 (0.00%)	$0.00 (0.00%)
738.	Woo Comet PAD-703	**4** **(0.01%)**	25.00%	1 (0.00%)	25.00%	2.25	00:07:43	0.00%	0 (0.00%)	$0.00 (0.00%)
739.	Xiaomi MI 2S	**4** **(0.01%)**	100.00%	4 (0.01%)	100.00%	1.00	00:00:00	0.00%	0 (0.00%)	$0.00 (0.00%)
740.	Xiaomi MI-2C	**4** **(0.01%)**	25.00%	1 (0.00%)	100.00%	1.00	00:00:00	0.00%	0 (0.00%)	$0.00 (0.00%)
741.	ZTE N860	**4** **(0.01%)**	75.00%	3 (0.01%)	75.00%	1.75	00:00:57	0.00%	0 (0.00%)	$0.00 (0.00%)
742.	ZTE U V856 Movistar One	**4** **(0.01%)**	100.00%	4 (0.01%)	75.00%	7.50	00:09:57	0.00%	0 (0.00%)	$0.00 (0.00%)
743.	ZTE X500 Score	**4** **(0.01%)**	25.00%	1 (0.00%)	25.00%	2.75	00:02:04	0.00%	0 (0.00%)	$0.00 (0.00%)
744.	Acer A511	**3** **(0.00%)**	66.67%	2 (0.00%)	100.00%	1.00	00:00:00	0.00%	0 (0.00%)	$0.00 (0.00%)
745.	Acer V370 Liquid E2 Duo	**3** **(0.00%)**	100.00%	3 (0.01%)	66.67%	4.33	00:03:31	0.00%	0 (0.00%)	$0.00 (0.00%)
746.	Alcatel One Touch 6033A Idol Ultra	**3** **(0.00%)**	100.00%	3 (0.01%)	66.67%	3.00	00:02:32	0.00%	0 (0.00%)	$0.00 (0.00%)
747.	Alcatel one touch 906Y one touch 906Y	**3** **(0.00%)**	100.00%	3 (0.01%)	100.00%	1.00	00:00:00	0.00%	0 (0.00%)	$0.00 (0.00%)
748.	Alcatel OT-6010X One Touch Star 6010X	**3** **(0.00%)**	66.67%	2 (0.00%)	66.67%	1.67	00:01:40	0.00%	0 (0.00%)	$0.00 (0.00%)
749.	Alcatel OT-6030X One Touch Idol	**3** **(0.00%)**	100.00%	3 (0.01%)	100.00%	1.00	00:00:00	0.00%	0 (0.00%)	$0.00 (0.00%)
750.	Alcatel OT-992D One Touch 992D	**3** **(0.00%)**	100.00%	3 (0.01%)	100.00%	1.00	00:00:00	0.00%	0 (0.00%)	$0.00 (0.00%)
751.	Alcatel OT-T10 One Touch T10	**3** **(0.00%)**	66.67%	2 (0.00%)	66.67%	1.67	00:00:11	0.00%	0 (0.00%)	$0.00 (0.00%)
752.	BlackBerry 8530 Curve	**3** **(0.00%)**	100.00%	3 (0.01%)	33.33%	2.33	00:01:46	0.00%	0 (0.00%)	$0.00 (0.00%)
753.	Coby MID8048 Kyros MID8048	**3** **(0.00%)**	100.00%	3 (0.01%)	33.33%	2.00	00:00:33	0.00%	0 (0.00%)	$0.00 (0.00%)
754.	Dell Streak	**3** **(0.00%)**	100.00%	3 (0.01%)	33.33%	4.00	00:01:05	0.00%	0 (0.00%)	$0.00 (0.00%)
755.	DoCoMo N-08D MEDIAS TAB UL	**3** **(0.00%)**	100.00%	3 (0.01%)	66.67%	1.67	00:00:25	0.00%	0 (0.00%)	$0.00 (0.00%)
756.	Flytouch VC882 FJ Vortex	**3** **(0.00%)**	66.67%	2 (0.00%)	66.67%	9.33	00:35:34	0.00%	0 (0.00%)	$0.00 (0.00%)
757.	HTC A3333 Wildfire	**3** **(0.00%)**	100.00%	3 (0.01%)	33.33%	1.67	00:00:49	0.00%	0 (0.00%)	$0.00 (0.00%)
758.	HTC A9191 Desire HD	**3** **(0.00%)**	100.00%	3 (0.01%)	66.67%	1.33	00:00:04	0.00%	0 (0.00%)	$0.00 (0.00%)
759.	HTC ADR6425LVW Rezound	**3** **(0.00%)**	100.00%	3 (0.01%)	100.00%	1.00	00:00:00	0.00%	0 (0.00%)	$0.00 (0.00%)
760.	HTC APA7373KT EVO Shift 4G	**3** **(0.00%)**	66.67%	2 (0.00%)	66.67%	1.67	00:00:55	0.00%	0 (0.00%)	$0.00 (0.00%)
761.	HTC HTC6500LVW 4G One	**3** **(0.00%)**	100.00%	3 (0.01%)	100.00%	1.00	00:00:00	0.00%	0 (0.00%)	$0.00 (0.00%)
762.	HTC myTouch 4G Slide	**3** **(0.00%)**	100.00%	3 (0.01%)	66.67%	3.00	00:01:06	0.00%	0 (0.00%)	$0.00 (0.00%)
763.	HTC One SV	**3** **(0.00%)**	100.00%	3 (0.01%)	33.33%	2.00	00:00:17	0.00%	0 (0.00%)	$0.00 (0.00%)
764.	HTC S710E Incredible S	**3** **(0.00%)**	100.00%	3 (0.01%)	66.67%	1.33	00:00:13	0.00%	0 (0.00%)	$0.00 (0.00%)
765.	HTC T9292 HD7	**3** **(0.00%)**	100.00%	3 (0.01%)	66.67%	1.67	00:00:16	0.00%	0 (0.00%)	$0.00 (0.00%)
	[illegible]									

766.	HTC Touch Pro Raphael 100	**3** **(0.00%)**	33.33%	1 (0.00%)	66.67%	2.33	00:04:15	0.00%	0 (0.00%)	$0.00 (0.00%)
767.	HTC X710a Raider 4G	**3** **(0.00%)**	100.00%	3 (0.01%)	66.67%	1.67	00:00:29	0.00%	0 (0.00%)	$0.00 (0.00%)
768.	HTC Z710 Sensation 4G	**3** **(0.00%)**	100.00%	3 (0.01%)	100.00%	1.00	00:00:00	0.00%	0 (0.00%)	$0.00 (0.00%)
769.	HTC Zara Desire 601	**3** **(0.00%)**	100.00%	3 (0.01%)	100.00%	1.00	00:00:00	0.00%	0 (0.00%)	$0.00 (0.00%)
770.	Huawei G510-0010 Ascend G510	**3** **(0.00%)**	100.00%	3 (0.01%)	0.00%	2.00	00:01:25	0.00%	0 (0.00%)	$0.00 (0.00%)
771.	Huawei M931 Premia 4G	**3** **(0.00%)**	66.67%	2 (0.00%)	100.00%	1.00	00:00:00	0.00%	0 (0.00%)	$0.00 (0.00%)
772.	Huawei S7-301w Mediapad	**3** **(0.00%)**	66.67%	2 (0.00%)	100.00%	1.00	00:00:00	0.00%	0 (0.00%)	$0.00 (0.00%)
773.	Huawei U8800-51 Impulse 4G for AT&T	**3** **(0.00%)**	100.00%	3 (0.01%)	100.00%	1.00	00:00:00	0.00%	0 (0.00%)	$0.00 (0.00%)
774.	Huawei U8815 Ascent G 300	**3** **(0.00%)**	100.00%	3 (0.01%)	33.33%	5.33	00:03:28	0.00%	0 (0.00%)	$0.00 (0.00%)
775.	Huawei U9508 Honor 2	**3** **(0.00%)**	100.00%	3 (0.01%)	66.67%	2.67	00:00:56	0.00%	0 (0.00%)	$0.00 (0.00%)
776.	Huawei UM840 Evolution	**3** **(0.00%)**	100.00%	3 (0.01%)	100.00%	1.00	00:00:00	0.00%	0 (0.00%)	$0.00 (0.00%)
777.	Huawei W1-U34 Ascend W1	**3** **(0.00%)**	33.33%	1 (0.00%)	33.33%	4.00	00:01:49	0.00%	0 (0.00%)	$0.00 (0.00%)
778.	Karbonn A111 Smart 111	**3** **(0.00%)**	66.67%	2 (0.00%)	33.33%	7.33	00:16:07	0.00%	0 (0.00%)	$0.00 (0.00%)
779.	Karbonn Titaniur S5	**3** **(0.00%)**	100.00%	3 (0.01%)	100.00%	1.00	00:00:00	0.00%	0 (0.00%)	$0.00 (0.00%)
780.	KDDI SHL21 Aquos Phone SHL21	**3** **(0.00%)**	100.00%	3 (0.01%)	100.00%	1.00	00:00:00	0.00%	0 (0.00%)	$0.00 (0.00%)
781.	Kyocera ISW11K Digno ISW11K	**3** **(0.00%)**	100.00%	3 (0.01%)	100.00%	1.00	00:00:00	0.00%	0 (0.00%)	$0.00 (0.00%)
782.	Lava 501 Iris 501	**3** **(0.00%)**	100.00%	3 (0.01%)	100.00%	1.00	00:00:00	0.00%	0 (0.00%)	$0.00 (0.00%)
783.	Lava N400 Iris N400	**3** **(0.00%)**	100.00%	3 (0.01%)	66.67%	1.67	00:02:59	0.00%	0 (0.00%)	$0.00 (0.00%)
784.	Lenovo A850	**3** **(0.00%)**	100.00%	3 (0.01%)	100.00%	1.00	00:00:00	0.00%	0 (0.00%)	$0.00 (0.00%)
785.	Lenovo S890	**3** **(0.00%)**	0.00%	0 (0.00%)	0.00%	24.67	00:13:51	0.00%	0 (0.00%)	$0.00 (0.00%)
786.	LG D801 G2	**3** **(0.00%)**	33.33%	1 (0.00%)	100.00%	1.00	00:00:00	0.00%	0 (0.00%)	$0.00 (0.00%)
787.	LG E405 Optimus L3	**3** **(0.00%)**	100.00%	3 (0.01%)	66.67%	1.33	00:00:11	0.00%	0 (0.00%)	$0.00 (0.00%)
788.	LG E612 Optimus L5	**3** **(0.00%)**	100.00%	3 (0.01%)	66.67%	1.67	00:00:12	0.00%	0 (0.00%)	$0.00 (0.00%)
789.	LG E900 Optimus 7	**3** **(0.00%)**	66.67%	2 (0.00%)	33.33%	6.00	00:03:22	0.00%	0 (0.00%)	$0.00 (0.00%)
790.	LG LG/D500	**3** **(0.00%)**	100.00%	3 (0.01%)	100.00%	1.00	00:00:00	0.00%	0 (0.00%)	$0.00 (0.00%)
791.	LG LG730 Venice	**3** **(0.00%)**	100.00%	3 (0.01%)	100.00%	1.00	00:00:00	0.00%	0 (0.00%)	$0.00 (0.00%)
792.	LG LG870 Optimus F7	**3** **(0.00%)**	100.00%	3 (0.01%)	66.67%	5.67	00:02:40	0.00%	0 (0.00%)	$0.00 (0.00%)
793.	LG LS720 Optimus F3	**3** **(0.00%)**	100.00%	3 (0.01%)	100.00%	1.00	00:00:00	0.00%	0 (0.00%)	$0.00 (0.00%)
794.	LG P500	**3** **(0.00%)**	100.00%	3 (0.01%)	100.00%	1.00	00:00:00	0.00%	0 (0.00%)	$0.00 (0.00%)
795.	LG P690f Optimus Spirit	**3** **(0.00%)**	100.00%	3 (0.01%)	33.33%	5.00	00:01:46	0.00%	0 (0.00%)	$0.00 (0.00%)
796.	LG P716 Optimus L7 II	**3** **(0.00%)**	100.00%	3 (0.01%)	66.67%	3.00	00:00:44	0.00%	0 (0.00%)	$0.00 (0.00%)

797.	LG P720h Optimus 3D Max	**3** **(0.00%)**	100.00%	3 (0.01%)	33.33%	3.67	00:03:22	0.00%	0 (0.00%)	$0.00 (0.00%)
798.	LG P990 Optimus 2X	**3** **(0.00%)**	100.00%	3 (0.01%)	100.00%	1.00	00:00:00	0.00%	0 (0.00%)	$0.00 (0.00%)
799.	LG SU640 Optimus LTE	**3** **(0.00%)**	33.33%	1 (0.00%)	100.00%	1.00	00:00:00	0.00%	0 (0.00%)	$0.00 (0.00%)
800.	LG VS950 4G Intuition	**3** **(0.00%)**	66.67%	2 (0.00%)	66.67%	2.33	00:00:15	0.00%	0 (0.00%)	$0.00 (0.00%)
801.	MediaTek Y220-U05	**3** **(0.00%)**	33.33%	1 (0.00%)	0.00%	12.67	00:09:30	0.00%	0 (0.00%)	$0.00 (0.00%)
802.	Micromax A111 Canvas Doodle	**3** **(0.00%)**	100.00%	3 (0.01%)	100.00%	1.00	00:00:00	0.00%	0 (0.00%)	$0.00 (0.00%)
803.	Micromax A25 A25 Smarty	**3** **(0.00%)**	100.00%	3 (0.01%)	66.67%	1.33	00:00:27	0.00%	0 (0.00%)	$0.00 (0.00%)
804.	Micromax A57 Ninja 3	**3** **(0.00%)**	100.00%	3 (0.01%)	66.67%	1.67	00:01:11	0.00%	0 (0.00%)	$0.00 (0.00%)
805.	Micromax A74 Canvas Fun	**3** **(0.00%)**	100.00%	3 (0.01%)	66.67%	1.33	00:01:50	0.00%	0 (0.00%)	$0.00 (0.00%)
806.	Micromax A88	**3** **(0.00%)**	66.67%	2 (0.00%)	100.00%	1.00	00:00:00	0.00%	0 (0.00%)	$0.00 (0.00%)
807.	Micromax A92	**3** **(0.00%)**	100.00%	3 (0.01%)	33.33%	1.67	00:00:57	0.00%	0 (0.00%)	$0.00 (0.00%)
808.	Motorola EX118 MOTOKEY XT	**3** **(0.00%)**	100.00%	3 (0.01%)	100.00%	1.00	00:00:00	0.00%	0 (0.00%)	$0.00 (0.00%)
809.	Motorola MZ608 XOOM 2 ME 3G	**3** **(0.00%)**	100.00%	3 (0.01%)	100.00%	1.00	00:00:00	0.00%	0 (0.00%)	$0.00 (0.00%)
810.	Motorola XT531 Fire XT	**3** **(0.00%)**	100.00%	3 (0.01%)	33.33%	5.33	00:05:43	0.00%	0 (0.00%)	$0.00 (0.00%)
811.	Nokia 2055 Asha 2055	**3** **(0.00%)**	100.00%	3 (0.01%)	66.67%	1.33	00:00:17	0.00%	0 (0.00%)	$0.00 (0.00%)
812.	Nokia 603	**3** **(0.00%)**	100.00%	3 (0.01%)	66.67%	1.67	00:00:17	0.00%	0 (0.00%)	$0.00 (0.00%)
813.	Nokia 6120 classic	**3** **(0.00%)**	100.00%	3 (0.01%)	33.33%	2.67	00:01:50	0.00%	0 (0.00%)	$0.00 (0.00%)
814.	Nokia C2-00	**3** **(0.00%)**	100.00%	3 (0.01%)	0.00%	4.00	00:02:32	0.00%	0 (0.00%)	$0.00 (0.00%)
815.	Nokia Lumia 928	**3** **(0.00%)**	100.00%	3 (0.01%)	66.67%	2.00	00:00:43	0.00%	0 (0.00%)	$0.00 (0.00%)
816.	Oppo R821T FInd Muse	**3** **(0.00%)**	100.00%	3 (0.01%)	66.67%	3.00	00:00:57	0.00%	0 (0.00%)	$0.00 (0.00%)
817.	Pantech IM-A850S Vega R3	**3** **(0.00%)**	100.00%	3 (0.01%)	33.33%	3.67	00:01:30	0.00%	0 (0.00%)	$0.00 (0.00%)
818.	Pomp W89	**3** **(0.00%)**	33.33%	1 (0.00%)	0.00%	12.33	00:22:44	0.00%	0 (0.00%)	$0.00 (0.00%)
819.	Samsung GT-E2252	**3** **(0.00%)**	100.00%	3 (0.01%)	100.00%	1.00	00:00:00	0.00%	0 (0.00%)	$0.00 (0.00%)
820.	Samsung GT-I9100T Galaxy S II	**3** **(0.00%)**	100.00%	3 (0.01%)	100.00%	1.00	00:00:00	0.00%	0 (0.00%)	$0.00 (0.00%)
821.	Samsung GT-I9103 Galaxy SII	**3** **(0.00%)**	66.67%	2 (0.00%)	100.00%	1.00	00:00:00	0.00%	0 (0.00%)	$0.00 (0.00%)
822.	Samsung GT-N7105T Galaxy Note II	**3** **(0.00%)**	100.00%	3 (0.01%)	100.00%	1.00	00:00:00	0.00%	0 (0.00%)	$0.00 (0.00%)
823.	Samsung GT-P7310 Galaxy Tab 8.9	**3** **(0.00%)**	100.00%	3 (0.01%)	33.33%	2.67	00:01:47	0.00%	0 (0.00%)	$0.00 (0.00%)
824.	Samsung GT-S3850	**3** **(0.00%)**	100.00%	3 (0.01%)	33.33%	1.67	00:00:19	0.00%	0 (0.00%)	$0.00 (0.00%)
825.	Samsung GT-S5310B Galaxy Pocket Neo	**3** **(0.00%)**	100.00%	3 (0.01%)	66.67%	3.67	00:00:14	0.00%	0 (0.00%)	$0.00 (0.00%)
826.	Samsung GT-S5363	**3** **(0.00%)**	66.67%	2 (0.00%)	100.00%	1.00	00:00:00	0.00%	0 (0.00%)	$0.00 (0.00%)
827.	Samsung GT-	**3** **(0.00%)**	100.00%	3 (0.01%)	66.67%	1.33	00:00:43	0.00%	0 (0.00%)	$0.00 (0.00%)

827.	[illegible] S5380	**3** **(0.00%)**	100.00%	3 (0.01%)	66.67%	1.33	[illegible]	0.00%	0 (0.00%)	$0.00 (0.00%)
828.	Samsung GT-S5830T Galaxy Ace	**3** **(0.00%)**	100.00%	3 (0.01%)	66.67%	1.33	00:00:08	0.00%	0 (0.00%)	$0.00 (0.00%)
829.	Samsung GT-S8600 Wave 3	**3** **(0.00%)**	100.00%	3 (0.01%)	66.67%	1.67	00:00:16	0.00%	0 (0.00%)	$0.00 (0.00%)
830.	Samsung SC-02C Galaxy S II SC-02C for DoCoMo	**3** **(0.00%)**	100.00%	3 (0.01%)	100.00%	1.00	00:00:00	0.00%	0 (0.00%)	$0.00 (0.00%)
831.	Samsung SCH-I747M Galaxy S III	**3** **(0.00%)**	100.00%	3 (0.01%)	100.00%	1.00	00:00:00	0.00%	0 (0.00%)	$0.00 (0.00%)
832.	Samsung SCH-R920 Galaxy Attain 4G	**3** **(0.00%)**	66.67%	2 (0.00%)	100.00%	1.00	00:00:00	0.00%	0 (0.00%)	$0.00 (0.00%)
833.	Samsung SCH-R970 Galaxy S IV	**3** **(0.00%)**	100.00%	3 (0.01%)	66.67%	1.67	00:00:19	0.00%	0 (0.00%)	$0.00 (0.00%)
834.	Samsung SGH-S959G Galaxy S II	**3** **(0.00%)**	100.00%	3 (0.01%)	100.00%	1.00	00:00:00	0.00%	0 (0.00%)	$0.00 (0.00%)
835.	Samsung SGH-X100	**3** **(0.00%)**	100.00%	3 (0.01%)	66.67%	1.33	00:00:10	0.00%	0 (0.00%)	$0.00 (0.00%)
836.	Samsung SHV-E230S Galaxy Note 10.1	**3** **(0.00%)**	33.33%	1 (0.00%)	33.33%	13.33	00:03:46	0.00%	0 (0.00%)	$0.00 (0.00%)
837.	Samsung SHV-E330S Galaxy S IV	**3** **(0.00%)**	33.33%	1 (0.00%)	66.67%	5.00	00:08:52	0.00%	0 (0.00%)	$0.00 (0.00%)
838.	Samsung SHW-M250S GALAXY S II (SKT)	**3** **(0.00%)**	100.00%	3 (0.01%)	66.67%	3.33	00:01:22	0.00%	0 (0.00%)	$0.00 (0.00%)
839.	Samsung SHW-M480S Galaxy Note 10.1	**3** **(0.00%)**	100.00%	3 (0.01%)	0.00%	4.00	00:01:17	0.00%	0 (0.00%)	$0.00 (0.00%)
840.	Samsung SM-P605 Galaxy Note 10.1 2014	**3** **(0.00%)**	100.00%	3 (0.01%)	66.67%	1.67	00:00:10	0.00%	0 (0.00%)	$0.00 (0.00%)
841.	Samsung SPH-L300 Gogh	**3** **(0.00%)**	100.00%	3 (0.01%)	66.67%	2.00	00:01:13	0.00%	0 (0.00%)	$0.00 (0.00%)
842.	Samsung SPH-L720 Galaxy S4	**3** **(0.00%)**	100.00%	3 (0.01%)	100.00%	1.00	00:00:00	0.00%	0 (0.00%)	$0.00 (0.00%)
843.	Samsung SPH-M820 Galaxy Prevail	**3** **(0.00%)**	100.00%	3 (0.01%)	66.67%	1.33	00:00:12	0.00%	0 (0.00%)	$0.00 (0.00%)
844.	Samsung SPH-M900 Moment	**3** **(0.00%)**	100.00%	3 (0.01%)	66.67%	8.67	00:06:50	0.00%	0 (0.00%)	$0.00 (0.00%)
845.	Samsung YP-GI1 Galaxy Player 4.2	**3** **(0.00%)**	100.00%	3 (0.01%)	100.00%	1.00	00:00:00	0.00%	0 (0.00%)	$0.00 (0.00%)
846.	Sharp A01 INFOBAR A01 for KDDI au iida	**3** **(0.00%)**	66.67%	2 (0.00%)	100.00%	1.00	00:00:00	0.00%	0 (0.00%)	$0.00 (0.00%)
847.	Sharp IS05 IS05 for KDDI	**3** **(0.00%)**	33.33%	1 (0.00%)	33.33%	4.00	00:00:57	0.00%	0 (0.00%)	$0.00 (0.00%)
848.	Smartfren Andromax-c	**3** **(0.00%)**	100.00%	3 (0.01%)	100.00%	1.00	00:00:00	0.00%	0 (0.00%)	$0.00 (0.00%)
849.	Sony C6902 Xperia Z1	**3** **(0.00%)**	66.67%	2 (0.00%)	66.67%	2.00	00:00:15	0.00%	0 (0.00%)	$0.00 (0.00%)
850.	Sony D5503 Xperia Z1 Compact	**3** **(0.00%)**	100.00%	3 (0.01%)	100.00%	1.00	00:00:00	0.00%	0 (0.00%)	$0.00 (0.00%)
851.	Sony Ericsson LT26ii Xperia SL	**3** **(0.00%)**	100.00%	3 (0.01%)	66.67%	1.67	00:00:24	0.00%	0 (0.00%)	$0.00 (0.00%)
852.	SonyEricsson C6606 Xperia Z	**3** **(0.00%)**	100.00%	3 (0.01%)	100.00%	1.00	00:00:00	0.00%	0 (0.00%)	$0.00 (0.00%)

853.	SonyEricsson MT11a Xperia Neo V	**3** **(0.00%)**	100.00%	3 (0.01%)	33.33%	2.33	00:01:18	0.00%	0 (0.00%)	$0.00 (0.00%)
854.	SonyEricsson MT15a Xperia Neo	**3** **(0.00%)**	100.00%	3 (0.01%)	100.00%	1.00	00:00:00	0.00%	0 (0.00%)	$0.00 (0.00%)
855.	SonyEricsson ST18a Urushi	**3** **(0.00%)**	100.00%	3 (0.01%)	100.00%	1.00	00:00:00	0.00%	0 (0.00%)	$0.00 (0.00%)
856.	Telstra T-Hub2	**3** **(0.00%)**	100.00%	3 (0.01%)	33.33%	2.33	00:00:29	0.00%	0 (0.00%)	$0.00 (0.00%)
857.	Xiaomi MI 2SC	**3** **(0.00%)**	100.00%	3 (0.01%)	33.33%	2.33	00:00:37	0.00%	0 (0.00%)	$0.00 (0.00%)
858.	Xolo A800	**3** **(0.00%)**	66.67%	2 (0.00%)	66.67%	1.33	00:00:13	0.00%	0 (0.00%)	$0.00 (0.00%)
859.	Zopo ZP980	**3** **(0.00%)**	33.33%	1 (0.00%)	66.67%	1.33	00:00:48	0.00%	0 (0.00%)	$0.00 (0.00%)
860.	ZTE U795	**3** **(0.00%)**	33.33%	1 (0.00%)	33.33%	5.00	00:12:20	0.00%	0 (0.00%)	$0.00 (0.00%)
861.	ZTE Z990g Merit	**3** **(0.00%)**	100.00%	3 (0.01%)	66.67%	1.33	00:00:05	0.00%	0 (0.00%)	$0.00 (0.00%)
862.	2 Degrees C8660 Smart Touch	**2** **(0.00%)**	100.00%	2 (0.00%)	100.00%	1.00	00:00:00	0.00%	0 (0.00%)	$0.00 (0.00%)
863.	Acer A101 Vangogh	**2** **(0.00%)**	100.00%	2 (0.00%)	100.00%	1.00	00:00:00	0.00%	0 (0.00%)	$0.00 (0.00%)
864.	Acer A510	**2** **(0.00%)**	100.00%	2 (0.00%)	50.00%	1.50	00:00:15	0.00%	0 (0.00%)	$0.00 (0.00%)
865.	Acer A701	**2** **(0.00%)**	100.00%	2 (0.00%)	100.00%	1.00	00:00:00	0.00%	0 (0.00%)	$0.00 (0.00%)
866.	Acer E350 Liquid Gallant E350	**2** **(0.00%)**	100.00%	2 (0.00%)	100.00%	1.00	00:00:00	0.00%	0 (0.00%)	$0.00 (0.00%)
867.	Advan Vandroid T	**2** **(0.00%)**	50.00%	1 (0.00%)	100.00%	1.00	00:00:00	0.00%	0 (0.00%)	$0.00 (0.00%)
868.	Alcatel Evo 7 One Touch Evo 7	**2** **(0.00%)**	100.00%	2 (0.00%)	100.00%	1.00	00:00:00	0.00%	0 (0.00%)	$0.00 (0.00%)
869.	Alcatel One Touch 6033X Idol Ultra	**2** **(0.00%)**	100.00%	2 (0.00%)	100.00%	1.00	00:00:00	0.00%	0 (0.00%)	$0.00 (0.00%)
870.	Alcatel OT-4010X One Touch T'Pop 4010X	**2** **(0.00%)**	100.00%	2 (0.00%)	0.00%	4.00	00:02:01	0.00%	0 (0.00%)	$0.00 (0.00%)
871.	Alcatel OT-4033A Pop C3	**2** **(0.00%)**	100.00%	2 (0.00%)	100.00%	1.00	00:00:00	0.00%	0 (0.00%)	$0.00 (0.00%)
872.	Alcatel OT-5020X One Touch M'Pop	**2** **(0.00%)**	100.00%	2 (0.00%)	50.00%	1.50	00:01:38	0.00%	0 (0.00%)	$0.00 (0.00%)
873.	Alcatel OT-5021E One Touch Soleil	**2** **(0.00%)**	100.00%	2 (0.00%)	100.00%	1.00	00:00:00	0.00%	0 (0.00%)	$0.00 (0.00%)
874.	Alcatel OT-5035D One Touch X'Pop 5035D	**2** **(0.00%)**	100.00%	2 (0.00%)	50.00%	1.50	00:00:16	0.00%	0 (0.00%)	$0.00 (0.00%)
875.	Alcatel OT-5035X One Touch X'Pop 5035X	**2** **(0.00%)**	100.00%	2 (0.00%)	100.00%	1.00	00:00:00	0.00%	0 (0.00%)	$0.00 (0.00%)
876.	Alcatel OT-8000A One Touch Scribe Easy	**2** **(0.00%)**	100.00%	2 (0.00%)	0.00%	13.50	00:24:48	0.00%	0 (0.00%)	$0.00 (0.00%)
877.	Alcatel OT-8000D One Touch Scribe Easy	**2** **(0.00%)**	50.00%	1 (0.00%)	100.00%	1.00	00:00:00	0.00%	0 (0.00%)	$0.00 (0.00%)
878.	Alcatel OT-903 One Touch 903	**2** **(0.00%)**	100.00%	2 (0.00%)	50.00%	2.50	00:13:59	0.00%	0 (0.00%)	$0.00 (0.00%)
879.	Alcatel OT-918N One Touch 918N	**2** **(0.00%)**	100.00%	2 (0.00%)	100.00%	1.00	00:00:00	0.00%	0 (0.00%)	$0.00 (0.00%)
880.	Alcatel OT-990 One Touch 990	**2** **(0.00%)**	100.00%	2 (0.00%)	100.00%	1.00	00:00:00	0.00%	0 (0.00%)	$0.00 (0.00%)
[illegible]	Alcatel OT-991 [illegible]	[illegible]	[illegible]	[illegible]	[illegible]	[illegible]	[illegible]	[illegible]	[illegible]	[illegible]

881.	One Touch 991 Play	**2** **(0.00%)**	100.00%	2 (0.00%)	100.00%	1.00	00:00:00	0.00%	0 (0.00%)	$0.00 (0.00%)
882.	Alcatel OT-997D One Touch 997D	**2** **(0.00%)**	100.00%	2 (0.00%)	100.00%	1.00	00:00:00	0.00%	0 (0.00%)	$0.00 (0.00%)
883.	Archos 80 G9	**2** **(0.00%)**	100.00%	2 (0.00%)	100.00%	1.00	00:00:00	0.00%	0 (0.00%)	$0.00 (0.00%)
884.	Asus ASUS-PadFone	**2** **(0.00%)**	100.00%	2 (0.00%)	50.00%	2.00	00:00:22	0.00%	0 (0.00%)	$0.00 (0.00%)
885.	Asus TF300G Transformer Pad TF300G	**2** **(0.00%)**	50.00%	1 (0.00%)	100.00%	1.00	00:00:00	0.00%	0 (0.00%)	$0.00 (0.00%)
886.	BlackBerry 8900 Curve	**2** **(0.00%)**	100.00%	2 (0.00%)	100.00%	1.00	00:00:00	0.00%	0 (0.00%)	$0.00 (0.00%)
887.	BlackBerry 9100 Pearl 3G	**2** **(0.00%)**	50.00%	1 (0.00%)	100.00%	1.00	00:00:00	0.00%	0 (0.00%)	$0.00 (0.00%)
888.	BlackBerry 9330 Curve	**2** **(0.00%)**	100.00%	2 (0.00%)	100.00%	1.00	00:00:00	0.00%	0 (0.00%)	$0.00 (0.00%)
889.	BlackBerry 9670	**2** **(0.00%)**	100.00%	2 (0.00%)	50.00%	2.00	00:01:20	0.00%	0 (0.00%)	$0.00 (0.00%)
890.	Casio IS11CA G'z One IS11CA for KDDI au	**2** **(0.00%)**	50.00%	1 (0.00%)	100.00%	1.00	00:00:00	0.00%	0 (0.00%)	$0.00 (0.00%)
891.	Coby MID7048 Kyros MID7048	**2** **(0.00%)**	100.00%	2 (0.00%)	100.00%	1.00	00:00:00	0.00%	0 (0.00%)	$0.00 (0.00%)
892.	Digital2 D2-927G Premier	**2** **(0.00%)**	0.00%	0 (0.00%)	50.00%	1.50	00:02:05	0.00%	0 (0.00%)	$0.00 (0.00%)
893.	DoCoMo F-04E Arrows V F-04E	**2** **(0.00%)**	50.00%	1 (0.00%)	100.00%	1.00	00:00:00	0.00%	0 (0.00%)	$0.00 (0.00%)
894.	DoCoMo L-05D Optimus it	**2** **(0.00%)**	100.00%	2 (0.00%)	100.00%	1.00	00:00:00	0.00%	0 (0.00%)	$0.00 (0.00%)
895.	DoCoMo SC-06D Galaxy SIII LTE	**2** **(0.00%)**	100.00%	2 (0.00%)	50.00%	3.00	00:02:15	0.00%	0 (0.00%)	$0.00 (0.00%)
896.	Fujitsu F-01D ARROWS Tab LTE F-01D	**2** **(0.00%)**	100.00%	2 (0.00%)	100.00%	1.00	00:00:00	0.00%	0 (0.00%)	$0.00 (0.00%)
897.	Fujitsu F-05D ARROWS X LTE F-05D	**2** **(0.00%)**	100.00%	2 (0.00%)	50.00%	5.00	00:00:47	0.00%	0 (0.00%)	$0.00 (0.00%)
898.	Garmin-Asus A1(	**2** **(0.00%)**	50.00%	1 (0.00%)	50.00%	1.50	00:03:02	0.00%	0 (0.00%)	$0.00 (0.00%)
899.	HCL U1 ME U1	**2** **(0.00%)**	100.00%	2 (0.00%)	0.00%	2.00	00:04:58	0.00%	0 (0.00%)	$0.00 (0.00%)
900.	HTC A02 INFOBAR A02 for KDDI	**2** **(0.00%)**	100.00%	2 (0.00%)	50.00%	4.00	00:00:25	0.00%	0 (0.00%)	$0.00 (0.00%)
901.	HTC A620t 8S	**2** **(0.00%)**	100.00%	2 (0.00%)	100.00%	1.00	00:00:00	0.00%	0 (0.00%)	$0.00 (0.00%)
902.	HTC Corporation PJ83100 One X	**2** **(0.00%)**	100.00%	2 (0.00%)	100.00%	1.00	00:00:00	0.00%	0 (0.00%)	$0.00 (0.00%)
903.	HTC Desire X0H6T	**2** **(0.00%)**	100.00%	2 (0.00%)	50.00%	2.00	00:00:45	0.00%	0 (0.00%)	$0.00 (0.00%)
904.	HTC HD2	**2** **(0.00%)**	100.00%	2 (0.00%)	100.00%	1.00	00:00:00	0.00%	0 (0.00%)	$0.00 (0.00%)
905.	HTC HTC6500LVW One	**2** **(0.00%)**	100.00%	2 (0.00%)	0.00%	6.00	00:00:45	0.00%	0 (0.00%)	$0.00 (0.00%)
906.	HTC HTL21 Butterfly	**2** **(0.00%)**	100.00%	2 (0.00%)	100.00%	1.00	00:00:00	0.00%	0 (0.00%)	$0.00 (0.00%)
907.	HTC PC36100 EVO 4G	**2** **(0.00%)**	100.00%	2 (0.00%)	100.00%	1.00	00:00:00	0.00%	0 (0.00%)	$0.00 (0.00%)
908.	HTC PM63100 One X+	**2** **(0.00%)**	50.00%	1 (0.00%)	100.00%	1.00	00:00:00	0.00%	0 (0.00%)	$0.00 (0.00%)
909.	HTC Radar 4G	**2** **(0.00%)**	100.00%	2 (0.00%)	100.00%	1.00	00:00:00	0.00%	0 (0.00%)	$0.00 (0.00%)
910.	HTC Salsa	**2** **(0.00%)**	100.00%	2 (0.00%)	100.00%	1.00	00:00:00	0.00%	0 (0.00%)	$0.00 (0.00%)

911.	HTC V1 Me V1	**2** **(0.00%)**	100.00%	2 (0.00%)	50.00%	1.50	00:00:16	0.00%	0 (0.00%)	$0.00 (0.00%)
912.	HTC Vision	**2** **(0.00%)**	100.00%	2 (0.00%)	100.00%	1.00	00:00:00	0.00%	0 (0.00%)	$0.00 (0.00%)
913.	HTC Z710a Sensation	**2** **(0.00%)**	50.00%	1 (0.00%)	50.00%	3.00	00:00:17	0.00%	0 (0.00%)	$0.00 (0.00%)
914.	Huawei G525-U00 Ascend G525-U00	**2** **(0.00%)**	100.00%	2 (0.00%)	100.00%	1.00	00:00:00	0.00%	0 (0.00%)	$0.00 (0.00%)
915.	Huawei G610-U00	**2** **(0.00%)**	100.00%	2 (0.00%)	50.00%	7.50	00:21:55	0.00%	0 (0.00%)	$0.00 (0.00%)
916.	Huawei G700-U10 Ascend G700	**2** **(0.00%)**	100.00%	2 (0.00%)	100.00%	1.00	00:00:00	0.00%	0 (0.00%)	$0.00 (0.00%)
917.	Huawei G730-U00 Ascend G730	**2** **(0.00%)**	100.00%	2 (0.00%)	50.00%	3.00	00:01:00	0.00%	0 (0.00%)	$0.00 (0.00%)
918.	Huawei H866C Ascend Y H866C	**2** **(0.00%)**	100.00%	2 (0.00%)	50.00%	1.50	00:00:30	0.00%	0 (0.00%)	$0.00 (0.00%)
919.	Huawei HUAWEI Y210-0200 Ascend	**2** **(0.00%)**	50.00%	1 (0.00%)	100.00%	1.00	00:00:00	0.00%	0 (0.00%)	$0.00 (0.00%)
920.	Huawei U8100 Tactile Internet	**2** **(0.00%)**	100.00%	2 (0.00%)	50.00%	1.50	00:00:22	0.00%	0 (0.00%)	$0.00 (0.00%)
921.	Huawei U8180	**2** **(0.00%)**	100.00%	2 (0.00%)	50.00%	2.00	00:02:21	0.00%	0 (0.00%)	$0.00 (0.00%)
922.	Huawei U8666E-51 Ascend Y201 Pro	**2** **(0.00%)**	100.00%	2 (0.00%)	100.00%	1.00	00:00:00	0.00%	0 (0.00%)	$0.00 (0.00%)
923.	Huawei U8820 Titan	**2** **(0.00%)**	100.00%	2 (0.00%)	50.00%	3.50	00:01:07	0.00%	0 (0.00%)	$0.00 (0.00%)
924.	Huawei U8825-1 Ascend G330	**2** **(0.00%)**	100.00%	2 (0.00%)	100.00%	1.00	00:00:00	0.00%	0 (0.00%)	$0.00 (0.00%)
925.	Huawei U8950D Ascend G600	**2** **(0.00%)**	100.00%	2 (0.00%)	50.00%	2.00	00:00:52	0.00%	0 (0.00%)	$0.00 (0.00%)
926.	Idea AURUS III	**2** **(0.00%)**	50.00%	1 (0.00%)	50.00%	4.00	00:01:44	0.00%	0 (0.00%)	$0.00 (0.00%)
927.	Jiayu JY-G3	**2** **(0.00%)**	100.00%	2 (0.00%)	100.00%	1.00	00:00:00	0.00%	0 (0.00%)	$0.00 (0.00%)
928.	Karbonn A15	**2** **(0.00%)**	100.00%	2 (0.00%)	100.00%	1.00	00:00:00	0.00%	0 (0.00%)	$0.00 (0.00%)
929.	Karbonn A4+	**2** **(0.00%)**	100.00%	2 (0.00%)	50.00%	1.50	00:00:13	0.00%	0 (0.00%)	$0.00 (0.00%)
930.	Karbonn A6	**2** **(0.00%)**	100.00%	2 (0.00%)	50.00%	2.50	00:00:52	0.00%	0 (0.00%)	$0.00 (0.00%)
931.	Karbonn A7 A7 Star	**2** **(0.00%)**	50.00%	1 (0.00%)	50.00%	2.00	00:00:23	0.00%	0 (0.00%)	$0.00 (0.00%)
932.	KDDI SOL22 Xperia UL	**2** **(0.00%)**	100.00%	2 (0.00%)	100.00%	1.00	00:00:00	0.00%	0 (0.00%)	$0.00 (0.00%)
933.	Kobo Touch Touch eReader	**2** **(0.00%)**	100.00%	2 (0.00%)	100.00%	1.00	00:00:00	0.00%	0 (0.00%)	$0.00 (0.00%)
934.	Kyocera C5171 Hydro	**2** **(0.00%)**	100.00%	2 (0.00%)	0.00%	7.00	00:14:31	0.00%	0 (0.00%)	$0.00 (0.00%)
935.	Lanix S105 Ilium	**2** **(0.00%)**	100.00%	2 (0.00%)	0.00%	4.00	00:02:38	0.00%	0 (0.00%)	$0.00 (0.00%)
936.	Lenovo B6000-H Yoga B6000-H	**2** **(0.00%)**	50.00%	1 (0.00%)	100.00%	1.00	00:00:00	0.00%	0 (0.00%)	$0.00 (0.00%)
937.	Lenovo K1	**2** **(0.00%)**	100.00%	2 (0.00%)	50.00%	1.50	00:10:25	0.00%	0 (0.00%)	$0.00 (0.00%)
938.	Lenovo S880	**2** **(0.00%)**	100.00%	2 (0.00%)	100.00%	1.00	00:00:00	0.00%	0 (0.00%)	$0.00 (0.00%)
939.	LG C900 Quantum	**2** **(0.00%)**	100.00%	2 (0.00%)	100.00%	1.00	00:00:00	0.00%	0 (0.00%)	$0.00 (0.00%)
940.	LG E400R Optimus L3	**2** **(0.00%)**	100.00%	2 (0.00%)	100.00%	1.00	00:00:00	0.00%	0 (0.00%)	$0.00 (0.00%)
941.	LG E440 Optimus L4 II	**2** **(0.00%)**	100.00%	2 (0.00%)	100.00%	1.00	00:00:00	0.00%	0 (0.00%)	$0.00 (0.00%)
942.	LG E455f	**2** **(0.00%)**	100.00%	2 (0.00%)	50.00%	3.50	00:06:24	0.00%	0 (0.00%)	$0.00 (0.00%)

	Optimus L5 II									
943.	LG E510g Optimus Hub	**2** **(0.00%)**	100.00%	2 (0.00%)	50.00%	1.50	00:01:09	0.00%	0 (0.00%)	$0.00 (0.00%)
944.	LG E976 Optimus G	**2** **(0.00%)**	50.00%	1 (0.00%)	50.00%	6.00	00:02:22	0.00%	0 (0.00%)	$0.00 (0.00%)
945.	LG E988 Optimus G Pro	**2** **(0.00%)**	100.00%	2 (0.00%)	50.00%	2.00	00:00:18	0.00%	0 (0.00%)	$0.00 (0.00%)
946.	LG F120K Optimus LTE TAG	**2** **(0.00%)**	50.00%	1 (0.00%)	50.00%	2.00	00:00:49	0.00%	0 (0.00%)	$0.00 (0.00%)
947.	LG F180L Optimus G	**2** **(0.00%)**	100.00%	2 (0.00%)	50.00%	1.50	00:00:23	0.00%	0 (0.00%)	$0.00 (0.00%)
948.	LG F240S Optimus G Pro	**2** **(0.00%)**	100.00%	2 (0.00%)	100.00%	1.00	00:00:00	0.00%	0 (0.00%)	$0.00 (0.00%)
949.	LG F260S Optimus LTE III	**2** **(0.00%)**	100.00%	2 (0.00%)	100.00%	1.00	00:00:00	0.00%	0 (0.00%)	$0.00 (0.00%)
950.	LG GM360	**2** **(0.00%)**	100.00%	2 (0.00%)	50.00%	1.50	00:00:12	0.00%	0 (0.00%)	$0.00 (0.00%)
951.	LG GT540 GT540 Optimus	**2** **(0.00%)**	100.00%	2 (0.00%)	100.00%	1.00	00:00:00	0.00%	0 (0.00%)	$0.00 (0.00%)
952.	LG KP500	**2** **(0.00%)**	100.00%	2 (0.00%)	100.00%	1.00	00:00:00	0.00%	0 (0.00%)	$0.00 (0.00%)
953.	LG L55c Optimus Q	**2** **(0.00%)**	50.00%	1 (0.00%)	100.00%	1.00	00:00:00	0.00%	0 (0.00%)	$0.00 (0.00%)
954.	LG LG855 Marquee	**2** **(0.00%)**	100.00%	2 (0.00%)	50.00%	2.50	00:00:52	0.00%	0 (0.00%)	$0.00 (0.00%)
955.	LG LS970 Optimus G	**2** **(0.00%)**	100.00%	2 (0.00%)	100.00%	1.00	00:00:00	0.00%	0 (0.00%)	$0.00 (0.00%)
956.	LG MS690 Optimus M	**2** **(0.00%)**	100.00%	2 (0.00%)	100.00%	1.00	00:00:00	0.00%	0 (0.00%)	$0.00 (0.00%)
957.	LG MS695 Optimus M+	**2** **(0.00%)**	100.00%	2 (0.00%)	100.00%	1.00	00:00:00	0.00%	0 (0.00%)	$0.00 (0.00%)
958.	LG P500 Optimus One	**2** **(0.00%)**	50.00%	1 (0.00%)	50.00%	3.00	00:03:24	0.00%	0 (0.00%)	$0.00 (0.00%)
959.	LG P509 Optimus T	**2** **(0.00%)**	100.00%	2 (0.00%)	50.00%	4.50	00:01:13	0.00%	0 (0.00%)	$0.00 (0.00%)
960.	LG P708g Optimus L7	**2** **(0.00%)**	100.00%	2 (0.00%)	50.00%	2.00	00:00:26	0.00%	0 (0.00%)	$0.00 (0.00%)
961.	LG P710 Optimus L7 II	**2** **(0.00%)**	100.00%	2 (0.00%)	50.00%	3.50	00:01:37	0.00%	0 (0.00%)	$0.00 (0.00%)
962.	LG P715 Optimus L7 II Dual	**2** **(0.00%)**	100.00%	2 (0.00%)	50.00%	1.50	00:03:02	0.00%	0 (0.00%)	$0.00 (0.00%)
963.	LG P930 Optimus LTE	**2** **(0.00%)**	100.00%	2 (0.00%)	100.00%	1.00	00:00:00	0.00%	0 (0.00%)	$0.00 (0.00%)
964.	LG SU760 Optimus 3D	**2** **(0.00%)**	100.00%	2 (0.00%)	0.00%	13.00	00:02:42	0.00%	0 (0.00%)	$0.00 (0.00%)
965.	LG V909 G Slate	**2** **(0.00%)**	100.00%	2 (0.00%)	50.00%	1.50	00:03:49	0.00%	0 (0.00%)	$0.00 (0.00%)
966.	LG VS930 Spectrum 2	**2** **(0.00%)**	100.00%	2 (0.00%)	100.00%	1.00	00:00:00	0.00%	0 (0.00%)	$0.00 (0.00%)
967.	Medion E10312 (MD 98486) Lifetab	**2** **(0.00%)**	0.00%	0 (0.00%)	0.00%	3.50	00:02:51	0.00%	0 (0.00%)	$0.00 (0.00%)
968.	Medion MD 98172 Life E3501	**2** **(0.00%)**	100.00%	2 (0.00%)	50.00%	12.50	00:03:28	0.00%	0 (0.00%)	$0.00 (0.00%)
969.	Meizu M040 MX2	**2** **(0.00%)**	100.00%	2 (0.00%)	100.00%	1.00	00:00:00	0.00%	0 (0.00%)	$0.00 (0.00%)
970.	Meizu M9	**2** **(0.00%)**	100.00%	2 (0.00%)	100.00%	1.00	00:00:00	0.00%	0 (0.00%)	$0.00 (0.00%)
971.	Micromax A26 A26 Bolt	**2** **(0.00%)**	100.00%	2 (0.00%)	50.00%	1.50	00:00:06	0.00%	0 (0.00%)	$0.00 (0.00%)
972.	Micromax A72 A72 Canvas Viva	**2** **(0.00%)**	100.00%	2 (0.00%)	50.00%	2.00	00:01:10	0.00%	0 (0.00%)	$0.00 (0.00%)

973.	Micromax A89 A89 Ninja	2	(0.00%)	100.00%	2	(0.00%)	100.00%	1.00	00:00:00	0.00%	0	(0.00%)	$0.00	(0.00%)
974.	Motorola Electrify	2	(0.00%)	50.00%	1	(0.00%)	50.00%	7.50	00:05:01	0.00%	0	(0.00%)	$0.00	(0.00%)
975.	Motorola ME525 Defy	2	(0.00%)	100.00%	2	(0.00%)	100.00%	1.00	00:00:00	0.00%	0	(0.00%)	$0.00	(0.00%)
976.	Motorola MotoXT860	2	(0.00%)	100.00%	2	(0.00%)	50.00%	12.00	00:21:31	0.00%	0	(0.00%)	$0.00	(0.00%)
977.	Motorola MT788 RAZR i	2	(0.00%)	100.00%	2	(0.00%)	100.00%	1.00	00:00:00	0.00%	0	(0.00%)	$0.00	(0.00%)
978.	Motorola MZ609 4G Droid XYBoard 8.2 4G	2	(0.00%)	100.00%	2	(0.00%)	50.00%	1.50	00:02:33	0.00%	0	(0.00%)	$0.00	(0.00%)
979.	Motorola MZ609 XOOM 2 ME	2	(0.00%)	100.00%	2	(0.00%)	50.00%	2.00	00:00:09	0.00%	0	(0.00%)	$0.00	(0.00%)
980.	Motorola MZ617 Droid XYBoard 10.1	2	(0.00%)	100.00%	2	(0.00%)	50.00%	2.00	00:00:13	0.00%	0	(0.00%)	$0.00	(0.00%)
981.	Motorola XT1056 Moto X	2	(0.00%)	100.00%	2	(0.00%)	50.00%	1.50	00:00:30	0.00%	0	(0.00%)	$0.00	(0.00%)
982.	Motorola XT390	2	(0.00%)	50.00%	1	(0.00%)	50.00%	6.50	00:08:46	0.00%	0	(0.00%)	$0.00	(0.00%)
983.	Motorola XT605 Jorian	2	(0.00%)	100.00%	2	(0.00%)	50.00%	1.50	00:00:07	0.00%	0	(0.00%)	$0.00	(0.00%)
984.	Motorola XT901 Electrify M	2	(0.00%)	100.00%	2	(0.00%)	50.00%	2.00	00:00:27	0.00%	0	(0.00%)	$0.00	(0.00%)
985.	Motorola XT915	2	(0.00%)	100.00%	2	(0.00%)	100.00%	1.00	00:00:00	0.00%	0	(0.00%)	$0.00	(0.00%)
986.	NEC N-01D MEDIAS PP N-01D for DoCoMo	2	(0.00%)	50.00%	1	(0.00%)	50.00%	3.00	00:01:24	0.00%	0	(0.00%)	$0.00	(0.00%)
987.	Nintendo 3DS	2	(0.00%)	100.00%	2	(0.00%)	50.00%	8.50	00:06:38	0.00%	0	(0.00%)	$0.00	(0.00%)
988.	Nokia 111	2	(0.00%)	100.00%	2	(0.00%)	100.00%	1.00	00:00:00	0.00%	0	(0.00%)	$0.00	(0.00%)
989.	Nokia 205.1 Asha 205	2	(0.00%)	100.00%	2	(0.00%)	50.00%	1.50	00:00:28	0.00%	0	(0.00%)	$0.00	(0.00%)
990.	Nokia 300 Nokia Asha 300	2	(0.00%)	100.00%	2	(0.00%)	100.00%	1.00	00:00:00	0.00%	0	(0.00%)	$0.00	(0.00%)
991.	Nokia 5235	2	(0.00%)	100.00%	2	(0.00%)	50.00%	1.50	00:03:02	0.00%	0	(0.00%)	$0.00	(0.00%)
992.	Nokia 6303 Classic	2	(0.00%)	100.00%	2	(0.00%)	100.00%	1.00	00:00:00	0.00%	0	(0.00%)	$0.00	(0.00%)
993.	Nokia 6303i classic Classic	2	(0.00%)	100.00%	2	(0.00%)	100.00%	1.00	00:00:00	0.00%	0	(0.00%)	$0.00	(0.00%)
994.	Nokia 700	2	(0.00%)	100.00%	2	(0.00%)	100.00%	1.00	00:00:00	0.00%	0	(0.00%)	$0.00	(0.00%)
995.	Nokia 701	2	(0.00%)	100.00%	2	(0.00%)	50.00%	1.50	00:01:00	0.00%	0	(0.00%)	$0.00	(0.00%)
996.	Nokia Asha 502	2	(0.00%)	100.00%	2	(0.00%)	100.00%	1.00	00:00:00	0.00%	0	(0.00%)	$0.00	(0.00%)
997.	Nokia C3-01	2	(0.00%)	100.00%	2	(0.00%)	100.00%	1.00	00:00:00	0.00%	0	(0.00%)	$0.00	(0.00%)
998.	Nokia C5-05	2	(0.00%)	100.00%	2	(0.00%)	50.00%	2.50	00:02:54	0.00%	0	(0.00%)	$0.00	(0.00%)
999.	Nokia E6-00 E6	2	(0.00%)	100.00%	2	(0.00%)	0.00%	4.50	00:11:24	0.00%	0	(0.00%)	$0.00	(0.00%)
1000.	Nokia Lumia 525	2	(0.00%)	100.00%	2	(0.00%)	0.00%	4.50	00:10:14	0.00%	0	(0.00%)	$0.00	(0.00%)
1001.	Nokia Lumia 810	2	(0.00%)	100.00%	2	(0.00%)	100.00%	1.00	00:00:00	0.00%	0	(0.00%)	$0.00	(0.00%)
1002.	Nokia N97	2	(0.00%)	100.00%	2	(0.00%)	100.00%	1.00	00:00:00	0.00%	0	(0.00%)	$0.00	(0.00%)
1003.	Nokia N97 mini N97 mini	2	(0.00%)	100.00%	2	(0.00%)	50.00%	1.50	00:00:44	0.00%	0	(0.00%)	$0.00	(0.00%)
1004.	Nokia X2-05	2	(0.00%)	100.00%	2	(0.00%)	50.00%	1.50	00:00:33	0.00%	0	(0.00%)	$0.00	(0.00%)
1005.	Oppo X909 Find5 X909	2	(0.00%)	100.00%	2	(0.00%)	100.00%	1.00	00:00:00	0.00%	0	(0.00%)	$0.00	(0.00%)

1006.	Panasonic 003P Sweety 003P for Softbank	**2**	**(0.00%)**	50.00%	1	(0.00%)	100.00%	1.00	00:00:00	0.00%	0	(0.00%)	$0.00	(0.00%)
1007.	Pandigital SuperNova	**2**	**(0.00%)**	100.00%	2	(0.00%)	50.00%	2.00	00:00:22	0.00%	0	(0.00%)	$0.00	(0.00%)
1008.	Pantech IM-A820L Vega LTE EX	**2**	**(0.00%)**	100.00%	2	(0.00%)	100.00%	1.00	00:00:00	0.00%	0	(0.00%)	$0.00	(0.00%)
1009.	Pantech IM-A850L Vega R3	**2**	**(0.00%)**	100.00%	2	(0.00%)	100.00%	1.00	00:00:00	0.00%	0	(0.00%)	$0.00	(0.00%)
1010.	Pantech IM-T100K Vega No 5	**2**	**(0.00%)**	100.00%	2	(0.00%)	50.00%	5.00	00:01:37	0.00%	0	(0.00%)	$0.00	(0.00%)
1011.	Philips W732	**2**	**(0.00%)**	100.00%	2	(0.00%)	100.00%	1.00	00:00:00	0.00%	0	(0.00%)	$0.00	(0.00%)
1012.	Samsung GT i7500 Galaxy	**2**	**(0.00%)**	100.00%	2	(0.00%)	0.00%	9.00	00:14:12	0.00%	0	(0.00%)	$0.00	(0.00%)
1013.	Samsung GT-B7510L Galaxy Pro	**2**	**(0.00%)**	50.00%	1	(0.00%)	50.00%	4.00	00:03:47	0.00%	0	(0.00%)	$0.00	(0.00%)
1014.	Samsung GT-C3303	**2**	**(0.00%)**	100.00%	2	(0.00%)	100.00%	1.00	00:00:00	0.00%	0	(0.00%)	$0.00	(0.00%)
1015.	Samsung GT-C3322 Metro Duos	**2**	**(0.00%)**	100.00%	2	(0.00%)	50.00%	1.50	00:00:01	0.00%	0	(0.00%)	$0.00	(0.00%)
1016.	Samsung GT-E1130B	**2**	**(0.00%)**	50.00%	1	(0.00%)	0.00%	3.00	00:01:04	0.00%	0	(0.00%)	$0.00	(0.00%)
1017.	Samsung GT-E1390	**2**	**(0.00%)**	100.00%	2	(0.00%)	50.00%	2.50	00:07:53	0.00%	0	(0.00%)	$0.00	(0.00%)
1018.	Samsung GT-E3309 Manhattan	**2**	**(0.00%)**	100.00%	2	(0.00%)	50.00%	2.50	00:03:32	0.00%	0	(0.00%)	$0.00	(0.00%)
1019.	Samsung GT-I5800 Galaxy 3 Apollo	**2**	**(0.00%)**	100.00%	2	(0.00%)	100.00%	1.00	00:00:00	0.00%	0	(0.00%)	$0.00	(0.00%)
1020.	Samsung GT-N7108 Galaxy Note II	**2**	**(0.00%)**	100.00%	2	(0.00%)	100.00%	1.00	00:00:00	0.00%	0	(0.00%)	$0.00	(0.00%)
1021.	Samsung GT-P7320 Galaxy Tab 8.9 LTE	**2**	**(0.00%)**	100.00%	2	(0.00%)	100.00%	1.00	00:00:00	0.00%	0	(0.00%)	$0.00	(0.00%)
1022.	Samsung GT-P7511 Galaxy Tab 10.1	**2**	**(0.00%)**	100.00%	2	(0.00%)	100.00%	1.00	00:00:00	0.00%	0	(0.00%)	$0.00	(0.00%)
1023.	Samsung GT-S5300L Galaxy Pocket	**2**	**(0.00%)**	100.00%	2	(0.00%)	50.00%	8.00	00:11:08	0.00%	0	(0.00%)	$0.00	(0.00%)
1024.	Samsung GT-S5333 Samsung Wave 533	**2**	**(0.00%)**	100.00%	2	(0.00%)	100.00%	1.00	00:00:00	0.00%	0	(0.00%)	$0.00	(0.00%)
1025.	Samsung GT-S5570B	**2**	**(0.00%)**	100.00%	2	(0.00%)	50.00%	3.50	00:02:59	0.00%	0	(0.00%)	$0.00	(0.00%)
1026.	Samsung GT-S5750E	**2**	**(0.00%)**	50.00%	1	(0.00%)	50.00%	1.50	00:00:33	0.00%	0	(0.00%)	$0.00	(0.00%)
1027.	Samsung GT-S5830C Galaxy Ace	**2**	**(0.00%)**	100.00%	2	(0.00%)	100.00%	1.00	00:00:00	0.00%	0	(0.00%)	$0.00	(0.00%)
1028.	Samsung GT-S7572 Galaxy Trend II Duos	**2**	**(0.00%)**	100.00%	2	(0.00%)	50.00%	1.50	00:00:03	0.00%	0	(0.00%)	$0.00	(0.00%)
1029.	Samsung SC-02B Galaxy S SC-02B for DoCoMo	**2**	**(0.00%)**	100.00%	2	(0.00%)	100.00%	1.00	00:00:00	0.00%	0	(0.00%)	$0.00	(0.00%)
1030.	Samsung SC-03D GALAXY S II LTE SC-03D for	**2**	**(0.00%)**	100.00%	2	(0.00%)	100.00%	1.00	00:00:00	0.00%	0	(0.00%)	$0.00	(0.00%)

	DoCoMo									
1031.	Samsung SCH-I400 Continuum	**2** **(0.00%)**	50.00%	1 (0.00%)	50.00%	1.50	00:00:13	0.00%	0 (0.00%)	$0.00 (0.00%)
1032.	Samsung SCH-I435 Galaxy S4 Mini	**2** **(0.00%)**	100.00%	2 (0.00%)	100.00%	1.00	00:00:00	0.00%	0 (0.00%)	$0.00 (0.00%)
1033.	Samsung SCH-I510 Droid Charge	**2** **(0.00%)**	100.00%	2 (0.00%)	50.00%	5.50	00:04:24	0.00%	0 (0.00%)	$0.00 (0.00%)
1034.	Samsung SCH-i699 Galaxy Trend	**2** **(0.00%)**	50.00%	1 (0.00%)	100.00%	1.00	00:00:00	0.00%	0 (0.00%)	$0.00 (0.00%)
1035.	Samsung SCH-R530C Galaxy S III	**2** **(0.00%)**	100.00%	2 (0.00%)	100.00%	1.00	00:00:00	0.00%	0 (0.00%)	$0.00 (0.00%)
1036.	Samsung SCH-R950 Galaxy Note II	**2** **(0.00%)**	100.00%	2 (0.00%)	50.00%	2.00	00:00:45	0.00%	0 (0.00%)	$0.00 (0.00%)
1037.	Samsung SCH-R960 Galaxy Mega 6.3	**2** **(0.00%)**	50.00%	1 (0.00%)	50.00%	2.00	00:00:11	0.00%	0 (0.00%)	$0.00 (0.00%)
1038.	Samsung SGH-A927 Flight II	**2** **(0.00%)**	100.00%	2 (0.00%)	100.00%	1.00	00:00:00	0.00%	0 (0.00%)	$0.00 (0.00%)
1039.	Samsung SGH-I467 Galaxy Note 8.0	**2** **(0.00%)**	100.00%	2 (0.00%)	100.00%	1.00	00:00:00	0.00%	0 (0.00%)	$0.00 (0.00%)
1040.	Samsung SGH-I527 Galaxy Mega 6.3	**2** **(0.00%)**	100.00%	2 (0.00%)	100.00%	1.00	00:00:00	0.00%	0 (0.00%)	$0.00 (0.00%)
1041.	Samsung SGH-i717R Galaxy Note	**2** **(0.00%)**	50.00%	1 (0.00%)	50.00%	1.50	00:03:32	0.00%	0 (0.00%)	$0.00 (0.00%)
1042.	Samsung SGH-S730M Galaxy Discover	**2** **(0.00%)**	100.00%	2 (0.00%)	100.00%	1.00	00:00:00	0.00%	0 (0.00%)	$0.00 (0.00%)
1043.	Samsung SGH-T589 Gravity Smart	**2** **(0.00%)**	100.00%	2 (0.00%)	50.00%	1.50	00:00:13	0.00%	0 (0.00%)	$0.00 (0.00%)
1044.	Samsung SGH-T839 T-Mobile Sidekick 4G	**2** **(0.00%)**	100.00%	2 (0.00%)	50.00%	7.00	00:02:49	0.00%	0 (0.00%)	$0.00 (0.00%)
1045.	Samsung SGH-T879 Galaxy Note	**2** **(0.00%)**	100.00%	2 (0.00%)	50.00%	2.00	00:00:06	0.00%	0 (0.00%)	$0.00 (0.00%)
1046.	Samsung SHV-E210L Galaxy S III	**2** **(0.00%)**	100.00%	2 (0.00%)	100.00%	1.00	00:00:00	0.00%	0 (0.00%)	$0.00 (0.00%)
1047.	Samsung SHV-E210S Galaxy SIII	**2** **(0.00%)**	100.00%	2 (0.00%)	50.00%	1.50	00:10:52	0.00%	0 (0.00%)	$0.00 (0.00%)
1048.	Samsung SHV-E300L Galaxy S IV	**2** **(0.00%)**	100.00%	2 (0.00%)	100.00%	1.00	00:00:00	0.00%	0 (0.00%)	$0.00 (0.00%)
1049.	Samsung SHV-E330L Galaxy S IV	**2** **(0.00%)**	100.00%	2 (0.00%)	100.00%	1.00	00:00:00	0.00%	0 (0.00%)	$0.00 (0.00%)
1050.	Samsung SHV-E370K Galaxy S4 Mini	**2** **(0.00%)**	50.00%	1 (0.00%)	50.00%	2.00	00:00:41	0.00%	0 (0.00%)	$0.00 (0.00%)
1051.	Samsung SHW-M250K GALAXY S II (KT)	**2** **(0.00%)**	100.00%	2 (0.00%)	100.00%	1.00	00:00:00	0.00%	0 (0.00%)	$0.00 (0.00%)
1052.	Samsung SM-G7102 Galaxy Grand 2	**2** **(0.00%)**	100.00%	2 (0.00%)	100.00%	1.00	00:00:00	0.00%	0 (0.00%)	$0.00 (0.00%)
	Samsung SM-G900F Galaxy									

1053.	S5	**2** **(0.00%)**	100.00%	2 (0.00%)	100.00%	1.00	00:00:00	0.00%	0 (0.00%)	$0.00 (0.00%)
1054.	Samsung SPH-930 Transform Ultra	**2** **(0.00%)**	100.00%	2 (0.00%)	100.00%	1.00	00:00:00	0.00%	0 (0.00%)	$0.00 (0.00%)
1055.	Samsung SPH-P500 Galaxy Tab 2 10.1	**2** **(0.00%)**	100.00%	2 (0.00%)	50.00%	2.00	00:00:29	0.00%	0 (0.00%)	$0.00 (0.00%)
1056.	Sharp 005SH Sharp Galapagos 005SH for SoftBank	**2** **(0.00%)**	50.00%	1 (0.00%)	0.00%	45.00	00:31:29	0.00%	0 (0.00%)	$0.00 (0.00%)
1057.	Sharp SH-03C LYNX 3D SH-03C for DoCoMo	**2** **(0.00%)**	50.00%	1 (0.00%)	100.00%	1.00	00:00:00	0.00%	0 (0.00%)	$0.00 (0.00%)
1058.	Sharp SHL23 AQUOS PHONE SERIE SHL23 for KDDI	**2** **(0.00%)**	50.00%	1 (0.00%)	50.00%	2.00	00:00:10	0.00%	0 (0.00%)	$0.00 (0.00%)
1059.	Smartfren AD683G Andromax-i AD683G	**2** **(0.00%)**	50.00%	1 (0.00%)	50.00%	3.50	00:01:21	0.00%	0 (0.00%)	$0.00 (0.00%)
1060.	Smartfren E860 Hisense E860	**2** **(0.00%)**	50.00%	1 (0.00%)	50.00%	4.00	00:01:28	0.00%	0 (0.00%)	$0.00 (0.00%)
1061.	Sony C5306 Xperia SP LTE	**2** **(0.00%)**	100.00%	2 (0.00%)	50.00%	3.00	00:00:21	0.00%	0 (0.00%)	$0.00 (0.00%)
1062.	Sony C5502 Xperia ZR	**2** **(0.00%)**	100.00%	2 (0.00%)	100.00%	1.00	00:00:00	0.00%	0 (0.00%)	$0.00 (0.00%)
1063.	Sony C6802 Xperia Z Ultra	**2** **(0.00%)**	100.00%	2 (0.00%)	100.00%	1.00	00:00:00	0.00%	0 (0.00%)	$0.00 (0.00%)
1064.	Sony C6943 Xperia Z1	**2** **(0.00%)**	50.00%	1 (0.00%)	50.00%	6.00	00:05:11	0.00%	0 (0.00%)	$0.00 (0.00%)
1065.	Sony D5503 Xperia Z1s	**2** **(0.00%)**	100.00%	2 (0.00%)	100.00%	1.00	00:00:00	0.00%	0 (0.00%)	$0.00 (0.00%)
1066.	Sony LT29i Xperia GX	**2** **(0.00%)**	100.00%	2 (0.00%)	100.00%	1.00	00:00:00	0.00%	0 (0.00%)	$0.00 (0.00%)
1067.	Sony LT30a Xperia TL	**2** **(0.00%)**	100.00%	2 (0.00%)	100.00%	1.00	00:00:00	0.00%	0 (0.00%)	$0.00 (0.00%)
1068.	Sony PlayStation Vita	**2** **(0.00%)**	100.00%	2 (0.00%)	50.00%	1.50	00:03:29	0.00%	0 (0.00%)	$0.00 (0.00%)
1069.	Sony SO-02E Xperia Z SO-02E for DoCoMo	**2** **(0.00%)**	100.00%	2 (0.00%)	100.00%	1.00	00:00:00	0.00%	0 (0.00%)	$0.00 (0.00%)
1070.	SonyEricsson E10i Xperia X10 Mini	**2** **(0.00%)**	100.00%	2 (0.00%)	50.00%	1.50	00:00:43	0.00%	0 (0.00%)	$0.00 (0.00%)
1071.	SonyEricsson J105i	**2** **(0.00%)**	100.00%	2 (0.00%)	50.00%	1.50	00:00:43	0.00%	0 (0.00%)	$0.00 (0.00%)
1072.	SonyEricsson LT15i Xperia arc SO-01C for DoCoMo	**2** **(0.00%)**	100.00%	2 (0.00%)	50.00%	1.50	00:00:21	0.00%	0 (0.00%)	$0.00 (0.00%)
1073.	SonyEricsson LT18a Xperia Arc	**2** **(0.00%)**	100.00%	2 (0.00%)	100.00%	1.00	00:00:00	0.00%	0 (0.00%)	$0.00 (0.00%)
1074.	SonyEricsson SK17i-o Xperia Mini Pro	**2** **(0.00%)**	50.00%	1 (0.00%)	50.00%	2.00	00:00:18	0.00%	0 (0.00%)	$0.00 (0.00%)
1075.	SonyEricsson SO-03C Xperia ray SO-03C for DoCoMo	**2** **(0.00%)**	50.00%	1 (0.00%)	50.00%	2.50	00:04:27	0.00%	0 (0.00%)	$0.00 (0.00%)
1076.	SonyEricsson ST15a Xperia Mini	**2** **(0.00%)**	100.00%	2 (0.00%)	50.00%	2.00	00:01:38	0.00%	0 (0.00%)	$0.00 (0.00%)

1077.	T-Mobile G2 Touch HTC Sapphire	**2**	**(0.00%)**	100.00%	2	(0.00%)	0.00%	3.50	00:10:26	0.00%	0	(0.00%)	$0.00	(0.00%)
1078.	Tecno M3	**2**	**(0.00%)**	100.00%	2	(0.00%)	50.00%	8.00	00:03:03	0.00%	0	(0.00%)	$0.00	(0.00%)
1079.	THL A1	**2**	**(0.00%)**	100.00%	2	(0.00%)	100.00%	1.00	00:00:00	0.00%	0	(0.00%)	$0.00	(0.00%)
1080.	Verizon Droid	**2**	**(0.00%)**	50.00%	1	(0.00%)	100.00%	1.00	00:00:00	0.00%	0	(0.00%)	$0.00	(0.00%)
1081.	Xolo A700	**2**	**(0.00%)**	50.00%	1	(0.00%)	50.00%	2.00	00:01:09	0.00%	0	(0.00%)	$0.00	(0.00%)
1082.	ZTE Skate Monte Carlo	**2**	**(0.00%)**	100.00%	2	(0.00%)	100.00%	1.00	00:00:00	0.00%	0	(0.00%)	$0.00	(0.00%)
1083.	ZTE U N720	**2**	**(0.00%)**	50.00%	1	(0.00%)	50.00%	2.00	00:01:20	0.00%	0	(0.00%)	$0.00	(0.00%)
1084.	ZTE U807	**2**	**(0.00%)**	50.00%	1	(0.00%)	50.00%	3.00	00:04:46	0.00%	0	(0.00%)	$0.00	(0.00%)
1085.	ZTE V71A Smart Tab 7	**2**	**(0.00%)**	100.00%	2	(0.00%)	50.00%	2.00	00:00:15	0.00%	0	(0.00%)	$0.00	(0.00%)
1086.	ZTE V793 Kis Flex	**2**	**(0.00%)**	100.00%	2	(0.00%)	50.00%	2.00	00:01:06	0.00%	0	(0.00%)	$0.00	(0.00%)
1087.	Access NetFront Ver. 3.5	**1**	**(0.00%)**	100.00%	1	(0.00%)	100.00%	1.00	00:00:00	0.00%	0	(0.00%)	$0.00	(0.00%)
1088.	Acer A1-811	**1**	**(0.00%)**	100.00%	1	(0.00%)	100.00%	1.00	00:00:00	0.00%	0	(0.00%)	$0.00	(0.00%)
1089.	Acer Acer S100 Liquid	**1**	**(0.00%)**	100.00%	1	(0.00%)	100.00%	1.00	00:00:00	0.00%	0	(0.00%)	$0.00	(0.00%)
1090.	Acer B1-711 Iconia B1-711	**1**	**(0.00%)**	100.00%	1	(0.00%)	100.00%	1.00	00:00:00	0.00%	0	(0.00%)	$0.00	(0.00%)
1091.	Acer G100W G100W	**1**	**(0.00%)**	100.00%	1	(0.00%)	100.00%	1.00	00:00:00	0.00%	0	(0.00%)	$0.00	(0.00%)
1092.	Acer S120 Liquid MT	**1**	**(0.00%)**	100.00%	1	(0.00%)	100.00%	1.00	00:00:00	0.00%	0	(0.00%)	$0.00	(0.00%)
1093.	Acer S510	**1**	**(0.00%)**	100.00%	1	(0.00%)	100.00%	1.00	00:00:00	0.00%	0	(0.00%)	$0.00	(0.00%)
1094.	Alcatel MOVE MOVE	**1**	**(0.00%)**	100.00%	1	(0.00%)	100.00%	1.00	00:00:00	0.00%	0	(0.00%)	$0.00	(0.00%)
1095.	Alcatel One Touch 6012A Idol Mini	**1**	**(0.00%)**	100.00%	1	(0.00%)	100.00%	1.00	00:00:00	0.00%	0	(0.00%)	$0.00	(0.00%)
1096.	ALCATEL ONE TOUCH 6040D	**1**	**(0.00%)**	100.00%	1	(0.00%)	100.00%	1.00	00:00:00	0.00%	0	(0.00%)	$0.00	(0.00%)
1097.	ALCATEL ONE TOUCH 7025D	**1**	**(0.00%)**	100.00%	1	(0.00%)	100.00%	1.00	00:00:00	0.00%	0	(0.00%)	$0.00	(0.00%)
1098.	Alcatel ONE TOUCH 7041D	**1**	**(0.00%)**	100.00%	1	(0.00%)	100.00%	1.00	00:00:00	0.00%	0	(0.00%)	$0.00	(0.00%)
1099.	Alcatel One Touch 890	**1**	**(0.00%)**	0.00%	0	(0.00%)	0.00%	5.00	00:05:23	0.00%	0	(0.00%)	$0.00	(0.00%)
1100.	Alcatel OT-6010D One Touch Star 6010D	**1**	**(0.00%)**	100.00%	1	(0.00%)	100.00%	1.00	00:00:00	0.00%	0	(0.00%)	$0.00	(0.00%)
1101.	Alcatel OT-8008D Scribe HD 8008D	**1**	**(0.00%)**	100.00%	1	(0.00%)	0.00%	5.00	00:02:25	0.00%	0	(0.00%)	$0.00	(0.00%)
1102.	Alcatel OT-918D One Touch 918D	**1**	**(0.00%)**	100.00%	1	(0.00%)	100.00%	1.00	00:00:00	0.00%	0	(0.00%)	$0.00	(0.00%)
1103.	Alcatel OT-983 One Touch 983	**1**	**(0.00%)**	100.00%	1	(0.00%)	100.00%	1.00	00:00:00	0.00%	0	(0.00%)	$0.00	(0.00%)
1104.	Alcatel OT-995 One Touch 995	**1**	**(0.00%)**	100.00%	1	(0.00%)	100.00%	1.00	00:00:00	0.00%	0	(0.00%)	$0.00	(0.00%)
1105.	Archos 80 Xenon	**1**	**(0.00%)**	100.00%	1	(0.00%)	100.00%	1.00	00:00:00	0.00%	0	(0.00%)	$0.00	(0.00%)
1106.	Archos A70CHT 7c Home	**1**	**(0.00%)**	100.00%	1	(0.00%)	100.00%	1.00	00:00:00	0.00%	0	(0.00%)	$0.00	(0.00%)
1107.	Arnova 9 G2I	**1**	**(0.00%)**	100.00%	1	(0.00%)	100.00%	1.00	00:00:00	0.00%	0	(0.00%)	$0.00	(0.00%)

1108.	Asus ME302KL MeMO Pad FHD 10	**1** **(0.00%)**	100.00%	1 (0.00%)	100.00%	1.00	00:00:00	0.00%	0 (0.00%)	$0.00 (0.00%)
1109.	Asus Memo pad ME102 K00F	**1** **(0.00%)**	100.00%	1 (0.00%)	100.00%	1.00	00:00:00	0.00%	0 (0.00%)	$0.00 (0.00%)
1110.	Asus Padfone Infinity	**1** **(0.00%)**	100.00%	1 (0.00%)	0.00%	3.00	00:00:28	0.00%	0 (0.00%)	$0.00 (0.00%)
1111.	BBK Vivo X1	**1** **(0.00%)**	100.00%	1 (0.00%)	100.00%	1.00	00:00:00	0.00%	0 (0.00%)	$0.00 (0.00%)
1112.	Bellefonte A10	**1** **(0.00%)**	100.00%	1 (0.00%)	0.00%	3.00	00:01:34	0.00%	0 (0.00%)	$0.00 (0.00%)
1113.	BlackBerry 5233	**1** **(0.00%)**	100.00%	1 (0.00%)	100.00%	1.00	00:00:00	0.00%	0 (0.00%)	$0.00 (0.00%)
1114.	BlackBerry 9310 Curve 9310	**1** **(0.00%)**	100.00%	1 (0.00%)	0.00%	2.00	00:00:15	0.00%	0 (0.00%)	$0.00 (0.00%)
1115.	BlackBerry 9370 BlackBerry Curve 9370	**1** **(0.00%)**	100.00%	1 (0.00%)	100.00%	1.00	00:00:00	0.00%	0 (0.00%)	$0.00 (0.00%)
1116.	BlackBerry 9520 Storm II	**1** **(0.00%)**	100.00%	1 (0.00%)	100.00%	1.00	00:00:00	0.00%	0 (0.00%)	$0.00 (0.00%)
1117.	BlackBerry 9850 Volt	**1** **(0.00%)**	100.00%	1 (0.00%)	100.00%	1.00	00:00:00	0.00%	0 (0.00%)	$0.00 (0.00%)
1118.	BlackBerry 9981 Porsche Design P'9981	**1** **(0.00%)**	100.00%	1 (0.00%)	100.00%	1.00	00:00:00	0.00%	0 (0.00%)	$0.00 (0.00%)
1119.	Blu Dash 3.5	**1** **(0.00%)**	100.00%	1 (0.00%)	100.00%	1.00	00:00:00	0.00%	0 (0.00%)	$0.00 (0.00%)
1120.	Blue Boxing L8301	**1** **(0.00%)**	100.00%	1 (0.00%)	100.00%	1.00	00:00:00	0.00%	0 (0.00%)	$0.00 (0.00%)
1121.	BP I9300	**1** **(0.00%)**	100.00%	1 (0.00%)	0.00%	6.00	00:03:42	0.00%	0 (0.00%)	$0.00 (0.00%)
1122.	BQ M803 Kepler	**1** **(0.00%)**	100.00%	1 (0.00%)	100.00%	1.00	00:00:00	0.00%	0 (0.00%)	$0.00 (0.00%)
1123.	Casio C811 4G G'zOne Commando 4G LTE	**1** **(0.00%)**	100.00%	1 (0.00%)	0.00%	14.00	00:11:56	0.00%	0 (0.00%)	$0.00 (0.00%)
1124.	Celkon A89	**1** **(0.00%)**	100.00%	1 (0.00%)	100.00%	1.00	00:00:00	0.00%	0 (0.00%)	$0.00 (0.00%)
1125.	Coby MID1042 Kyros MID1042	**1** **(0.00%)**	100.00%	1 (0.00%)	100.00%	1.00	00:00:00	0.00%	0 (0.00%)	$0.00 (0.00%)
1126.	Coby MID7033 Kyros 7033	**1** **(0.00%)**	100.00%	1 (0.00%)	100.00%	1.00	00:00:00	0.00%	0 (0.00%)	$0.00 (0.00%)
1127.	Coby MID7034 Kyros MID7034	**1** **(0.00%)**	100.00%	1 (0.00%)	100.00%	1.00	00:00:00	0.00%	0 (0.00%)	$0.00 (0.00%)
1128.	Coby MID7042 Kyros MID7042	**1** **(0.00%)**	100.00%	1 (0.00%)	100.00%	1.00	00:00:00	0.00%	0 (0.00%)	$0.00 (0.00%)
1129.	Coby MID7065 Kyros MID7065	**1** **(0.00%)**	100.00%	1 (0.00%)	100.00%	1.00	00:00:00	0.00%	0 (0.00%)	$0.00 (0.00%)
1130.	Coby MID8024 Kyros MID8024	**1** **(0.00%)**	100.00%	1 (0.00%)	100.00%	1.00	00:00:00	0.00%	0 (0.00%)	$0.00 (0.00%)
1131.	Coby MID8127 Kyros MID8127	**1** **(0.00%)**	100.00%	1 (0.00%)	100.00%	1.00	00:00:00	0.00%	0 (0.00%)	$0.00 (0.00%)
1132.	Coby MID9742 Kyros MID9742	**1** **(0.00%)**	100.00%	1 (0.00%)	0.00%	2.00	00:01:49	0.00%	0 (0.00%)	$0.00 (0.00%)
1133.	Coolpad 5216	**1** **(0.00%)**	100.00%	1 (0.00%)	100.00%	1.00	00:00:00	0.00%	0 (0.00%)	$0.00 (0.00%)
1134.	Coolpad 7295+	**1** **(0.00%)**	100.00%	1 (0.00%)	100.00%	1.00	00:00:00	0.00%	0 (0.00%)	$0.00 (0.00%)
1135.	Coolpad 8150	**1** **(0.00%)**	100.00%	1 (0.00%)	100.00%	1.00	00:00:00	0.00%	0 (0.00%)	$0.00 (0.00%)
1136.	Dell Streak 7	**1** **(0.00%)**	100.00%	1 (0.00%)	0.00%	3.00	00:00:28	0.00%	0 (0.00%)	$0.00 (0.00%)
1137.	Dell Venue	**1** **(0.00%)**	100.00%	1 (0.00%)	0.00%	12.00	00:05:04	0.00%	0 (0.00%)	$0.00 (0.00%)
1138.	Diyomate A6	**1** **(0.00%)**	100.00%	1 (0.00%)	100.00%	1.00	00:00:00	0.00%	0 (0.00%)	$0.00 (0.00%)
1139.	DoCoMo F-02E Arrows X F-02E	**1** **(0.00%)**	100.00%	1 (0.00%)	0.00%	3.00	00:01:32	0.00%	0 (0.00%)	$0.00 (0.00%)

1140.	DoCoMo N-04D NEXT series MEDIAS LTE N-04D	**1** **(0.00%)**	100.00%	1 (0.00%)	100.00%	1.00	00:00:00	0.00%	0 (0.00%)	$0.00 (0.00%)
1141.	DoCoMo P-02E	**1** **(0.00%)**	100.00%	1 (0.00%)	100.00%	1.00	00:00:00	0.00%	0 (0.00%)	$0.00 (0.00%)
1142.	DoCoMo SH-04E Aquos Phone EX	**1** **(0.00%)**	100.00%	1 (0.00%)	100.00%	1.00	00:00:00	0.00%	0 (0.00%)	$0.00 (0.00%)
1143.	DoCoMo SH-10D Aquos Phone sv	**1** **(0.00%)**	100.00%	1 (0.00%)	100.00%	1.00	00:00:00	0.00%	0 (0.00%)	$0.00 (0.00%)
1144.	DoCoMo SO-04E Xperia A	**1** **(0.00%)**	100.00%	1 (0.00%)	100.00%	1.00	00:00:00	0.00%	0 (0.00%)	$0.00 (0.00%)
1145.	DoCoMo T-02D Regza T-02D	**1** **(0.00%)**	100.00%	1 (0.00%)	100.00%	1.00	00:00:00	0.00%	0 (0.00%)	$0.00 (0.00%)
1146.	Elocity A7 A7 Internet Tablet	**1** **(0.00%)**	100.00%	1 (0.00%)	0.00%	11.00	00:02:09	0.00%	0 (0.00%)	$0.00 (0.00%)
1147.	Elson Cynus T1	**1** **(0.00%)**	100.00%	1 (0.00%)	0.00%	3.00	00:00:39	0.00%	0 (0.00%)	$0.00 (0.00%)
1148.	Feiteng GT-H9500	**1** **(0.00%)**	100.00%	1 (0.00%)	100.00%	1.00	00:00:00	0.00%	0 (0.00%)	$0.00 (0.00%)
1149.	Feiteng H7100	**1** **(0.00%)**	100.00%	1 (0.00%)	100.00%	1.00	00:00:00	0.00%	0 (0.00%)	$0.00 (0.00%)
1150.	Fuhu NABI2-NV7A Nabi 2	**1** **(0.00%)**	100.00%	1 (0.00%)	100.00%	1.00	00:00:00	0.00%	0 (0.00%)	$0.00 (0.00%)
1151.	Fujitsu F-02E ARROWS X F-02E for DoCoMo	**1** **(0.00%)**	100.00%	1 (0.00%)	0.00%	2.00	00:01:26	0.00%	0 (0.00%)	$0.00 (0.00%)
1152.	Fujitsu F-05D ARROWS X LTE F-05D for DoCoMo	**1** **(0.00%)**	100.00%	1 (0.00%)	100.00%	1.00	00:00:00	0.00%	0 (0.00%)	$0.00 (0.00%)
1153.	Fujitsu F-07D ARROWS u F-07D	**1** **(0.00%)**	100.00%	1 (0.00%)	100.00%	1.00	00:00:00	0.00%	0 (0.00%)	$0.00 (0.00%)
1154.	Fujitsu IS12F ARROWS ES IS12F for KDDI	**1** **(0.00%)**	100.00%	1 (0.00%)	100.00%	1.00	00:00:00	0.00%	0 (0.00%)	$0.00 (0.00%)
1155.	Fujitsu T-01D REGZA Phone T-01D for DoCoMo	**1** **(0.00%)**	100.00%	1 (0.00%)	100.00%	1.00	00:00:00	0.00%	0 (0.00%)	$0.00 (0.00%)
1156.	Fujitsu Toshiba T-01D REGZA Phone T-01D for DoCoMo	**1** **(0.00%)**	100.00%	1 (0.00%)	100.00%	1.00	00:00:00	0.00%	0 (0.00%)	$0.00 (0.00%)
1157.	Gionee GN708W	**1** **(0.00%)**	100.00%	1 (0.00%)	100.00%	1.00	00:00:00	0.00%	0 (0.00%)	$0.00 (0.00%)
1158.	Google Android App Engine	**1** **(0.00%)**	100.00%	1 (0.00%)	100.00%	1.00	00:00:00	0.00%	0 (0.00%)	$0.00 (0.00%)
1159.	Hisense E926	**1** **(0.00%)**	100.00%	1 (0.00%)	100.00%	1.00	00:00:00	0.00%	0 (0.00%)	$0.00 (0.00%)
1160.	Hisense M470BSA Sero 7 Pro	**1** **(0.00%)**	100.00%	1 (0.00%)	0.00%	16.00	00:04:57	0.00%	0 (0.00%)	$0.00 (0.00%)
1161.	HTC 6500LVW One	**1** **(0.00%)**	100.00%	1 (0.00%)	100.00%	1.00	00:00:00	0.00%	0 (0.00%)	$0.00 (0.00%)
1162.	HTC A620m 8S	**1** **(0.00%)**	100.00%	1 (0.00%)	100.00%	1.00	00:00:00	0.00%	0 (0.00%)	$0.00 (0.00%)
1163.	HTC ADR6325 Merge	**1** **(0.00%)**	100.00%	1 (0.00%)	100.00%	1.00	00:00:00	0.00%	0 (0.00%)	$0.00 (0.00%)
1164.	HTC ADR6330 Rhyme	**1** **(0.00%)**	100.00%	1 (0.00%)	100.00%	1.00	00:00:00	0.00%	0 (0.00%)	$0.00 (0.00%)
1165.	HTC C525c One SV	**1** **(0.00%)**	100.00%	1 (0.00%)	100.00%	1.00	00:00:00	0.00%	0 (0.00%)	$0.00 (0.00%)
1166.	HTC Desire S	**1** **(0.00%)**	100.00%	1 (0.00%)	100.00%	1.00	00:00:00	0.00%	0 (0.00%)	$0.00 (0.00%)
1167.	HTC Hero	**1** **(0.00%)**	100.00%	1 (0.00%)	0.00%	4.00	00:05:45	0.00%	0 (0.00%)	$0.00 (0.00%)

1168.	HTC HTC Desire HD	**1** **(0.00%)**	100.00%	1 (0.00%)	100.00%	1.00	00:00:00	0.00%	0 (0.00%)	$0.00 (0.00%)
1169.	HTC HTC Desire SV	**1** **(0.00%)**	100.00%	1 (0.00%)	100.00%	1.00	00:00:00	0.00%	0 (0.00%)	$0.00 (0.00%)
1170.	HTC ISW11HT EVO WiMAX ISW11HT for KDDI	**1** **(0.00%)**	100.00%	1 (0.00%)	100.00%	1.00	00:00:00	0.00%	0 (0.00%)	$0.00 (0.00%)
1171.	HTC ISW13HT HTC J ISW13HT for KDDI	**1** **(0.00%)**	100.00%	1 (0.00%)	100.00%	1.00	00:00:00	0.00%	0 (0.00%)	$0.00 (0.00%)
1172.	HTC Magic	**1** **(0.00%)**	100.00%	1 (0.00%)	100.00%	1.00	00:00:00	0.00%	0 (0.00%)	$0.00 (0.00%)
1173.	HTC Panache 4G	**1** **(0.00%)**	100.00%	1 (0.00%)	100.00%	1.00	00:00:00	0.00%	0 (0.00%)	$0.00 (0.00%)
1174.	HTC PB99400 Incredible	**1** **(0.00%)**	100.00%	1 (0.00%)	100.00%	1.00	00:00:00	0.00%	0 (0.00%)	$0.00 (0.00%)
1175.	HTC PH06130 Status	**1** **(0.00%)**	100.00%	1 (0.00%)	100.00%	1.00	00:00:00	0.00%	0 (0.00%)	$0.00 (0.00%)
1176.	HTC PM23300 Windows Phone 8X	**1** **(0.00%)**	100.00%	1 (0.00%)	0.00%	3.00	00:02:23	0.00%	0 (0.00%)	$0.00 (0.00%)
1177.	HTC PM3511 One X+	**1** **(0.00%)**	100.00%	1 (0.00%)	100.00%	1.00	00:00:00	0.00%	0 (0.00%)	$0.00 (0.00%)
1178.	HTC PN07120 One	**1** **(0.00%)**	100.00%	1 (0.00%)	0.00%	9.00	00:03:48	0.00%	0 (0.00%)	$0.00 (0.00%)
1179.	HTC PO58220 One mini	**1** **(0.00%)**	100.00%	1 (0.00%)	100.00%	1.00	00:00:00	0.00%	0 (0.00%)	$0.00 (0.00%)
1180.	HTC Radar	**1** **(0.00%)**	100.00%	1 (0.00%)	100.00%	1.00	00:00:00	0.00%	0 (0.00%)	$0.00 (0.00%)
1181.	HTC T120C One V	**1** **(0.00%)**	100.00%	1 (0.00%)	100.00%	1.00	00:00:00	0.00%	0 (0.00%)	$0.00 (0.00%)
1182.	HTC T320e One V	**1** **(0.00%)**	100.00%	1 (0.00%)	100.00%	1.00	00:00:00	0.00%	0 (0.00%)	$0.00 (0.00%)
1183.	HTC T528t One ST	**1** **(0.00%)**	100.00%	1 (0.00%)	100.00%	1.00	00:00:00	0.00%	0 (0.00%)	$0.00 (0.00%)
1184.	HTC T8788 Surround	**1** **(0.00%)**	100.00%	1 (0.00%)	100.00%	1.00	00:00:00	0.00%	0 (0.00%)	$0.00 (0.00%)
1185.	HTC Windows Phone 8X for Orange	**1** **(0.00%)**	100.00%	1 (0.00%)	100.00%	1.00	00:00:00	0.00%	0 (0.00%)	$0.00 (0.00%)
1186.	HTC X315b Sensation XL with Beats Audio	**1** **(0.00%)**	100.00%	1 (0.00%)	0.00%	2.00	00:00:23	0.00%	0 (0.00%)	$0.00 (0.00%)
1187.	Huawei 4Afrika	**1** **(0.00%)**	100.00%	1 (0.00%)	0.00%	3.00	00:02:07	0.00%	0 (0.00%)	$0.00 (0.00%)
1188.	Huawei C8650	**1** **(0.00%)**	100.00%	1 (0.00%)	100.00%	1.00	00:00:00	0.00%	0 (0.00%)	$0.00 (0.00%)
1189.	Huawei C8812E Ascend C8812E	**1** **(0.00%)**	100.00%	1 (0.00%)	0.00%	2.00	00:00:50	0.00%	0 (0.00%)	$0.00 (0.00%)
1190.	Huawei C8813Q	**1** **(0.00%)**	100.00%	1 (0.00%)	0.00%	4.00	00:01:23	0.00%	0 (0.00%)	$0.00 (0.00%)
1191.	Huawei C8815	**1** **(0.00%)**	100.00%	1 (0.00%)	100.00%	1.00	00:00:00	0.00%	0 (0.00%)	$0.00 (0.00%)
1192.	Huawei Comet T-Mobile Comet	**1** **(0.00%)**	100.00%	1 (0.00%)	100.00%	1.00	00:00:00	0.00%	0 (0.00%)	$0.00 (0.00%)
1193.	Huawei G520-5000	**1** **(0.00%)**	100.00%	1 (0.00%)	0.00%	5.00	00:02:36	0.00%	0 (0.00%)	$0.00 (0.00%)
1194.	Huawei H883G	**1** **(0.00%)**	100.00%	1 (0.00%)	0.00%	2.00	00:00:23	0.00%	0 (0.00%)	$0.00 (0.00%)
1195.	Huawei HUAWEI U8815 Ascent G 300	**1** **(0.00%)**	100.00%	1 (0.00%)	100.00%	1.00	00:00:00	0.00%	0 (0.00%)	$0.00 (0.00%)
1196.	Huawei M660	**1** **(0.00%)**	100.00%	1 (0.00%)	100.00%	1.00	00:00:00	0.00%	0 (0.00%)	$0.00 (0.00%)
1197.	Huawei M865 Ascend II	**1** **(0.00%)**	100.00%	1 (0.00%)	0.00%	3.00	00:01:01	0.00%	0 (0.00%)	$0.00 (0.00%)

1198.	Huawei M886 Glory	**1**	**(0.00%)**	100.00%	1	(0.00%)	0.00%	2.00	00:01:25	0.00%	0	(0.00%)	$0.00	(0.00%)
1199.	Huawei S41HW Pocket WiFi S II for emobile	**1**	**(0.00%)**	100.00%	1	(0.00%)	100.00%	1.00	00:00:00	0.00%	0	(0.00%)	$0.00	(0.00%)
1200.	Huawei S7-103	**1**	**(0.00%)**	100.00%	1	(0.00%)	0.00%	4.00	00:03:16	0.00%	0	(0.00%)	$0.00	(0.00%)
1201.	Huawei U8150 Ideos	**1**	**(0.00%)**	100.00%	1	(0.00%)	100.00%	1.00	00:00:00	0.00%	0	(0.00%)	$0.00	(0.00%)
1202.	Huawei U8650 Sonic	**1**	**(0.00%)**	100.00%	1	(0.00%)	0.00%	3.00	00:00:59	0.00%	0	(0.00%)	$0.00	(0.00%)
1203.	Huawei U8655 Ascend Y200	**1**	**(0.00%)**	100.00%	1	(0.00%)	100.00%	1.00	00:00:00	0.00%	0	(0.00%)	$0.00	(0.00%)
1204.	Huawei U8666-1 Ascend Y201 Pro	**1**	**(0.00%)**	100.00%	1	(0.00%)	100.00%	1.00	00:00:00	0.00%	0	(0.00%)	$0.00	(0.00%)
1205.	Huawei U8950-1 Ascend G600	**1**	**(0.00%)**	100.00%	1	(0.00%)	100.00%	1.00	00:00:00	0.00%	0	(0.00%)	$0.00	(0.00%)
1206.	Huawei U9202L-1 Ascend P1	**1**	**(0.00%)**	100.00%	1	(0.00%)	100.00%	1.00	00:00:00	0.00%	0	(0.00%)	$0.00	(0.00%)
1207.	Huawei U9510E Honor 2	**1**	**(0.00%)**	100.00%	1	(0.00%)	0.00%	5.00	00:01:27	0.00%	0	(0.00%)	$0.00	(0.00%)
1208.	Huawei Y210-2010 Ascend Y210	**1**	**(0.00%)**	100.00%	1	(0.00%)	0.00%	2.00	00:00:18	0.00%	0	(0.00%)	$0.00	(0.00%)
1209.	Huawei Y301-A1 Valiant	**1**	**(0.00%)**	100.00%	1	(0.00%)	100.00%	1.00	00:00:00	0.00%	0	(0.00%)	$0.00	(0.00%)
1210.	Huawei Y310-T10 Ascend Y 310	**1**	**(0.00%)**	100.00%	1	(0.00%)	100.00%	1.00	00:00:00	0.00%	0	(0.00%)	$0.00	(0.00%)
1211.	Huawei Y511-U30	**1**	**(0.00%)**	100.00%	1	(0.00%)	0.00%	2.00	00:02:49	0.00%	0	(0.00%)	$0.00	(0.00%)
1212.	Intex Aqua 3.2	**1**	**(0.00%)**	100.00%	1	(0.00%)	100.00%	1.00	00:00:00	0.00%	0	(0.00%)	$0.00	(0.00%)
1213.	Jiayu JY-G2	**1**	**(0.00%)**	100.00%	1	(0.00%)	0.00%	3.00	00:13:05	0.00%	0	(0.00%)	$0.00	(0.00%)
1214.	K-Touch T619	**1**	**(0.00%)**	100.00%	1	(0.00%)	100.00%	1.00	00:00:00	0.00%	0	(0.00%)	$0.00	(0.00%)
1215.	K-Touch U81t	**1**	**(0.00%)**	100.00%	1	(0.00%)	100.00%	1.00	00:00:00	0.00%	0	(0.00%)	$0.00	(0.00%)
1216.	Karbonn A1+	**1**	**(0.00%)**	100.00%	1	(0.00%)	100.00%	1.00	00:00:00	0.00%	0	(0.00%)	$0.00	(0.00%)
1217.	Karbonn A2+	**1**	**(0.00%)**	100.00%	1	(0.00%)	0.00%	3.00	00:00:40	0.00%	0	(0.00%)	$0.00	(0.00%)
1218.	Karbonn A4	**1**	**(0.00%)**	100.00%	1	(0.00%)	100.00%	1.00	00:00:00	0.00%	0	(0.00%)	$0.00	(0.00%)
1219.	Karbonn A7	**1**	**(0.00%)**	100.00%	1	(0.00%)	0.00%	4.00	00:01:42	0.00%	0	(0.00%)	$0.00	(0.00%)
1220.	KDDI IS12S acro HD IS12S	**1**	**(0.00%)**	100.00%	1	(0.00%)	100.00%	1.00	00:00:00	0.00%	0	(0.00%)	$0.00	(0.00%)
1221.	KDDI KYL21 DIGNO S KYL21	**1**	**(0.00%)**	100.00%	1	(0.00%)	100.00%	1.00	00:00:00	0.00%	0	(0.00%)	$0.00	(0.00%)
1222.	KT Tech KM-S300 TAKE HD	**1**	**(0.00%)**	100.00%	1	(0.00%)	100.00%	1.00	00:00:00	0.00%	0	(0.00%)	$0.00	(0.00%)
1223.	Kyocera C5120 Milano	**1**	**(0.00%)**	100.00%	1	(0.00%)	100.00%	1.00	00:00:00	0.00%	0	(0.00%)	$0.00	(0.00%)
1224.	Kyocera M9300 Echo	**1**	**(0.00%)**	100.00%	1	(0.00%)	100.00%	1.00	00:00:00	0.00%	0	(0.00%)	$0.00	(0.00%)
1225.	Lava Iris 349	**1**	**(0.00%)**	100.00%	1	(0.00%)	100.00%	1.00	00:00:00	0.00%	0	(0.00%)	$0.00	(0.00%)
1226.	Le Pan II	**1**	**(0.00%)**	100.00%	1	(0.00%)	0.00%	3.00	00:00:45	0.00%	0	(0.00%)	$0.00	(0.00%)
1227.	Lenovo A2207 LePad A2207	**1**	**(0.00%)**	100.00%	1	(0.00%)	100.00%	1.00	00:00:00	0.00%	0	(0.00%)	$0.00	(0.00%)
1228.	Lenovo A60	**1**	**(0.00%)**	100.00%	1	(0.00%)	0.00%	4.00	00:13:11	0.00%	0	(0.00%)	$0.00	(0.00%)
1229.	Lenovo A630T	**1**	**(0.00%)**	100.00%	1	(0.00%)	100.00%	1.00	00:00:00	0.00%	0	(0.00%)	$0.00	(0.00%)
1230.	Lenovo A660	**1**	**(0.00%)**	100.00%	1	(0.00%)	100.00%	1.00	00:00:00	0.00%	0	(0.00%)	$0.00	(0.00%)

1231.	Lenovo A766	**1** **(0.00%)**	100.00%	1 (0.00%)	100.00%	1.00	00:00:00	0.00%	0 (0.00%)	$0.00 (0.00%)
1232.	Lenovo A790e	**1** **(0.00%)**	100.00%	1 (0.00%)	0.00%	3.00	00:01:09	0.00%	0 (0.00%)	$0.00 (0.00%)
1233.	Lenovo A800	**1** **(0.00%)**	100.00%	1 (0.00%)	100.00%	1.00	00:00:00	0.00%	0 (0.00%)	$0.00 (0.00%)
1234.	LENOVO Lenovo A516	**1** **(0.00%)**	100.00%	1 (0.00%)	0.00%	6.00	00:08:24	0.00%	0 (0.00%)	$0.00 (0.00%)
1235.	Lenovo Lenovo P770	**1** **(0.00%)**	100.00%	1 (0.00%)	100.00%	1.00	00:00:00	0.00%	0 (0.00%)	$0.00 (0.00%)
1236.	Lenovo S930	**1** **(0.00%)**	100.00%	1 (0.00%)	100.00%	1.00	00:00:00	0.00%	0 (0.00%)	$0.00 (0.00%)
1237.	LG Ally	**1** **(0.00%)**	100.00%	1 (0.00%)	0.00%	3.00	00:00:52	0.00%	0 (0.00%)	$0.00 (0.00%)
1238.	LG C330	**1** **(0.00%)**	100.00%	1 (0.00%)	100.00%	1.00	00:00:00	0.00%	0 (0.00%)	$0.00 (0.00%)
1239.	LG C397	**1** **(0.00%)**	100.00%	1 (0.00%)	0.00%	12.00	02:03:24	0.00%	0 (0.00%)	$0.00 (0.00%)
1240.	LG C660h Optimus Pro	**1** **(0.00%)**	100.00%	1 (0.00%)	0.00%	3.00	00:09:31	0.00%	0 (0.00%)	$0.00 (0.00%)
1241.	LG D300	**1** **(0.00%)**	100.00%	1 (0.00%)	100.00%	1.00	00:00:00	0.00%	0 (0.00%)	$0.00 (0.00%)
1242.	LG D605 Optimus L9 II	**1** **(0.00%)**	100.00%	1 (0.00%)	100.00%	1.00	00:00:00	0.00%	0 (0.00%)	$0.00 (0.00%)
1243.	LG E411g Optimus II L1	**1** **(0.00%)**	100.00%	1 (0.00%)	100.00%	1.00	00:00:00	0.00%	0 (0.00%)	$0.00 (0.00%)
1244.	LG E435 Optimus L3 II Dual	**1** **(0.00%)**	100.00%	1 (0.00%)	0.00%	2.00	00:06:25	0.00%	0 (0.00%)	$0.00 (0.00%)
1245.	LG E450B Optimus L5 II	**1** **(0.00%)**	100.00%	1 (0.00%)	100.00%	1.00	00:00:00	0.00%	0 (0.00%)	$0.00 (0.00%)
1246.	LG E617G Optimus L5	**1** **(0.00%)**	100.00%	1 (0.00%)	0.00%	3.00	00:01:38	0.00%	0 (0.00%)	$0.00 (0.00%)
1247.	LG E720 Optimus Chic	**1** **(0.00%)**	100.00%	1 (0.00%)	100.00%	1.00	00:00:00	0.00%	0 (0.00%)	$0.00 (0.00%)
1248.	LG E730	**1** **(0.00%)**	100.00%	1 (0.00%)	100.00%	1.00	00:00:00	0.00%	0 (0.00%)	$0.00 (0.00%)
1249.	LG E739BK myTouch E739BK	**1** **(0.00%)**	100.00%	1 (0.00%)	100.00%	1.00	00:00:00	0.00%	0 (0.00%)	$0.00 (0.00%)
1250.	LG E971 Optimus G	**1** **(0.00%)**	100.00%	1 (0.00%)	0.00%	4.00	00:00:56	0.00%	0 (0.00%)	$0.00 (0.00%)
1251.	LG E973 Optimus G	**1** **(0.00%)**	100.00%	1 (0.00%)	100.00%	1.00	00:00:00	0.00%	0 (0.00%)	$0.00 (0.00%)
1252.	LG F160L Optimus LTE2	**1** **(0.00%)**	100.00%	1 (0.00%)	0.00%	4.00	00:00:36	0.00%	0 (0.00%)	$0.00 (0.00%)
1253.	LG F160LV Optimus LTE2	**1** **(0.00%)**	100.00%	1 (0.00%)	100.00%	1.00	00:00:00	0.00%	0 (0.00%)	$0.00 (0.00%)
1254.	LG F160S Optimus LTE2	**1** **(0.00%)**	100.00%	1 (0.00%)	0.00%	3.00	00:00:18	0.00%	0 (0.00%)	$0.00 (0.00%)
1255.	LG F180K Optimus G	**1** **(0.00%)**	100.00%	1 (0.00%)	100.00%	1.00	00:00:00	0.00%	0 (0.00%)	$0.00 (0.00%)
1256.	LG F180S Optimus G	**1** **(0.00%)**	100.00%	1 (0.00%)	100.00%	1.00	00:00:00	0.00%	0 (0.00%)	$0.00 (0.00%)
1257.	LG F320K G2	**1** **(0.00%)**	100.00%	1 (0.00%)	0.00%	3.00	00:00:32	0.00%	0 (0.00%)	$0.00 (0.00%)
1258.	LG F320S Optimus G2	**1** **(0.00%)**	100.00%	1 (0.00%)	100.00%	1.00	00:00:00	0.00%	0 (0.00%)	$0.00 (0.00%)
1259.	LG F350S G Pro 2	**1** **(0.00%)**	100.00%	1 (0.00%)	100.00%	1.00	00:00:00	0.00%	0 (0.00%)	$0.00 (0.00%)
1260.	LG KM555	**1** **(0.00%)**	100.00%	1 (0.00%)	100.00%	1.00	00:00:00	0.00%	0 (0.00%)	$0.00 (0.00%)
1261.	LG L96G Optimus Ultimate	**1** **(0.00%)**	100.00%	1 (0.00%)	0.00%	2.00	00:01:16	0.00%	0 (0.00%)	$0.00 (0.00%)
1262.	LG LG-GX500	**1** **(0.00%)**	100.00%	1 (0.00%)	100.00%	1.00	00:00:00	0.00%	0 (0.00%)	$0.00 (0.00%)

1263.	LG LG-KP570	**1** (0.00%)	100.00%	1 (0.00%)	0.00%	9.00	00:16:10	0.00%	0 (0.00%)	$0.00 (0.00%)
1264.	LG LG-P713 Optimus L7 II	**1** (0.00%)	100.00%	1 (0.00%)	0.00%	3.00	00:01:36	0.00%	0 (0.00%)	$0.00 (0.00%)
1265.	LG LG/E988/V1.(	**1** (0.00%)	100.00%	1 (0.00%)	100.00%	1.00	00:00:00	0.00%	0 (0.00%)	$0.00 (0.00%)
1266.	LG LG/P875/V1.(	**1** (0.00%)	100.00%	1 (0.00%)	0.00%	3.00	00:01:10	0.00%	0 (0.00%)	$0.00 (0.00%)
1267.	LG LS696 Optimus Elite	**1** (0.00%)	100.00%	1 (0.00%)	0.00%	3.00	00:04:04	0.00%	0 (0.00%)	$0.00 (0.00%)
1268.	LG LS840 Viper 4G LTE	**1** (0.00%)	100.00%	1 (0.00%)	100.00%	1.00	00:00:00	0.00%	0 (0.00%)	$0.00 (0.00%)
1269.	LG LS855 Marquee	**1** (0.00%)	100.00%	1 (0.00%)	0.00%	4.00	00:02:46	0.00%	0 (0.00%)	$0.00 (0.00%)
1270.	LG LW770 Optimus Regard	**1** (0.00%)	100.00%	1 (0.00%)	0.00%	2.00	00:02:11	0.00%	0 (0.00%)	$0.00 (0.00%)
1271.	LG MS840 Connect 4G	**1** (0.00%)	100.00%	1 (0.00%)	0.00%	8.00	00:03:17	0.00%	0 (0.00%)	$0.00 (0.00%)
1272.	LG MS910 Bryce	**1** (0.00%)	100.00%	1 (0.00%)	0.00%	4.00	00:02:00	0.00%	0 (0.00%)	$0.00 (0.00%)
1273.	LG P505 Phoeni›	**1** (0.00%)	100.00%	1 (0.00%)	100.00%	1.00	00:00:00	0.00%	0 (0.00%)	$0.00 (0.00%)
1274.	LG P698F Optimus Dual Sim	**1** (0.00%)	100.00%	1 (0.00%)	100.00%	1.00	00:00:00	0.00%	0 (0.00%)	$0.00 (0.00%)
1275.	LG P720 Optimus 3D Max	**1** (0.00%)	100.00%	1 (0.00%)	100.00%	1.00	00:00:00	0.00%	0 (0.00%)	$0.00 (0.00%)
1276.	LG P870 Escape	**1** (0.00%)	100.00%	1 (0.00%)	100.00%	1.00	00:00:00	0.00%	0 (0.00%)	$0.00 (0.00%)
1277.	LG P895 Optimus Vu	**1** (0.00%)	100.00%	1 (0.00%)	100.00%	1.00	00:00:00	0.00%	0 (0.00%)	$0.00 (0.00%)
1278.	LG P925 Thrill	**1** (0.00%)	100.00%	1 (0.00%)	100.00%	1.00	00:00:00	0.00%	0 (0.00%)	$0.00 (0.00%)
1279.	LG P936 Optimus LTE	**1** (0.00%)	100.00%	1 (0.00%)	100.00%	1.00	00:00:00	0.00%	0 (0.00%)	$0.00 (0.00%)
1280.	LG P990h Optimus 2X	**1** (0.00%)	100.00%	1 (0.00%)	100.00%	1.00	00:00:00	0.00%	0 (0.00%)	$0.00 (0.00%)
1281.	LG P999 T-Mobile G2x	**1** (0.00%)	100.00%	1 (0.00%)	100.00%	1.00	00:00:00	0.00%	0 (0.00%)	$0.00 (0.00%)
1282.	LG VM670 Optimus V	**1** (0.00%)	100.00%	1 (0.00%)	100.00%	1.00	00:00:00	0.00%	0 (0.00%)	$0.00 (0.00%)
1283.	LG VM696 Optimus Elite	**1** (0.00%)	100.00%	1 (0.00%)	100.00%	1.00	00:00:00	0.00%	0 (0.00%)	$0.00 (0.00%)
1284.	LG VM701 Optimus Slider	**1** (0.00%)	100.00%	1 (0.00%)	100.00%	1.00	00:00:00	0.00%	0 (0.00%)	$0.00 (0.00%)
1285.	LG VN271 Extravert	**1** (0.00%)	100.00%	1 (0.00%)	100.00%	1.00	00:00:00	0.00%	0 (0.00%)	$0.00 (0.00%)
1286.	LG VS410PP Optimus Zone	**1** (0.00%)	100.00%	1 (0.00%)	100.00%	1.00	00:00:00	0.00%	0 (0.00%)	$0.00 (0.00%)
1287.	LG VS660 Vorte›	**1** (0.00%)	100.00%	1 (0.00%)	0.00%	2.00	00:03:22	0.00%	0 (0.00%)	$0.00 (0.00%)
1288.	LG VS700 Enlighten	**1** (0.00%)	100.00%	1 (0.00%)	100.00%	1.00	00:00:00	0.00%	0 (0.00%)	$0.00 (0.00%)
1289.	LG VS870 4G Lucid 2	**1** (0.00%)	100.00%	1 (0.00%)	100.00%	1.00	00:00:00	0.00%	0 (0.00%)	$0.00 (0.00%)
1290.	Majestic TAB 17(	**1** (0.00%)	100.00%	1 (0.00%)	100.00%	1.00	00:00:00	0.00%	0 (0.00%)	$0.00 (0.00%)
1291.	Medion LIFETAB S9714	**1** (0.00%)	100.00%	1 (0.00%)	100.00%	1.00	00:00:00	0.00%	0 (0.00%)	$0.00 (0.00%)
1292.	Micromax A34	**1** (0.00%)	100.00%	1 (0.00%)	100.00%	1.00	00:00:00	0.00%	0 (0.00%)	$0.00 (0.00%)
1293.	Micromax A54 A54 Smarty 3.5	**1** (0.00%)	100.00%	1 (0.00%)	100.00%	1.00	00:00:00	0.00%	0 (0.00%)	$0.00 (0.00%)
1294.	Micromax A63 Canvas Fun	**1** (0.00%)	100.00%	1 (0.00%)	0.00%	5.00	00:03:42	0.00%	0 (0.00%)	$0.00 (0.00%)
1295.	Micromax A65	**1** (0.00%)	100.00%	1 (0.00%)	100.00%	1.00	00:00:00	0.00%	0 (0.00%)	$0.00 (0.00%)

1296.	Micromax A67 Bolt A67	**1** **(0.00%)**	100.00%	1 (0.00%)	100.00%	1.00	00:00:00	0.00%	0 (0.00%)	$0.00 (0.00%)
1297.	Micromax A87 Ninja 4	**1** **(0.00%)**	100.00%	1 (0.00%)	100.00%	1.00	00:00:00	0.00%	0 (0.00%)	$0.00 (0.00%)
1298.	Micromax A90s Superfone Pixel	**1** **(0.00%)**	100.00%	1 (0.00%)	100.00%	1.00	00:00:00	0.00%	0 (0.00%)	$0.00 (0.00%)
1299.	Micromax P275 Funbook Infinity P275	**1** **(0.00%)**	100.00%	1 (0.00%)	100.00%	1.00	00:00:00	0.00%	0 (0.00%)	$0.00 (0.00%)
1300.	Motorola EX430 MotoGO	**1** **(0.00%)**	100.00%	1 (0.00%)	100.00%	1.00	00:00:00	0.00%	0 (0.00%)	$0.00 (0.00%)
1301.	Motorola MB200 Cliq	**1** **(0.00%)**	100.00%	1 (0.00%)	100.00%	1.00	00:00:00	0.00%	0 (0.00%)	$0.00 (0.00%)
1302.	Motorola MB501 CLIQ XT	**1** **(0.00%)**	100.00%	1 (0.00%)	100.00%	1.00	00:00:00	0.00%	0 (0.00%)	$0.00 (0.00%)
1303.	Motorola MB502	**1** **(0.00%)**	100.00%	1 (0.00%)	0.00%	3.00	00:01:29	0.00%	0 (0.00%)	$0.00 (0.00%)
1304.	Motorola MB508 Sage	**1** **(0.00%)**	100.00%	1 (0.00%)	0.00%	5.00	00:03:04	0.00%	0 (0.00%)	$0.00 (0.00%)
1305.	Motorola MB511 Ruth	**1** **(0.00%)**	100.00%	1 (0.00%)	0.00%	4.00	00:17:51	0.00%	0 (0.00%)	$0.00 (0.00%)
1306.	Motorola MB867 Milestone X2	**1** **(0.00%)**	100.00%	1 (0.00%)	0.00%	5.00	00:06:24	0.00%	0 (0.00%)	$0.00 (0.00%)
1307.	Motorola MZ607 Xoom 2 ME	**1** **(0.00%)**	100.00%	1 (0.00%)	100.00%	1.00	00:00:00	0.00%	0 (0.00%)	$0.00 (0.00%)
1308.	Motorola WX445 Ciena	**1** **(0.00%)**	100.00%	1 (0.00%)	0.00%	2.00	00:00:25	0.00%	0 (0.00%)	$0.00 (0.00%)
1309.	Motorola XT557 Defy XT	**1** **(0.00%)**	100.00%	1 (0.00%)	0.00%	3.00	00:00:49	0.00%	0 (0.00%)	$0.00 (0.00%)
1310.	Motorola XT682 Atrix TV	**1** **(0.00%)**	100.00%	1 (0.00%)	0.00%	5.00	00:03:53	0.00%	0 (0.00%)	$0.00 (0.00%)
1311.	Motorola XT882 MOTO XT882	**1** **(0.00%)**	100.00%	1 (0.00%)	100.00%	1.00	00:00:00	0.00%	0 (0.00%)	$0.00 (0.00%)
1312.	Motorola XT897 Photon Q 4G LTE	**1** **(0.00%)**	100.00%	1 (0.00%)	0.00%	3.00	00:00:24	0.00%	0 (0.00%)	$0.00 (0.00%)
1313.	Motorola XT905 RAZR M 4G LTE	**1** **(0.00%)**	100.00%	1 (0.00%)	0.00%	3.00	00:00:40	0.00%	0 (0.00%)	$0.00 (0.00%)
1314.	MTC 970H	**1** **(0.00%)**	100.00%	1 (0.00%)	100.00%	1.00	00:00:00	0.00%	0 (0.00%)	$0.00 (0.00%)
1315.	NEC N-04C Medias N-04C for DoCoMo	**1** **(0.00%)**	100.00%	1 (0.00%)	100.00%	1.00	00:00:00	0.00%	0 (0.00%)	$0.00 (0.00%)
1316.	NEC N-04D MEDIAS LTE N-04D for DoCoMo	**1** **(0.00%)**	100.00%	1 (0.00%)	100.00%	1.00	00:00:00	0.00%	0 (0.00%)	$0.00 (0.00%)
1317.	NEC N-06C Medias WP N-06C for DoCoMo	**1** **(0.00%)**	100.00%	1 (0.00%)	0.00%	8.00	00:02:06	0.00%	0 (0.00%)	$0.00 (0.00%)
1318.	NEC N-06E MEDIAS X N-06E for DoCoMo	**1** **(0.00%)**	100.00%	1 (0.00%)	100.00%	1.00	00:00:00	0.00%	0 (0.00%)	$0.00 (0.00%)
1319.	Nextbook Next7P12	**1** **(0.00%)**	100.00%	1 (0.00%)	100.00%	1.00	00:00:00	0.00%	0 (0.00%)	$0.00 (0.00%)
1320.	Nextbook Next7P12-8G	**1** **(0.00%)**	100.00%	1 (0.00%)	100.00%	1.00	00:00:00	0.00%	0 (0.00%)	$0.00 (0.00%)
1321.	Nextbook NX008HD8G	**1** **(0.00%)**	100.00%	1 (0.00%)	0.00%	3.00	00:00:04	0.00%	0 (0.00%)	$0.00 (0.00%)
1322.	NGM Forward Prime	**1** **(0.00%)**	100.00%	1 (0.00%)	100.00%	1.00	00:00:00	0.00%	0 (0.00%)	$0.00 (0.00%)
1323.	Nokia 109	**1** **(0.00%)**	100.00%	1 (0.00%)	0.00%	3.00	00:03:06	0.00%	0 (0.00%)	$0.00 (0.00%)
1324.	Nokia 208	**1** **(0.00%)**	100.00%	1 (0.00%)	100.00%	1.00	00:00:00	0.00%	0 (0.00%)	$0.00 (0.00%)

1325.	Nokia 208.3	**1** **(0.00%)**	100.00%	1 (0.00%)	0.00%	2.00	00:01:24	0.00%	0 (0.00%)	$0.00 (0.00%)
1326.	Nokia 5228	**1** **(0.00%)**	100.00%	1 (0.00%)	100.00%	1.00	00:00:00	0.00%	0 (0.00%)	$0.00 (0.00%)
1327.	Nokia 5232	**1** **(0.00%)**	100.00%	1 (0.00%)	100.00%	1.00	00:00:00	0.00%	0 (0.00%)	$0.00 (0.00%)
1328.	Nokia 5236	**1** **(0.00%)**	100.00%	1 (0.00%)	100.00%	1.00	00:00:00	0.00%	0 (0.00%)	$0.00 (0.00%)
1329.	Nokia 5238	**1** **(0.00%)**	100.00%	1 (0.00%)	100.00%	1.00	00:00:00	0.00%	0 (0.00%)	$0.00 (0.00%)
1330.	Nokia 6700 classic	**1** **(0.00%)**	100.00%	1 (0.00%)	0.00%	2.00	00:01:17	0.00%	0 (0.00%)	$0.00 (0.00%)
1331.	Nokia 6790 Slide	**1** **(0.00%)**	100.00%	1 (0.00%)	100.00%	1.00	00:00:00	0.00%	0 (0.00%)	$0.00 (0.00%)
1332.	Nokia 7310 Supernova	**1** **(0.00%)**	100.00%	1 (0.00%)	100.00%	1.00	00:00:00	0.00%	0 (0.00%)	$0.00 (0.00%)
1333.	Nokia 808 PureView	**1** **(0.00%)**	100.00%	1 (0.00%)	100.00%	1.00	00:00:00	0.00%	0 (0.00%)	$0.00 (0.00%)
1334.	Nokia Asha 210	**1** **(0.00%)**	100.00%	1 (0.00%)	100.00%	1.00	00:00:00	0.00%	0 (0.00%)	$0.00 (0.00%)
1335.	Nokia Asha 230 DualSIM	**1** **(0.00%)**	100.00%	1 (0.00%)	100.00%	1.00	00:00:00	0.00%	0 (0.00%)	$0.00 (0.00%)
1336.	Nokia E75 E75	**1** **(0.00%)**	100.00%	1 (0.00%)	0.00%	5.00	00:01:46	0.00%	0 (0.00%)	$0.00 (0.00%)
1337.	Nokia E90 Communicator Communicator	**1** **(0.00%)**	100.00%	1 (0.00%)	100.00%	1.00	00:00:00	0.00%	0 (0.00%)	$0.00 (0.00%)
1338.	Nokia N-Gage QD	**1** **(0.00%)**	100.00%	1 (0.00%)	100.00%	1.00	00:00:00	0.00%	0 (0.00%)	$0.00 (0.00%)
1339.	Nokia N72	**1** **(0.00%)**	100.00%	1 (0.00%)	100.00%	1.00	00:00:00	0.00%	0 (0.00%)	$0.00 (0.00%)
1340.	Nokia N79	**1** **(0.00%)**	100.00%	1 (0.00%)	100.00%	1.00	00:00:00	0.00%	0 (0.00%)	$0.00 (0.00%)
1341.	Nokia N82	**1** **(0.00%)**	100.00%	1 (0.00%)	0.00%	2.00	00:00:43	0.00%	0 (0.00%)	$0.00 (0.00%)
1342.	Nokia N95 8GB	**1** **(0.00%)**	100.00%	1 (0.00%)	100.00%	1.00	00:00:00	0.00%	0 (0.00%)	$0.00 (0.00%)
1343.	Nokia N95-3 NAM	**1** **(0.00%)**	100.00%	1 (0.00%)	100.00%	1.00	00:00:00	0.00%	0 (0.00%)	$0.00 (0.00%)
1344.	Nokia N97 mini	**1** **(0.00%)**	100.00%	1 (0.00%)	100.00%	1.00	00:00:00	0.00%	0 (0.00%)	$0.00 (0.00%)
1345.	Nokia X3-00	**1** **(0.00%)**	100.00%	1 (0.00%)	0.00%	2.00	00:00:41	0.00%	0 (0.00%)	$0.00 (0.00%)
1346.	Nokia X5-01	**1** **(0.00%)**	100.00%	1 (0.00%)	100.00%	1.00	00:00:00	0.00%	0 (0.00%)	$0.00 (0.00%)
1347.	Odys Xpress	**1** **(0.00%)**	100.00%	1 (0.00%)	0.00%	3.00	00:00:28	0.00%	0 (0.00%)	$0.00 (0.00%)
1348.	Olivetti OliPad 100	**1** **(0.00%)**	100.00%	1 (0.00%)	100.00%	1.00	00:00:00	0.00%	0 (0.00%)	$0.00 (0.00%)
1349.	Olivetti OliPad 110	**1** **(0.00%)**	100.00%	1 (0.00%)	100.00%	1.00	00:00:00	0.00%	0 (0.00%)	$0.00 (0.00%)
1350.	Oppo R815T	**1** **(0.00%)**	100.00%	1 (0.00%)	100.00%	1.00	00:00:00	0.00%	0 (0.00%)	$0.00 (0.00%)
1351.	Oppo R833T	**1** **(0.00%)**	100.00%	1 (0.00%)	100.00%	1.00	00:00:00	0.00%	0 (0.00%)	$0.00 (0.00%)
1352.	Orange G740-L00 Yumo	**1** **(0.00%)**	100.00%	1 (0.00%)	100.00%	1.00	00:00:00	0.00%	0 (0.00%)	$0.00 (0.00%)
1353.	Panasonic P-03E ELUGA P P-03E for DoCoMo	**1** **(0.00%)**	100.00%	1 (0.00%)	100.00%	1.00	00:00:00	0.00%	0 (0.00%)	$0.00 (0.00%)
1354.	Pantech IM-A830L Vega Racer 2	**1** **(0.00%)**	100.00%	1 (0.00%)	0.00%	2.00	00:00:47	0.00%	0 (0.00%)	$0.00 (0.00%)
1355.	Pantech IM-A860S Vega No 6	**1** **(0.00%)**	100.00%	1 (0.00%)	100.00%	1.00	00:00:00	0.00%	0 (0.00%)	$0.00 (0.00%)
1356.	Pantech IM-A880S Vega LTE-A	**1** **(0.00%)**	100.00%	1 (0.00%)	100.00%	1.00	00:00:00	0.00%	0 (0.00%)	$0.00 (0.00%)
1357.	PendoPad PNDPP4MT9G2 4.0 Multi-Touch	**1** **(0.00%)**	100.00%	1 (0.00%)	100.00%	1.00	00:00:00	0.00%	0 (0.00%)	$0.00 (0.00%)

	9.7									
1358.	Pioneer E80w	**1** **(0.00%)**	100.00%	1 (0.00%)	100.00%	1.00	00:00:00	0.00%	0 (0.00%)	$0.00 (0.00%)
1359.	PROSCAN PLT7223G	**1** **(0.00%)**	100.00%	1 (0.00%)	100.00%	1.00	00:00:00	0.00%	0 (0.00%)	$0.00 (0.00%)
1360.	QMobile A8 Noir A8	**1** **(0.00%)**	100.00%	1 (0.00%)	0.00%	3.00	00:02:38	0.00%	0 (0.00%)	$0.00 (0.00%)
1361.	RCA RCT6378W2	**1** **(0.00%)**	100.00%	1 (0.00%)	100.00%	1.00	00:00:00	0.00%	0 (0.00%)	$0.00 (0.00%)
1362.	RIM Q10	**1** **(0.00%)**	100.00%	1 (0.00%)	0.00%	2.00	00:01:45	0.00%	0 (0.00%)	$0.00 (0.00%)
1363.	Samsung C5212	**1** **(0.00%)**	100.00%	1 (0.00%)	100.00%	1.00	00:00:00	0.00%	0 (0.00%)	$0.00 (0.00%)
1364.	Samsung EK-GC100 Galaxy Camera	**1** **(0.00%)**	100.00%	1 (0.00%)	0.00%	9.00	00:01:09	0.00%	0 (0.00%)	$0.00 (0.00%)
1365.	Samsung GP-P6810 Galaxy Tab 7.7	**1** **(0.00%)**	100.00%	1 (0.00%)	100.00%	1.00	00:00:00	0.00%	0 (0.00%)	$0.00 (0.00%)
1366.	Samsung GT I9000T Galaxy S	**1** **(0.00%)**	100.00%	1 (0.00%)	100.00%	1.00	00:00:00	0.00%	0 (0.00%)	$0.00 (0.00%)
1367.	Samsung GT-3770 Champ 3.5G	**1** **(0.00%)**	100.00%	1 (0.00%)	100.00%	1.00	00:00:00	0.00%	0 (0.00%)	$0.00 (0.00%)
1368.	Samsung GT-3770M Champ 3.5G	**1** **(0.00%)**	100.00%	1 (0.00%)	0.00%	2.00	00:01:10	0.00%	0 (0.00%)	$0.00 (0.00%)
1369.	Samsung GT-C3330	**1** **(0.00%)**	100.00%	1 (0.00%)	100.00%	1.00	00:00:00	0.00%	0 (0.00%)	$0.00 (0.00%)
1370.	Samsung GT-E3300L Manhattan	**1** **(0.00%)**	100.00%	1 (0.00%)	100.00%	1.00	00:00:00	0.00%	0 (0.00%)	$0.00 (0.00%)
1371.	Samsung GT-I5500L	**1** **(0.00%)**	100.00%	1 (0.00%)	0.00%	2.00	00:00:21	0.00%	0 (0.00%)	$0.00 (0.00%)
1372.	Samsung GT-I5510	**1** **(0.00%)**	100.00%	1 (0.00%)	100.00%	1.00	00:00:00	0.00%	0 (0.00%)	$0.00 (0.00%)
1373.	Samsung GT-I5510T	**1** **(0.00%)**	100.00%	1 (0.00%)	100.00%	1.00	00:00:00	0.00%	0 (0.00%)	$0.00 (0.00%)
1374.	Samsung GT-I5801 Galaxy Apollo	**1** **(0.00%)**	100.00%	1 (0.00%)	100.00%	1.00	00:00:00	0.00%	0 (0.00%)	$0.00 (0.00%)
1375.	Samsung GT-I9000 Galaxy S/Vibrant	**1** **(0.00%)**	100.00%	1 (0.00%)	100.00%	1.00	00:00:00	0.00%	0 (0.00%)	$0.00 (0.00%)
1376.	Samsung GT-I9100M Galaxy S II	**1** **(0.00%)**	100.00%	1 (0.00%)	0.00%	3.00	00:00:21	0.00%	0 (0.00%)	$0.00 (0.00%)
1377.	Samsung GT-I9150 Galaxy Mega 5.8	**1** **(0.00%)**	100.00%	1 (0.00%)	100.00%	1.00	00:00:00	0.00%	0 (0.00%)	$0.00 (0.00%)
1378.	Samsung GT-I9210 Galaxy SII LTE	**1** **(0.00%)**	100.00%	1 (0.00%)	0.00%	2.00	00:00:28	0.00%	0 (0.00%)	$0.00 (0.00%)
1379.	Samsung GT-I9220 Galaxy Note	**1** **(0.00%)**	100.00%	1 (0.00%)	100.00%	1.00	00:00:00	0.00%	0 (0.00%)	$0.00 (0.00%)
1380.	Samsung GT-I9305N Galaxy S3 LTE International	**1** **(0.00%)**	100.00%	1 (0.00%)	100.00%	1.00	00:00:00	0.00%	0 (0.00%)	$0.00 (0.00%)
1381.	Samsung GT-I9308 Galaxy S III	**1** **(0.00%)**	100.00%	1 (0.00%)	100.00%	1.00	00:00:00	0.00%	0 (0.00%)	$0.00 (0.00%)
1382.	Samsung GT-I9508 Galaxy S IV	**1** **(0.00%)**	100.00%	1 (0.00%)	100.00%	1.00	00:00:00	0.00%	0 (0.00%)	$0.00 (0.00%)

1383.	Samsung GT-P1000T Galaxy Tab	**1**	**(0.00%)**	100.00%	1	(0.00%)	0.00%	8.00	00:12:38	0.00%	0 (0.00%)	$0.00	(0.00%)
1384.	Samsung GT-P3100B Galaxy Tab 2 7.0	**1**	**(0.00%)**	100.00%	1	(0.00%)	100.00%	1.00	00:00:00	0.00%	0 (0.00%)	$0.00	(0.00%)
1385.	Samsung GT-S5250 Wave 2	**1**	**(0.00%)**	100.00%	1	(0.00%)	100.00%	1.00	00:00:00	0.00%	0 (0.00%)	$0.00	(0.00%)
1386.	Samsung GT-S5260 Star II	**1**	**(0.00%)**	100.00%	1	(0.00%)	100.00%	1.00	00:00:00	0.00%	0 (0.00%)	$0.00	(0.00%)
1387.	Samsung GT-S5270K Chat	**1**	**(0.00%)**	100.00%	1	(0.00%)	100.00%	1.00	00:00:00	0.00%	0 (0.00%)	$0.00	(0.00%)
1388.	Samsung GT-S5280 Galaxy Star	**1**	**(0.00%)**	100.00%	1	(0.00%)	100.00%	1.00	00:00:00	0.00%	0 (0.00%)	$0.00	(0.00%)
1389.	Samsung GT-S5310 Galaxy Pocket Neo	**1**	**(0.00%)**	100.00%	1	(0.00%)	100.00%	1.00	00:00:00	0.00%	0 (0.00%)	$0.00	(0.00%)
1390.	Samsung GT-S5312B Galaxy Pocket Neo	**1**	**(0.00%)**	100.00%	1	(0.00%)	0.00%	2.00	00:00:58	0.00%	0 (0.00%)	$0.00	(0.00%)
1391.	Samsung GT-S5330	**1**	**(0.00%)**	100.00%	1	(0.00%)	100.00%	1.00	00:00:00	0.00%	0 (0.00%)	$0.00	(0.00%)
1392.	Samsung GT-S5380K Wave Y	**1**	**(0.00%)**	100.00%	1	(0.00%)	100.00%	1.00	00:00:00	0.00%	0 (0.00%)	$0.00	(0.00%)
1393.	Samsung GT-S5670B Galaxy Fit	**1**	**(0.00%)**	100.00%	1	(0.00%)	0.00%	2.00	00:00:59	0.00%	0 (0.00%)	$0.00	(0.00%)
1394.	Samsung GT-S6500T Galaxy Mini 2	**1**	**(0.00%)**	100.00%	1	(0.00%)	100.00%	1.00	00:00:00	0.00%	0 (0.00%)	$0.00	(0.00%)
1395.	Samsung GT-S7272 Galaxy Ace 3	**1**	**(0.00%)**	100.00%	1	(0.00%)	0.00%	3.00	00:03:01	0.00%	0 (0.00%)	$0.00	(0.00%)
1396.	Samsung GT-S7273T Galaxy SII TV	**1**	**(0.00%)**	100.00%	1	(0.00%)	100.00%	1.00	00:00:00	0.00%	0 (0.00%)	$0.00	(0.00%)
1397.	Samsung GT-S7530E Omnia M	**1**	**(0.00%)**	100.00%	1	(0.00%)	0.00%	3.00	00:00:41	0.00%	0 (0.00%)	$0.00	(0.00%)
1398.	Samsung GT-S7568 Galaxy S Duos	**1**	**(0.00%)**	100.00%	1	(0.00%)	100.00%	1.00	00:00:00	0.00%	0 (0.00%)	$0.00	(0.00%)
1399.	Samsung GT-S8530 Wave 2	**1**	**(0.00%)**	100.00%	1	(0.00%)	100.00%	1.00	00:00:00	0.00%	0 (0.00%)	$0.00	(0.00%)
1400.	Samsung S3330 Ch@t	**1**	**(0.00%)**	100.00%	1	(0.00%)	100.00%	1.00	00:00:00	0.00%	0 (0.00%)	$0.00	(0.00%)
1401.	Samsung S5753E	**1**	**(0.00%)**	100.00%	1	(0.00%)	100.00%	1.00	00:00:00	0.00%	0 (0.00%)	$0.00	(0.00%)
1402.	Samsung SC-01C Galaxy Tab SC-01C for DoCoMo	**1**	**(0.00%)**	100.00%	1	(0.00%)	100.00%	1.00	00:00:00	0.00%	0 (0.00%)	$0.00	(0.00%)
1403.	Samsung SC-02E	**1**	**(0.00%)**	100.00%	1	(0.00%)	0.00%	2.00	00:00:11	0.00%	0 (0.00%)	$0.00	(0.00%)
1404.	Samsung SC-02E Galaxy Note II	**1**	**(0.00%)**	100.00%	1	(0.00%)	100.00%	1.00	00:00:00	0.00%	0 (0.00%)	$0.00	(0.00%)
1405.	Samsung SC-02F GALAXY J SC-02F for DoCoMo	**1**	**(0.00%)**	100.00%	1	(0.00%)	100.00%	1.00	00:00:00	0.00%	0 (0.00%)	$0.00	(0.00%)
1406.	Samsung SCH I869	**1**	**(0.00%)**	100.00%	1	(0.00%)	100.00%	1.00	00:00:00	0.00%	0 (0.00%)	$0.00	(0.00%)
1407.	Samsung SCH-i110 Illusion	**1**	**(0.00%)**	100.00%	1	(0.00%)	100.00%	1.00	00:00:00	0.00%	0 (0.00%)	$0.00	(0.00%)

1408.	Samsung SCH-I535 4G Galaxy S III	**1**	**(0.00%)**	100.00%	1	(0.00%)	0.00%	3.00	00:01:30	0.00%	0	(0.00%)	$0.00	(0.00%)
1409.	Samsung SCH-I739 Trend II	**1**	**(0.00%)**	100.00%	1	(0.00%)	100.00%	1.00	00:00:00	0.00%	0	(0.00%)	$0.00	(0.00%)
1410.	Samsung SCH-I779	**1**	**(0.00%)**	100.00%	1	(0.00%)	100.00%	1.00	00:00:00	0.00%	0	(0.00%)	$0.00	(0.00%)
1411.	Samsung SCH-I939 Galaxy SIII	**1**	**(0.00%)**	100.00%	1	(0.00%)	100.00%	1.00	00:00:00	0.00%	0	(0.00%)	$0.00	(0.00%)
1412.	Samsung SCH-R530C Galaxy SIII	**1**	**(0.00%)**	100.00%	1	(0.00%)	100.00%	1.00	00:00:00	0.00%	0	(0.00%)	$0.00	(0.00%)
1413.	Samsung SCH-R530M Galaxy SIII	**1**	**(0.00%)**	100.00%	1	(0.00%)	100.00%	1.00	00:00:00	0.00%	0	(0.00%)	$0.00	(0.00%)
1414.	Samsung SCH-R530X Galaxy S III	**1**	**(0.00%)**	100.00%	1	(0.00%)	100.00%	1.00	00:00:00	0.00%	0	(0.00%)	$0.00	(0.00%)
1415.	Samsung SCH-R680 Repp	**1**	**(0.00%)**	100.00%	1	(0.00%)	100.00%	1.00	00:00:00	0.00%	0	(0.00%)	$0.00	(0.00%)
1416.	Samsung SCH-R930 Galaxy S Aviator	**1**	**(0.00%)**	100.00%	1	(0.00%)	100.00%	1.00	00:00:00	0.00%	0	(0.00%)	$0.00	(0.00%)
1417.	Samsung SGH A667	**1**	**(0.00%)**	100.00%	1	(0.00%)	0.00%	6.00	00:05:29	0.00%	0	(0.00%)	$0.00	(0.00%)
1418.	Samsung SGH i900 OMNIA	**1**	**(0.00%)**	100.00%	1	(0.00%)	0.00%	2.00	00:04:31	0.00%	0	(0.00%)	$0.00	(0.00%)
1419.	Samsung SGH-I437 Galaxy Express	**1**	**(0.00%)**	100.00%	1	(0.00%)	100.00%	1.00	00:00:00	0.00%	0	(0.00%)	$0.00	(0.00%)
1420.	Samsung SGH-I467M Galaxy Note 8.0	**1**	**(0.00%)**	100.00%	1	(0.00%)	100.00%	1.00	00:00:00	0.00%	0	(0.00%)	$0.00	(0.00%)
1421.	Samsung SGH-i717D Galaxy Note	**1**	**(0.00%)**	100.00%	1	(0.00%)	0.00%	3.00	00:04:21	0.00%	0	(0.00%)	$0.00	(0.00%)
1422.	Samsung SGH-I757M Galaxy SII HD LTE	**1**	**(0.00%)**	100.00%	1	(0.00%)	100.00%	1.00	00:00:00	0.00%	0	(0.00%)	$0.00	(0.00%)
1423.	Samsung SGH-I896 Galaxy S Captivate	**1**	**(0.00%)**	100.00%	1	(0.00%)	100.00%	1.00	00:00:00	0.00%	0	(0.00%)	$0.00	(0.00%)
1424.	Samsung SGH-I957 Galaxy Tab 8.9 LTE	**1**	**(0.00%)**	100.00%	1	(0.00%)	100.00%	1.00	00:00:00	0.00%	0	(0.00%)	$0.00	(0.00%)
1425.	Samsung SGH-T889V Galaxy Note II	**1**	**(0.00%)**	100.00%	1	(0.00%)	100.00%	1.00	00:00:00	0.00%	0	(0.00%)	$0.00	(0.00%)
1426.	Samsung SGH-T959 Vibrant	**1**	**(0.00%)**	100.00%	1	(0.00%)	0.00%	5.00	00:01:03	0.00%	0	(0.00%)	$0.00	(0.00%)
1427.	Samsung SGH-T959P Vibrant	**1**	**(0.00%)**	100.00%	1	(0.00%)	100.00%	1.00	00:00:00	0.00%	0	(0.00%)	$0.00	(0.00%)
1428.	Samsung SGH-T999V Galaxy S III	**1**	**(0.00%)**	100.00%	1	(0.00%)	100.00%	1.00	00:00:00	0.00%	0	(0.00%)	$0.00	(0.00%)
1429.	Samsung SHV-E120L Galaxy S II HD	**1**	**(0.00%)**	100.00%	1	(0.00%)	100.00%	1.00	00:00:00	0.00%	0	(0.00%)	$0.00	(0.00%)
1430.	Samsung SHV-E120S Galaxy S II HD	**1**	**(0.00%)**	100.00%	1	(0.00%)	0.00%	3.00	00:01:37	0.00%	0	(0.00%)	$0.00	(0.00%)
1431.	Samsung SHV-E210K Galaxy S III	**1**	**(0.00%)**	100.00%	1	(0.00%)	100.00%	1.00	00:00:00	0.00%	0	(0.00%)	$0.00	(0.00%)
	Samsung SHV-													

1432.	E210K Galaxy SIII	**1** **(0.00%)**	0.00%	0 (0.00%)	0.00%	11.00	00:03:02	0.00%	0 (0.00%)	$0.00 (0.00%)
1433.	Samsung SHV-E220S Galaxy Pop	**1** **(0.00%)**	100.00%	1 (0.00%)	100.00%	1.00	00:00:00	0.00%	0 (0.00%)	$0.00 (0.00%)
1434.	Samsung SHV-E250L Galaxy Note II	**1** **(0.00%)**	100.00%	1 (0.00%)	100.00%	1.00	00:00:00	0.00%	0 (0.00%)	$0.00 (0.00%)
1435.	Samsung SHV-E300K Galaxy S IV	**1** **(0.00%)**	100.00%	1 (0.00%)	100.00%	1.00	00:00:00	0.00%	0 (0.00%)	$0.00 (0.00%)
1436.	Samsung SHV-E300S Galaxy S IV	**1** **(0.00%)**	100.00%	1 (0.00%)	100.00%	1.00	00:00:00	0.00%	0 (0.00%)	$0.00 (0.00%)
1437.	Samsung SHV-E310S Galaxy Mega 6.3	**1** **(0.00%)**	100.00%	1 (0.00%)	100.00%	1.00	00:00:00	0.00%	0 (0.00%)	$0.00 (0.00%)
1438.	Samsung SHW-M110S Galaxy S	**1** **(0.00%)**	100.00%	1 (0.00%)	0.00%	9.00	00:03:50	0.00%	0 (0.00%)	$0.00 (0.00%)
1439.	Samsung SHW-M250L GALAXY S II (LG U+)	**1** **(0.00%)**	100.00%	1 (0.00%)	0.00%	2.00	00:00:27	0.00%	0 (0.00%)	$0.00 (0.00%)
1440.	Samsung SHW-M380S Galaxy Tab 10.1	**1** **(0.00%)**	0.00%	0 (0.00%)	100.00%	1.00	00:00:00	0.00%	0 (0.00%)	$0.00 (0.00%)
1441.	Samsung SHW-M440S Galaxy S III	**1** **(0.00%)**	0.00%	0 (0.00%)	0.00%	7.00	00:10:25	0.00%	0 (0.00%)	$0.00 (0.00%)
1442.	Samsung SM-G900H Galaxy S5	**1** **(0.00%)**	100.00%	1 (0.00%)	100.00%	1.00	00:00:00	0.00%	0 (0.00%)	$0.00 (0.00%)
1443.	Samsung SM-G900S Galaxy S5	**1** **(0.00%)**	100.00%	1 (0.00%)	0.00%	5.00	00:01:39	0.00%	0 (0.00%)	$0.00 (0.00%)
1444.	Samsung SM-N900S Galaxy Note 3	**1** **(0.00%)**	100.00%	1 (0.00%)	100.00%	1.00	00:00:00	0.00%	0 (0.00%)	$0.00 (0.00%)
1445.	Samsung SPH-M580 Replenish	**1** **(0.00%)**	100.00%	1 (0.00%)	100.00%	1.00	00:00:00	0.00%	0 (0.00%)	$0.00 (0.00%)
1446.	Samsung SPH-M910 Intercept	**1** **(0.00%)**	100.00%	1 (0.00%)	100.00%	1.00	00:00:00	0.00%	0 (0.00%)	$0.00 (0.00%)
1447.	Sanei N10-QM	**1** **(0.00%)**	100.00%	1 (0.00%)	0.00%	7.00	00:13:07	0.00%	0 (0.00%)	$0.00 (0.00%)
1448.	Sharp 007SH AQUOS Phone	**1** **(0.00%)**	100.00%	1 (0.00%)	100.00%	1.00	00:00:00	0.00%	0 (0.00%)	$0.00 (0.00%)
1449.	Sharp 204SH	**1** **(0.00%)**	100.00%	1 (0.00%)	100.00%	1.00	00:00:00	0.00%	0 (0.00%)	$0.00 (0.00%)
1450.	Sharp ADS1 FX Plus	**1** **(0.00%)**	100.00%	1 (0.00%)	100.00%	1.00	00:00:00	0.00%	0 (0.00%)	$0.00 (0.00%)
1451.	Sharp IS03 IS03 for KDDI	**1** **(0.00%)**	100.00%	1 (0.00%)	100.00%	1.00	00:00:00	0.00%	0 (0.00%)	$0.00 (0.00%)
1452.	Sharp IS11SH AQUOS PHONE IS11SH for KDDI	**1** **(0.00%)**	100.00%	1 (0.00%)	100.00%	1.00	00:00:00	0.00%	0 (0.00%)	$0.00 (0.00%)
1453.	Sharp SH-13C AQUOS PHONE f SH-13C for DoCoMo	**1** **(0.00%)**	100.00%	1 (0.00%)	100.00%	1.00	00:00:00	0.00%	0 (0.00%)	$0.00 (0.00%)
1454.	Simvalley SPX-5 3G	**1** **(0.00%)**	100.00%	1 (0.00%)	100.00%	1.00	00:00:00	0.00%	0 (0.00%)	$0.00 (0.00%)
1455.	Sky IM-A800S Vega LTE	**1** **(0.00%)**	100.00%	1 (0.00%)	100.00%	1.00	00:00:00	0.00%	0 (0.00%)	$0.00 (0.00%)
1456.	Smartfren S7 AndroTab S7	**1** **(0.00%)**	100.00%	1 (0.00%)	0.00%	3.00	00:03:36	0.00%	0 (0.00%)	$0.00 (0.00%)
1457.	Softbank 001DL Dell Streak 5	**1** **(0.00%)**	100.00%	1 (0.00%)	0.00%	3.00	00:00:23	0.00%	0 (0.00%)	$0.00 (0.00%)

1458.	Softbank SBM107SH Pantone 5 107SH	**1**	**(0.00%)**	100.00%	1	(0.00%)	100.00%	1.00	00:00:00	0.00%	0	(0.00%)	$0.00	(0.00%)
1459.	SoftwinerEvb Tat	**1**	**(0.00%)**	100.00%	1	(0.00%)	100.00%	1.00	00:00:00	0.00%	0	(0.00%)	$0.00	(0.00%)
1460.	Sony D6503 Xperia Z2	**1**	**(0.00%)**	100.00%	1	(0.00%)	100.00%	1.00	00:00:00	0.00%	0	(0.00%)	$0.00	(0.00%)
1461.	Sony Playstation 3	**1**	**(0.00%)**	100.00%	1	(0.00%)	0.00%	6.00	00:02:49	0.00%	0	(0.00%)	$0.00	(0.00%)
1462.	Sony PlayStation Portable PSP	**1**	**(0.00%)**	100.00%	1	(0.00%)	100.00%	1.00	00:00:00	0.00%	0	(0.00%)	$0.00	(0.00%)
1463.	Sony SGPT13 Xperia Tablet S	**1**	**(0.00%)**	100.00%	1	(0.00%)	100.00%	1.00	00:00:00	0.00%	0	(0.00%)	$0.00	(0.00%)
1464.	Sony SOL21 Xperia VL SOL21 for KDDI	**1**	**(0.00%)**	100.00%	1	(0.00%)	100.00%	1.00	00:00:00	0.00%	0	(0.00%)	$0.00	(0.00%)
1465.	Sony SOL24 Xperia Z Ultra SOL24 for KDDI	**1**	**(0.00%)**	100.00%	1	(0.00%)	100.00%	1.00	00:00:00	0.00%	0	(0.00%)	$0.00	(0.00%)
1466.	Sony ST21a2 Xperia Tipo Dual	**1**	**(0.00%)**	100.00%	1	(0.00%)	100.00%	1.00	00:00:00	0.00%	0	(0.00%)	$0.00	(0.00%)
1467.	SonyEricsson F100i	**1**	**(0.00%)**	100.00%	1	(0.00%)	100.00%	1.00	00:00:00	0.00%	0	(0.00%)	$0.00	(0.00%)
1468.	SonyEricsson IS12S	**1**	**(0.00%)**	100.00%	1	(0.00%)	100.00%	1.00	00:00:00	0.00%	0	(0.00%)	$0.00	(0.00%)
1469.	SonyEricsson J10i2 Elm	**1**	**(0.00%)**	100.00%	1	(0.00%)	0.00%	3.00	00:01:50	0.00%	0	(0.00%)	$0.00	(0.00%)
1470.	SonyEricsson J20i	**1**	**(0.00%)**	100.00%	1	(0.00%)	0.00%	8.00	00:08:40	0.00%	0	(0.00%)	$0.00	(0.00%)
1471.	SonyEricsson LT15a Xperia Arc	**1**	**(0.00%)**	100.00%	1	(0.00%)	100.00%	1.00	00:00:00	0.00%	0	(0.00%)	$0.00	(0.00%)
1472.	SonyEricsson R800x Xperia PLAY	**1**	**(0.00%)**	100.00%	1	(0.00%)	100.00%	1.00	00:00:00	0.00%	0	(0.00%)	$0.00	(0.00%)
1473.	SonyEricsson SO-01B Xperia SO-01B for DoCoMo	**1**	**(0.00%)**	100.00%	1	(0.00%)	100.00%	1.00	00:00:00	0.00%	0	(0.00%)	$0.00	(0.00%)
1474.	SonyEricsson SO-02C Xperia acro SO-02C for DoCoMo	**1**	**(0.00%)**	100.00%	1	(0.00%)	100.00%	1.00	00:00:00	0.00%	0	(0.00%)	$0.00	(0.00%)
1475.	SonyEricsson SO-02D Xperia TM NX SO-02D	**1**	**(0.00%)**	100.00%	1	(0.00%)	100.00%	1.00	00:00:00	0.00%	0	(0.00%)	$0.00	(0.00%)
1476.	SonyEricsson ST17a Xperia Active	**1**	**(0.00%)**	100.00%	1	(0.00%)	0.00%	5.00	00:07:48	0.00%	0	(0.00%)	$0.00	(0.00%)
1477.	SonyEricsson ST17i Xperia Active	**1**	**(0.00%)**	100.00%	1	(0.00%)	100.00%	1.00	00:00:00	0.00%	0	(0.00%)	$0.00	(0.00%)
1478.	SonyEricsson T700	**1**	**(0.00%)**	100.00%	1	(0.00%)	100.00%	1.00	00:00:00	0.00%	0	(0.00%)	$0.00	(0.00%)
1479.	SonyEricsson U1 Satio	**1**	**(0.00%)**	100.00%	1	(0.00%)	100.00%	1.00	00:00:00	0.00%	0	(0.00%)	$0.00	(0.00%)
1480.	SonyEricsson U20i	**1**	**(0.00%)**	100.00%	1	(0.00%)	100.00%	1.00	00:00:00	0.00%	0	(0.00%)	$0.00	(0.00%)
1481.	SonyEricsson U5iv Vivaz	**1**	**(0.00%)**	100.00%	1	(0.00%)	100.00%	1.00	00:00:00	0.00%	0	(0.00%)	$0.00	(0.00%)
1482.	SonyEricsson W910i	**1**	**(0.00%)**	100.00%	1	(0.00%)	0.00%	2.00	00:00:30	0.00%	0	(0.00%)	$0.00	(0.00%)
1483.	T-Mobile myTouch	**1**	**(0.00%)**	100.00%	1	(0.00%)	100.00%	1.00	00:00:00	0.00%	0	(0.00%)	$0.00	(0.00%)

1484.	T-Mobile U8651T Prism	**1** **(0.00%)**	100.00%	1 (0.00%)	100.00%	1.00	00:00:00	0.00%	0 (0.00%)	$0.00 (0.00%)
1485.	TCL A510	**1** **(0.00%)**	100.00%	1 (0.00%)	100.00%	1.00	00:00:00	0.00%	0 (0.00%)	$0.00 (0.00%)
1486.	Tecno P3	**1** **(0.00%)**	100.00%	1 (0.00%)	100.00%	1.00	00:00:00	0.00%	0 (0.00%)	$0.00 (0.00%)
1487.	ThL W100	**1** **(0.00%)**	0.00%	0 (0.00%)	0.00%	6.00	00:01:46	0.00%	0 (0.00%)	$0.00 (0.00%)
1488.	Toshiba AT10LE-A Excite Pro	**1** **(0.00%)**	100.00%	1 (0.00%)	100.00%	1.00	00:00:00	0.00%	0 (0.00%)	$0.00 (0.00%)
1489.	TrekStor ST701041 SurfTab Breeze 7.0	**1** **(0.00%)**	100.00%	1 (0.00%)	0.00%	7.00	00:03:47	0.00%	0 (0.00%)	$0.00 (0.00%)
1490.	Turkcell T21 Max Plus 5	**1** **(0.00%)**	100.00%	1 (0.00%)	100.00%	1.00	00:00:00	0.00%	0 (0.00%)	$0.00 (0.00%)
1491.	Versus TouchPad 7	**1** **(0.00%)**	100.00%	1 (0.00%)	100.00%	1.00	00:00:00	0.00%	0 (0.00%)	$0.00 (0.00%)
1492.	Vivo S6t	**1** **(0.00%)**	100.00%	1 (0.00%)	100.00%	1.00	00:00:00	0.00%	0 (0.00%)	$0.00 (0.00%)
1493.	Vivo X510t	**1** **(0.00%)**	100.00%	1 (0.00%)	100.00%	1.00	00:00:00	0.00%	0 (0.00%)	$0.00 (0.00%)
1494.	Vodafone Smart Tab 10	**1** **(0.00%)**	100.00%	1 (0.00%)	100.00%	1.00	00:00:00	0.00%	0 (0.00%)	$0.00 (0.00%)
1495.	Vodafone Smart Tab III 10	**1** **(0.00%)**	100.00%	1 (0.00%)	100.00%	1.00	00:00:00	0.00%	0 (0.00%)	$0.00 (0.00%)
1496.	Wiko Cink Peax	**1** **(0.00%)**	100.00%	1 (0.00%)	100.00%	1.00	00:00:00	0.00%	0 (0.00%)	$0.00 (0.00%)
1497.	Wiko Cink Slim	**1** **(0.00%)**	100.00%	1 (0.00%)	100.00%	1.00	00:00:00	0.00%	0 (0.00%)	$0.00 (0.00%)
1498.	Xiaomi MI 2A	**1** **(0.00%)**	100.00%	1 (0.00%)	100.00%	1.00	00:00:00	0.00%	0 (0.00%)	$0.00 (0.00%)
1499.	Zopo C2	**1** **(0.00%)**	0.00%	0 (0.00%)	0.00%	3.00	00:00:41	0.00%	0 (0.00%)	$0.00 (0.00%)
1500.	ZTE Blade	**1** **(0.00%)**	100.00%	1 (0.00%)	100.00%	1.00	00:00:00	0.00%	0 (0.00%)	$0.00 (0.00%)
1501.	ZTE BM-LTBU300 Light Tab	**1** **(0.00%)**	100.00%	1 (0.00%)	100.00%	1.00	00:00:00	0.00%	0 (0.00%)	$0.00 (0.00%)
1502.	ZTE Corporation ZTE N855D	**1** **(0.00%)**	100.00%	1 (0.00%)	100.00%	1.00	00:00:00	0.00%	0 (0.00%)	$0.00 (0.00%)
1503.	ZTE N721 Personal Touch	**1** **(0.00%)**	100.00%	1 (0.00%)	0.00%	2.00	00:21:12	0.00%	0 (0.00%)	$0.00 (0.00%)
1504.	ZTE Open	**1** **(0.00%)**	100.00%	1 (0.00%)	0.00%	3.00	00:00:38	0.00%	0 (0.00%)	$0.00 (0.00%)
1505.	ZTE TU812	**1** **(0.00%)**	100.00%	1 (0.00%)	100.00%	1.00	00:00:00	0.00%	0 (0.00%)	$0.00 (0.00%)
1506.	ZTE V880 Blade	**1** **(0.00%)**	100.00%	1 (0.00%)	100.00%	1.00	00:00:00	0.00%	0 (0.00%)	$0.00 (0.00%)
1507.	ZTE V889S	**1** **(0.00%)**	100.00%	1 (0.00%)	100.00%	1.00	00:00:00	0.00%	0 (0.00%)	$0.00 (0.00%)
1508.	ZTE V967S	**1** **(0.00%)**	100.00%	1 (0.00%)	100.00%	1.00	00:00:00	0.00%	0 (0.00%)	$0.00 (0.00%)
1509.	ZTE ZTE LEO Q1	**1** **(0.00%)**	100.00%	1 (0.00%)	0.00%	4.00	00:03:21	0.00%	0 (0.00%)	$0.00 (0.00%)

Rows 1 - 1509 of 1509

© 2014 Google
