# Supplementary material for: Trends in access of plant biodiversity data revealed by Google Analytics
Source: Biodivers Data J. 2014 Nov 11;(2):e1558. doi: 10.3897/BDJ.2.e1558 (PMC4238075; doi:10.3897/BDJ.2.e1558)
Supplement: Supplementary material 11 — Five top language percentages at Tropicos over six years [file biodiversity_data_journal-2-e1558-s011.pdf]

Language

Jun 1, 2007 - Jun 1, 2014

All Sessions
100.00%

+ Add Segment

Explorer

Summary

Language	Acquisition			Behavior			Conversions		
	Sessions	% New Sessions	New Users	Bounce Rate	Pages / Session	Avg. Session Duration	Goal Conversion Rate	Goal Completions	Goal Value
	7,549,849 % of Total: 100.00% (7,549,849)	29.24% Site Avg: 29.19% (0.18%)	2,207,842 % of Total: 100.18% (2,203,942)	30.70% Site Avg: 30.70% (0.00%)	12.64 Site Avg: 12.64 (0.00%)	00:12:58 Site Avg: 00:12:58 (0.00%)	0.00% Site Avg: 0.00% (0.00%)	0 % of Total: 0.00% (0)	$0.00 % of Total: 0.00% ($0.00)
1. en-us	**2,676,116** **(35.45%)**	31.95%	854,906 (38.72%)	34.42%	12.51	00:12:05	0.00%	0 (0.00%)	$0.00 (0.00%)
2. es	**1,358,102** **(17.99%)**	25.85%	351,061 (15.90%)	24.63%	15.15	00:15:29	0.00%	0 (0.00%)	$0.00 (0.00%)
3. pt-br	**951,149** **(12.60%)**	26.58%	252,854 (11.45%)	23.39%	11.58	00:12:52	0.00%	0 (0.00%)	$0.00 (0.00%)
4. es-es	**572,127** **(7.58%)**	24.70%	141,318 (6.40%)	34.91%	13.65	00:13:37	0.00%	0 (0.00%)	$0.00 (0.00%)
5. fr	**406,307** **(5.38%)**	25.75%	104,607 (4.74%)	24.83%	14.31	00:17:05	0.00%	0 (0.00%)	$0.00 (0.00%)
6. de	**185,327** **(2.45%)**	32.09%	59,479 (2.69%)	32.11%	10.08	00:11:48	0.00%	0 (0.00%)	$0.00 (0.00%)
7. en-gb	**146,759** **(1.94%)**	29.34%	43,058 (1.95%)	34.11%	9.90	00:10:36	0.00%	0 (0.00%)	$0.00 (0.00%)
8. zh-cn	**143,763** **(1.90%)**	22.98%	33,033 (1.50%)	21.87%	12.67	00:14:36	0.00%	0 (0.00%)	$0.00 (0.00%)
9. de-de	**115,500** **(1.53%)**	36.09%	41,682 (1.89%)	36.87%	9.18	00:10:20	0.00%	0 (0.00%)	$0.00 (0.00%)
10. es-419	**90,453** **(1.20%)**	26.97%	24,397 (1.11%)	25.36%	14.57	00:14:39	0.00%	0 (0.00%)	$0.00 (0.00%)
11. ru	**77,856** **(1.03%)**	35.34%	27,516 (1.25%)	43.24%	7.45	00:08:32	0.00%	0 (0.00%)	$0.00 (0.00%)
12. en	**75,305** **(1.00%)**	36.35%	27,371 (1.24%)	46.54%	8.21	00:09:07	0.00%	0 (0.00%)	$0.00 (0.00%)
13. zh-tw	**67,031** **(0.89%)**	21.98%	14,733 (0.67%)	32.07%	8.11	00:10:56	0.00%	0 (0.00%)	$0.00 (0.00%)
14. it	**55,897** **(0.74%)**	36.84%	20,591 (0.93%)	32.96%	12.27	00:11:00	0.00%	0 (0.00%)	$0.00 (0.00%)
15. es-mx	**54,383** **(0.72%)**	26.53%	14,430 (0.65%)	19.03%	17.51	00:16:58	0.00%	0 (0.00%)	$0.00 (0.00%)
16. ja	**48,617** **(0.64%)**	31.52%	15,326 (0.69%)	29.32%	11.57	00:08:41	0.00%	0 (0.00%)	$0.00 (0.00%)
17. ko	**46,808** **(0.62%)**	23.71%	11,098 (0.50%)	22.98%	12.17	00:13:44	0.00%	0 (0.00%)	$0.00 (0.00%)
18. pl	**45,725** **(0.61%)**	31.41%	14,362 (0.65%)	40.20%	10.54	00:09:51	0.00%	0 (0.00%)	$0.00 (0.00%)
19. es-ar	**44,511** **(0.59%)**	26.26%	11,689 (0.53%)	21.55%	14.85	00:15:34	0.00%	0 (0.00%)	$0.00 (0.00%)
20. nl	**44,299** **(0.59%)**	38.32%	16,974 (0.77%)	39.96%	10.06	00:08:33	0.00%	0 (0.00%)	$0.00 (0.00%)
21. fr-fr	**38,794** **(0.51%)**	24.49%	9,500 (0.43%)	47.23%	9.05	00:07:43	0.00%	0 (0.00%)	$0.00 (0.00%)
22. cs	**30,899** **(0.41%)**	34.23%	10,578 (0.48%)	40.00%	10.46	00:09:07	0.00%	0 (0.00%)	$0.00 (0.00%)
23. it-it	**19,614** **(0.26%)**	40.79%	8,000 (0.36%)	43.66%	7.59	00:07:14	0.00%	0 (0.00%)	$0.00 (0.00%)
24. sv-se	**19,222** **(0.25%)**	16.59%	3,189 (0.14%)	27.93%	8.40	00:10:42	0.00%	0 (0.00%)	$0.00 (0.00%)
25. pt-pt	**17,567** **(0.23%)**	35.95%	6,315 (0.29%)	34.37%	9.09	00:09:12	0.00%	0 (0.00%)	$0.00 (0.00%)
26. ru-ru	**17,534** **(0.23%)**	28.54%	5,005 (0.23%)	40.55%	7.65	00:08:04	0.00%	0 (0.00%)	$0.00 (0.00%)

27.	sv	**15,208**	**(0.20%)**	28.25%	4,296	(0.19%)	30.44%	9.55	00:11:19	0.00%	0	(0.00%)	$0.00	(0.00%)
28.	tr	**13,630**	**(0.18%)**	52.83%	7,201	(0.33%)	42.65%	8.91	00:06:35	0.00%	0	(0.00%)	$0.00	(0.00%)
29.	ja-jp	**13,236**	**(0.18%)**	27.45%	3,633	(0.16%)	39.77%	7.52	00:06:39	0.00%	0	(0.00%)	$0.00	(0.00%)
30.	pt	**8,970**	**(0.12%)**	45.31%	4,064	(0.18%)	33.57%	11.34	00:10:35	0.00%	0	(0.00%)	$0.00	(0.00%)
31.	sk	**8,524**	**(0.11%)**	29.22%	2,491	(0.11%)	43.02%	8.33	00:08:29	0.00%	0	(0.00%)	$0.00	(0.00%)
32.	ca	**7,849**	**(0.10%)**	34.37%	2,698	(0.12%)	25.39%	11.04	00:15:17	0.00%	0	(0.00%)	$0.00	(0.00%)
33.	da	**7,532**	**(0.10%)**	44.54%	3,355	(0.15%)	39.66%	7.15	00:06:15	0.00%	0	(0.00%)	$0.00	(0.00%)
34.	ko-kr	**7,336**	**(0.10%)**	21.76%	1,596	(0.07%)	26.80%	13.57	00:14:23	0.00%	0	(0.00%)	$0.00	(0.00%)
35.	th	**6,792**	**(0.09%)**	47.44%	3,222	(0.15%)	47.47%	5.65	00:06:29	0.00%	0	(0.00%)	$0.00	(0.00%)
36.	id	**6,389**	**(0.08%)**	56.58%	3,615	(0.16%)	51.56%	5.79	00:08:21	0.00%	0	(0.00%)	$0.00	(0.00%)
37.	vi	**6,203**	**(0.08%)**	48.67%	3,019	(0.14%)	42.14%	6.88	00:07:52	0.00%	0	(0.00%)	$0.00	(0.00%)
38.	hu	**5,588**	**(0.07%)**	65.37%	3,653	(0.17%)	60.79%	3.87	00:02:36	0.00%	0	(0.00%)	$0.00	(0.00%)
39.	nl-nl	**5,103**	**(0.07%)**	50.44%	2,574	(0.12%)	58.16%	4.87	00:03:10	0.00%	0	(0.00%)	$0.00	(0.00%)
40.	es-la	**4,811**	**(0.06%)**	21.10%	1,015	(0.05%)	41.88%	11.48	00:11:36	0.00%	0	(0.00%)	$0.00	(0.00%)
41.	el	**4,350**	**(0.06%)**	58.83%	2,559	(0.12%)	53.47%	4.46	00:03:02	0.00%	0	(0.00%)	$0.00	(0.00%)
42.	es-cl	**3,717**	**(0.05%)**	38.04%	1,414	(0.06%)	28.36%	9.32	00:10:23	0.00%	0	(0.00%)	$0.00	(0.00%)
43.	es-xl	**3,447**	**(0.05%)**	5.66%	195	(0.01%)	55.61%	8.28	00:08:22	0.00%	0	(0.00%)	$0.00	(0.00%)
44.	fi	**3,286**	**(0.04%)**	48.60%	1,597	(0.07%)	40.38%	9.25	00:05:42	0.00%	0	(0.00%)	$0.00	(0.00%)
45.	nb-no	**2,923**	**(0.04%)**	26.99%	789	(0.04%)	45.77%	12.28	00:08:11	0.00%	0	(0.00%)	$0.00	(0.00%)
46.	tr-tr	**2,884**	**(0.04%)**	44.17%	1,274	(0.06%)	48.37%	7.97	00:06:02	0.00%	0	(0.00%)	$0.00	(0.00%)
47.	th-th	**2,844**	**(0.04%)**	36.71%	1,044	(0.05%)	44.83%	5.42	00:06:15	0.00%	0	(0.00%)	$0.00	(0.00%)
48.	hu-hu	**2,816**	**(0.04%)**	63.99%	1,802	(0.08%)	63.85%	3.09	00:01:58	0.00%	0	(0.00%)	$0.00	(0.00%)
49.	fr-ca	**2,746**	**(0.04%)**	4.04%	111	(0.01%)	9.65%	71.40	00:59:13	0.00%	0	(0.00%)	$0.00	(0.00%)
50.	ja-jp-mac	**2,671**	**(0.04%)**	19.43%	519	(0.02%)	16.02%	29.11	00:16:30	0.00%	0	(0.00%)	$0.00	(0.00%)
51.	ar	**2,407**	**(0.03%)**	59.41%	1,430	(0.06%)	57.96%	3.48	00:03:30	0.00%	0	(0.00%)	$0.00	(0.00%)
52.	lt	**2,331**	**(0.03%)**	41.57%	969	(0.04%)	43.07%	9.90	00:10:22	0.00%	0	(0.00%)	$0.00	(0.00%)
53.	fi-fi	**2,318**	**(0.03%)**	40.08%	929	(0.04%)	43.70%	6.98	00:07:07	0.00%	0	(0.00%)	$0.00	(0.00%)
54.	zh-hk	**2,300**	**(0.03%)**	35.17%	809	(0.04%)	35.22%	7.16	00:05:24	0.00%	0	(0.00%)	$0.00	(0.00%)
55.	no	**2,247**	**(0.03%)**	40.10%	901	(0.04%)	36.98%	10.29	00:07:33	0.00%	0	(0.00%)	$0.00	(0.00%)
56.	et	**2,239**	**(0.03%)**	41.80%	936	(0.04%)	38.28%	5.85	00:06:03	0.00%	0	(0.00%)	$0.00	(0.00%)
57.	en-au	**2,184**	**(0.03%)**	25.18%	550	(0.02%)	30.13%	6.77	00:08:19	0.00%	0	(0.00%)	$0.00	(0.00%)
58.	bg	**2,137**	**(0.03%)**	65.70%	1,404	(0.06%)	66.31%	3.68	00:02:22	0.00%	0	(0.00%)	$0.00	(0.00%)
59.	cs-cz	**2,097**	**(0.03%)**	32.09%	673	(0.03%)	33.33%	12.36	00:12:18	0.00%	0	(0.00%)	$0.00	(0.00%)
60.	es-us	**1,828**	**(0.02%)**	50.16%	917	(0.04%)	46.61%	5.71	00:06:23	0.00%	0	(0.00%)	$0.00	(0.00%)
61.	el-gr	**1,725**	**(0.02%)**	65.33%	1,127	(0.05%)	59.94%	4.77	00:03:22	0.00%	0	(0.00%)	$0.00	(0.00%)
62.	ro	**1,691**	**(0.02%)**	64.34%	1,088	(0.05%)	58.55%	5.78	00:04:12	0.00%	0	(0.00%)	$0.00	(0.00%)
63.	he	**1,643**	**(0.02%)**	67.32%	1,106	(0.05%)	57.21%	4.26	00:02:38	0.00%	0	(0.00%)	$0.00	(0.00%)
64.	fil	**1,605**	**(0.02%)**	70.03%	1,124	(0.05%)	61.74%	2.61	00:02:48	0.00%	0	(0.00%)	$0.00	(0.00%)
65.	de-at	**1,559**	**(0.02%)**	49.90%	778	(0.04%)	14.75%	10.25	00:14:58	0.00%	0	(0.00%)	$0.00	(0.00%)
66.	vi-vn	**1,375**	**(0.02%)**	53.24%	732	(0.03%)	51.27%	5.26	00:04:56	0.00%	0	(0.00%)	$0.00	(0.00%)

67.	sl	**1,343** **(0.02%)**	62.92%	845 (0.04%)	63.89%	3.09	00:01:47	0.00%	0 (0.00%)	$0.00 (0.00%)
68.	da-dk	**1,233** **(0.02%)**	54.58%	673 (0.03%)	64.96%	3.64	00:02:34	0.00%	0 (0.00%)	$0.00 (0.00%)
69.	uk	**1,168** **(0.02%)**	58.65%	685 (0.03%)	52.31%	5.44	00:04:39	0.00%	0 (0.00%)	$0.00 (0.00%)
70.	lv	**976** **(0.01%)**	51.43%	502 (0.02%)	50.72%	10.35	00:06:58	0.00%	0 (0.00%)	$0.00 (0.00%)
71.	hr	**900** **(0.01%)**	72.11%	649 (0.03%)	62.67%	4.63	00:03:05	0.00%	0 (0.00%)	$0.00 (0.00%)
72.	pl-pl	**822** **(0.01%)**	64.48%	530 (0.02%)	63.87%	6.42	00:02:36	0.00%	0 (0.00%)	$0.00 (0.00%)
73.	ro-ro	**798** **(0.01%)**	43.98%	351 (0.02%)	50.88%	8.39	00:07:49	0.00%	0 (0.00%)	$0.00 (0.00%)
74.	hr-hr	**753** **(0.01%)**	78.35%	590 (0.03%)	41.43%	4.33	00:02:47	0.00%	0 (0.00%)	$0.00 (0.00%)
75.	ar-sa	**730** **(0.01%)**	79.18%	578 (0.03%)	64.25%	3.08	00:02:13	0.00%	0 (0.00%)	$0.00 (0.00%)
76.	sr	**687** **(0.01%)**	77.15%	530 (0.02%)	67.54%	2.49	00:01:30	0.00%	0 (0.00%)	$0.00 (0.00%)
77.	nb	**659** **(0.01%)**	55.24%	364 (0.02%)	54.93%	9.95	00:04:49	0.00%	0 (0.00%)	$0.00 (0.00%)
78.	es-co	**624** **(0.01%)**	22.76%	142 (0.01%)	57.85%	7.83	00:07:31	0.00%	0 (0.00%)	$0.00 (0.00%)
79.	ca-es	**597** **(0.01%)**	39.20%	234 (0.01%)	33.84%	8.04	00:11:34	0.00%	0 (0.00%)	$0.00 (0.00%)
80.	(not set)	**534** **(0.01%)**	72.85%	389 (0.02%)	40.82%	5.73	00:05:48	0.00%	0 (0.00%)	$0.00 (0.00%)
81.	es-pe	**516** **(0.01%)**	20.16%	104 (0.00%)	14.34%	14.25	00:13:51	0.00%	0 (0.00%)	$0.00 (0.00%)
82.	c	**494** **(0.01%)**	97.77%	483 (0.02%)	96.56%	1.10	00:00:05	0.00%	0 (0.00%)	$0.00 (0.00%)
83.	en-za	**449** **(0.01%)**	70.16%	315 (0.01%)	63.25%	3.34	00:02:58	0.00%	0 (0.00%)	$0.00 (0.00%)
84.	en-ca	**384** **(0.01%)**	65.10%	250 (0.01%)	50.00%	4.53	00:06:57	0.00%	0 (0.00%)	$0.00 (0.00%)
85.	es-pa	**361** **(0.00%)**	27.70%	100 (0.00%)	24.10%	8.70	00:08:32	0.00%	0 (0.00%)	$0.00 (0.00%)
86.	en_gb	**318** **(0.00%)**	84.59%	269 (0.01%)	71.70%	2.19	00:01:56	0.00%	0 (0.00%)	$0.00 (0.00%)
87.	en_us	**289** **(0.00%)**	85.81%	248 (0.01%)	66.78%	2.16	00:01:39	0.00%	0 (0.00%)	$0.00 (0.00%)
88.	en-in	**257** **(0.00%)**	90.66%	233 (0.01%)	70.04%	1.82	00:01:28	0.00%	0 (0.00%)	$0.00 (0.00%)
89.	ru; alexa toolbar	**236** **(0.00%)**	0.42%	1 (0.00%)	67.37%	6.61	00:14:21	0.00%	0 (0.00%)	$0.00 (0.00%)
90.	he-il	**220** **(0.00%)**	54.55%	120 (0.01%)	59.09%	4.65	00:03:14	0.00%	0 (0.00%)	$0.00 (0.00%)
91.	es-bo	**201** **(0.00%)**	13.93%	28 (0.00%)	13.43%	51.72	00:29:32	0.00%	0 (0.00%)	$0.00 (0.00%)
92.	id-id	**189** **(0.00%)**	68.78%	130 (0.01%)	59.26%	3.92	00:04:26	0.00%	0 (0.00%)	$0.00 (0.00%)
93.	de-ch	**187** **(0.00%)**	63.64%	119 (0.01%)	57.22%	7.89	00:02:47	0.00%	0 (0.00%)	$0.00 (0.00%)
94.	es-ve	**182** **(0.00%)**	46.15%	84 (0.00%)	30.22%	11.41	00:14:48	0.00%	0 (0.00%)	$0.00 (0.00%)
95.	fa	**177** **(0.00%)**	51.41%	91 (0.00%)	57.63%	3.53	00:05:25	0.00%	0 (0.00%)	$0.00 (0.00%)
96.	es-ni	**161** **(0.00%)**	9.32%	15 (0.00%)	21.12%	16.17	00:14:52	0.00%	0 (0.00%)	$0.00 (0.00%)
97.	fr-ch	**156** **(0.00%)**	39.74%	62 (0.00%)	35.26%	6.01	00:05:09	0.00%	0 (0.00%)	$0.00 (0.00%)
98.	es-uy	**141** **(0.00%)**	63.83%	90 (0.00%)	16.31%	14.91	00:10:00	0.00%	0 (0.00%)	$0.00 (0.00%)
99.	es-cr	**137** **(0.00%)**	42.34%	58 (0.00%)	25.55%	9.91	00:10:44	0.00%	0 (0.00%)	$0.00 (0.00%)
100.	fa-ir	**137** **(0.00%)**	55.47%	76 (0.00%)	54.74%	4.70	00:04:53	0.00%	0 (0.00%)	$0.00 (0.00%)

Rows 1 - 100 of 283

© 2014 Google
