# Supplementary material for: Trends in access of plant biodiversity data revealed by Google Analytics
Source: Biodivers Data J. 2014 Nov 11;(2):e1558. doi: 10.3897/BDJ.2.e1558 (PMC4238075; doi:10.3897/BDJ.2.e1558)
Supplement: Supplementary material 12 — Tropicos by year for language [file biodiversity_data_journal-2-e1558-s012.pdf]

Language

Jun 1, 2007 - Jun 1, 2008

All Sessions
100.00%

+ Add Segment

Explorer

Summary

Language	Acquisition			Behavior			Conversions		
	Sessions	% New Sessions	New Users	Bounce Rate	Pages / Session	Avg. Session Duration	Goal Conversion Rate	Goal Completions	Goal Value
	97,172 % of Total: 100.00% (97,172)	26.71% Site Avg: 26.65% (0.24%)	25,956 % of Total: 100.24% (25,893)	20.11% Site Avg: 20.11% (0.00%)	13.93 Site Avg: 13.93 (0.00%)	00:14:37 Site Avg: 00:14:37 (0.00%)	0.00% Site Avg: 0.00% (0.00%)	0 % of Total: 0.00% (0)	$0.00 % of Total: 0.00% ($0.00)
1. en-us	33,434 (34.41%)	26.81%	8,962 (34.53%)	19.67%	12.16	00:12:46	0.00%	0 (0.00%)	$0.00 (0.00%)
2. es	22,796 (23.46%)	23.58%	5,375 (20.71%)	21.88%	16.38	00:16:41	0.00%	0 (0.00%)	$0.00 (0.00%)
3. pt-br	16,586 (17.07%)	30.07%	4,987 (19.21%)	18.14%	13.99	00:14:35	0.00%	0 (0.00%)	$0.00 (0.00%)
4. fr	5,962 (6.14%)	22.26%	1,327 (5.11%)	24.05%	12.82	00:14:41	0.00%	0 (0.00%)	$0.00 (0.00%)
5. es-es	3,993 (4.11%)	28.55%	1,140 (4.39%)	18.08%	16.45	00:18:44	0.00%	0 (0.00%)	$0.00 (0.00%)
6. de	2,840 (2.92%)	29.96%	851 (3.28%)	22.43%	13.57	00:12:28	0.00%	0 (0.00%)	$0.00 (0.00%)
7. en	1,889 (1.94%)	27.10%	512 (1.97%)	18.58%	10.47	00:13:29	0.00%	0 (0.00%)	$0.00 (0.00%)
8. zh-cn	1,312 (1.35%)	20.27%	266 (1.02%)	13.95%	16.96	00:16:13	0.00%	0 (0.00%)	$0.00 (0.00%)
9. en-gb	1,139 (1.17%)	22.48%	256 (0.99%)	18.70%	11.34	00:14:59	0.00%	0 (0.00%)	$0.00 (0.00%)
10. it	1,012 (1.04%)	21.74%	220 (0.85%)	15.81%	20.67	00:22:53	0.00%	0 (0.00%)	$0.00 (0.00%)
11. zh-tw	821 (0.84%)	28.87%	237 (0.91%)	24.97%	14.10	00:13:24	0.00%	0 (0.00%)	$0.00 (0.00%)
12. es-ar	754 (0.78%)	25.20%	190 (0.73%)	19.23%	20.48	00:17:44	0.00%	0 (0.00%)	$0.00 (0.00%)
13. ja	605 (0.62%)	40.50%	245 (0.94%)	20.17%	9.40	00:06:23	0.00%	0 (0.00%)	$0.00 (0.00%)
14. pl	440 (0.45%)	29.32%	129 (0.50%)	26.59%	11.82	00:11:47	0.00%	0 (0.00%)	$0.00 (0.00%)
15. nl	414 (0.43%)	43.96%	182 (0.70%)	20.05%	8.85	00:08:18	0.00%	0 (0.00%)	$0.00 (0.00%)
16. sv	369 (0.38%)	17.07%	63 (0.24%)	11.65%	9.45	00:16:21	0.00%	0 (0.00%)	$0.00 (0.00%)
17. ru	348 (0.36%)	43.97%	153 (0.59%)	24.43%	9.49	00:08:53	0.00%	0 (0.00%)	$0.00 (0.00%)
18. ko	320 (0.33%)	26.25%	84 (0.32%)	21.25%	16.55	00:13:42	0.00%	0 (0.00%)	$0.00 (0.00%)
19. cs	306 (0.31%)	40.52%	124 (0.48%)	27.45%	19.24	00:13:21	0.00%	0 (0.00%)	$0.00 (0.00%)
20. sv-se	293 (0.30%)	15.36%	45 (0.17%)	8.19%	17.29	00:32:29	0.00%	0 (0.00%)	$0.00 (0.00%)
21. de-de	193 (0.20%)	32.64%	63 (0.24%)	12.44%	16.12	00:16:12	0.00%	0 (0.00%)	$0.00 (0.00%)
22. fr-fr	188 (0.19%)	18.09%	34 (0.13%)	30.85%	5.21	00:14:12	0.00%	0 (0.00%)	$0.00 (0.00%)
23. pt	145 (0.15%)	57.24%	83 (0.32%)	28.28%	7.88	00:09:35	0.00%	0 (0.00%)	$0.00 (0.00%)
24. ja-jp	125 (0.13%)	26.40%	33 (0.13%)	7.20%	10.98	00:12:50	0.00%	0 (0.00%)	$0.00 (0.00%)
25. fi	124 (0.13%)	20.16%	25 (0.10%)	11.29%	9.47	00:12:00	0.00%	0 (0.00%)	$0.00 (0.00%)

© 2014 Google
