# Supplementary material for: Trends in access of plant biodiversity data revealed by Google Analytics
Source: Biodivers Data J. 2014 Nov 11;(2):e1558. doi: 10.3897/BDJ.2.e1558 (PMC4238075; doi:10.3897/BDJ.2.e1558)
Supplement: Supplementary material 13 — Tropicos by year for language 2 [file biodiversity_data_journal-2-e1558-s013.pdf]

Language

Jun 1, 2008 - Jun 1, 2009

All Sessions
100.00%

+ Add Segment

Explorer

Summary

Language	Acquisition			Behavior			Conversions		
	Sessions	% New Sessions	New Users	Bounce Rate	Pages / Session	Avg. Session Duration	Goal Conversion Rate	Goal Completions	Goal Value
	718,961 % of Total: 100.00% (718,961)	27.92% Site Avg: 27.88% (0.15%)	200,740 % of Total: 100.15% (200,440)	27.09% Site Avg: 27.09% (0.00%)	12.81 Site Avg: 12.81 (0.00%)	00:13:05 Site Avg: 00:13:05 (0.00%)	0.00% Site Avg: 0.00% (0.00%)	0 % of Total: 0.00% (0)	$0.00 % of Total: 0.00% ($0.00)
1. en-us	269,476 (37.48%)	29.89%	80,541 (40.12%)	28.31%	11.92	00:12:11	0.00%	0 (0.00%)	$0.00 (0.00%)
2. es	136,818 (19.03%)	21.46%	29,366 (14.63%)	26.48%	15.07	00:14:39	0.00%	0 (0.00%)	$0.00 (0.00%)
3. pt-br	103,352 (14.38%)	27.37%	28,290 (14.09%)	20.75%	13.76	00:13:56	0.00%	0 (0.00%)	$0.00 (0.00%)
4. fr	44,365 (6.17%)	29.40%	13,045 (6.50%)	31.82%	11.29	00:13:35	0.00%	0 (0.00%)	$0.00 (0.00%)
5. es-es	35,030 (4.87%)	24.33%	8,524 (4.25%)	22.40%	17.93	00:17:02	0.00%	0 (0.00%)	$0.00 (0.00%)
6. de	27,414 (3.81%)	31.63%	8,670 (4.32%)	28.77%	9.94	00:10:36	0.00%	0 (0.00%)	$0.00 (0.00%)
7. en	11,738 (1.63%)	25.52%	2,996 (1.49%)	27.74%	11.56	00:14:59	0.00%	0 (0.00%)	$0.00 (0.00%)
8. en-gb	10,509 (1.46%)	25.18%	2,646 (1.32%)	28.55%	10.03	00:12:57	0.00%	0 (0.00%)	$0.00 (0.00%)
9. zh-cn	9,968 (1.39%)	23.91%	2,383 (1.19%)	21.71%	11.24	00:13:44	0.00%	0 (0.00%)	$0.00 (0.00%)
10. zh-tw	8,071 (1.12%)	17.17%	1,386 (0.69%)	27.25%	7.77	00:13:10	0.00%	0 (0.00%)	$0.00 (0.00%)
11. it	6,889 (0.96%)	38.76%	2,670 (1.33%)	30.80%	13.46	00:11:12	0.00%	0 (0.00%)	$0.00 (0.00%)
12. es-ar	6,213 (0.86%)	21.76%	1,352 (0.67%)	18.62%	16.90	00:17:13	0.00%	0 (0.00%)	$0.00 (0.00%)
13. nl	5,396 (0.75%)	41.49%	2,239 (1.12%)	39.20%	9.37	00:06:54	0.00%	0 (0.00%)	$0.00 (0.00%)
14. ru	5,337 (0.74%)	47.70%	2,546 (1.27%)	49.92%	6.57	00:06:01	0.00%	0 (0.00%)	$0.00 (0.00%)
15. ko	4,901 (0.68%)	21.69%	1,063 (0.53%)	20.38%	13.92	00:12:54	0.00%	0 (0.00%)	$0.00 (0.00%)
16. ja	4,674 (0.65%)	42.34%	1,979 (0.99%)	29.70%	8.23	00:05:51	0.00%	0 (0.00%)	$0.00 (0.00%)
17. pl	4,607 (0.64%)	38.68%	1,782 (0.89%)	37.27%	11.18	00:09:46	0.00%	0 (0.00%)	$0.00 (0.00%)
18. sv	2,795 (0.39%)	28.19%	788 (0.39%)	27.58%	10.11	00:12:03	0.00%	0 (0.00%)	$0.00 (0.00%)
19. cs	2,655 (0.37%)	48.96%	1,300 (0.65%)	40.45%	11.11	00:08:18	0.00%	0 (0.00%)	$0.00 (0.00%)
20. sv-se	1,992 (0.28%)	22.24%	443 (0.22%)	16.37%	12.06	00:12:34	0.00%	0 (0.00%)	$0.00 (0.00%)
21. fr-fr	1,577 (0.22%)	17.95%	283 (0.14%)	26.82%	14.60	00:12:21	0.00%	0 (0.00%)	$0.00 (0.00%)
22. pt	1,566 (0.22%)	45.59%	714 (0.36%)	32.50%	7.02	00:06:41	0.00%	0 (0.00%)	$0.00 (0.00%)
23. da	1,262 (0.18%)	44.77%	565 (0.28%)	39.06%	5.92	00:06:21	0.00%	0 (0.00%)	$0.00 (0.00%)
24. de-de	1,240 (0.17%)	32.82%	407 (0.20%)	20.24%	12.09	00:10:54	0.00%	0 (0.00%)	$0.00 (0.00%)
25. tr	1,207 (0.17%)	63.21%	763 (0.38%)	44.74%	5.53	00:04:37	0.00%	0 (0.00%)	$0.00 (0.00%)
26. ca	971 (0.14%)	7.93%	77 (0.04%)	22.66%	12.09	00:22:22	0.00%	0 (0.00%)	$0.00 (0.00%)
27. pt-pt	773 (0.11%)	37.00%	286 (0.14%)	23.80%	12.72	00:11:31	0.00%	0 (0.00%)	$0.00 (0.00%)
28. hu	746 (0.10%)	74.26%	554 (0.28%)	61.66%	3.24	00:01:39	0.00%	0 (0.00%)	$0.00 (0.00%)
29. ja-jp	574 (0.08%)	32.75%	188 (0.09%)	31.01%	13.11	00:07:26	0.00%	0 (0.00%)	$0.00 (0.00%)
30. ja-jp-mac	551 (0.08%)	11.07%	61 (0.03%)	10.34%	25.66	00:17:25	0.00%	0 (0.00%)	$0.00 (0.00%)
31. fi	536 (0.07%)	62.69%	336 (0.17%)	41.79%	5.83	00:03:43	0.00%	0 (0.00%)	$0.00 (0.00%)
32. es-419	466 (0.06%)	19.74%	92 (0.05%)	18.03%	13.18	00:15:22	0.00%	0 (0.00%)	$0.00 (0.00%)

33.	es-la	**452** **(0.06%)**	22.12%	100 (0.05%)	37.39%	23.75	00:14:18	0.00%	0 (0.00%)	$0.00 (0.00%)
34.	sk	**409** **(0.06%)**	64.79%	265 (0.13%)	61.37%	3.58	00:01:39	0.00%	0 (0.00%)	$0.00 (0.00%)
35.	de-at	**397** **(0.06%)**	35.01%	139 (0.07%)	12.09%	11.40	00:10:57	0.00%	0 (0.00%)	$0.00 (0.00%)
36.	no	**395** **(0.05%)**	51.90%	205 (0.10%)	37.22%	6.87	00:05:22	0.00%	0 (0.00%)	$0.00 (0.00%)
37.	el	**385** **(0.05%)**	75.84%	292 (0.15%)	62.08%	2.93	00:01:43	0.00%	0 (0.00%)	$0.00 (0.00%)
38.	fr-ca	**368** **(0.05%)**	1.09%	4 (0.00%)	21.74%	35.19	00:44:26	0.00%	0 (0.00%)	$0.00 (0.00%)
39.	it-it	**296** **(0.04%)**	39.19%	116 (0.06%)	31.08%	7.92	00:08:55	0.00%	0 (0.00%)	$0.00 (0.00%)
40.	nb-no	**288** **(0.04%)**	20.14%	58 (0.03%)	74.65%	3.14	00:03:20	0.00%	0 (0.00%)	$0.00 (0.00%)
41.	ru; alexa toolbar	**228** **(0.03%)**	0.44%	1 (0.00%)	68.42%	6.63	00:14:26	0.00%	0 (0.00%)	$0.00 (0.00%)
42.	sl	**182** **(0.03%)**	61.54%	112 (0.06%)	67.58%	2.26	00:01:04	0.00%	0 (0.00%)	$0.00 (0.00%)
43.	nl-nl	**165** **(0.02%)**	44.24%	73 (0.04%)	31.52%	6.61	00:10:47	0.00%	0 (0.00%)	$0.00 (0.00%)
44.	bg	**159** **(0.02%)**	66.67%	106 (0.05%)	54.72%	8.99	00:04:31	0.00%	0 (0.00%)	$0.00 (0.00%)
45.	he	**121** **(0.02%)**	68.60%	83 (0.04%)	74.38%	1.87	00:00:18	0.00%	0 (0.00%)	$0.00 (0.00%)
46.	ar-sa	**120** **(0.02%)**	81.67%	98 (0.05%)	69.17%	2.41	00:01:32	0.00%	0 (0.00%)	$0.00 (0.00%)
47.	et	**116** **(0.02%)**	24.14%	28 (0.01%)	31.90%	14.62	00:17:29	0.00%	0 (0.00%)	$0.00 (0.00%)
48.	th	**108** **(0.02%)**	35.19%	38 (0.02%)	16.67%	11.78	00:13:51	0.00%	0 (0.00%)	$0.00 (0.00%)
49.	pl-pl	**99** **(0.01%)**	35.35%	35 (0.02%)	28.28%	5.53	00:05:47	0.00%	0 (0.00%)	$0.00 (0.00%)
50.	lt	**97** **(0.01%)**	71.13%	69 (0.03%)	73.20%	1.97	00:00:59	0.00%	0 (0.00%)	$0.00 (0.00%)
51.	(not set)	**85** **(0.01%)**	65.88%	56 (0.03%)	24.71%	5.72	00:08:32	0.00%	0 (0.00%)	$0.00 (0.00%)
52.	ro	**85** **(0.01%)**	84.71%	72 (0.04%)	64.71%	6.38	00:02:43	0.00%	0 (0.00%)	$0.00 (0.00%)
53.	id	**73** **(0.01%)**	68.49%	50 (0.02%)	39.73%	3.66	00:03:46	0.00%	0 (0.00%)	$0.00 (0.00%)
54.	cs-cz	**60** **(0.01%)**	71.67%	43 (0.02%)	41.67%	5.35	00:02:14	0.00%	0 (0.00%)	$0.00 (0.00%)
55.	pt-br; alexa	**57** **(0.01%)**	43.86%	25 (0.01%)	15.79%	17.02	00:14:20	0.00%	0 (0.00%)	$0.00 (0.00%)
56.	hr	**48** **(0.01%)**	91.67%	44 (0.02%)	68.75%	4.52	00:01:39	0.00%	0 (0.00%)	$0.00 (0.00%)
57.	gl	**46** **(0.01%)**	6.52%	3 (0.00%)	15.22%	9.35	00:21:17	0.00%	0 (0.00%)	$0.00 (0.00%)
58.	zh-hk	**44** **(0.01%)**	63.64%	28 (0.01%)	31.82%	29.82	00:08:22	0.00%	0 (0.00%)	$0.00 (0.00%)
59.	ru-ru	**38** **(0.01%)**	68.42%	26 (0.01%)	73.68%	1.79	00:00:24	0.00%	0 (0.00%)	$0.00 (0.00%)
60.	es-xl	**36** **(0.01%)**	5.56%	2 (0.00%)	8.33%	22.78	00:33:26	0.00%	0 (0.00%)	$0.00 (0.00%)
61.	hu-hu	**24** **(0.00%)**	54.17%	13 (0.01%)	66.67%	2.21	00:01:30	0.00%	0 (0.00%)	$0.00 (0.00%)
62.	uk	**24** **(0.00%)**	75.00%	18 (0.01%)	54.17%	4.12	00:04:12	0.00%	0 (0.00%)	$0.00 (0.00%)
63.	lv	**23** **(0.00%)**	69.57%	16 (0.01%)	91.30%	1.13	00:00:07	0.00%	0 (0.00%)	$0.00 (0.00%)
64.	da-dk	**20** **(0.00%)**	75.00%	15 (0.01%)	55.00%	3.45	00:00:38	0.00%	0 (0.00%)	$0.00 (0.00%)
65.	pt_br	**19** **(0.00%)**	10.53%	2 (0.00%)	5.26%	4.79	00:01:08	0.00%	0 (0.00%)	$0.00 (0.00%)
66.	he-il	**18** **(0.00%)**	5.56%	1 (0.00%)	11.11%	8.56	00:07:20	0.00%	0 (0.00%)	$0.00 (0.00%)
67.	fi-fi	**15** **(0.00%)**	73.33%	11 (0.01%)	40.00%	9.60	00:06:06	0.00%	0 (0.00%)	$0.00 (0.00%)
68.	en_us	**14** **(0.00%)**	85.71%	12 (0.01%)	57.14%	5.00	00:05:56	0.00%	0 (0.00%)	$0.00 (0.00%)
69.	nb	**14** **(0.00%)**	92.86%	13 (0.01%)	64.29%	4.64	00:02:33	0.00%	0 (0.00%)	$0.00 (0.00%)
70.	ar	**13** **(0.00%)**	100.00%	13 (0.01%)	84.62%	1.85	00:00:17	0.00%	0 (0.00%)	$0.00 (0.00%)
71.	es-mx	**12** **(0.00%)**	33.33%	4 (0.00%)	16.67%	5.08	00:02:40	0.00%	0 (0.00%)	$0.00 (0.00%)
72.	sr	**10** **(0.00%)**	100.00%	10 (0.00%)	50.00%	3.00	00:00:18	0.00%	0 (0.00%)	$0.00 (0.00%)
73.	el-gr	**9** **(0.00%)**	77.78%	7 (0.00%)	55.56%	2.44	00:02:59	0.00%	0 (0.00%)	$0.00 (0.00%)
74.	mn	**9** **(0.00%)**	100.00%	9 (0.00%)	44.44%	1.78	00:00:27	0.00%	0 (0.00%)	$0.00 (0.00%)
75.	th-th	**7** **(0.00%)**	71.43%	5 (0.00%)	85.71%	1.14	00:00:01	0.00%	0 (0.00%)	$0.00 (0.00%)
76.	vi	**7** **(0.00%)**	42.86%	3 (0.00%)	14.29%	29.14	00:29:33	0.00%	0 (0.00%)	$0.00 (0.00%)
77.	ca-ad	**6** **(0.00%)**	50.00%	3 (0.00%)	16.67%	4.67	00:00:50	0.00%	0 (0.00%)	$0.00 (0.00%)
[illegible]	[illegible]	[illegible]	[illegible]	[illegible]	[illegible]	[illegible]	[illegible]	[illegible]	[illegible]	[illegible]

78.	chrome://navigator/locale/navigator.properties	**6**	**(0.00%)**	100.00%	6	(0.00%)	83.33%	1.50	00:01:07	0.00%	0	(0.00%)	$0.00	(0.00%)
79.	mk	**6**	**(0.00%)**	83.33%	5	(0.00%)	50.00%	8.50	00:02:15	0.00%	0	(0.00%)	$0.00	(0.00%)
80.	sl-si	**6**	**(0.00%)**	83.33%	5	(0.00%)	100.00%	1.00	00:00:00	0.00%	0	(0.00%)	$0.00	(0.00%)
81.	en-ca	**5**	**(0.00%)**	80.00%	4	(0.00%)	40.00%	2.00	00:00:08	0.00%	0	(0.00%)	$0.00	(0.00%)
82.	eu	**5**	**(0.00%)**	80.00%	4	(0.00%)	80.00%	2.00	00:00:25	0.00%	0	(0.00%)	$0.00	(0.00%)
83.	de-ch	**4**	**(0.00%)**	100.00%	4	(0.00%)	75.00%	3.50	00:10:45	0.00%	0	(0.00%)	$0.00	(0.00%)
84.	en-au	**4**	**(0.00%)**	50.00%	2	(0.00%)	50.00%	3.25	00:04:28	0.00%	0	(0.00%)	$0.00	(0.00%)
85.	ga-ie	**4**	**(0.00%)**	100.00%	4	(0.00%)	75.00%	1.25	00:00:06	0.00%	0	(0.00%)	$0.00	(0.00%)
86.	ko-kr	**4**	**(0.00%)**	75.00%	3	(0.00%)	50.00%	1.75	00:00:08	0.00%	0	(0.00%)	$0.00	(0.00%)
87.	lt-lt	**3**	**(0.00%)**	33.33%	1	(0.00%)	100.00%	1.00	00:00:00	0.00%	0	(0.00%)	$0.00	(0.00%)
88.	tr-tr	**3**	**(0.00%)**	33.33%	1	(0.00%)	33.33%	32.00	00:11:31	0.00%	0	(0.00%)	$0.00	(0.00%)
89.	bg-bg	**2**	**(0.00%)**	100.00%	2	(0.00%)	50.00%	2.00	00:00:12	0.00%	0	(0.00%)	$0.00	(0.00%)
90.	c	**2**	**(0.00%)**	100.00%	2	(0.00%)	50.00%	1.50	00:00:38	0.00%	0	(0.00%)	$0.00	(0.00%)
91.	ca-es	**2**	**(0.00%)**	100.00%	2	(0.00%)	100.00%	1.00	00:00:00	0.00%	0	(0.00%)	$0.00	(0.00%)
92.	en-us; megaupload 1.0	**2**	**(0.00%)**	100.00%	2	(0.00%)	0.00%	14.00	00:04:42	0.00%	0	(0.00%)	$0.00	(0.00%)
93.	et-ee	**2**	**(0.00%)**	100.00%	2	(0.00%)	50.00%	2.00	00:00:12	0.00%	0	(0.00%)	$0.00	(0.00%)
94.	gl-es	**2**	**(0.00%)**	50.00%	1	(0.00%)	50.00%	3.00	00:00:20	0.00%	0	(0.00%)	$0.00	(0.00%)
95.	is	**2**	**(0.00%)**	100.00%	2	(0.00%)	0.00%	2.50	00:00:29	0.00%	0	(0.00%)	$0.00	(0.00%)
96.	pt-br;alexa	**2**	**(0.00%)**	100.00%	2	(0.00%)	0.00%	4.50	00:12:42	0.00%	0	(0.00%)	$0.00	(0.00%)
97.	af-za	**1**	**(0.00%)**	100.00%	1	(0.00%)	0.00%	6.00	00:02:13	0.00%	0	(0.00%)	$0.00	(0.00%)
98.	cy-gb	**1**	**(0.00%)**	100.00%	1	(0.00%)	100.00%	1.00	00:00:00	0.00%	0	(0.00%)	$0.00	(0.00%)
99.	en-br; alexa	**1**	**(0.00%)**	100.00%	1	(0.00%)	0.00%	90.00	00:48:17	0.00%	0	(0.00%)	$0.00	(0.00%)
100.	en-br;alexa	**1**	**(0.00%)**	100.00%	1	(0.00%)	100.00%	1.00	00:00:00	0.00%	0	(0.00%)	$0.00	(0.00%)

Rows 1 - 100 of 121

© 2014 Google
