# Supplementary material for: Trends in access of plant biodiversity data revealed by Google Analytics
Source: Biodivers Data J. 2014 Nov 11;(2):e1558. doi: 10.3897/BDJ.2.e1558 (PMC4238075; doi:10.3897/BDJ.2.e1558)
Supplement: Supplementary material 15 — Tropicos by year for language 3 [file biodiversity_data_journal-2-e1558-s015.pdf]

Google Analytics

http://www.tropicos.org - http://www.tro... www.tropicos.org

Go to this report

Language

Jun 1, 2009 - Jun 1, 2010

All Sessions
100.00%

+ Add Segment

Explorer

Summary

Language	Acquisition			Behavior			Conversions		
	Sessions	% New Sessions	New Users	Bounce Rate	Pages / Session	Avg. Session Duration	Goal Conversion Rate	Goal Completions	Goal Value
	907,669 % of Total: 100.00% (907,669)	25.72% Site Avg: 25.66% (0.20%)	233,415 % of Total: 100.20% (232,953)	26.14% Site Avg: 26.14% (0.00%)	14.47 Site Avg: 14.47 (0.00%)	00:14:50 Site Avg: 00:14:50 (0.00%)	0.00% Site Avg: 0.00% (0.00%)	0 % of Total: 0.00% (0)	$0.00 % of Total: 0.00% ($0.00)
1. en-us	**342,807** **(37.77%)**	29.93%	102,617 (43.96%)	31.54%	14.11	00:13:31	0.00%	0 (0.00%)	$0.00 (0.00%)
2. es	**157,127** **(17.31%)**	22.01%	34,582 (14.82%)	19.42%	16.77	00:16:45	0.00%	0 (0.00%)	$0.00 (0.00%)
3. pt-br	**132,187** **(14.56%)**	25.09%	33,170 (14.21%)	20.45%	12.96	00:14:37	0.00%	0 (0.00%)	$0.00 (0.00%)
4. es-es	**63,197** **(6.96%)**	20.27%	12,812 (5.49%)	26.33%	16.39	00:15:39	0.00%	0 (0.00%)	$0.00 (0.00%)
5. fr	**51,488** **(5.67%)**	18.05%	9,294 (3.98%)	19.37%	16.20	00:21:08	0.00%	0 (0.00%)	$0.00 (0.00%)
6. de	**31,762** **(3.50%)**	26.60%	8,450 (3.62%)	25.69%	12.50	00:14:58	0.00%	0 (0.00%)	$0.00 (0.00%)
7. en-gb	**15,240** **(1.68%)**	21.64%	3,298 (1.41%)	26.42%	12.59	00:13:07	0.00%	0 (0.00%)	$0.00 (0.00%)
8. zh-cn	**13,297** **(1.46%)**	17.59%	2,339 (1.00%)	17.12%	14.60	00:16:52	0.00%	0 (0.00%)	$0.00 (0.00%)
9. en	**10,709** **(1.18%)**	17.90%	1,917 (0.82%)	38.37%	10.48	00:12:05	0.00%	0 (0.00%)	$0.00 (0.00%)
10. es-ar	**9,361** **(1.03%)**	21.46%	2,009 (0.86%)	24.23%	15.01	00:16:26	0.00%	0 (0.00%)	$0.00 (0.00%)
11. ru	**9,020** **(0.99%)**	55.08%	4,968 (2.13%)	49.14%	6.32	00:05:56	0.00%	0 (0.00%)	$0.00 (0.00%)
12. zh-tw	**8,828** **(0.97%)**	15.73%	1,389 (0.60%)	32.68%	8.18	00:11:25	0.00%	0 (0.00%)	$0.00 (0.00%)
13. it	**8,082** **(0.89%)**	27.73%	2,241 (0.96%)	21.89%	21.92	00:17:13	0.00%	0 (0.00%)	$0.00 (0.00%)
14. nl	**5,349** **(0.59%)**	28.29%	1,513 (0.65%)	30.70%	13.31	00:10:06	0.00%	0 (0.00%)	$0.00 (0.00%)
15. es-419	**5,148** **(0.57%)**	17.00%	875 (0.37%)	18.92%	14.85	00:18:05	0.00%	0 (0.00%)	$0.00 (0.00%)
16. ko	**5,075** **(0.56%)**	22.92%	1,163 (0.50%)	21.34%	10.13	00:11:54	0.00%	0 (0.00%)	$0.00 (0.00%)
17. pl	**4,955** **(0.55%)**	26.84%	1,330 (0.57%)	34.15%	10.98	00:10:46	0.00%	0 (0.00%)	$0.00 (0.00%)
18. ja	**4,706** **(0.52%)**	34.32%	1,615 (0.69%)	22.55%	10.28	00:09:41	0.00%	0 (0.00%)	$0.00 (0.00%)
19. fr-fr	**3,693** **(0.41%)**	8.10%	299 (0.13%)	30.73%	17.41	00:14:19	0.00%	0 (0.00%)	$0.00 (0.00%)
20. sv-se	**2,694** **(0.30%)**	10.54%	284 (0.12%)	15.59%	9.72	00:13:25	0.00%	0 (0.00%)	$0.00 (0.00%)
21. sv	**2,681** **(0.30%)**	20.59%	552 (0.24%)	24.84%	10.31	00:12:26	0.00%	0 (0.00%)	$0.00 (0.00%)
22. cs	**2,396** **(0.26%)**	40.40%	968 (0.41%)	31.89%	12.19	00:11:51	0.00%	0 (0.00%)	$0.00 (0.00%)
23. de-de	**1,935** **(0.21%)**	21.65%	419 (0.18%)	24.81%	16.43	00:13:14	0.00%	0 (0.00%)	$0.00 (0.00%)
24. es-mx	**1,716** **(0.19%)**	16.72%	287 (0.12%)	20.80%	17.02	00:17:21	0.00%	0 (0.00%)	$0.00 (0.00%)
25. pt	**1,462** **(0.16%)**	41.86%	612 (0.26%)	31.40%	12.88	00:11:54	0.00%	0 (0.00%)	$0.00 (0.00%)
26. pt-pt	**1,254** **(0.14%)**	37.08%	465 (0.20%)	33.01%	8.93	00:09:27	0.00%	0 (0.00%)	$0.00 (0.00%)

27.	tr	**1,134**	**(0.12%)**	46.65%	529	(0.23%)	36.07%	9.60	00:07:14	0.00%	0	(0.00%)	$0.00	(0.00%)
28.	ca	**1,072**	**(0.12%)**	10.17%	109	(0.05%)	30.32%	12.58	00:14:01	0.00%	0	(0.00%)	$0.00	(0.00%)
29.	ja-jp	**908**	**(0.10%)**	13.11%	119	(0.05%)	37.33%	12.35	00:13:41	0.00%	0	(0.00%)	$0.00	(0.00%)
30.	da	**891**	**(0.10%)**	36.70%	327	(0.14%)	28.73%	8.70	00:06:24	0.00%	0	(0.00%)	$0.00	(0.00%)
31.	fr-ca	**736**	**(0.08%)**	0.82%	6	(0.00%)	5.71%	98.96	01:14:50	0.00%	0	(0.00%)	$0.00	(0.00%)
32.	ja-jp-mac	**641**	**(0.07%)**	16.07%	103	(0.04%)	12.17%	49.65	00:26:23	0.00%	0	(0.00%)	$0.00	(0.00%)
33.	es-xl	**621**	**(0.07%)**	0.97%	6	(0.00%)	24.96%	15.09	00:21:56	0.00%	0	(0.00%)	$0.00	(0.00%)
34.	el	**562**	**(0.06%)**	46.26%	260	(0.11%)	38.43%	5.85	00:03:10	0.00%	0	(0.00%)	$0.00	(0.00%)
35.	hu	**495**	**(0.05%)**	77.37%	383	(0.16%)	63.23%	3.20	00:01:25	0.00%	0	(0.00%)	$0.00	(0.00%)
36.	es-la	**381**	**(0.04%)**	21.52%	82	(0.04%)	37.53%	9.37	00:22:50	0.00%	0	(0.00%)	$0.00	(0.00%)
37.	fi	**369**	**(0.04%)**	48.78%	180	(0.08%)	30.35%	10.55	00:06:37	0.00%	0	(0.00%)	$0.00	(0.00%)
38.	no	**316**	**(0.03%)**	37.66%	119	(0.05%)	35.13%	5.86	00:05:46	0.00%	0	(0.00%)	$0.00	(0.00%)
39.	sk	**314**	**(0.03%)**	57.96%	182	(0.08%)	62.10%	4.75	00:03:11	0.00%	0	(0.00%)	$0.00	(0.00%)
40.	it-it	**297**	**(0.03%)**	25.93%	77	(0.03%)	27.27%	11.89	00:13:58	0.00%	0	(0.00%)	$0.00	(0.00%)
41.	de-at	**291**	**(0.03%)**	63.23%	184	(0.08%)	6.53%	8.41	00:11:49	0.00%	0	(0.00%)	$0.00	(0.00%)
42.	th	**244**	**(0.03%)**	27.46%	67	(0.03%)	24.59%	9.76	00:09:29	0.00%	0	(0.00%)	$0.00	(0.00%)
43.	id	**214**	**(0.02%)**	48.60%	104	(0.04%)	33.64%	8.06	00:14:49	0.00%	0	(0.00%)	$0.00	(0.00%)
44.	nb-no	**203**	**(0.02%)**	24.63%	50	(0.02%)	72.41%	2.45	00:02:04	0.00%	0	(0.00%)	$0.00	(0.00%)
45.	nl-nl	**150**	**(0.02%)**	32.00%	48	(0.02%)	26.00%	7.88	00:06:17	0.00%	0	(0.00%)	$0.00	(0.00%)
46.	et	**133**	**(0.01%)**	62.41%	83	(0.04%)	51.13%	2.86	00:01:30	0.00%	0	(0.00%)	$0.00	(0.00%)
47.	bg	**122**	**(0.01%)**	71.31%	87	(0.04%)	54.92%	3.37	00:01:45	0.00%	0	(0.00%)	$0.00	(0.00%)
48.	es-cl	**120**	**(0.01%)**	32.50%	39	(0.02%)	25.00%	14.90	00:16:24	0.00%	0	(0.00%)	$0.00	(0.00%)
49.	lt	**104**	**(0.01%)**	70.19%	73	(0.03%)	63.46%	3.76	00:02:25	0.00%	0	(0.00%)	$0.00	(0.00%)
50.	sl	**104**	**(0.01%)**	64.42%	67	(0.03%)	75.96%	3.16	00:01:13	0.00%	0	(0.00%)	$0.00	(0.00%)
51.	ro	**93**	**(0.01%)**	70.97%	66	(0.03%)	51.61%	6.83	00:05:33	0.00%	0	(0.00%)	$0.00	(0.00%)
52.	he	**88**	**(0.01%)**	78.41%	69	(0.03%)	63.64%	2.15	00:01:05	0.00%	0	(0.00%)	$0.00	(0.00%)
53.	zh-hk	**73**	**(0.01%)**	56.16%	41	(0.02%)	46.58%	17.44	00:06:10	0.00%	0	(0.00%)	$0.00	(0.00%)
54.	ar-sa	**61**	**(0.01%)**	83.61%	51	(0.02%)	54.10%	3.97	00:02:41	0.00%	0	(0.00%)	$0.00	(0.00%)
55.	ru-ru	**57**	**(0.01%)**	78.95%	45	(0.02%)	63.16%	2.23	00:03:13	0.00%	0	(0.00%)	$0.00	(0.00%)
56.	pt_br	**56**	**(0.01%)**	10.71%	6	(0.00%)	12.50%	9.20	00:12:01	0.00%	0	(0.00%)	$0.00	(0.00%)
57.	uk	**49**	**(0.01%)**	73.47%	36	(0.02%)	46.94%	3.27	00:02:43	0.00%	0	(0.00%)	$0.00	(0.00%)
58.	pl-pl	**44**	**(0.00%)**	54.55%	24	(0.01%)	59.09%	4.91	00:01:38	0.00%	0	(0.00%)	$0.00	(0.00%)
59.	vi	**44**	**(0.00%)**	50.00%	22	(0.01%)	40.91%	15.48	00:09:16	0.00%	0	(0.00%)	$0.00	(0.00%)
60.	cs-cz	**41**	**(0.00%)**	43.90%	18	(0.01%)	26.83%	22.71	00:14:15	0.00%	0	(0.00%)	$0.00	(0.00%)
61.	lv	**40**	**(0.00%)**	52.50%	21	(0.01%)	37.50%	3.82	00:02:48	0.00%	0	(0.00%)	$0.00	(0.00%)
62.	gl	**39**	**(0.00%)**	20.51%	8	(0.00%)	25.64%	13.44	00:29:19	0.00%	0	(0.00%)	$0.00	(0.00%)
63.	th-th	**34**	**(0.00%)**	20.59%	7	(0.00%)	44.12%	5.71	00:10:45	0.00%	0	(0.00%)	$0.00	(0.00%)
64.	hr	**31**	**(0.00%)**	93.55%	29	(0.01%)	64.52%	2.42	00:01:20	0.00%	0	(0.00%)	$0.00	(0.00%)
65.	pt-br; alexa	**30**	**(0.00%)**	60.00%	18	(0.01%)	20.00%	10.50	00:13:39	0.00%	0	(0.00%)	$0.00	(0.00%)
66.	(not set)	**29**	**(0.00%)**	82.76%	24	(0.01%)	24.14%	9.52	00:10:57	0.00%	0	(0.00%)	$0.00	(0.00%)

67.	hu-hu	**27**	**(0.00%)**	40.74%	11	(0.00%)	29.63%	5.70	00:04:06	0.00%	0	(0.00%)	$0.00	(0.00%)
68.	ko-kr	**25**	**(0.00%)**	32.00%	8	(0.00%)	44.00%	9.32	00:07:59	0.00%	0	(0.00%)	$0.00	(0.00%)
69.	fi-fi	**23**	**(0.00%)**	73.91%	17	(0.01%)	39.13%	3.87	00:02:51	0.00%	0	(0.00%)	$0.00	(0.00%)
70.	sr	**20**	**(0.00%)**	85.00%	17	(0.01%)	80.00%	1.60	00:00:36	0.00%	0	(0.00%)	$0.00	(0.00%)
71.	da-dk	**17**	**(0.00%)**	70.59%	12	(0.01%)	47.06%	4.47	00:01:17	0.00%	0	(0.00%)	$0.00	(0.00%)
72.	ar	**15**	**(0.00%)**	66.67%	10	(0.00%)	73.33%	3.73	00:01:46	0.00%	0	(0.00%)	$0.00	(0.00%)
73.	el-gr	**14**	**(0.00%)**	14.29%	2	(0.00%)	85.71%	2.93	00:05:03	0.00%	0	(0.00%)	$0.00	(0.00%)
74.	mn	**14**	**(0.00%)**	57.14%	8	(0.00%)	28.57%	3.00	00:04:20	0.00%	0	(0.00%)	$0.00	(0.00%)
75.	en_us	**11**	**(0.00%)**	90.91%	10	(0.00%)	63.64%	2.18	00:01:17	0.00%	0	(0.00%)	$0.00	(0.00%)
76.	mk	**11**	**(0.00%)**	45.45%	5	(0.00%)	81.82%	1.18	00:00:02	0.00%	0	(0.00%)	$0.00	(0.00%)
77.	nb	**11**	**(0.00%)**	90.91%	10	(0.00%)	63.64%	2.27	00:00:19	0.00%	0	(0.00%)	$0.00	(0.00%)
78.	en-ca	**9**	**(0.00%)**	77.78%	7	(0.00%)	66.67%	9.33	00:10:00	0.00%	0	(0.00%)	$0.00	(0.00%)
79.	de-ch	**6**	**(0.00%)**	50.00%	3	(0.00%)	33.33%	5.00	00:01:25	0.00%	0	(0.00%)	$0.00	(0.00%)
80.	et-ee	**6**	**(0.00%)**	100.00%	6	(0.00%)	66.67%	2.00	00:00:15	0.00%	0	(0.00%)	$0.00	(0.00%)
81.	eu	**6**	**(0.00%)**	83.33%	5	(0.00%)	66.67%	2.17	00:00:13	0.00%	0	(0.00%)	$0.00	(0.00%)
82.	c	**4**	**(0.00%)**	100.00%	4	(0.00%)	100.00%	1.00	00:00:00	0.00%	0	(0.00%)	$0.00	(0.00%)
83.	fil	**4**	**(0.00%)**	100.00%	4	(0.00%)	25.00%	5.50	00:02:27	0.00%	0	(0.00%)	$0.00	(0.00%)
84.	is	**3**	**(0.00%)**	100.00%	3	(0.00%)	66.67%	3.67	00:03:32	0.00%	0	(0.00%)	$0.00	(0.00%)
85.	sk-sk	**3**	**(0.00%)**	100.00%	3	(0.00%)	33.33%	2.67	00:00:48	0.00%	0	(0.00%)	$0.00	(0.00%)
86.	sq	**3**	**(0.00%)**	66.67%	2	(0.00%)	33.33%	4.33	00:00:58	0.00%	0	(0.00%)	$0.00	(0.00%)
87.	af	**2**	**(0.00%)**	50.00%	1	(0.00%)	50.00%	7.50	00:01:41	0.00%	0	(0.00%)	$0.00	(0.00%)
88.	ca-es	**2**	**(0.00%)**	100.00%	2	(0.00%)	50.00%	1.50	00:00:23	0.00%	0	(0.00%)	$0.00	(0.00%)
89.	en-au	**2**	**(0.00%)**	100.00%	2	(0.00%)	50.00%	2.50	00:00:14	0.00%	0	(0.00%)	$0.00	(0.00%)
90.	en-br; alexa	**2**	**(0.00%)**	100.00%	2	(0.00%)	50.00%	2.50	00:00:32	0.00%	0	(0.00%)	$0.00	(0.00%)
91.	en-us; megaupload 1.0	**2**	**(0.00%)**	50.00%	1	(0.00%)	50.00%	20.50	00:05:27	0.00%	0	(0.00%)	$0.00	(0.00%)
92.	he-il	**2**	**(0.00%)**	50.00%	1	(0.00%)	50.00%	2.00	00:00:44	0.00%	0	(0.00%)	$0.00	(0.00%)
93.	hi-in	**2**	**(0.00%)**	50.00%	1	(0.00%)	0.00%	2.50	00:00:53	0.00%	0	(0.00%)	$0.00	(0.00%)
94.	nn	**2**	**(0.00%)**	50.00%	1	(0.00%)	50.00%	13.00	00:02:47	0.00%	0	(0.00%)	$0.00	(0.00%)
95.	sr-ba-latn	**2**	**(0.00%)**	50.00%	1	(0.00%)	50.00%	2.50	00:00:53	0.00%	0	(0.00%)	$0.00	(0.00%)
96.	tr-tr	**2**	**(0.00%)**	100.00%	2	(0.00%)	50.00%	4.00	00:00:45	0.00%	0	(0.00%)	$0.00	(0.00%)
97.	uk-ua	**2**	**(0.00%)**	100.00%	2	(0.00%)	100.00%	1.00	00:00:00	0.00%	0	(0.00%)	$0.00	(0.00%)
98.	be	**1**	**(0.00%)**	100.00%	1	(0.00%)	0.00%	2.00	00:01:23	0.00%	0	(0.00%)	$0.00	(0.00%)
99.	bn-bd	**1**	**(0.00%)**	100.00%	1	(0.00%)	100.00%	1.00	00:00:00	0.00%	0	(0.00%)	$0.00	(0.00%)
100.	ca-ad	**1**	**(0.00%)**	100.00%	1	(0.00%)	0.00%	12.00	00:28:54	0.00%	0	(0.00%)	$0.00	(0.00%)

Rows 1 - 100 of 112

© 2014 Google
