# Supplementary material for: Trends in access of plant biodiversity data revealed by Google Analytics
Source: Biodivers Data J. 2014 Nov 11;(2):e1558. doi: 10.3897/BDJ.2.e1558 (PMC4238075; doi:10.3897/BDJ.2.e1558)
Supplement: Supplementary material 16 — Tropicos by year for language 4 [file biodiversity_data_journal-2-e1558-s016.pdf]

Language

Jun 1, 2010 - Jun 1, 2011

All Sessions
100.00%

+ Add Segment

Explorer

Summary

Language	Acquisition			Behavior			Conversions		
	Sessions	% New Sessions	New Users	Bounce Rate	Pages / Session	Avg. Session Duration	Goal Conversion Rate	Goal Completions	Goal Value
	1,083,185 % of Total: 100.00% (1,083,185)	22.64% Site Avg: 22.59% (0.23%)	245,287 % of Total: 100.23% (244,712)	25.52% Site Avg: 25.52% (0.00%)	14.65 Site Avg: 14.65 (0.00%)	00:14:34 Site Avg: 00:14:34 (0.00%)	0.00% Site Avg: 0.00% (0.00%)	0 % of Total: 0.00% (0)	$0.00 % of Total: 0.00% ($0.00)
1. en-us	**374,890** **(34.61%)**	21.50%	80,586 (32.85%)	27.70%	14.98	00:14:06	0.00%	0 (0.00%)	$0.00 (0.00%)
2. es	**194,385** **(17.95%)**	21.86%	42,499 (17.33%)	20.37%	16.68	00:16:45	0.00%	0 (0.00%)	$0.00 (0.00%)
3. pt-br	**155,097** **(14.32%)**	25.50%	39,542 (16.12%)	21.25%	12.48	00:13:21	0.00%	0 (0.00%)	$0.00 (0.00%)
4. es-es	**89,823** **(8.29%)**	18.64%	16,747 (6.83%)	33.15%	15.19	00:14:48	0.00%	0 (0.00%)	$0.00 (0.00%)
5. fr	**60,957** **(5.63%)**	21.77%	13,271 (5.41%)	19.04%	17.47	00:18:59	0.00%	0 (0.00%)	$0.00 (0.00%)
6. de	**38,713** **(3.57%)**	29.05%	11,246 (4.58%)	27.24%	11.49	00:13:16	0.00%	0 (0.00%)	$0.00 (0.00%)
7. en-gb	**22,770** **(2.10%)**	16.86%	3,838 (1.56%)	25.03%	12.09	00:14:01	0.00%	0 (0.00%)	$0.00 (0.00%)
8. zh-cn	**17,170** **(1.59%)**	22.23%	3,817 (1.56%)	17.83%	13.46	00:14:20	0.00%	0 (0.00%)	$0.00 (0.00%)
9. ru	**14,384** **(1.33%)**	38.65%	5,560 (2.27%)	42.64%	7.89	00:08:10	0.00%	0 (0.00%)	$0.00 (0.00%)
10. es-419	**11,645** **(1.08%)**	25.50%	2,969 (1.21%)	18.23%	18.16	00:16:34	0.00%	0 (0.00%)	$0.00 (0.00%)
11. en	**10,372** **(0.96%)**	20.65%	2,142 (0.87%)	45.78%	9.59	00:09:55	0.00%	0 (0.00%)	$0.00 (0.00%)
12. zh-tw	**9,893** **(0.91%)**	16.79%	1,661 (0.68%)	29.97%	7.83	00:11:13	0.00%	0 (0.00%)	$0.00 (0.00%)
13. it	**8,564** **(0.79%)**	28.69%	2,457 (1.00%)	21.45%	19.33	00:16:31	0.00%	0 (0.00%)	$0.00 (0.00%)
14. es-ar	**8,553** **(0.79%)**	23.00%	1,967 (0.80%)	23.13%	14.51	00:15:52	0.00%	0 (0.00%)	$0.00 (0.00%)
15. ko	**6,798** **(0.63%)**	20.02%	1,361 (0.55%)	17.11%	13.06	00:14:44	0.00%	0 (0.00%)	$0.00 (0.00%)
16. ja	**6,628** **(0.61%)**	22.33%	1,480 (0.60%)	23.30%	12.31	00:08:28	0.00%	0 (0.00%)	$0.00 (0.00%)
17. pl	**5,598** **(0.52%)**	27.55%	1,542 (0.63%)	34.71%	9.36	00:09:41	0.00%	0 (0.00%)	$0.00 (0.00%)
18. es-mx	**5,456** **(0.50%)**	17.82%	972 (0.40%)	18.91%	17.85	00:17:43	0.00%	0 (0.00%)	$0.00 (0.00%)
19. nl	**5,440** **(0.50%)**	32.24%	1,754 (0.72%)	27.50%	20.96	00:12:51	0.00%	0 (0.00%)	$0.00 (0.00%)
20. fr-fr	**4,768** **(0.44%)**	9.92%	473 (0.19%)	49.77%	11.04	00:09:31	0.00%	0 (0.00%)	$0.00 (0.00%)
21. de-de	**3,189** **(0.29%)**	24.62%	785 (0.32%)	43.99%	7.25	00:06:46	0.00%	0 (0.00%)	$0.00 (0.00%)
22. cs	**3,118** **(0.29%)**	34.45%	1,074 (0.44%)	29.44%	13.04	00:10:46	0.00%	0 (0.00%)	$0.00 (0.00%)
23. sv-se	**2,744** **(0.25%)**	12.50%	343 (0.14%)	21.76%	9.21	00:13:39	0.00%	0 (0.00%)	$0.00 (0.00%)
24. sv	**2,512** **(0.23%)**	18.79%	472 (0.19%)	16.68%	15.04	00:16:02	0.00%	0 (0.00%)	$0.00 (0.00%)
25. pt-pt	**2,302** **(0.21%)**	30.58%	704 (0.29%)	33.80%	10.34	00:10:04	0.00%	0 (0.00%)	$0.00 (0.00%)
26. tr	**1,325** **(0.12%)**	44.68%	592 (0.24%)	28.38%	11.73	00:11:45	0.00%	0 (0.00%)	$0.00 (0.00%)

27.	pt	**1,305**	**(0.12%)**	45.52%	594	(0.24%)	32.26%	9.75	00:09:39	0.00%	0	(0.00%)	$0.00	(0.00%)
28.	ja-jp	**1,130**	**(0.10%)**	14.25%	161	(0.07%)	44.51%	6.35	00:05:27	0.00%	0	(0.00%)	$0.00	(0.00%)
29.	es-xl	**1,097**	**(0.10%)**	0.64%	7	(0.00%)	54.51%	6.64	00:06:09	0.00%	0	(0.00%)	$0.00	(0.00%)
30.	es-la	**1,080**	**(0.10%)**	12.13%	131	(0.05%)	45.93%	12.76	00:12:48	0.00%	0	(0.00%)	$0.00	(0.00%)
31.	da	**979**	**(0.09%)**	32.07%	314	(0.13%)	24.21%	11.03	00:08:59	0.00%	0	(0.00%)	$0.00	(0.00%)
32.	fr-ca	**979**	**(0.09%)**	0.10%	1	(0.00%)	3.88%	68.45	00:56:15	0.00%	0	(0.00%)	$0.00	(0.00%)
33.	ja-jp-mac	**873**	**(0.08%)**	20.73%	181	(0.07%)	19.47%	30.70	00:15:18	0.00%	0	(0.00%)	$0.00	(0.00%)
34.	sk	**813**	**(0.08%)**	29.64%	241	(0.10%)	33.09%	20.95	00:21:00	0.00%	0	(0.00%)	$0.00	(0.00%)
35.	th	**800**	**(0.07%)**	21.75%	174	(0.07%)	23.88%	10.09	00:12:36	0.00%	0	(0.00%)	$0.00	(0.00%)
36.	el	**622**	**(0.06%)**	51.61%	321	(0.13%)	35.85%	7.25	00:06:32	0.00%	0	(0.00%)	$0.00	(0.00%)
37.	ca	**621**	**(0.06%)**	33.98%	211	(0.09%)	21.10%	10.60	00:10:21	0.00%	0	(0.00%)	$0.00	(0.00%)
38.	hu	**579**	**(0.05%)**	81.17%	470	(0.19%)	57.34%	4.67	00:02:29	0.00%	0	(0.00%)	$0.00	(0.00%)
39.	es-cl	**541**	**(0.05%)**	39.37%	213	(0.09%)	17.01%	10.43	00:13:28	0.00%	0	(0.00%)	$0.00	(0.00%)
40.	fi	**445**	**(0.04%)**	51.24%	228	(0.09%)	31.46%	8.92	00:05:44	0.00%	0	(0.00%)	$0.00	(0.00%)
41.	et	**358**	**(0.03%)**	36.31%	130	(0.05%)	30.45%	5.33	00:05:13	0.00%	0	(0.00%)	$0.00	(0.00%)
42.	vi	**334**	**(0.03%)**	41.32%	138	(0.06%)	32.04%	8.44	00:10:49	0.00%	0	(0.00%)	$0.00	(0.00%)
43.	no	**324**	**(0.03%)**	36.73%	119	(0.05%)	23.46%	28.60	00:19:05	0.00%	0	(0.00%)	$0.00	(0.00%)
44.	id	**269**	**(0.02%)**	80.67%	217	(0.09%)	48.70%	4.03	00:06:32	0.00%	0	(0.00%)	$0.00	(0.00%)
45.	nl-nl	**265**	**(0.02%)**	21.13%	56	(0.02%)	45.28%	7.39	00:06:15	0.00%	0	(0.00%)	$0.00	(0.00%)
46.	it-it	**249**	**(0.02%)**	42.17%	105	(0.04%)	39.36%	5.85	00:04:49	0.00%	0	(0.00%)	$0.00	(0.00%)
47.	nb-no	**202**	**(0.02%)**	25.25%	51	(0.02%)	28.71%	57.02	00:21:59	0.00%	0	(0.00%)	$0.00	(0.00%)
48.	de-at	**197**	**(0.02%)**	80.71%	159	(0.06%)	6.09%	16.96	00:49:08	0.00%	0	(0.00%)	$0.00	(0.00%)
49.	lv	**172**	**(0.02%)**	35.47%	61	(0.02%)	18.60%	28.39	00:19:29	0.00%	0	(0.00%)	$0.00	(0.00%)
50.	zh-hk	**161**	**(0.01%)**	40.37%	65	(0.03%)	46.58%	9.81	00:05:47	0.00%	0	(0.00%)	$0.00	(0.00%)
51.	ro	**155**	**(0.01%)**	71.61%	111	(0.05%)	59.35%	4.34	00:02:47	0.00%	0	(0.00%)	$0.00	(0.00%)
52.	lt	**143**	**(0.01%)**	50.35%	72	(0.03%)	44.06%	18.14	00:11:17	0.00%	0	(0.00%)	$0.00	(0.00%)
53.	uk	**141**	**(0.01%)**	56.74%	80	(0.03%)	39.01%	15.46	00:09:01	0.00%	0	(0.00%)	$0.00	(0.00%)
54.	bg	**137**	**(0.01%)**	66.42%	91	(0.04%)	54.74%	3.88	00:01:32	0.00%	0	(0.00%)	$0.00	(0.00%)
55.	nb	**122**	**(0.01%)**	14.75%	18	(0.01%)	36.07%	37.66	00:16:13	0.00%	0	(0.00%)	$0.00	(0.00%)
56.	sl	**116**	**(0.01%)**	76.72%	89	(0.04%)	61.21%	2.91	00:01:00	0.00%	0	(0.00%)	$0.00	(0.00%)
57.	he	**86**	**(0.01%)**	89.53%	77	(0.03%)	54.65%	4.03	00:01:57	0.00%	0	(0.00%)	$0.00	(0.00%)
58.	ru-ru	**76**	**(0.01%)**	67.11%	51	(0.02%)	61.84%	2.29	00:01:00	0.00%	0	(0.00%)	$0.00	(0.00%)
59.	ar-sa	**68**	**(0.01%)**	88.24%	60	(0.02%)	54.41%	6.78	00:05:48	0.00%	0	(0.00%)	$0.00	(0.00%)
60.	hr	**63**	**(0.01%)**	85.71%	54	(0.02%)	55.56%	5.92	00:04:03	0.00%	0	(0.00%)	$0.00	(0.00%)
61.	ar	**61**	**(0.01%)**	81.97%	50	(0.02%)	50.82%	3.56	00:03:29	0.00%	0	(0.00%)	$0.00	(0.00%)
62.	pl-pl	**52**	**(0.00%)**	78.85%	41	(0.02%)	65.38%	2.52	00:00:51	0.00%	0	(0.00%)	$0.00	(0.00%)
63.	sr	**49**	**(0.00%)**	65.31%	32	(0.01%)	57.14%	3.04	00:01:38	0.00%	0	(0.00%)	$0.00	(0.00%)
64.	da-dk	**42**	**(0.00%)**	66.67%	28	(0.01%)	42.86%	11.45	00:05:28	0.00%	0	(0.00%)	$0.00	(0.00%)
65.	ko-kr	**42**	**(0.00%)**	26.19%	11	(0.00%)	45.24%	3.88	00:03:47	0.00%	0	(0.00%)	$0.00	(0.00%)
66.	th-th	**37**	**(0.00%)**	8.11%	3	(0.00%)	70.27%	2.32	00:01:55	0.00%	0	(0.00%)	$0.00	(0.00%)

67.	fil	**28**	**(0.00%)**	64.29%	18	(0.01%)	32.14%	4.00	00:05:06	0.00%	0	(0.00%)	$0.00	(0.00%)
68.	c	**26**	**(0.00%)**	84.62%	22	(0.01%)	96.15%	1.12	00:00:07	0.00%	0	(0.00%)	$0.00	(0.00%)
69.	cs-cz	**20**	**(0.00%)**	65.00%	13	(0.01%)	35.00%	5.30	00:03:54	0.00%	0	(0.00%)	$0.00	(0.00%)
70.	hu-hu	**19**	**(0.00%)**	63.16%	12	(0.00%)	63.16%	2.42	00:00:35	0.00%	0	(0.00%)	$0.00	(0.00%)
71.	el-gr	**17**	**(0.00%)**	58.82%	10	(0.00%)	52.94%	3.53	00:01:52	0.00%	0	(0.00%)	$0.00	(0.00%)
72.	(not set)	**16**	**(0.00%)**	87.50%	14	(0.01%)	50.00%	3.62	00:04:54	0.00%	0	(0.00%)	$0.00	(0.00%)
73.	fi-fi	**15**	**(0.00%)**	80.00%	12	(0.00%)	60.00%	2.53	00:01:03	0.00%	0	(0.00%)	$0.00	(0.00%)
74.	en_us	**14**	**(0.00%)**	92.86%	13	(0.01%)	57.14%	4.00	00:03:36	0.00%	0	(0.00%)	$0.00	(0.00%)
75.	en-au	**14**	**(0.00%)**	35.71%	5	(0.00%)	14.29%	8.07	00:05:02	0.00%	0	(0.00%)	$0.00	(0.00%)
76.	ca-es	**13**	**(0.00%)**	46.15%	6	(0.00%)	53.85%	3.62	00:00:57	0.00%	0	(0.00%)	$0.00	(0.00%)
77.	pt-br; alexa	**11**	**(0.00%)**	81.82%	9	(0.00%)	45.45%	6.00	00:02:52	0.00%	0	(0.00%)	$0.00	(0.00%)
78.	fa	**10**	**(0.00%)**	60.00%	6	(0.00%)	50.00%	5.80	00:03:31	0.00%	0	(0.00%)	$0.00	(0.00%)
79.	en-ca	**9**	**(0.00%)**	88.89%	8	(0.00%)	44.44%	3.22	00:01:02	0.00%	0	(0.00%)	$0.00	(0.00%)
80.	ms	**9**	**(0.00%)**	0.00%	0	(0.00%)	33.33%	6.67	00:07:02	0.00%	0	(0.00%)	$0.00	(0.00%)
81.	lv-lv	**7**	**(0.00%)**	57.14%	4	(0.00%)	42.86%	4.14	00:04:50	0.00%	0	(0.00%)	$0.00	(0.00%)
82.	eu	**6**	**(0.00%)**	100.00%	6	(0.00%)	50.00%	4.17	00:04:58	0.00%	0	(0.00%)	$0.00	(0.00%)
83.	gl	**5**	**(0.00%)**	80.00%	4	(0.00%)	0.00%	4.00	00:03:01	0.00%	0	(0.00%)	$0.00	(0.00%)
84.	mn	**5**	**(0.00%)**	60.00%	3	(0.00%)	100.00%	1.00	00:00:00	0.00%	0	(0.00%)	$0.00	(0.00%)
85.	af-za	**4**	**(0.00%)**	75.00%	3	(0.00%)	25.00%	20.25	00:21:14	0.00%	0	(0.00%)	$0.00	(0.00%)
86.	be	**4**	**(0.00%)**	50.00%	2	(0.00%)	50.00%	208.00	01:11:04	0.00%	0	(0.00%)	$0.00	(0.00%)
87.	en-in	**4**	**(0.00%)**	100.00%	4	(0.00%)	75.00%	4.50	00:04:22	0.00%	0	(0.00%)	$0.00	(0.00%)
88.	en-za	**4**	**(0.00%)**	75.00%	3	(0.00%)	50.00%	33.50	00:10:20	0.00%	0	(0.00%)	$0.00	(0.00%)
89.	is	**4**	**(0.00%)**	75.00%	3	(0.00%)	50.00%	3.75	00:01:52	0.00%	0	(0.00%)	$0.00	(0.00%)
90.	mk	**4**	**(0.00%)**	100.00%	4	(0.00%)	75.00%	2.00	00:00:29	0.00%	0	(0.00%)	$0.00	(0.00%)
91.	tr-tr	**4**	**(0.00%)**	75.00%	3	(0.00%)	75.00%	1.25	00:00:05	0.00%	0	(0.00%)	$0.00	(0.00%)
92.	de-ch	**3**	**(0.00%)**	100.00%	3	(0.00%)	33.33%	2.00	00:00:05	0.00%	0	(0.00%)	$0.00	(0.00%)
93.	cy	**2**	**(0.00%)**	50.00%	1	(0.00%)	50.00%	3.50	00:00:21	0.00%	0	(0.00%)	$0.00	(0.00%)
94.	en-br; megaupload	**2**	**(0.00%)**	100.00%	2	(0.00%)	0.00%	7.50	00:03:07	0.00%	0	(0.00%)	$0.00	(0.00%)
95.	et-ee	**2**	**(0.00%)**	100.00%	2	(0.00%)	0.00%	2.50	00:00:20	0.00%	0	(0.00%)	$0.00	(0.00%)
96.	hi	**2**	**(0.00%)**	100.00%	2	(0.00%)	100.00%	1.00	00:00:00	0.00%	0	(0.00%)	$0.00	(0.00%)
97.	ru; alexa toolbar	**2**	**(0.00%)**	0.00%	0	(0.00%)	50.00%	1.50	00:00:12	0.00%	0	(0.00%)	$0.00	(0.00%)
98.	ast	**1**	**(0.00%)**	100.00%	1	(0.00%)	0.00%	5.00	00:00:36	0.00%	0	(0.00%)	$0.00	(0.00%)
99.	bg-bg	**1**	**(0.00%)**	100.00%	1	(0.00%)	100.00%	1.00	00:00:00	0.00%	0	(0.00%)	$0.00	(0.00%)
100.	bn	**1**	**(0.00%)**	100.00%	1	(0.00%)	0.00%	5.00	00:02:38	0.00%	0	(0.00%)	$0.00	(0.00%)

Rows 1 - 100 of 118

© 2014 Google
