# Supplementary material for: Trends in access of plant biodiversity data revealed by Google Analytics
Source: Biodivers Data J. 2014 Nov 11;(2):e1558. doi: 10.3897/BDJ.2.e1558 (PMC4238075; doi:10.3897/BDJ.2.e1558)
Supplement: Supplementary material 17 — Tropicos by year for languages [file biodiversity_data_journal-2-e1558-s017.pdf]

Language

Jun 1, 2011 - Jun 1, 2012

All Sessions 100.00%

+ Add Segment

Explorer

Summary

Language	Acquisition			Behavior			Conversions		
	Sessions	% New Sessions	New Users	Bounce Rate	Pages / Session	Avg. Session Duration	Goal Conversion Rate	Goal Completions	Goal Value
	1,544,270 % of Total: 100.00% (1,544,270)	30.60% Site Avg: 30.54% (0.17%)	472,479 % of Total: 100.17% (471,694)	34.36% Site Avg: 34.36% (0.00%)	12.00 Site Avg: 12.00 (0.00%)	00:12:08 Site Avg: 00:12:08 (0.00%)	0.00% Site Avg: 0.00% (0.00%)	0 % of Total: 0.00% (0)	$0.00 % of Total: 0.00% ($0.00)
1. en-us	**545,540** **(35.33%)**	32.48%	177,190 (37.50%)	37.76%	12.24	00:11:32	0.00%	0 (0.00%)	$0.00 (0.00%)
2. es	**260,751** **(16.89%)**	28.12%	73,331 (15.52%)	26.59%	14.51	00:14:53	0.00%	0 (0.00%)	$0.00 (0.00%)
3. pt-br	**185,924** **(12.04%)**	26.74%	49,722 (10.52%)	26.02%	11.00	00:12:23	0.00%	0 (0.00%)	$0.00 (0.00%)
4. es-es	**121,417** **(7.86%)**	27.23%	33,067 (7.00%)	42.45%	11.96	00:11:55	0.00%	0 (0.00%)	$0.00 (0.00%)
5. fr	**84,517** **(5.47%)**	27.69%	23,399 (4.95%)	26.97%	14.84	00:16:41	0.00%	0 (0.00%)	$0.00 (0.00%)
6. de	**36,752** **(2.38%)**	34.06%	12,519 (2.65%)	38.44%	8.35	00:10:13	0.00%	0 (0.00%)	$0.00 (0.00%)
7. en-gb	**34,354** **(2.22%)**	31.21%	10,723 (2.27%)	36.28%	8.66	00:09:06	0.00%	0 (0.00%)	$0.00 (0.00%)
8. de-de	**30,842** **(2.00%)**	35.47%	10,939 (2.32%)	39.77%	8.35	00:08:53	0.00%	0 (0.00%)	$0.00 (0.00%)
9. zh-cn	**27,562** **(1.78%)**	25.25%	6,959 (1.47%)	23.97%	14.09	00:15:17	0.00%	0 (0.00%)	$0.00 (0.00%)
10. es-419	**17,663** **(1.14%)**	27.76%	4,904 (1.04%)	26.01%	13.43	00:14:01	0.00%	0 (0.00%)	$0.00 (0.00%)
11. zh-tw	**15,058** **(0.98%)**	21.88%	3,295 (0.70%)	36.66%	7.25	00:09:20	0.00%	0 (0.00%)	$0.00 (0.00%)
12. en	**14,698** **(0.95%)**	43.22%	6,352 (1.34%)	53.97%	7.11	00:07:01	0.00%	0 (0.00%)	$0.00 (0.00%)
13. ru	**14,561** **(0.94%)**	36.12%	5,260 (1.11%)	45.04%	6.67	00:06:35	0.00%	0 (0.00%)	$0.00 (0.00%)
14. ja	**13,338** **(0.86%)**	29.30%	3,908 (0.83%)	32.21%	13.72	00:09:34	0.00%	0 (0.00%)	$0.00 (0.00%)
15. es-mx	**12,397** **(0.80%)**	27.01%	3,349 (0.71%)	19.55%	18.75	00:17:52	0.00%	0 (0.00%)	$0.00 (0.00%)
16. it	**10,426** **(0.68%)**	48.27%	5,033 (1.07%)	46.26%	6.77	00:06:10	0.00%	0 (0.00%)	$0.00 (0.00%)
17. nl	**10,387** **(0.67%)**	43.14%	4,481 (0.95%)	45.80%	8.22	00:07:38	0.00%	0 (0.00%)	$0.00 (0.00%)
18. ko	**10,267** **(0.66%)**	27.53%	2,826 (0.60%)	26.78%	12.14	00:14:30	0.00%	0 (0.00%)	$0.00 (0.00%)
19. pl	**10,121** **(0.66%)**	34.86%	3,528 (0.75%)	48.35%	9.72	00:08:42	0.00%	0 (0.00%)	$0.00 (0.00%)
20. fr-fr	**8,270** **(0.54%)**	24.67%	2,040 (0.43%)	53.80%	6.30	00:05:49	0.00%	0 (0.00%)	$0.00 (0.00%)
21. cs	**8,091** **(0.52%)**	33.27%	2,692 (0.57%)	43.65%	7.84	00:07:29	0.00%	0 (0.00%)	$0.00 (0.00%)
22. es-ar	**7,697** **(0.50%)**	29.34%	2,258 (0.48%)	21.28%	13.50	00:14:14	0.00%	0 (0.00%)	$0.00 (0.00%)
23. it-it	**4,443** **(0.29%)**	46.05%	2,046 (0.43%)	46.66%	6.97	00:05:27	0.00%	0 (0.00%)	$0.00 (0.00%)
24. ru-ru	**4,193** **(0.27%)**	31.96%	1,340 (0.28%)	45.53%	6.77	00:06:13	0.00%	0 (0.00%)	$0.00 (0.00%)
25. sv-se	**4,078** **(0.26%)**	17.02%	694 (0.15%)	38.38%	6.35	00:08:14	0.00%	0 (0.00%)	$0.00 (0.00%)
26. tr	**3,972** **(0.26%)**	49.62%	1,971 (0.42%)	43.30%	7.12	00:06:26	0.00%	0 (0.00%)	$0.00 (0.00%)

27.	sk	**3,546**	**(0.23%)**	18.50%	656	(0.14%)	37.08%	8.66	00:09:12	0.00%	0	(0.00%)	$0.00	(0.00%)
28.	sv	**3,429**	**(0.22%)**	32.93%	1,129	(0.24%)	35.23%	8.18	00:11:04	0.00%	0	(0.00%)	$0.00	(0.00%)
29.	pt-pt	**3,289**	**(0.21%)**	39.50%	1,299	(0.27%)	39.65%	8.01	00:08:11	0.00%	0	(0.00%)	$0.00	(0.00%)
30.	pt	**2,267**	**(0.15%)**	48.21%	1,093	(0.23%)	34.94%	17.04	00:14:23	0.00%	0	(0.00%)	$0.00	(0.00%)
31.	ja-jp	**2,097**	**(0.14%)**	21.41%	449	(0.10%)	43.68%	5.03	00:04:39	0.00%	0	(0.00%)	$0.00	(0.00%)
32.	da	**1,950**	**(0.13%)**	47.74%	931	(0.20%)	46.82%	5.91	00:05:29	0.00%	0	(0.00%)	$0.00	(0.00%)
33.	id	**1,561**	**(0.10%)**	58.74%	917	(0.19%)	57.72%	5.61	00:06:34	0.00%	0	(0.00%)	$0.00	(0.00%)
34.	ko-kr	**1,456**	**(0.09%)**	7.83%	114	(0.02%)	24.31%	20.29	00:22:08	0.00%	0	(0.00%)	$0.00	(0.00%)
35.	hu	**1,453**	**(0.09%)**	58.29%	847	(0.18%)	64.35%	3.26	00:02:56	0.00%	0	(0.00%)	$0.00	(0.00%)
36.	th	**1,359**	**(0.09%)**	45.33%	616	(0.13%)	58.13%	5.12	00:04:46	0.00%	0	(0.00%)	$0.00	(0.00%)
37.	es-la	**1,352**	**(0.09%)**	15.24%	206	(0.04%)	43.71%	9.45	00:08:48	0.00%	0	(0.00%)	$0.00	(0.00%)
38.	vi	**1,218**	**(0.08%)**	46.22%	563	(0.12%)	40.97%	7.97	00:08:42	0.00%	0	(0.00%)	$0.00	(0.00%)
39.	es-xl	**1,026**	**(0.07%)**	6.24%	64	(0.01%)	86.84%	2.68	00:01:31	0.00%	0	(0.00%)	$0.00	(0.00%)
40.	es-cl	**973**	**(0.06%)**	38.64%	376	(0.08%)	32.37%	8.17	00:08:08	0.00%	0	(0.00%)	$0.00	(0.00%)
41.	fi-fi	**932**	**(0.06%)**	36.91%	344	(0.07%)	34.76%	8.08	00:10:34	0.00%	0	(0.00%)	$0.00	(0.00%)
42.	el	**921**	**(0.06%)**	63.74%	587	(0.12%)	62.65%	4.06	00:02:39	0.00%	0	(0.00%)	$0.00	(0.00%)
43.	ca	**871**	**(0.06%)**	49.25%	429	(0.09%)	35.13%	8.38	00:07:30	0.00%	0	(0.00%)	$0.00	(0.00%)
44.	fi	**838**	**(0.05%)**	43.20%	362	(0.08%)	48.57%	7.98	00:04:12	0.00%	0	(0.00%)	$0.00	(0.00%)
45.	hu-hu	**823**	**(0.05%)**	67.19%	553	(0.12%)	68.65%	2.69	00:01:47	0.00%	0	(0.00%)	$0.00	(0.00%)
46.	th-th	**780**	**(0.05%)**	28.97%	226	(0.05%)	37.44%	6.72	00:07:26	0.00%	0	(0.00%)	$0.00	(0.00%)
47.	tr-tr	**755**	**(0.05%)**	36.42%	275	(0.06%)	54.04%	5.49	00:05:27	0.00%	0	(0.00%)	$0.00	(0.00%)
48.	nl-nl	**748**	**(0.05%)**	46.26%	346	(0.07%)	74.20%	3.63	00:01:39	0.00%	0	(0.00%)	$0.00	(0.00%)
49.	lt	**742**	**(0.05%)**	31.54%	234	(0.05%)	41.78%	8.24	00:10:11	0.00%	0	(0.00%)	$0.00	(0.00%)
50.	en-au	**688**	**(0.04%)**	17.30%	119	(0.03%)	22.09%	7.23	00:10:05	0.00%	0	(0.00%)	$0.00	(0.00%)
51.	no	**672**	**(0.04%)**	34.23%	230	(0.05%)	42.11%	5.43	00:04:20	0.00%	0	(0.00%)	$0.00	(0.00%)
52.	ar	**630**	**(0.04%)**	45.71%	288	(0.06%)	52.54%	4.98	00:06:06	0.00%	0	(0.00%)	$0.00	(0.00%)
53.	zh-hk	**628**	**(0.04%)**	28.82%	181	(0.04%)	35.19%	6.02	00:04:40	0.00%	0	(0.00%)	$0.00	(0.00%)
54.	bg	**581**	**(0.04%)**	64.54%	375	(0.08%)	73.67%	2.59	00:01:33	0.00%	0	(0.00%)	$0.00	(0.00%)
55.	et	**578**	**(0.04%)**	41.87%	242	(0.05%)	44.12%	4.97	00:04:12	0.00%	0	(0.00%)	$0.00	(0.00%)
56.	nb-no	**555**	**(0.04%)**	33.87%	188	(0.04%)	36.58%	7.68	00:08:00	0.00%	0	(0.00%)	$0.00	(0.00%)
57.	fr-ca	**549**	**(0.04%)**	6.92%	38	(0.01%)	10.56%	73.35	01:00:06	0.00%	0	(0.00%)	$0.00	(0.00%)
58.	el-gr	**496**	**(0.03%)**	61.29%	304	(0.06%)	58.67%	4.39	00:03:26	0.00%	0	(0.00%)	$0.00	(0.00%)
59.	vi-vn	**431**	**(0.03%)**	46.40%	200	(0.04%)	49.88%	5.44	00:04:32	0.00%	0	(0.00%)	$0.00	(0.00%)
60.	he	**426**	**(0.03%)**	72.54%	309	(0.07%)	56.57%	4.27	00:02:28	0.00%	0	(0.00%)	$0.00	(0.00%)
61.	ro	**389**	**(0.03%)**	70.95%	276	(0.06%)	70.44%	2.51	00:01:30	0.00%	0	(0.00%)	$0.00	(0.00%)
62.	fil	**358**	**(0.02%)**	72.63%	260	(0.06%)	62.85%	2.78	00:02:12	0.00%	0	(0.00%)	$0.00	(0.00%)
63.	sl	**339**	**(0.02%)**	70.50%	239	(0.05%)	75.52%	2.61	00:00:57	0.00%	0	(0.00%)	$0.00	(0.00%)
64.	da-dk	**320**	**(0.02%)**	39.06%	125	(0.03%)	79.69%	2.10	00:01:29	0.00%	0	(0.00%)	$0.00	(0.00%)
65.	uk	**315**	**(0.02%)**	55.87%	176	(0.04%)	54.92%	4.40	00:05:00	0.00%	0	(0.00%)	$0.00	(0.00%)
66.	ja-jp-mac	**313**	**(0.02%)**	32.59%	102	(0.02%)	28.12%	9.31	00:06:28	0.00%	0	(0.00%)	$0.00	(0.00%)

67.	de-at	**301**	**(0.02%)**	45.51%	137	(0.03%)	16.28%	8.28	00:08:02	0.00%	0	(0.00%)	$0.00	(0.00%)
68.	ro-ro	**296**	**(0.02%)**	35.14%	104	(0.02%)	45.27%	12.60	00:10:57	0.00%	0	(0.00%)	$0.00	(0.00%)
69.	es-co	**295**	**(0.02%)**	12.20%	36	(0.01%)	73.56%	7.82	00:05:40	0.00%	0	(0.00%)	$0.00	(0.00%)
70.	ar-sa	**251**	**(0.02%)**	78.09%	196	(0.04%)	68.13%	2.37	00:01:26	0.00%	0	(0.00%)	$0.00	(0.00%)
71.	lv	**206**	**(0.01%)**	71.84%	148	(0.03%)	77.18%	1.86	00:00:52	0.00%	0	(0.00%)	$0.00	(0.00%)
72.	sr	**205**	**(0.01%)**	72.68%	149	(0.03%)	74.63%	1.64	00:01:02	0.00%	0	(0.00%)	$0.00	(0.00%)
73.	hr	**202**	**(0.01%)**	78.22%	158	(0.03%)	67.33%	2.95	00:01:55	0.00%	0	(0.00%)	$0.00	(0.00%)
74.	es-pe	**198**	**(0.01%)**	7.58%	15	(0.00%)	11.11%	11.24	00:09:43	0.00%	0	(0.00%)	$0.00	(0.00%)
75.	hr-hr	**190**	**(0.01%)**	78.95%	150	(0.03%)	38.95%	4.31	00:02:31	0.00%	0	(0.00%)	$0.00	(0.00%)
76.	nb	**171**	**(0.01%)**	52.63%	90	(0.02%)	48.54%	5.29	00:03:45	0.00%	0	(0.00%)	$0.00	(0.00%)
77.	es-us	**168**	**(0.01%)**	39.29%	66	(0.01%)	14.29%	9.01	00:07:37	0.00%	0	(0.00%)	$0.00	(0.00%)
78.	en-za	**129**	**(0.01%)**	75.19%	97	(0.02%)	63.57%	2.38	00:01:42	0.00%	0	(0.00%)	$0.00	(0.00%)
79.	(not set)	**124**	**(0.01%)**	67.74%	84	(0.02%)	42.74%	7.81	00:06:20	0.00%	0	(0.00%)	$0.00	(0.00%)
80.	pl-pl	**114**	**(0.01%)**	59.65%	68	(0.01%)	66.67%	23.61	00:04:47	0.00%	0	(0.00%)	$0.00	(0.00%)
81.	es-bo	**112**	**(0.01%)**	0.89%	1	(0.00%)	10.71%	49.59	00:31:36	0.00%	0	(0.00%)	$0.00	(0.00%)
82.	en-in	**98**	**(0.01%)**	94.90%	93	(0.02%)	65.31%	1.63	00:00:42	0.00%	0	(0.00%)	$0.00	(0.00%)
83.	fa	**90**	**(0.01%)**	42.22%	38	(0.01%)	67.78%	2.20	00:03:03	0.00%	0	(0.00%)	$0.00	(0.00%)
84.	en-ca	**84**	**(0.01%)**	71.43%	60	(0.01%)	53.57%	6.23	00:02:14	0.00%	0	(0.00%)	$0.00	(0.00%)
85.	es-ve	**74**	**(0.00%)**	44.59%	33	(0.01%)	27.03%	7.68	00:10:50	0.00%	0	(0.00%)	$0.00	(0.00%)
86.	c	**70**	**(0.00%)**	98.57%	69	(0.01%)	95.71%	1.09	00:00:06	0.00%	0	(0.00%)	$0.00	(0.00%)
87.	de-ch	**69**	**(0.00%)**	34.78%	24	(0.01%)	46.38%	17.45	00:05:46	0.00%	0	(0.00%)	$0.00	(0.00%)
88.	en-us.<br>en	**66**	**(0.00%)**	1.52%	1	(0.00%)	95.45%	1.20	00:00:11	0.00%	0	(0.00%)	$0.00	(0.00%)
89.	en_us	**62**	**(0.00%)**	93.55%	58	(0.01%)	67.74%	2.10	00:01:11	0.00%	0	(0.00%)	$0.00	(0.00%)
90.	cs-cz	**56**	**(0.00%)**	92.86%	52	(0.01%)	73.21%	2.96	00:01:14	0.00%	0	(0.00%)	$0.00	(0.00%)
91.	es-uy	**48**	**(0.00%)**	62.50%	30	(0.01%)	22.92%	12.88	00:11:32	0.00%	0	(0.00%)	$0.00	(0.00%)
92.	en_gb	**42**	**(0.00%)**	92.86%	39	(0.01%)	73.81%	3.00	00:03:34	0.00%	0	(0.00%)	$0.00	(0.00%)
93.	ca-es	**41**	**(0.00%)**	48.78%	20	(0.00%)	46.34%	3.90	00:01:33	0.00%	0	(0.00%)	$0.00	(0.00%)
94.	fr-ch	**32**	**(0.00%)**	46.88%	15	(0.00%)	53.12%	3.31	00:03:09	0.00%	0	(0.00%)	$0.00	(0.00%)
95.	fa-ir	**26**	**(0.00%)**	69.23%	18	(0.00%)	73.08%	3.65	00:01:36	0.00%	0	(0.00%)	$0.00	(0.00%)
96.	en-nz	**24**	**(0.00%)**	62.50%	15	(0.00%)	79.17%	2.08	00:00:29	0.00%	0	(0.00%)	$0.00	(0.00%)
97.	es-cr	**23**	**(0.00%)**	91.30%	21	(0.00%)	39.13%	6.96	00:04:20	0.00%	0	(0.00%)	$0.00	(0.00%)
98.	es-<br>es_tradnl	**22**	**(0.00%)**	31.82%	7	(0.00%)	22.73%	8.50	00:09:18	0.00%	0	(0.00%)	$0.00	(0.00%)
99.	eu	**22**	**(0.00%)**	63.64%	14	(0.00%)	77.27%	5.59	00:08:13	0.00%	0	(0.00%)	$0.00	(0.00%)
100.	gl-gl	**22**	**(0.00%)**	54.55%	12	(0.00%)	45.45%	5.00	00:04:55	0.00%	0	(0.00%)	$0.00	(0.00%)

Rows 1 - 100 of 189

© 2014 Google
