# Supplementary material for: Trends in access of plant biodiversity data revealed by Google Analytics
Source: Biodivers Data J. 2014 Nov 11;(2):e1558. doi: 10.3897/BDJ.2.e1558 (PMC4238075; doi:10.3897/BDJ.2.e1558)
Supplement: Supplementary material 18 — Tropicos by year for language [file biodiversity_data_journal-2-e1558-s018.pdf]

Language

Jun 1, 2012 - Jun 1, 2013

All Sessions
100.00%

+ Add Segment

Explorer

Summary

Language	Acquisition			Behavior			Conversions		
	Sessions	% New Sessions	New Users	Bounce Rate	Pages / Session	Avg. Session Duration	Goal Conversion Rate	Goal Completions	Goal Value
	1,578,586 % of Total: 100.00% (1,578,586)	30.63% Site Avg: 30.58% (0.17%)	483,567 % of Total: 100.17% (482,754)	33.18% Site Avg: 33.18% (0.00%)	12.04 Site Avg: 12.04 (0.00%)	00:12:20 Site Avg: 00:12:20 (0.00%)	0.00% Site Avg: 0.00% (0.00%)	0 % of Total: 0.00% (0)	$0.00 % of Total: 0.00% ($0.00)
1. en-us	**550,095** **(34.85%)**	33.95%	186,745 (38.62%)	37.26%	12.07	00:11:28	0.00%	0 (0.00%)	$0.00 (0.00%)
2. es	**294,913** **(18.68%)**	28.69%	84,603 (17.50%)	26.17%	14.56	00:15:08	0.00%	0 (0.00%)	$0.00 (0.00%)
3. pt-br	**180,046** **(11.41%)**	26.78%	48,221 (9.97%)	26.08%	10.66	00:11:58	0.00%	0 (0.00%)	$0.00 (0.00%)
4. es-es	**126,197** **(7.99%)**	23.15%	29,212 (6.04%)	41.48%	11.79	00:12:05	0.00%	0 (0.00%)	$0.00 (0.00%)
5. fr	**80,477** **(5.10%)**	27.08%	21,796 (4.51%)	24.84%	13.63	00:16:38	0.00%	0 (0.00%)	$0.00 (0.00%)
6. de-de	**35,556** **(2.25%)**	36.79%	13,080 (2.70%)	36.29%	9.78	00:10:00	0.00%	0 (0.00%)	$0.00 (0.00%)
7. zh-cn	**31,605** **(2.00%)**	23.02%	7,274 (1.50%)	22.25%	12.53	00:14:32	0.00%	0 (0.00%)	$0.00 (0.00%)
8. en-gb	**30,474** **(1.93%)**	32.78%	9,990 (2.07%)	36.93%	10.07	00:10:27	0.00%	0 (0.00%)	$0.00 (0.00%)
9. es-419	**26,486** **(1.68%)**	27.12%	7,182 (1.49%)	26.92%	15.68	00:14:37	0.00%	0 (0.00%)	$0.00 (0.00%)
10. de	**26,317** **(1.67%)**	32.70%	8,606 (1.78%)	36.69%	9.37	00:10:54	0.00%	0 (0.00%)	$0.00 (0.00%)
11. es-mx	**16,939** **(1.07%)**	27.51%	4,660 (0.96%)	18.10%	17.53	00:17:14	0.00%	0 (0.00%)	$0.00 (0.00%)
12. ru	**15,260** **(0.97%)**	27.11%	4,137 (0.86%)	37.68%	8.16	00:11:05	0.00%	0 (0.00%)	$0.00 (0.00%)
13. en	**14,729** **(0.93%)**	45.63%	6,721 (1.39%)	56.01%	6.02	00:06:12	0.00%	0 (0.00%)	$0.00 (0.00%)
14. zh-tw	**13,107** **(0.83%)**	24.01%	3,147 (0.65%)	30.65%	9.46	00:11:55	0.00%	0 (0.00%)	$0.00 (0.00%)
15. ko	**11,369** **(0.72%)**	22.16%	2,519 (0.52%)	24.87%	11.19	00:12:37	0.00%	0 (0.00%)	$0.00 (0.00%)
16. ja	**10,871** **(0.69%)**	30.47%	3,312 (0.68%)	29.45%	12.85	00:09:40	0.00%	0 (0.00%)	$0.00 (0.00%)
17. it	**10,529** **(0.67%)**	38.93%	4,099 (0.85%)	33.93%	8.76	00:09:08	0.00%	0 (0.00%)	$0.00 (0.00%)
18. pl	**10,167** **(0.64%)**	29.08%	2,957 (0.61%)	42.18%	10.12	00:09:00	0.00%	0 (0.00%)	$0.00 (0.00%)
19. nl	**9,993** **(0.63%)**	37.91%	3,788 (0.78%)	42.72%	6.11	00:07:00	0.00%	0 (0.00%)	$0.00 (0.00%)
20. fr-fr	**7,363** **(0.47%)**	25.32%	1,864 (0.39%)	54.45%	6.84	00:05:32	0.00%	0 (0.00%)	$0.00 (0.00%)
21. cs	**7,337** **(0.46%)**	29.66%	2,176 (0.45%)	40.15%	12.01	00:10:11	0.00%	0 (0.00%)	$0.00 (0.00%)
22. es-ar	**7,089** **(0.45%)**	30.10%	2,134 (0.44%)	18.18%	15.36	00:16:00	0.00%	0 (0.00%)	$0.00 (0.00%)
23. ru-ru	**6,284** **(0.40%)**	25.10%	1,577 (0.33%)	40.82%	8.22	00:08:05	0.00%	0 (0.00%)	$0.00 (0.00%)
24. it-it	**5,821** **(0.37%)**	37.12%	2,161 (0.45%)	43.67%	6.78	00:06:39	0.00%	0 (0.00%)	$0.00 (0.00%)
25. pt-pt	**4,255** **(0.27%)**	37.44%	1,593 (0.33%)	35.06%	7.29	00:07:34	0.00%	0 (0.00%)	$0.00 (0.00%)
26. sv-se	**3,496** **(0.22%)**	16.19%	566 (0.12%)	38.84%	7.49	00:08:11	0.00%	0 (0.00%)	$0.00 (0.00%)

27.	tr	**3,007** **(0.19%)**	54.14%	1,628 (0.34%)	46.69%	8.70	00:05:54	0.00%	0 (0.00%)	$0.00 (0.00%)
28.	ja-jp	**2,262** **(0.14%)**	27.94%	632 (0.13%)	48.28%	6.46	00:04:42	0.00%	0 (0.00%)	$0.00 (0.00%)
29.	sv	**1,992** **(0.13%)**	37.70%	751 (0.16%)	42.62%	5.86	00:06:24	0.00%	0 (0.00%)	$0.00 (0.00%)
30.	sk	**1,968** **(0.12%)**	29.07%	572 (0.12%)	44.92%	6.04	00:06:23	0.00%	0 (0.00%)	$0.00 (0.00%)
31.	th	**1,736** **(0.11%)**	56.45%	980 (0.20%)	50.17%	4.56	00:04:53	0.00%	0 (0.00%)	$0.00 (0.00%)
32.	vi	**1,707** **(0.11%)**	49.68%	848 (0.18%)	39.07%	7.84	00:08:17	0.00%	0 (0.00%)	$0.00 (0.00%)
33.	pt	**1,625** **(0.10%)**	42.89%	697 (0.14%)	34.83%	8.82	00:09:01	0.00%	0 (0.00%)	$0.00 (0.00%)
34.	id	**1,623** **(0.10%)**	58.72%	953 (0.20%)	52.62%	6.42	00:09:05	0.00%	0 (0.00%)	$0.00 (0.00%)
35.	ca	**1,601** **(0.10%)**	51.78%	829 (0.17%)	27.73%	8.55	00:08:51	0.00%	0 (0.00%)	$0.00 (0.00%)
36.	ko-kr	**1,316** **(0.08%)**	14.06%	185 (0.04%)	24.16%	9.70	00:11:14	0.00%	0 (0.00%)	$0.00 (0.00%)
37.	da	**1,294** **(0.08%)**	47.76%	618 (0.13%)	42.58%	6.66	00:05:44	0.00%	0 (0.00%)	$0.00 (0.00%)
38.	nb-no	**1,066** **(0.07%)**	15.76%	168 (0.03%)	40.62%	15.29	00:10:21	0.00%	0 (0.00%)	$0.00 (0.00%)
39.	es-cl	**1,011** **(0.06%)**	38.28%	387 (0.08%)	27.70%	9.23	00:10:24	0.00%	0 (0.00%)	$0.00 (0.00%)
40.	hu	**893** **(0.06%)**	68.98%	616 (0.13%)	62.15%	4.06	00:02:36	0.00%	0 (0.00%)	$0.00 (0.00%)
41.	el	**878** **(0.06%)**	63.90%	561 (0.12%)	58.31%	3.78	00:02:25	0.00%	0 (0.00%)	$0.00 (0.00%)
42.	nl-nl	**804** **(0.05%)**	61.07%	491 (0.10%)	66.42%	5.09	00:02:06	0.00%	0 (0.00%)	$0.00 (0.00%)
43.	hu-hu	**800** **(0.05%)**	64.12%	513 (0.11%)	66.62%	3.52	00:01:58	0.00%	0 (0.00%)	$0.00 (0.00%)
44.	th-th	**786** **(0.05%)**	42.11%	331 (0.07%)	41.22%	6.42	00:06:45	0.00%	0 (0.00%)	$0.00 (0.00%)
45.	es-la	**783** **(0.05%)**	29.50%	231 (0.05%)	41.51%	9.17	00:09:12	0.00%	0 (0.00%)	$0.00 (0.00%)
46.	ar	**762** **(0.05%)**	51.05%	389 (0.08%)	56.96%	3.29	00:03:04	0.00%	0 (0.00%)	$0.00 (0.00%)
47.	tr-tr	**717** **(0.05%)**	36.54%	262 (0.05%)	48.26%	4.89	00:05:30	0.00%	0 (0.00%)	$0.00 (0.00%)
48.	en-au	**634** **(0.04%)**	25.08%	159 (0.03%)	26.34%	8.41	00:09:26	0.00%	0 (0.00%)	$0.00 (0.00%)
49.	fi	**620** **(0.04%)**	41.77%	259 (0.05%)	38.06%	16.66	00:09:55	0.00%	0 (0.00%)	$0.00 (0.00%)
50.	bg	**616** **(0.04%)**	62.82%	387 (0.08%)	61.20%	4.21	00:03:49	0.00%	0 (0.00%)	$0.00 (0.00%)
51.	zh-hk	**613** **(0.04%)**	32.63%	200 (0.04%)	30.18%	5.98	00:04:49	0.00%	0 (0.00%)	$0.00 (0.00%)
52.	fi-fi	**603** **(0.04%)**	38.97%	235 (0.05%)	42.29%	7.07	00:07:41	0.00%	0 (0.00%)	$0.00 (0.00%)
53.	et	**595** **(0.04%)**	42.35%	252 (0.05%)	32.44%	5.46	00:07:02	0.00%	0 (0.00%)	$0.00 (0.00%)
54.	fil	**543** **(0.03%)**	69.43%	377 (0.08%)	70.72%	2.04	00:01:29	0.00%	0 (0.00%)	$0.00 (0.00%)
55.	el-gr	**513** **(0.03%)**	68.81%	353 (0.07%)	54.39%	5.99	00:03:45	0.00%	0 (0.00%)	$0.00 (0.00%)
56.	vi-vn	**504** **(0.03%)**	49.01%	247 (0.05%)	43.85%	5.70	00:06:18	0.00%	0 (0.00%)	$0.00 (0.00%)
57.	lt	**480** **(0.03%)**	45.62%	219 (0.05%)	45.00%	10.29	00:08:54	0.00%	0 (0.00%)	$0.00 (0.00%)
58.	he	**449** **(0.03%)**	66.37%	298 (0.06%)	59.24%	4.42	00:02:59	0.00%	0 (0.00%)	$0.00 (0.00%)
59.	es-us	**434** **(0.03%)**	44.70%	194 (0.04%)	45.39%	6.68	00:08:54	0.00%	0 (0.00%)	$0.00 (0.00%)
60.	ro	**397** **(0.03%)**	60.96%	242 (0.05%)	55.92%	5.70	00:04:27	0.00%	0 (0.00%)	$0.00 (0.00%)
61.	sl	**365** **(0.02%)**	43.84%	160 (0.03%)	45.75%	4.36	00:03:41	0.00%	0 (0.00%)	$0.00 (0.00%)
62.	no	**351** **(0.02%)**	45.58%	160 (0.03%)	41.03%	7.67	00:05:19	0.00%	0 (0.00%)	$0.00 (0.00%)
63.	es-xl	**297** **(0.02%)**	13.80%	41 (0.01%)	34.01%	16.78	00:10:30	0.00%	0 (0.00%)	$0.00 (0.00%)
64.	lv	**279** **(0.02%)**	35.13%	98 (0.02%)	36.92%	6.94	00:06:19	0.00%	0 (0.00%)	$0.00 (0.00%)
65.	hr-hr	**270** **(0.02%)**	81.85%	221 (0.05%)	41.85%	4.40	00:02:30	0.00%	0 (0.00%)	$0.00 (0.00%)
66.	de-at	**251** **(0.02%)**	35.46%	89 (0.02%)	23.51%	9.35	00:11:08	0.00%	0 (0.00%)	$0.00 (0.00%)

67.	hr	**233**	**(0.01%)**	68.24%	159	(0.03%)	62.23%	2.88	00:01:38	0.00%	0	(0.00%)	$0.00	(0.00%)
68.	da-dk	**232**	**(0.01%)**	48.71%	113	(0.02%)	68.10%	2.91	00:02:19	0.00%	0	(0.00%)	$0.00	(0.00%)
69.	es-pe	**215**	**(0.01%)**	11.16%	24	(0.00%)	8.37%	18.13	00:20:19	0.00%	0	(0.00%)	$0.00	(0.00%)
70.	ro-ro	**215**	**(0.01%)**	49.30%	106	(0.02%)	45.12%	9.48	00:09:43	0.00%	0	(0.00%)	$0.00	(0.00%)
71.	uk	**209**	**(0.01%)**	68.90%	144	(0.03%)	59.33%	4.10	00:02:51	0.00%	0	(0.00%)	$0.00	(0.00%)
72.	(not set)	**197**	**(0.01%)**	73.60%	145	(0.03%)	38.58%	3.98	00:03:28	0.00%	0	(0.00%)	$0.00	(0.00%)
73.	cs-cz	**194**	**(0.01%)**	65.46%	127	(0.03%)	66.49%	2.96	00:02:29	0.00%	0	(0.00%)	$0.00	(0.00%)
74.	sr	**174**	**(0.01%)**	80.46%	140	(0.03%)	65.52%	3.45	00:02:19	0.00%	0	(0.00%)	$0.00	(0.00%)
75.	es-pa	**171**	**(0.01%)**	22.22%	38	(0.01%)	19.88%	11.39	00:09:53	0.00%	0	(0.00%)	$0.00	(0.00%)
76.	ja-jp-mac	**167**	**(0.01%)**	26.35%	44	(0.01%)	16.77%	5.62	00:06:47	0.00%	0	(0.00%)	$0.00	(0.00%)
77.	pl-pl	**160**	**(0.01%)**	56.25%	90	(0.02%)	72.50%	3.49	00:02:20	0.00%	0	(0.00%)	$0.00	(0.00%)
78.	es-co	**153**	**(0.01%)**	29.41%	45	(0.01%)	69.93%	2.94	00:02:14	0.00%	0	(0.00%)	$0.00	(0.00%)
79.	en-za	**140**	**(0.01%)**	71.43%	100	(0.02%)	67.14%	3.62	00:02:55	0.00%	0	(0.00%)	$0.00	(0.00%)
80.	en_gb	**130**	**(0.01%)**	83.08%	108	(0.02%)	74.62%	1.92	00:01:05	0.00%	0	(0.00%)	$0.00	(0.00%)
81.	ar-sa	**117**	**(0.01%)**	73.50%	86	(0.02%)	65.81%	2.10	00:01:10	0.00%	0	(0.00%)	$0.00	(0.00%)
82.	nb	**111**	**(0.01%)**	72.97%	81	(0.02%)	69.37%	2.40	00:00:58	0.00%	0	(0.00%)	$0.00	(0.00%)
83.	en_us	**99**	**(0.01%)**	87.88%	87	(0.02%)	68.69%	1.94	00:01:04	0.00%	0	(0.00%)	$0.00	(0.00%)
84.	en-ca	**94**	**(0.01%)**	79.79%	75	(0.02%)	50.00%	4.10	00:03:21	0.00%	0	(0.00%)	$0.00	(0.00%)
85.	c	**91**	**(0.01%)**	98.90%	90	(0.02%)	95.60%	1.22	00:00:13	0.00%	0	(0.00%)	$0.00	(0.00%)
86.	es-uy	**85**	**(0.01%)**	62.35%	53	(0.01%)	11.76%	16.92	00:09:45	0.00%	0	(0.00%)	$0.00	(0.00%)
87.	ca-es	**77**	**(0.00%)**	79.22%	61	(0.01%)	58.44%	3.34	00:02:37	0.00%	0	(0.00%)	$0.00	(0.00%)
88.	en-in	**69**	**(0.00%)**	92.75%	64	(0.01%)	75.36%	1.65	00:00:42	0.00%	0	(0.00%)	$0.00	(0.00%)
89.	es-ve	**59**	**(0.00%)**	40.68%	24	(0.00%)	28.81%	17.68	00:21:41	0.00%	0	(0.00%)	$0.00	(0.00%)
90.	es-bo	**52**	**(0.00%)**	19.23%	10	(0.00%)	3.85%	78.08	00:34:39	0.00%	0	(0.00%)	$0.00	(0.00%)
91.	es-cr	**52**	**(0.00%)**	32.69%	17	(0.00%)	19.23%	10.00	00:12:46	0.00%	0	(0.00%)	$0.00	(0.00%)
92.	fa-ir	**51**	**(0.00%)**	50.98%	26	(0.01%)	56.86%	3.80	00:03:54	0.00%	0	(0.00%)	$0.00	(0.00%)
93.	fr-ch	**50**	**(0.00%)**	50.00%	25	(0.01%)	34.00%	7.28	00:04:20	0.00%	0	(0.00%)	$0.00	(0.00%)
94.	es-ec	**48**	**(0.00%)**	75.00%	36	(0.01%)	37.50%	17.21	00:14:37	0.00%	0	(0.00%)	$0.00	(0.00%)
95.	he-il	**47**	**(0.00%)**	51.06%	24	(0.00%)	68.09%	1.96	00:01:07	0.00%	0	(0.00%)	$0.00	(0.00%)
96.	es-ni	**43**	**(0.00%)**	18.60%	8	(0.00%)	30.23%	22.00	00:11:58	0.00%	0	(0.00%)	$0.00	(0.00%)
97.	fr-ca	**41**	**(0.00%)**	63.41%	26	(0.01%)	36.59%	46.32	00:10:40	0.00%	0	(0.00%)	$0.00	(0.00%)
98.	de-ch	**40**	**(0.00%)**	70.00%	28	(0.01%)	57.50%	2.52	00:01:07	0.00%	0	(0.00%)	$0.00	(0.00%)
99.	en-nz	**36**	**(0.00%)**	75.00%	27	(0.01%)	72.22%	3.00	00:01:19	0.00%	0	(0.00%)	$0.00	(0.00%)
100.	kau	**33**	**(0.00%)**	93.94%	31	(0.01%)	72.73%	1.48	00:00:42	0.00%	0	(0.00%)	$0.00	(0.00%)

Rows 1 - 100 of 177

© 2014 Google
