# Supplementary material for: Trends in access of plant biodiversity data revealed by Google Analytics
Source: Biodivers Data J. 2014 Nov 11;(2):e1558. doi: 10.3897/BDJ.2.e1558 (PMC4238075; doi:10.3897/BDJ.2.e1558)
Supplement: Supplementary material 19 — Tropicos by year for language [file biodiversity_data_journal-2-e1558-s019.pdf]

Google Analytics

http://www.tropicos.org - http://www.tro… www.tropicos.org [Go to this report]

Language

Jun 1, 2013 - Jun 1, 2014

All Sessions
100.00%

+ Add Segment

Explorer

Summary

Language	Acquisition			Behavior			Conversions		
	Sessions	% New Sessions	New Users	Bounce Rate	Pages / Session	Avg. Session Duration	Goal Conversion Rate	Goal Completions	Goal Value
	1,638,764 % of Total: 100.00% (1,638,764)	33.66% Site Avg: 33.60% (0.16%)	551,605 % of Total: 100.16% (550,703)	33.01% Site Avg: 33.01% (0.00%)	11.32 Site Avg: 11.32 (0.00%)	00:12:07 Site Avg: 00:12:07 (0.00%)	0.00% Site Avg: 0.00% (0.00%)	0 % of Total: 0.00% (0)	$0.00 % of Total: 0.00% ($0.00)
1. en-us	**566,081** **(34.54%)**	38.86%	220,000 (39.88%)	38.41%	10.91	00:10:58	0.00%	0 (0.00%)	$0.00 (0.00%)
2. es	**294,958** **(18.00%)**	27.87%	82,218 (14.91%)	26.29%	14.36	00:15:10	0.00%	0 (0.00%)	$0.00 (0.00%)
3. pt-br	**180,655** **(11.02%)**	27.48%	49,638 (9.00%)	24.02%	9.82	00:11:50	0.00%	0 (0.00%)	$0.00 (0.00%)
4. es-es	**134,068** **(8.18%)**	29.97%	40,178 (7.28%)	30.91%	13.39	00:13:49	0.00%	0 (0.00%)	$0.00 (0.00%)
5. fr	**79,440** **(4.85%)**	28.61%	22,724 (4.12%)	26.64%	12.59	00:16:05	0.00%	0 (0.00%)	$0.00 (0.00%)
6. zh-cn	**43,169** **(2.63%)**	23.32%	10,067 (1.83%)	23.58%	11.14	00:13:46	0.00%	0 (0.00%)	$0.00 (0.00%)
7. de-de	**42,734** **(2.61%)**	37.58%	16,061 (2.91%)	35.85%	8.99	00:11:45	0.00%	0 (0.00%)	$0.00 (0.00%)
8. en-gb	**32,600** **(1.99%)**	38.03%	12,398 (2.25%)	41.44%	8.15	00:07:49	0.00%	0 (0.00%)	$0.00 (0.00%)
9. es-419	**29,290** **(1.79%)**	28.77%	8,428 (1.53%)	27.58%	12.78	00:13:39	0.00%	0 (0.00%)	$0.00 (0.00%)
10. de	**22,002** **(1.34%)**	42.30%	9,307 (1.69%)	39.30%	7.55	00:09:49	0.00%	0 (0.00%)	$0.00 (0.00%)
11. ru	**19,164** **(1.17%)**	26.14%	5,009 (0.91%)	42.46%	7.87	00:10:09	0.00%	0 (0.00%)	$0.00 (0.00%)
12. es-mx	**17,983** **(1.10%)**	28.84%	5,187 (0.94%)	19.44%	16.54	00:15:48	0.00%	0 (0.00%)	$0.00 (0.00%)
13. fr-fr	**13,005** **(0.79%)**	34.79%	4,525 (0.82%)	45.37%	8.38	00:06:57	0.00%	0 (0.00%)	$0.00 (0.00%)
14. zh-tw	**11,458** **(0.70%)**	31.97%	3,663 (0.66%)	32.90%	7.66	00:09:32	0.00%	0 (0.00%)	$0.00 (0.00%)
15. en	**11,334** **(0.69%)**	59.93%	6,792 (1.23%)	57.12%	5.22	00:05:16	0.00%	0 (0.00%)	$0.00 (0.00%)
16. it	**10,534** **(0.64%)**	37.44%	3,944 (0.72%)	39.88%	6.44	00:07:03	0.00%	0 (0.00%)	$0.00 (0.00%)
17. pl	**9,951** **(0.61%)**	31.50%	3,135 (0.57%)	37.99%	11.91	00:11:31	0.00%	0 (0.00%)	$0.00 (0.00%)
18. it-it	**8,530** **(0.52%)**	41.02%	3,499 (0.63%)	43.33%	8.36	00:08:20	0.00%	0 (0.00%)	$0.00 (0.00%)
19. ko	**8,158** **(0.50%)**	25.78%	2,103 (0.38%)	23.17%	12.90	00:15:09	0.00%	0 (0.00%)	$0.00 (0.00%)
20. ja	**7,958** **(0.49%)**	35.66%	2,838 (0.51%)	33.74%	8.48	00:07:19	0.00%	0 (0.00%)	$0.00 (0.00%)
21. nl	**7,402** **(0.45%)**	41.21%	3,050 (0.55%)	45.56%	8.13	00:08:55	0.00%	0 (0.00%)	$0.00 (0.00%)
22. cs	**7,065** **(0.43%)**	32.05%	2,264 (0.41%)	43.45%	9.50	00:08:19	0.00%	0 (0.00%)	$0.00 (0.00%)
23. ru-ru	**6,920** **(0.42%)**	28.54%	1,975 (0.36%)	36.66%	7.77	00:09:19	0.00%	0 (0.00%)	$0.00 (0.00%)
24. ja-jp	**6,170** **(0.38%)**	33.42%	2,062 (0.37%)	36.35%	7.67	00:07:00	0.00%	0 (0.00%)	$0.00 (0.00%)
25. pt-pt	**5,616** **(0.34%)**	34.08%	1,914 (0.35%)	33.19%	9.89	00:10:06	0.00%	0 (0.00%)	$0.00 (0.00%)
26. es-ar	**5,008** **(0.31%)**	36.50%	1,828 (0.33%)	23.20%	13.14	00:12:30	0.00%	0 (0.00%)	$0.00 (0.00%)

27.	ko-kr	**4,497** **(0.27%)**	28.40%	1,277 (0.23%)	28.11%	12.64	00:12:56	0.00%	0 (0.00%)	$0.00 (0.00%)
28.	sv-se	**3,962** **(0.24%)**	20.75%	822 (0.15%)	27.49%	7.36	00:08:58	0.00%	0 (0.00%)	$0.00 (0.00%)
29.	nl-nl	**2,985** **(0.18%)**	52.36%	1,563 (0.28%)	56.31%	4.64	00:02:58	0.00%	0 (0.00%)	$0.00 (0.00%)
30.	tr	**2,968** **(0.18%)**	57.21%	1,698 (0.31%)	45.75%	11.44	00:05:42	0.00%	0 (0.00%)	$0.00 (0.00%)
31.	vi	**2,905** **(0.18%)**	49.91%	1,450 (0.26%)	45.71%	5.49	00:06:49	0.00%	0 (0.00%)	$0.00 (0.00%)
32.	ca	**2,689** **(0.16%)**	38.38%	1,032 (0.19%)	20.97%	12.51	00:20:44	0.00%	0 (0.00%)	$0.00 (0.00%)
33.	id	**2,656** **(0.16%)**	51.92%	1,379 (0.25%)	49.40%	5.55	00:08:43	0.00%	0 (0.00%)	$0.00 (0.00%)
34.	th	**2,561** **(0.16%)**	52.83%	1,353 (0.25%)	50.84%	4.62	00:05:57	0.00%	0 (0.00%)	$0.00 (0.00%)
35.	cs-cz	**1,724** **(0.11%)**	24.13%	416 (0.08%)	28.19%	13.79	00:14:11	0.00%	0 (0.00%)	$0.00 (0.00%)
36.	sk	**1,476** **(0.09%)**	38.89%	574 (0.10%)	51.15%	5.09	00:05:27	0.00%	0 (0.00%)	$0.00 (0.00%)
37.	sv	**1,466** **(0.09%)**	37.24%	546 (0.10%)	46.18%	6.27	00:06:18	0.00%	0 (0.00%)	$0.00 (0.00%)
38.	tr-tr	**1,398** **(0.09%)**	52.36%	732 (0.13%)	45.49%	10.66	00:06:33	0.00%	0 (0.00%)	$0.00 (0.00%)
39.	hu	**1,386** **(0.08%)**	55.56%	770 (0.14%)	57.14%	4.16	00:02:45	0.00%	0 (0.00%)	$0.00 (0.00%)
40.	es-us	**1,226** **(0.07%)**	53.59%	657 (0.12%)	51.47%	4.92	00:05:20	0.00%	0 (0.00%)	$0.00 (0.00%)
41.	th-th	**1,202** **(0.07%)**	39.27%	472 (0.09%)	50.92%	4.03	00:05:13	0.00%	0 (0.00%)	$0.00 (0.00%)
42.	hu-hu	**1,133** **(0.07%)**	62.22%	705 (0.13%)	59.22%	3.05	00:02:03	0.00%	0 (0.00%)	$0.00 (0.00%)
43.	da	**1,086** **(0.07%)**	51.84%	563 (0.10%)	48.25%	6.63	00:05:32	0.00%	0 (0.00%)	$0.00 (0.00%)
44.	es-cl	**1,086** **(0.07%)**	37.11%	403 (0.07%)	31.31%	9.32	00:10:13	0.00%	0 (0.00%)	$0.00 (0.00%)
45.	el	**976** **(0.06%)**	54.20%	529 (0.10%)	57.38%	3.44	00:02:10	0.00%	0 (0.00%)	$0.00 (0.00%)
46.	ar	**929** **(0.06%)**	73.41%	682 (0.12%)	62.43%	2.63	00:02:09	0.00%	0 (0.00%)	$0.00 (0.00%)
47.	en-au	**842** **(0.05%)**	31.24%	263 (0.05%)	39.67%	5.16	00:06:08	0.00%	0 (0.00%)	$0.00 (0.00%)
48.	zh-hk	**786** **(0.05%)**	37.66%	296 (0.05%)	36.13%	6.23	00:06:09	0.00%	0 (0.00%)	$0.00 (0.00%)
49.	es-la	**777** **(0.05%)**	34.62%	269 (0.05%)	37.84%	9.56	00:10:19	0.00%	0 (0.00%)	$0.00 (0.00%)
50.	lt	**760** **(0.05%)**	39.34%	299 (0.05%)	36.05%	11.64	00:13:39	0.00%	0 (0.00%)	$0.00 (0.00%)
51.	fi-fi	**733** **(0.04%)**	41.88%	307 (0.06%)	55.80%	6.14	00:02:45	0.00%	0 (0.00%)	$0.00 (0.00%)
52.	el-gr	**677** **(0.04%)**	66.62%	451 (0.08%)	64.84%	4.21	00:03:02	0.00%	0 (0.00%)	$0.00 (0.00%)
53.	fil	**673** **(0.04%)**	69.24%	466 (0.08%)	55.42%	2.90	00:04:05	0.00%	0 (0.00%)	$0.00 (0.00%)
54.	pt	**625** **(0.04%)**	44.16%	276 (0.05%)	36.32%	8.56	00:09:55	0.00%	0 (0.00%)	$0.00 (0.00%)
55.	nb-no	**605** **(0.04%)**	44.63%	270 (0.05%)	47.11%	3.77	00:04:14	0.00%	0 (0.00%)	$0.00 (0.00%)
56.	da-dk	**604** **(0.04%)**	62.91%	380 (0.07%)	58.61%	4.15	00:03:07	0.00%	0 (0.00%)	$0.00 (0.00%)
57.	ro	**575** **(0.04%)**	56.35%	324 (0.06%)	52.70%	8.15	00:06:13	0.00%	0 (0.00%)	$0.00 (0.00%)
58.	bg	**527** **(0.03%)**	68.50%	361 (0.07%)	73.06%	2.76	00:01:22	0.00%	0 (0.00%)	$0.00 (0.00%)
59.	he	**472** **(0.03%)**	56.78%	268 (0.05%)	50.64%	5.15	00:03:31	0.00%	0 (0.00%)	$0.00 (0.00%)
60.	ca-es	**464** **(0.03%)**	31.03%	144 (0.03%)	28.02%	9.34	00:14:17	0.00%	0 (0.00%)	$0.00 (0.00%)
61.	et	**462** **(0.03%)**	43.94%	203 (0.04%)	42.64%	6.50	00:06:09	0.00%	0 (0.00%)	$0.00 (0.00%)
62.	vi-vn	**440** **(0.03%)**	64.55%	284 (0.05%)	61.14%	4.59	00:03:48	0.00%	0 (0.00%)	$0.00 (0.00%)
63.	uk	**429** **(0.03%)**	53.61%	230 (0.04%)	51.75%	3.89	00:04:06	0.00%	0 (0.00%)	$0.00 (0.00%)
64.	es-xl	**377** **(0.02%)**	20.16%	76 (0.01%)	45.62%	8.90	00:06:50	0.00%	0 (0.00%)	$0.00 (0.00%)
65.	fi	**364** **(0.02%)**	58.52%	213 (0.04%)	54.40%	3.50	00:02:03	0.00%	0 (0.00%)	$0.00 (0.00%)
66.	pl-pl	**353** **(0.02%)**	76.77%	271 (0.05%)	69.41%	3.24	00:01:29	0.00%	0 (0.00%)	$0.00 (0.00%)

67.	hr	**310** **(0.02%)**	63.23%	196 (0.04%)	63.23%	5.25	00:04:06	0.00%	0 (0.00%)	$0.00 (0.00%)
68.	c	**302** **(0.02%)**	98.34%	297 (0.05%)	97.35%	1.06	00:00:02	0.00%	0 (0.00%)	$0.00 (0.00%)
69.	hr-hr	**294** **(0.02%)**	74.83%	220 (0.04%)	42.52%	4.28	00:03:12	0.00%	0 (0.00%)	$0.00 (0.00%)
70.	ro-ro	**286** **(0.02%)**	48.95%	140 (0.03%)	60.84%	3.24	00:03:11	0.00%	0 (0.00%)	$0.00 (0.00%)
71.	lv	**258** **(0.02%)**	62.02%	160 (0.03%)	64.73%	10.56	00:05:23	0.00%	0 (0.00%)	$0.00 (0.00%)
72.	sl	**235** **(0.01%)**	74.89%	176 (0.03%)	68.94%	2.49	00:01:11	0.00%	0 (0.00%)	$0.00 (0.00%)
73.	sr	**231** **(0.01%)**	79.65%	184 (0.03%)	64.94%	2.45	00:01:22	0.00%	0 (0.00%)	$0.00 (0.00%)
74.	nb	**229** **(0.01%)**	65.94%	151 (0.03%)	62.01%	3.05	00:01:47	0.00%	0 (0.00%)	$0.00 (0.00%)
75.	es-pa	**186** **(0.01%)**	31.72%	59 (0.01%)	27.42%	6.02	00:06:18	0.00%	0 (0.00%)	$0.00 (0.00%)
76.	en-ca	**183** **(0.01%)**	52.46%	96 (0.02%)	48.09%	3.87	00:11:18	0.00%	0 (0.00%)	$0.00 (0.00%)
77.	en-za	**178** **(0.01%)**	65.73%	117 (0.02%)	60.11%	3.16	00:03:51	0.00%	0 (0.00%)	$0.00 (0.00%)
78.	es-co	**177** **(0.01%)**	33.90%	60 (0.01%)	22.03%	11.92	00:14:41	0.00%	0 (0.00%)	$0.00 (0.00%)
79.	id-id	**164** **(0.01%)**	65.85%	108 (0.02%)	57.32%	3.97	00:04:43	0.00%	0 (0.00%)	$0.00 (0.00%)
80.	en_gb	**145** **(0.01%)**	83.45%	121 (0.02%)	69.66%	2.12	00:02:06	0.00%	0 (0.00%)	$0.00 (0.00%)
81.	he-il	**139** **(0.01%)**	61.15%	85 (0.02%)	59.71%	5.40	00:03:42	0.00%	0 (0.00%)	$0.00 (0.00%)
82.	bg-bg	**121** **(0.01%)**	17.36%	21 (0.00%)	28.10%	11.00	00:09:34	0.00%	0 (0.00%)	$0.00 (0.00%)
83.	no	**119** **(0.01%)**	45.38%	54 (0.01%)	52.94%	7.08	00:03:25	0.00%	0 (0.00%)	$0.00 (0.00%)
84.	es-ni	**114** **(0.01%)**	5.26%	6 (0.00%)	18.42%	13.82	00:15:47	0.00%	0 (0.00%)	$0.00 (0.00%)
85.	ar-sa	**110** **(0.01%)**	75.45%	83 (0.02%)	62.73%	3.25	00:02:57	0.00%	0 (0.00%)	$0.00 (0.00%)
86.	sl-si	**107** **(0.01%)**	45.79%	49 (0.01%)	55.14%	2.59	00:01:18	0.00%	0 (0.00%)	$0.00 (0.00%)
87.	es-pe	**103** **(0.01%)**	63.11%	65 (0.01%)	33.01%	11.94	00:08:17	0.00%	0 (0.00%)	$0.00 (0.00%)
88.	en_us	**89** **(0.01%)**	76.40%	68 (0.01%)	66.29%	1.73	00:01:41	0.00%	0 (0.00%)	$0.00 (0.00%)
89.	en-in	**86** **(0.01%)**	83.72%	72 (0.01%)	70.93%	2.05	00:02:50	0.00%	0 (0.00%)	$0.00 (0.00%)
90.	sk-sk	**80** **(0.00%)**	68.75%	55 (0.01%)	83.75%	1.40	00:00:13	0.00%	0 (0.00%)	$0.00 (0.00%)
91.	(not set)	**74** **(0.00%)**	79.73%	59 (0.01%)	67.57%	4.70	00:04:32	0.00%	0 (0.00%)	$0.00 (0.00%)
92.	fr-ch	**73** **(0.00%)**	28.77%	21 (0.00%)	30.14%	6.30	00:06:36	0.00%	0 (0.00%)	$0.00 (0.00%)
93.	de-at	**70** **(0.00%)**	58.57%	41 (0.01%)	60.00%	7.27	00:05:03	0.00%	0 (0.00%)	$0.00 (0.00%)
94.	et-ee	**70** **(0.00%)**	28.57%	20 (0.00%)	35.71%	5.91	00:04:40	0.00%	0 (0.00%)	$0.00 (0.00%)
95.	de-ch	**65** **(0.00%)**	86.15%	56 (0.01%)	69.23%	1.98	00:00:24	0.00%	0 (0.00%)	$0.00 (0.00%)
96.	es-cr	**62** **(0.00%)**	32.26%	20 (0.00%)	25.81%	10.94	00:11:23	0.00%	0 (0.00%)	$0.00 (0.00%)
97.	fa-ir	**59** **(0.00%)**	52.54%	31 (0.01%)	45.76%	5.98	00:07:09	0.00%	0 (0.00%)	$0.00 (0.00%)
98.	ja-jp-mac	**56** **(0.00%)**	23.21%	13 (0.00%)	10.71%	7.18	00:07:22	0.00%	0 (0.00%)	$0.00 (0.00%)
99.	es-ec	**55** **(0.00%)**	47.27%	26 (0.00%)	30.91%	11.40	00:13:49	0.00%	0 (0.00%)	$0.00 (0.00%)
100.	fa	**52** **(0.00%)**	55.77%	29 (0.01%)	34.62%	5.92	00:11:19	0.00%	0 (0.00%)	$0.00 (0.00%)

Rows 1 - 100 of 217

© 2014 Google
